# A Subset Screen of the Compounds Australia Scaffold Library Identifies 7-Acylaminodibenzoxazepinones as Potent and Selective Hits for Anti-*Giardia* Drug Discovery

**DOI:** 10.3390/biomedicines10123182

**Published:** 2022-12-08

**Authors:** Christopher J. S. Hart, Andrew G. Riches, Snigdha Tiash, Erin Clapper, Soumya Ramu, Johannes Zuegg, John H. Ryan, Tina S. Skinner-Adams

**Affiliations:** 1 Department of Microbiology and Molecular Genetics, University of California Davis, Davis, CA 95616, USA; 2 Commonwealth Scientific and Industrial Research Organization, Biomedical Manufacturing, Clayton, VIC 3168, Australia; 3 Griffith Institute for Drug Discovery, Griffith University, Nathan, QLD 4111, Australia; 4 Institute for Molecular Bioscience, The University of Queensland, St Lucia, QLD 4072, Australia

**Keywords:** *Giardia duodenalis*, giardiasis, drug discovery, Compounds Australia Scaffold Library, acylaminodibenzoxazepinones

## Abstract

On an annual basis the flagellate protozoan, *Giardia duodenalis*, is responsible for an estimated one billion human infections of which approximately two hundred million cause disease. However, the treatment of *Giardia* infections is reliant on a small group of chemotherapeutic classes that have a broad spectrum of antimicrobial activity and increasing treatment failure rates. To improve this situation, we need new drugs. In this study we screened the Compounds Australia Scaffolds Library for compounds with potent and selective activity against these parasites. Unlike previous drug discovery efforts that have focused on drug repurposing, this library is comprised of commercially available synthetic compounds arranged into lead-like scaffolds to facilitate structure activity relationship assessments and de novo drug discovery. A screen of 2451 compounds in this library identified 40 hits (>50% inhibitory activity at 10 µM, over 48 h). Secondary testing identified three compounds with IC_50_ values <1 μM and >50-fold selectivity for parasites over mammalian cells and a hit series, CL9406, comprising compounds with potent (lowest IC_50_ 180 nM) and selective activity for *Giardia* parasites. The most promising compound in this series, SN00797640, displayed selective activity against assemblage A, B, and metronidazole resistant parasites which was parasiticidal (minimum lethal concentration 625 nM) and synergistic with albendazole. SN00797640 was well-tolerated when administered to mice at doses of 50 mg/kg daily for three days paving the way for pre-clinical in vivo activity assessment.

## 1. Introduction

Giardiasis is a common but neglected cause of intestinal illness world-wide. *Giardia duodenalis* (aka *G. lamblia* and *G. intestinalis*), a flagellate protozoan parasite that colonizes the proximal small intestine of its host causes giardiasis. Humans become infected with parasites when they ingest environmentally stable cysts via contaminated food, water or by direct fecal-oral contact. Two major ancestral lineages of *G. duodenalis* (assemblage A and B) commonly infect humans, both are associated with disease and can be zoonotic (reviewed in [1]).

While often self-resolving, infections with *G. duodenalis* can be severe, resulting in a diverse range of clinical manifestations including diarrhea, abdominal pain, nausea, anemia, vomiting, zinc deficiency, iron deficiency, weight loss and failure to thrive in children [2]. Infections in children can also cause poor cognitive performance [2,3] and there is emerging evidence that people infected with *G. duodenalis* are at risk of developing post-infectious disorders including irritable bowel syndrome and chronic fatigue [4,5,6]. While these diverse pathologies are incompletely understood, they are likely linked to parasite and host factors, including the impact of parasites and treatments on the host gut microbiota [7,8,9].

The control and treatment of *Giardia* infections in humans relies on education and chemotherapy. There is no vaccine. In addition, chemotherapeutic options rely on a small group of compound classes that have broad antimicrobial activity and increasing failure rates. As an example, the front-line 5-nitroimidazoles, including metronidazole and tinidazole have reported clinical failure rates of 40–70% (reviewed in [10]) and activity against a wide-range of anaerobic gut microbiota [11,12]. Metronidazole is also associated with side-effects which together with its unpleasant taste and long treatment schedule (three times/day, for 5–7 days [13]) can result in poor compliance, rapid re-infection, and treatment failure. Alternative drugs including the benzimidazoles and nitazoxanide also have broad spectrum antimicrobial activity, with the benzimidazoles demonstrating variable treatment efficacy rates (25–90% for albendazole [14]) and nitazoxanide demonstrating activity concerningly like the 5-nitroimidazoles (reviewed in [1]). To improve the health and well-being of millions globally, we need new anti-*Giardia* agents with improved potency and selective activity to combat these parasites.

The identification of new anti-*Giardia* compounds has historically focused on drug repurposing. This approach has been attractive given the neglected status of this disease and the limited funds available for de novo drug discovery and development. However, it has had limited success in recent years, identifying a comparatively small number of new active agents (reviewed in [1]). In addition, many of the compounds that have been identified with in vivo activity, including auranofin, disulfiram and fumagillin have known liabilities including broad antimicrobial activity, toxicity, and contraindications in specific populations (reviewed in [1]). 

In contrast to prior repurposing studies, the goal of the current study was to identify new selective, anti-*Giardia* compounds by screening a synthetic scaffolds library specifically designed to facilitate de novo drug discovery efforts. This library, the Compounds Australia Scaffolds Library (previously known as The Queensland Compound Library), contained >30,000 compounds, strategically chosen for their lead-like scaffolds and representation of “developable” chemical diversity [15]. Importantly, each compound within the library belonged to one of the ~1200 core scaffolds, allowing preliminary structure activity relationship assessments. The arrangement of the library into core scaffolds also facilitated the screening of a compound subset representative of each chemotype, a strategy that significantly reduced the cost of this de novo drug discovery effort.

## 2. Materials and Methods

### 2.1. Cell Lines and Cultivation

Metronidazole-sensitive *G. duodenalis* assemblage B (BRIS/91/HEPU/1279 [16]) and assemblage A (BRIS/87/HEPU/713 [17]) parasites were maintained essentially as previously described in 8 mL sealed borosilicate cultures tubes, using modified Keisters TYI-S-33 medium supplemented with 10% heat inactivated fetal bovine serum, 100 units/mL penicillin, and 100 µg/mL streptomycin [18,19]. Metronidazole resistant *G. duodenalis* assemblage B (BRIS/91/HEPU/1279m1 [20]) parasites were maintained in the presence of 15 µM metronidazole which was removed two days prior to compound activity assessments. Neonatal foreskin fibroblast (NFF) cells were maintained in RPMI-1640 medium supplemented with 10% foetal bovine serum, 100 units/mL penicillin, and 100 µg/mL streptomycin, in cell culture flasks (Corning, USA). Cation-adjusted Mueller Hinton broth was used to maintain methicillin-resistant *Staphylococcus aureus*, *Escherichia coli* (ATCC 25922), *Acinetobacter baumannii* (ATCC 19606) and *Klebsiella pneumoniae* (ATCC 700603) at 37 °C as previously described [21].

### 2.2. Compounds

A subset of the Compound Australia’s Scaffold Library (>two randomly selected compounds per scaffold; 2451 compounds), was provided by Compounds Australia (Griffith University, Nathan, Brisbane Australia) in 100% DMSO as 5 mM stocks. Analogues of prioritized hits were also provided by Compounds Australia (Griffith University, Nathan, Brisbane Australia) in 100% DMSO as 5 mM stocks. Compounds Australia compounds were stored under robust environmental conditions in assay ready plate format. SN00797640 (M013-0086) and SN00776497 (ZZ22822646) were repurchased from ChemDiv (San Diego, CA, USA) and Enamine (Kyiv, Ukraine) respectively for additional activity assays. Additional quantities of SN00797640 were also synthesized for follow up activity studies as recently described [22].

### 2.3. Primary Giardia Screen Compounds

Each compound was assessed in singlicate, at a final concentration of 10 μM (0.4 μL of a 5 mM stock; diluted to a final volume of 200 μL in culture medium; 0.2% DMSO). All compounds and controls (0.4 μL DMSO and 0.4 μL 5 mM albendazole; final 10 μM) were plated by Compounds Australia using an Echo acoustic dispenser (Labcyte, Sunnyvale, CA, USA) before the addition of media inoculated with BRIS/91/HEPU/1279 trophozoites (200 μL final volume; 1.5 × 10^4^ trophozoites per well). Plates were incubated in sealed chambers filled with 3% O_2_, 5% CO_2_ in N_2_ and growth was assessed at 24 and 48 h using live cell imaging and automated counting as previously described [23]. Growth inhibition was calculated by subtracting media only background counts and determining percentage growth relative to vehicle (0.2% DMSO) controls. The reliability of these assays was assessed by calculating Z’ factors for each plate as previously described [24]. Growth inhibitory activity was reassessed (in singlicate) if compounds demonstrated ≥50% growth inhibition or if they belonged to a scaffold of interest.

### 2.4. Giardia Dose Response Assays

The dose response activity of compounds that inhibited parasite growth by >50% in screening assays was assessed to determine IC_50_ values. In these assays, compounds were serially diluted (8–12-point doubling dilutions in technical duplicate) in DMSO control media, with all test wells inoculated with parasites (1.5 × 10^4^ parasites for BRIS/91/HEPU/1279 and BRIS/91/HEPU/1279m1, or 3 × 10^3^ parasites for BRIS/87/HEPU/713). Albendazole (positive control) plates were run with each experiment. All plates were sealed in culture chambers and gassed with 3% O_2_, 5% CO_2_ in N_2_ and incubated at 37 °C for 48 h, with parasites imaged and enumerated at 24 and 48 h [23]. Growth inhibition was calculated as a percentage relative to vehicle controls minus any background as described above and IC_50_ values were determined using log-linear interpolation as previously described [25]. All assays were performed at least twice, with data displayed as mean IC_50_ ± standard derivation. Statistical comparison of IC_50_ values when required were performed using Student’s *t*-test.

### 2.5. Mammalian Cell Cytotoxicity Assays

The selectivity of compounds of interest was assessed against NFF cells. Assays were performed using sulforhodamine B and included media only, negative (no compound, no DMSO), and vehicle (DMSO) controls as previously described [26]. A chloroquine control plate was included in each assay. Due to limited compound availability, initial hits and selected scaffold compounds were tested in duplicate against NFF only in dose response titrations from 10 µM (compounds displaying sub or low μM IC_50_ against BRIS/91/HEPU/1279) or at a single concentration of 20 µM (compounds displaying BRIS/91/HEPU/1279 IC_50_ >1 μM). Repurchased SN00797640 was tested in dose–response from 100 µM against NFF. Selectivity indices (SI) were calculated by dividing mammalian cell IC_50_ values by *G. duodenalis* BRIS/91/HEPU/1279 IC_50_ values.

### 2.6. Anti-Bacterial Activity Assays

The activity of compounds against methicillin resistant *Staphylococcus aureus* (ATCC 43300), *Escherichia coli* (ATCC 25922), *Acinetobacter baumannii* (ATCC 19606) and *Klebsiella pneumoniae* (ATCC 700603) was assessed at 50 µM in duplicate as previously described [21]. In brief, mid-log phase bacteria cultures were added to 384-well microtiter plate wells containing compound to final cell density of 5 × 10^5^ CFU/mL in 50 µL. Plates were then covered and incubated at 37 °C for 18 h without shaking. Inhibition of bacterial growth was determined by measuring absorbance at 600 nm (Tecan M1000 Pro monochromator plate reader) and calculating percentage growth inhibition relative to the negative media and bacteria without inhibitors. Vancomycin hydrochloride (Sigma-Aldrich an affiliate of Merck KGaA, Darmstadt, Germany, #861987) and colistin sulfate (Sigma-Aldrich an affiliate of Merck KGaA, Darmstadt, Germany, C4461) were on each plate as positive controls for Gram-positive (*S. aureus*) and Gram-negative (*E. coli*, *A. baumannii* and *K. pneumoniae*) bacteria, respectively.

### 2.7. Minimum Lethal Concentration Assessments

BRIS/91/HEPU/1279 trophozoites were grown in dose–response plates as described above. Following 48 h incubation plates were sealed with Parafilm (Bemis Manufacturing, Sheboygan Falls, Wisconsin USA) and growth was assessed manually by light microscopy before being placed on ice for 30 min to dislodge adherent cells. The content of each well was aspirated and used to inoculate individual 8 mL culture tubes. After 4 days, viable cells and minimum lethal concentrations were examined by assessing parasite growth in tubes by light microscopy. Experiments were conducted three times in in technical duplicate. Representative images were taken on an Olympus BX-75 inverted microscope, Olympus, Tokyo, Japan.

### 2.8. Combination Studies

The interaction of SN00797640 with albendazole and metronidazole was examined. The activity of each combination was assessed against BRIS/91/HEPU/1279. A total of 14, 8-point dose–response assays were performed with each combination in triplicate on at least two occasions in 96-well microtiter plates (1.5x10^4^ BRIS/91/HEPU/1279 trophozoites per well). Plates were incubated in chambers gassed with 3% O_2_, 5% CO_2_ in N_2_ at 37 °C for 24 h before parasite growth was assessed by imaging [23]. Media and vehicle controls were included on each plate. Isobolograms were constructed by identifying combinations of compounds that resulted in the same effect, an IC_50_, and then plotting these values as normalized fractional-inhibitory concentration (FIC) values. Isoboles were fitted using the standard hyperbolic function defined by the parameter I as previously described [27,28], where Positive values of I indicate synergy, negative values indicate antagonism and addition occurs when I equals 0. The significance of the difference of I from zero was assessed with Student’s *t* test.

### 2.9. In Vivo Tolerability Assessments

The in vivo tolerability of SN00797640 was assessed in female outbred Swiss mice purchased from the Animal Research Centre (Perth, Australia) in accordance with ethical approval ESK/01/17/AEC (Griffith University approved 10.04.2017) using a dose escalation toxicity protocol. In brief, separate groups of three mice were administered single, increasing doses of compound via oral gavage to examine tolerability. All mice were assessed for adverse effects for a week following compound administration with any increase in dose being dependent on the safety of the prior, lower dose administration. After a week of observation, mice in each group were euthanized and samples collected for pathology assessment. The highest dose assessed was 50 mg/kg. To further assess the safety of SN00797640, this highest dose was also administered to a final group of three mice, daily for three days. All mice in this group were observed for one week after the final dose, after which they were euthanized, and samples collected for pathology assessment.

## 3. Results

### 3.1. Identification of Compounds with Anti-Giardia Activity

**Figure 1:**
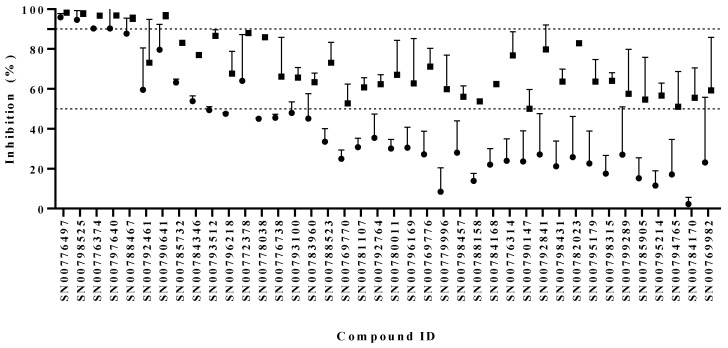
Anti-*Giardia* activity of hits identified in a sub-set screen of the Compounds Australia Scaffold Library. Compounds were tested for activity at 10 µM for 24 h (●) and 48 h (■). Data are presented as mean % inhibition of trophozoite growth + SD (n = 2). Only compounds which inhibited growth by >50% (above the lower dotted line) at one or both time-points are shown. Data for all other compounds can be found in Supplementary Material, Table S1.

A subset of 2451 compounds from the Compound’s Australia Library were screened for anti-Giardia activity at 10 µM. Data identified 66 compounds that were able to inhibit parasite growth by ≥50% at 24 or 48 h (Table S1). These compounds, together with an additional 17 compounds that showed inhibitory activity in the screen or were from scaffolds of interest were retested. Of those rescreened, 40 compounds were confirmed as hits (Figure 1; Table S1; hit rate 1.6%). Six hit compounds (SN00776497, SN00776374, SN00797640, SN00798525, SN00788467, and SN00790641) showed >90% inhibitory activity by 48 h (Figure 1). The reliability of data obtained from all assay plates in the primary screen was assessed by calculating Z’ factors [24]. While average Z’ factors derived from 24 h exposures suggested this time-point to be more reliable than the 48 h time-point, scores from both time-points were indicative of excellent screening assays (mean Z’ factor values were 0.82 ± 0.09 and 0.77 ± 0.13 for the 24 and 48 h time-points, respectively). A total of 46 compounds were taken forward to dose response assays. This included hits and hit analogues which showed some inhibitory activity in screen assays (for structure activity assessment).

Data from dose response experiments identified 23 compounds with 48 h IC_50_ values greater than 10 µM, 12 compounds with 48 h IC_50_ of 5 to 10 µM and eight compounds with IC_50_ values of 1 to 5 µM (Table S2). Three compounds, each from a different scaffold group, demonstrated potent IC_50_ values < 1 µM which was less than the 48 h IC_50_ of control drug, metronidazole (Table 1). The 48 h IC_50_ values for these compounds were 0.06 µM (SN00788467), 0.08 µM (SN00776497) and 0.18 µM (SN00797640). While the paired scaffold compounds for these hits were not hits in screening assays (Table 1; IC_50_ values > 10 µM), all demonstrated promising selectivity for parasites versus human cells, with Neonatal Foreskin Fibroblast (NFF) selectivity indices of >55 (Table 1). In addition, SN00797640 demonstrated little activity against any of the bacterial species examined (Table S2). SN00788467 and SN00776497 demonstrated inhibition of *Staphylococcus aureus*, 99% and 56% at 50 µM, respectively, but did not inhibit *Escherichia coli*, *Acinetobactor baumannii* or *Klebsiella pneumoniae* (Table S2).

### 3.2. Activity Assessments with Selected Scaffold Sets

Given the promising activity displayed by SN00788467, SN00776497 and SN00797640, all analogues in their respective scaffold sets CL3439, SC003542 and CL9406 were assessed for the anti-Giardia activity. No additional active compounds were found in CL3439 or SC003542 (IC_50_ >10 µM; data not shown). However, four additional active compounds (SN00797643, SN00797637, SN00797646; SN00797648) were identified in the CL9406 scaffold set (Table 2). While the most active of these four compounds SN00797643 (48 h IC_50_ 470 nM; Table 2), was less potent than the original hit, SN00797640 (IC_50_ 180 nM), it displayed sub-µM activity and did not impact the growth of NFF cells at the highest concentration tested (10 µM), suggesting 7-acylamidodibenzoxazepinones as a promising anti-Giardia compound series.

### 3.3. Follow-Up Activity Studies with CL9406 Scaffold Compound SN00797640

**Figure 2:**
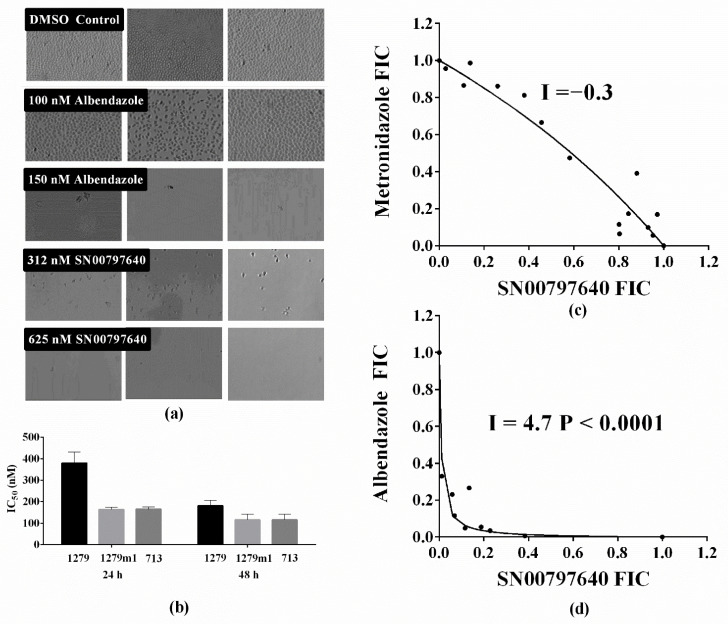
Anti-*Giardia* activity of SN00797640. To further investigate the potential of scaffold CL9406 compounds the activity of SN00797640 against *G. duodenalis* was further investigated. (**a**) Images of parasites exposed to SN00797640, albendazole or DMSO demonstrated parasiticidal activity and SN00797640 to have a sub-µM minimum lethal concentration; (**b**) The activity (IC_50_ + SD) of SN00797640 against the assemblage B, A, and metronidazole tolerant *G. duodenalis* cell lines, BRIS/91/HEPU/1279, BRIS/87/HEPU/713 and BRIS/91/HEPU/1279m1, respectively; (**c**) Isobologram demonstrating the additive anti-*Giardia* activity of SN00797640 when combined with metronidazole; (**d**) Isobologram demonstrating the synergistic anti-*Giardia* activity of SN00797640 when combined with albendazole.

To further assess the potential of CL9406 scaffold compounds as new anti-Giardia compound leads, the in vitro activity of SN00797640 was further investigated. This included re-purchasing the compound and examining activity against the assemblage A parasite line BRIS/87/HEPU/713, the metronidazole resistant line BRIS/91/HEPU/1279m1, estimating this compound’s minimum lethal concentration and examining the activity of this compound when combined with metronidazole or albendazole. Data from these studies demonstrated activity against assemblage A (24 h IC_50_ 164 ± 11 nM; 48 h IC_50_ 115 ± 30 nM), and metronidazole resistant parasites (24 h IC_50_ 160 ± 27 nM; 48 h IC_50_ 124 ± 3 nM; Figure 2). Importantly no significant differences were observed in the 24 h or 48 h IC_50_ values of all parasite lines tested (*P* > 0.05 for all comparisons). However, there was a trend for reduced sensitivity with assemblage B parasites (Figure 2). Despite this trend, SN00797640 demonstrated potent sub-µM parasiticidal activity against BRIS/91/HEPU/1279, with a minimum lethal concentration < 625 nM (Figure 2). The minimum lethal concentration of albendazole (150 nM; Figure 2) during these studies was in line with previously reported values [29]. Combination studies with metronidazole demonstrated additive activity (I = −0.3 which was not statistically different from 0; *P* = 0.1; Figure 2c). However, in vitro combination studies with albendazole demonstrated promising synergistic activity worthy of further investigation (I = 4.7, *P* < 0.001; Figure 2d).

To facilitate in vivo studies, including combination treatment assessments, preliminary tolerability studies were performed in mice. Data demonstrated SN00797640 to be well tolerated with no adverse reactions detected at doses of up to 50 mg/kg administered daily for three consecutive days. Histological analysis of liver and kidney samples taken from test animals also suggested no toxicity (data not shown).

## 4. Discussion

New anti-*Giardia* drugs and treatment strategies are required to combat increasing reports of parasite drug resistance and treatment failures. To widen the search for new and specific anti-*Giardia* agents, the current project sought to investigate the Compounds Australia Scaffolds Library for compounds with activity against this parasite. A sub-set screen of 2451 compounds (two per scaffold) identified 40 compounds (1.6% hit rate) with >50% inhibition at 10 µM. Further dose response assays with hits identified 11 compounds with IC_50_ values < 5 µM and three (SN00776497, SN00799467 and SN00797640; Table 1) with exciting sub-µM IC_50_ values. The most potent of these compounds, SN00788467 demonstrated a 48 h IC_50_ of 0.06 µM against *G. duodenalis* assemblage B parasites, >45-fold more potent than metronidazole and like the activity displayed by albendazole (Table 1). SN00788467 also displayed promising selectivity for *G. duodenalis* over NFF cells (Selectivity Index > 166; Table 1) and most bacteria (SI > 800 for *E. coli*, *A. baumannii* and *K. pneumoniae*, Table S2) suggesting the identification of a promising new antiparasitic drug lead. However, SN00788467 demonstrated activity against *S. aureus* (Table S2; 99% inhibition at 50 µM) and no additional analogues with anti-*Giardia* activity were detected within scaffold CL3439. While the activity of SN00788467 is not yet understood it contains a nitrofuran which may be reduced by redox active enzymes. In addition, the activity of this compound against *S. aureus* is consistent with previous work that described the activity of similar compounds against these microbes [30]. A sub-structure search for relevant literature identified two patents [31,32] and an additional publication suggesting that SN00788467 analogues may have multiple biological activities including activity against selected tyrosine phosphatases and adenosine A2A receptor antagonists [33]. Whether these activities account for the antiparasitic activity observed in the current study requires further investigation. Related 5-nitro-2-furancarboxylamides have been reported to have trypanocidal activity, without exhibiting cross resistance to nifurtimox [34].

The second most potent compound with a 48 h IC_50_ of 0.08 µM against *G. duodenalis* assemblage B parasites, >30-fold more potent activity than metronidazole, was SN00776497 (Table 1). SN00776497 also displayed promising selectivity for *G. duodenalis* over NFF cells (SI 101; Table 1) and bacteria (SI > 600, Table S2) suggesting the identification of a promising new antiparasitic drug lead. However, like the CL3439 series, no additional analogues with anti-*Giardia* activity were detected within the SC003542 scaffold series so further studies that permit pharmacophore identification are needed. The importance of the (N-(6-4-methylpiperazin-1-yl) acetamide moiety of SN00776497 in mediating activity will be particularly interesting given screened analogues did not contain this substructure. Importantly, an additional 369 compounds with this moiety that have not been assessed for anti-*Giardia* activity are available from Compounds Australia to facilitate this work. Studies to further understand the anti-*Giardia* potential of SN00776497 are warranted given that images of parasites treated with SN00776497 suggest parasiticidal activity and the repurchase and reassessment of a secondary batch of SN00776497 (data not shown) confirmed activity.

The final compound to demonstrate a sub-µM IC_50_ value in screening assays was SN00797640 (48 h IC_50_ 181 nM). While less active than SN00788467 and SN00776497, SN00797640 was >10-fold more active than metronidazole (Table 1). In contrast to SN00788467 and SN00776497, however, testing the CL9406 scaffold set identified an additional four active compounds (SN00797643 IC_50_ 470 nM, SN00797648 IC_50_ 1.4 µM, SN00797637 IC_50_ 5.0 µM and SN00797646 IC_50_ 8.6 µM), providing confidence in anti-*Giardia* activity and some structure activity relationship information. Of note, all active CL9406 compounds possessed an N-methyl group on the central ring rather than the bulkier N-isopropyl (compare SN00797640 with SN00797658; Table 2). Within the N-methyl compounds, a limited range of substitutions in the phenyl ring of the benzamide seem to be tolerated: Other than 4-methylphenyl, the only other aryl groups to show activity were 2-indolyl (SN00797648) and 4-ethylphenyl (SN00797643), with possible weak activity for the 3,5-dimethoxyphenyl (SN00797637) and 3,4-dimethoxyphenyl (SN00797646) compounds. Notably the unsubstituted phenyl (SN00797647) and 3-methylphenyl (SN00797639) compounds were inactive. Given the promising activity observed in the CL9406 scaffold set and the absence of any perceived liabilities, additional activity assays were performed with the most active candidate, SN00797640. Data from this work further demonstrated the potential of SN00797640 as a lead anti-*Giardia* drug candidate, demonstrating this compound to have activity against both human-infecting *G. duodenalis* assemblages (A and B) as well as metronidazole tolerant parasites (Figure 2). While additional drug resistant isolates including isolates from clinically refractory infections should be tested in future work, these data suggest that the metronidazole resistance mechanisms employed by BRIS/91/HEPU/1279m1 do not affect the potency of SN00797640. SN00797640, demonstrated similar activity (*P* > 0.05) against all *Giardia* isolates tested. The antiparasitic activity demonstrated by SN00797640 was also parasiticidal, with sub-µM concentrations effective in completely killing parasites after 48 h exposure (Figure 2). These data are encouraging as they suggest SN00797640 is toxic to parasites at low concentrations that have limited activity against other microbes (<5% inhibition of all bacteria species tested at 50 µM; Table S2). While further studies are required to ascertain the in vivo activity of this compound, this in vitro activity, together with data suggesting good tolerability in animals (no adverse reactions at doses up to 50 mg/kg daily for three days) bodes well for the development of a new specific anti-*Giardia* treatment that does not impact the host microbiota. The impact of anti-*Giardia* compounds on commensal microbes has been a neglected aspect of current interventions that is being increasingly recognized as having important consequences for long-term health (reviewed in [1]).

The additive and synergistic interactions of SN00797640 with metronidazole and albendazole, respectively, also suggest that the use of this compound or an optimized analogue in the in vivo setting may have advantages in combating drug resistance and treatment failures. While clinicians often address treatment failures by increasing drug doses or extending the length of treatment regimes, drug combination treatment strategies, particularly those that behave synergistically may well be the best way to combat these infections [35]. However, current combination treatment recommendations are limited by data, with very few studies investigating anti-*Giardia* compound interactions (review in [1]).

The synergistic interaction of SN00797640 with albendazole may also be instrumental in improving our understanding of this compound’s mode of action noting that dibenzoxazepinones bearing a nitrogen at C7 have not previously been reported to have antiparasitic activity. Mode of action studies using untargeted proteomic and transcriptomic approaches are currently underway and this compound’s interaction with albendazole will guide the selection of putative targets implicated by this work. Benzimidazoles, including albendazole are believed to target parasites by selectively preventing microtubule assembly [36,37] and inducing oxidative stress [38].

## 5. Conclusions

The current study identified multiple compounds with anti-*Giardia* activity worthy of further investigation, including three compounds with sub-µM IC_50_ values (SN00776497, SN00799467 and SN00797640). Each of these three compounds was more potent than metronidazole, displayed promising in vitro selectivity for *Giardia* parasites over human cells (SI > 55) and displayed limited activity against selected bacteria. Scaffold studies identified multiple compounds in the CL9406 scaffold series with anti-*Giardia* activity, with the most potent, SN00797640, demonstrating parasiticidal activity at concentrations as low as 625 nM. SN00797640 also behaved synergistically with albendazole, was active against multiple parasite lines (assemblage A, B, and the metronidazole tolerant line BRIS/91/HEPU/1279m1) and was well-tolerated in mice at doses up to 50 mg/kg suggesting it to be a promising new candidate for anti-*Giardia* drug development.

## Figures and Tables

**Table 1:** The activity of subset compounds within scaffold sets that contained at least one member with a sub-µM IC_50_ against Bris/91/HEPU/1279.

Compound (Scaffold)	Structure	IC_50_ (µM; Mean ± SD)		Selectivity
		*Giardia* (48 h)	NFF	
SN00776497 (SC003542)	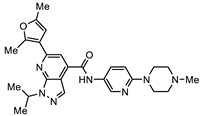	0.08 + 0.09	8.1 ^2^	101
SN00776477 (SC003542)	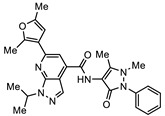	>10.0	Not tested	Not tested
SN00788467 (CL3439)	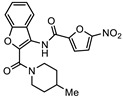	0.06 + 0.06	>10.0	>166
SN00785329 (CL3439)	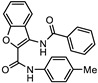	>10.0	Not tested	Not tested
SN00797640 (CL9406)	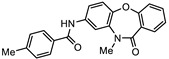	0.18 + 0.02	>10.0	>55
SN00797660 (CL9406)	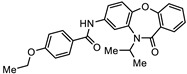	>10.0	Not tested	Not tested
Metronidazole ^1^	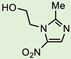	2.7 ± 0.7	>75	>27
Albendazole ^1^	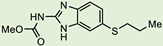	0.04 ± 0.02	1.1 ± 0.5	27

^1^ Metronidazole and albendazole (green shading) were control compounds; ^2^ Only one replicate reached 50% inhibition.

**Table 2:**
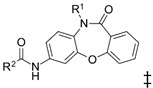
The in vitro activity of compounds in the CL9406 scaffold set.

Compound	R^1^	R^2^	IC_50_ (µM; Mean ± SD)		Selectivity
			*Giardia* (48 h)	NFF	
SN00797638	Me	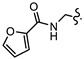	>10	Not determined	Not determined
SN00797644	Me	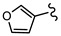	>10	Not determined	Not determined
SN00797647	Me	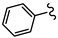	>10	Not determined	Not determined
SN00797642	Me	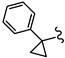	>10	Not determined	Not determined
SN00797645	Me	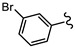	>10	Not determined	Not determined
SN00797639	Me	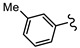	>10	Not determined	Not determined
SN00797640	Me	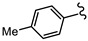	0.18 + 0.02	21.8 + 0.57	121
SN00797643	Me	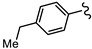	0.47 + 0.08	>10	>20
SN00797641	Me	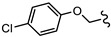	>10	Not determined	Not determined
SN00797646	Me	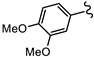	>8.6 *	Not determined	Not determined
SN00797635	Me	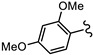	>10	Not determined	Not determined
SN00797637	Me	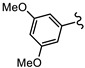	5.0 + 0.7	Not determined	Not determined
SN00797636	Me	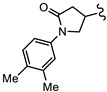	>10	Not determined	Not determined
SN00797648	Me	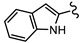	1.7 + 0.4	>20	>11
SN00797649	Me	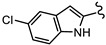	>10	Not determined	Not determined
SN00797659	Me_2_CH	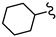	>10	Not determined	Not determined
SN00797661	Me_2_CH	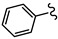	>10	Not determined	Not determined
SN00797658	Me_2_CH	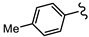	>10	Not determined	Not determined
SN00797656	Me_2_CH	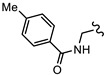	>10	Not determined	Not determined
SN00797660	Me_2_CH	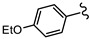	>10	Not determined	Not determined
SN00797650	Me_2_CH	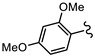	>10	Not determined	Not determined
SN00797651	Me_2_CH	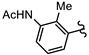	>10	Not determined	Not determined
SN00797652	Me_2_CH	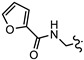	>10	Not determined	Not determined
SN00797655	Me_2_CH	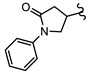	>10	Not determined	Not determined
SN00797654	Me_2_CH	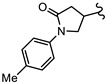	>10	Not determined	Not determined
SN00797653	Me_2_CH	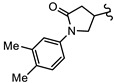	>10	Not determined	Not determined
SN00797663	Me_2_CH	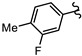	>10	Not determined	Not determined
SN00797657	Me_2_CH	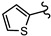	>10	Not determined	Not determined
SN00797662	Me_2_CH	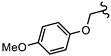	>10	Not determined	Not determined
SN00797664	Me_2_CH	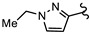	>10	Not determined	Not determined

‡ NOTE: The commercial supplier of the CL9406 scaffold incorrectly designated the set as a series of 8-acylamides. However, the set are a series of regioisomeric 7-acylamides (ChemDiv personal communication and [22]). * Only one replicate reached 50% inhibition.

## Data Availability

Data will be deposited into PubChem on publication acceptance.

## Supplementary Materials

The following supporting information can be downloaded at: https://www.mdpi.com/article/10.3390/biomedicines10123182/s1, Supplementary Table S1: Activity of Compounds Australia Scaffold Library Compounds against *G. duodenalis* in primary screens and Supplementary Table S2: Activity of selected Compounds Australia Scaffold Library compounds in cytotoxicity and *Giardia* dose response assays.
