# Supplementary material for: A Subset Screen of the Compounds Australia Scaffold Library Identifies 7-Acylaminodibenzoxazepinones as Potent and Selective Hits for Anti-Giardia Drug Discovery
Source: Biomedicines. 2022 Dec 8;10(12):3182. doi: 10.3390/biomedicines10123182 (PMC9775191; doi:10.3390/biomedicines10123182)
Supplement: Supplementary file 1 [file biomedicines-10-03182-s001.zip › biomedicines-2073275-supplementary.pdf]

Table S1. Activity of Compounds Australia Scaffold Library Compounds against *G. duodenalis* in primary screens.

A sub-set of the Compounds Australia Scaffold Library was assessed for activity against *G. duodenalis* BRIS/91/HEPU/1279. Each compound was assessed in singlicate, at a final concentration of 10µM with activity assessed at 24 and 48h via live-cell imaging and automated enumeration. Compounds with $\geq$50% inhibitory activity or from a scaffold containing compounds with activity were re-assessed in singlicate. Data from both assays and average growth inhibition relative to untreated vehicle control wells are provided.

Compound ID	Scaffold	Smiles	% Inhibition n=1		% Inhibition n=2		Average % Inhibition	
			24h	48h	24h	48h	24h	48h
SN00797640	CL9406	Cc1ccc(cc1)C(=O)Nc1ccc2Oc3ccccc3C(=O)N(C)c2c1	97.5	96.3	83.1	97.2	90.3	96.8
SN00776497	SC003542	CN1CCN(CC1)c1ccc(cn1)NC(=O)c1cc(nc2c1cnn2C(C)C)c1cc(C)oc1C	94.6	98.0	97.2	98.5	95.9	98.2
SN00788467	CL3439	CC1CCN(CC1)C(=O)c1oc2ccccc2c1NC(=O)c1ccc(o1)N(=O)=O	93.2	93.9	82.3	96.4	87.7	95.2
SN00798525	CL9569	Cc1cc2[nH]c(C(=O)Nc3ccccc3C)c(CN3CCOCC3)c2s1	91.4	96.5	97.9	98.8	94.7	97.6
SN00776374	SC003155	COc1ccc(C)cc1N1C(=C(c2nc3ccccc3s2)C(c2ccccc2)C1=O)N	90.5	95.8	90.0	97.6	90.3	96.7
SN00790641	CL7819	Fc1ccc(cc1)N(C)C(=O)Cn1nc2c3cc(nn3ccn2c1=O)c1cc(C)ccc1C	88.6	95.0	70.6	97.7	79.6	96.3
SN00772378	SC012476	O=N(=O)c1ccc(N2CCC(CC2)c2ncc[nH]2)c2ccncc12	80.4	88.7	47.7	87.4	64.0	88.0
SN00785732	CL4038	O=C(c1ccc2OCOc2c1)c1c(N)c(c2scc(n2)c2ccccc2)c2ccccn12	64.4	82.4	62.0	83.8	63.2	83.1
SN00783960	CL3097	CC(=O)Nc1ccc(N(=O)=O)c2nonc12	53.9	66.5	36.2	60.2	45.1	63.3
SN00784346	CL5887	Cc1ccc(cc1)n1c(=O)n(CC(=O)Nc2ccccc2C)c2cnn(C)c2c1=O	52.0	77.6	55.8	76.3	53.9	76.9
SN00793100	CL2800	COc1ccccc1N1CCN(CC1)C(=O)c1cc2c(C)nn(c3ccc(C)cc3)c2s1	51.9	62.1	44.0	69.2	48.0	65.6
SN00793512	CL6605B	COc1ccc(cc1)Nc1cc(C)nc(n1)N1CCN(CC1)C(=O)Nc1cccc(OC)c1	50.6	88.7	48.1	84.5	49.3	86.6
SN00796218	CL4659	Clc1ccc(cc1)NC(=O)Cc1csc2nc(cn12)c1ccccc1	47.2	59.9	48.0	75.5	47.6	67.7
SN00776738	SC005830	O=S1(=O)CCC(C1)n1ncc(c1)c1cnn(c1)c1ccccc1	46.8	52.3	44.3	80.1	45.5	66.2
SN00769982	SC001350	COc1cc(OC)c(C2=CCN(CC2)C(=O)c2ccc(C)s2)c(OC)c1	46.2	78.1	0.0	40.5	23.1	59.3
SN00778038	SC007824	CN(C)CCn1cc(cc1c1nc2ccccc2c(=O)[nH]1)C(=O)c1cc(Cl)ccc1O	45.3	85.2	44.7	86.5	45.0	85.8
SN00792461	CL7138	O=C(NC1CCCCC1)c1nc(Nc2ccccc2)c2ccccc2n1	44.7	57.7	74.4	88.5	59.5	73.1
SN00799289	CL9921	CC(C)c1ccc(cc1)Nc1ncccc1S(=O)(=O)N1CCCC1	43.9	73.3	10.1	41.7	27.0	57.5
SN00792764	CL7220	Cc1ccc(cc1)NC(=O)CN1C(=O)C2CCCCN2c2ccc(cc12)C(=O)N1CCCC1	43.9	59.0	27.1	65.8	35.5	62.4
SN00792841	CL7285A	CCOc1cc(CCNC(=O)c2ccc3c(c2)nc2COCCn32)ccc1OCC	41.7	88.5	12.7	71.1	27.2	79.8
SN00788144	CL5291	Cc1ccc(cc1)NC(=O)c1cc2c(Cl)ccc3OCCn1c23	41.0	65.8	20.1	32.9	30.5	49.3
SN00782023	SC015404	COc1c(C)cnc(Cn2cnc(c2)N(=O)=O)c1C	40.2	83.4	11.3	82.5	25.8	82.9
SN00798457	CL9915	Cc1ccc(cc1)C(=O)Nc1cc(sc1C)c1scc(C)n1	39.3	60.0	16.7	52.2	28.0	56.1
SN00788523	CL5077	CCc1nnc2n1c(nc1ccccc21)N1CCN(CC1)c1cccc(Cl)c1	38.2	65.8	28.9	80.4	33.6	73.1
SN00769776	SC001254	O=C(Nc1sc(c2ccco2)c(n1)c1ccco1)c1ccccc1	35.4	77.6	19.0	64.8	27.2	71.2
SN00790147	CL8253	COc1cc(cc2sc(NC(=O)c3ccccc3)nc12)NS(=O)(=O)c1ccc(C)cc1	34.5	56.9	12.8	43.2	23.6	50.1
SN00795993	CL8934	O=S(=O)(Nc1cccc(c1)c1nnc(Cc2ccc3OCOc3c2)o1)Cc1ccccc1	34.4	80.2	0.0	13.6	17.2	46.9
SN00795179	CL8496	Cc1csc(n1)c1cc(c(C)n1C)S(=O)(=O)N1C(C)Cc2ccccc12	34.1	71.5	11.1	55.8	22.6	63.7
SN00783575	CL2800	O=C(N1CCCCC1)c1cc2c(C)nn(c3ccccc3)c2s1	34.0	63.8	8.3	15.4	21.1	39.6
SN00781107	SC013130	Fc1ccc(Sc2nnc(s2)N2CCOCC2)c(c1)N(=O)=O	34.0	64.2	27.7	57.4	30.8	60.8
SN00780011	SC010778	O=N(=O)c1cnc(C)n1Cc1csc(Cc2ccccc2)n1	33.3	54.9	27.0	79.2	30.1	67.1
SN00776314	SC003136	COc1cc(ccc1OC)c1csc(n1)C1=C(N)N(CC1=O)CC(C)(C)CN(C)C	31.7	85.1	16.2	68.5	24.0	76.8
SN00798431	CL8499	Cc1cccc(CNS(=O)(=O)c2cc(sc2C)c2csc(C)n2)c1	30.2	59.2	12.3	68.1	21.2	63.6
SN00795039	CL7935	Cc1ccnc(n1)N1CCCC(C1)C(=O)N1CCC(=CC1)c1ccccc1	29.9	57.6	6.1	34.6	18.0	46.1

Compound ID	Scaffold	Smiles	% Inhibition n=1		% Inhibition n=2		Average % Inhibition	
			24h	48h	24h	48h	24h	48h
SN00794765	CL8472	COc1ccc(cc1)NC(=O)c1ccc(cc1)c1ncnc(NC2CCCCC2)c1	29.5	63.5	4.7	38.6	17.1	51.0
SN00784168	CL3010	COc1cccc(c1)Nc1c2CCCc2nc2ccccc12	27.7	63.3	16.4	61.4	22.0	62.4
SN00786825	CL9262	NC(=O)C1=C(Nc2ccccc2)c2ccccc2S1(=O)=O	25.7	65.7	0.0	31.3	12.8	48.5
SN00791795	CL6760	COc1ccc(cc1)C1=Nc2ccccc2N(CC(=O)Nc2cccc(F)c2)C(=O)C1	25.2	54.2	0.0	26.4	12.6	40.3
SN00798315	CL9560	Fc1cccc(c1)c1onc(n1)c1cccc(c1)c1ccnn1C	24.1	66.9	11.1	61.2	17.6	64.0
SN00785905	CL5635	Cc1ccc2NC(=NC(=Nc2c1)c1ccc(C)o1)c1cccs1	22.4	69.6	8.0	39.6	15.2	54.6
SN00790827	CL5098	Fc1ccc(cc1)Nc1nc(cc2occc12)C(=O)N1CCN(CC1)c1ccccn1	21.9	53.8	6.5	8.1	14.2	31.0
SN00769770	SC001254	CC(=O)c1c(C)[nH]c(C(=O)Nc2sc(c3ccco3)c(n2)c2ccco2)c1C	21.9	59.6	28.1	45.9	25.0	52.7
SN00796317	CL9131	COc1ccc(Cl)cc1NC(=O)Nc1ccc2CN(Cc3ccccc3)Cc2c1	17.7	57.6	0.0	17.4	8.8	37.5
SN00791806	CL6760	COc1ccc(cc1)C1=Nc2ccccc2N(Cc2cccc(F)c2)C(=O)C1	17.4	50.8	0.0	9.6	8.7	30.2
SN00798443	CL8499	CCc1scc(n1)c1sc(C)c(c1)S(=O)(=O)NCc1ccc(F)c(Cl)c1	16.9	50.2	13.6	37.5	15.2	43.8
SN00795214	CL8595	COc1ccc2oc(c3noc(n3)C(=O)NCCN3CCCCCC3)c(C)c2c1	16.8	52.1	6.3	61.1	11.6	56.6
SN00783501	CM2887	Brc1ccc(cc1)C(=O)NCC1(CCOCC1)c1ccccc1	16.8	61.1	0.9	14.4	8.8	37.7
SN00786482	CL8245B	Cc1nc(cc(n1)N(C)C)N1CCN(CC1)C(=O)Nc1cccc(c1)C(F)(F)F	15.1	55.4	6.1	2.9	10.6	29.2
SN00786172	CL7099	CCCNC(=O)c1sc(nc1C)n1nc(C)c(Cl)c1C	14.5	52.5	0.0	1.5	7.3	27.0
SN00780175	SC011001	Clc1ccc(cc1)C(=O)C1=C(O)C(=O)N(c2noc(C)c2)C1c1ccc(cc1)N(=O)=O	12.8	59.1	0.0	0.0	6.4	29.6
SN00788158	CL5291	CCC1Oc2cccc3cc(C(=O)NCCN(C)C4CCCCC4)n(C1)c23	11.2	54.5	16.5	52.9	13.9	53.7
SN00793485	CL6605A	COc1ccc(cc1)Nc1cc(C)nc(n1)N1CCN(CC1)C(=O)c1ccccc1	11.1	58.6	0.0	0.0	5.5	29.3
SN00798298	CL9560	Cc1ccc(cc1)c1onc(n1)c1cccc(c1)c1ccn[nH]1	9.4	55.6	3.2	27.4	6.3	41.5
SN00784170	CL3010	Fc1cccc(c1)Nc1c2CCCc2nc2ccccc12	4.6	66.2	0.0	44.9	2.3	55.5
SN00796169	CL5888	Fc1cccc(c1)NC(=O)Cn1c2cn(C)nc2c(=O)n(Cc2ccccc2)c1=O	37.8	46.9	23.4	78.6	30.6	62.8
SN00795169	CL8496	CCCNS(=O)(=O)c1cc(c2scc(n2)c2cccc(Cl)c2)n(C)c1C	31.8	47.2	12.0	18.5	21.9	32.8
SN00803183	CL9110	Clc1cccc(CNc2ncnc3c(sc(C)c23)C(=O)N2CCCC2)c1	29.7	47.9	1.5	7.3	15.6	27.6
SN00799296	CL9921	CCOC(=O)c1cccc(c1)Nc1ncccc1S(=O)(=O)N	29.1	70.8	0.0	0.0	14.5	35.4
SN00789104	CL7757	CCc1onc(n1)c1sc(C)c(c1)S(=O)(=O)N(C)c1cccc(C)c1	28.4	48.8	0.0	12.8	14.2	30.8
SN00783428	CL0341	Brc1ccc(cc1)[C@@H]1Nc2c(cnn2[C@@H](C1)C(F)(F)F)C(=O)NCc1ccco1	27.8	35.3	2.5	1.8	15.1	18.5
SN00776417	SC003253	FC(F)Oc1ccc(cc1)c1csc(n1)Cc1nnc2CCCCCn12	22.0	34.2	2.6	5.0	12.3	19.6
SN00783518	CL1132	Cc1ccc(cc1)[C@H]1Nc2nnnn2[C@H](C1)c1ccccc1	21.3	40.1	0.0	0.0	10.7	20.0
SN00777378	SC007224	CCN(CC)S(=O)(=O)c1ccc(Nc2ccn(CCc3ccccn3)n2)c(c1)N(=O)=O	21.3	39.2	0.0	11.3	10.7	25.2
SN00798157	CL9487	COc1cc(ccc1OC)Nc1snc(n1)c1nnn(c1C)c1ccc(F)cc1F	18.3	52.7	4.5	0.0	11.4	26.3
SN00777397	SC007239	COc1cccc(c1)N1CCN(CC1)C(=O)c1ccc2noc(c3ccccc3)c2c1	17.2	56.5	2.5	0.0	9.9	28.2
SN00796834	CL9207	Cn1nc(c2nnc(o2)c2ccccc2Cl)c2CN(CCc12)C(=O)c1ccc(F)c(F)c1	12.9	25.5	0.0	0.0	6.4	12.8
SN00776775	SC005855	O=C(Nc1[nH]nc(c1)c1ccncc1)c1cccc(N(=O)=O)c1C	12.8	37.2	0.0	5.6	6.4	21.4
SN00778954	SC009242	CCOC(=O)N1CCN(CC1)C(=O)c1nn(c2ccccc2)c2CCCc12	12.5	38.8	0.0	33.3	6.2	36.1
SN00782002	SC015304	O=N(=O)c1ccccc1Sc1nnnn1C1CCOCC1	12.3	15.2	0.0	2.0	6.2	8.6
SN00786504	CL8245B	COc1cc(OC)cc(c1)NC(=O)N1CCN(CC1)c1nc(C)nc(c1)N(C)C	9.8	55.1	0.0	0.0	4.9	27.5
SN00793136	CL4469C	CCCNC(=O)N1CCN(CC1)c1nc2ccccc2n2cccc12	7.8	44.8	0.0	0.0	3.9	22.4
SN00786874	CL9434	Cc1cccc(c1)NC(=O)N1CCN(CC1)c1cc(C)nc(n1)N1CCCC1	7.1	49.3	0.0	30.5	3.5	39.9
SN00802023	CM1159	O=C(NC1CCCCCC1)c1cc2cn(cc2s1)C1CC1	3.6	43.4	0.0	50.4	1.8	46.9
SN00779876	SC010596	CCN(CC)C(=O)C1CSCN1C(=O)c1cc(C)nc2c1nc1CCCCCn21	1.9	62.1	0.0	0.0	1.0	31.1
SN00777183	SC006761	CCOC(=O)c1sc2ncnc(Nc3c[nH]nc3)c2c1C	0.1	42.7	0.0	18.9	0.0	30.8
SN00778065	SC007836	O=C(CCSCc1[nH]c(=O)c2c(n1)sc1CCCc21)N1CCCCCC1c1cccn1C	0.0	67.7	0.4	9.8	0.2	38.7
SN00772332	SC012288	OC(COCc1ccco1)CN1CCN(CC1)Cc1nnc(C)n1C	0.0	57.1	0.0	0.0	0.0	28.5
SN00778086	SC007836	Cc1ccc(cc1)c1nnn(n1)CC(=O)N1CCCCCC1c1cccn1C	0.0	56.5	4.1	0.0	2.1	28.3
SN00772523	SC013041	Cc1ccccc1C1CCCN1Cc1[nH]c(=O)c2cnn(C)c2n1	0.0	56.4	0.0	0.0	0.0	28.2
SN00776945	SC006559	CC(=O)Nc1cc(ccc1F)NC(=O)c1cc(nc2n(ncc12)Cc1cccnc1)c1ccccc1	0.0	50.1	0.0	0.0	0.0	25.1

Compound ID	Scaffold	Smiles	% Inhibition n=1		% Inhibition n=2		Average % Inhibition	
			24h	48h	24h	48h	24h	48h
SN00779996	SC010778	O=C(Cc1scc(n1)Cn1cnc(c1)N(=O)=O)Nc1cc(C)ccc1C	0.0	47.9	16.9	71.9	8.5	59.9
SN00776094	SC003062	Fc1ccc(cc1)c1ccc(o1)C1NC(=Nc2nc3ccccc3n12)N	0.0	31.0	0.0	5.8	0.0	18.4
SN00778869	SC008972	O=C(CCn1nc(C)c2c(C)onc2c1=O)Nc1cccc(F)c1	0.0	21.9	0.0	59.9	0.0	40.9
SN00793856	CL8306A	O=N(=O)c1ccc(cc1)S(=O)(=O)N1CCCCCC1c1cccs1	39.7	39.9	not tested	not tested	39.7	39.9
SN00786890	CL9402A	C=CCn1c(C)c(CC(=O)Nc2cc(F)ccc2C)c(=O)n2nc(nc12)c1ccccc1	38.7	40.6	not tested	not tested	38.7	40.6
SN00801319	CM2426	O=C(c1ccc2OCOc2c1)N(C)C[C@]12CCC[C@H]2N(C)C(=O)CC1	33.4	28.7	not tested	not tested	33.4	28.7
SN00796649	CL9305	CCN1CCN(CC1)c1ccc(cn1)c1onc(n1)c1cccc(F)c1	31.4	48.2	not tested	not tested	31.4	48.2
SN00779286	SC009701	O=N(=O)c1ncn(Cc2onc(n2)c2ccc(cc2)C(F)(F)F)n1	30.9	16.5	not tested	not tested	30.9	16.5
SN00793643	CL2286	Cc1nc2sc(C(=O)Nc3ccccc3Cl)c(C)c2c(n1)N1CCCC1	30.1	49.2	not tested	not tested	30.1	49.2
SN00793247	CL4110	COc1cccc(c1)Cn1c(CNC(=O)CCc2c(C)noc2C)nc2cccnc12	30.0	17.5	not tested	not tested	30.0	17.5
SN00803001	CL9791	CN(C)CCn1cnc2sc(C)c(C(=O)Nc3ccc(nc3)S(=O)(=O)N)c2c1=O	29.7	26.5	not tested	not tested	29.7	26.5
SN00803162	CL9065	Fc1ccc(cc1)n1nc(C)c2sc(nc12)N1CCCN(CC1)C(=O)Cc1cccs1	29.0	27.0	not tested	not tested	29.0	27.0
SN00794158	CL1240	COc1ccc(cc1)n1nnnc1C1(NC)CCCCC1	28.1	11.8	not tested	not tested	28.1	11.8
SN00798065	CL7707A	Clc1ccc(F)c(c1)C(=O)N1CCN(CC1)c1ncnc(c1)c1cccc(C)c1	27.8	47.1	not tested	not tested	27.8	47.1
SN00801956	CM1028	NC(=O)c1nc2csc(C)c2c(n1)N1CCCC1	27.3	22.6	not tested	not tested	27.3	22.6
SN00791542	CL6662A	COc1ccccc1NC(=O)N1Cc2c3CCN(C)Cc3sc2n2cccc2C1C	27.1	24.5	not tested	not tested	27.1	24.5
SN00796277	CL9005	Cc1ccc(cc1)NC(=O)Nc1ccc2c(CCN2S(=O)(=O)c2ccccc2)c1	26.8	10.0	not tested	not tested	26.8	10.0
SN00794182	CL9259A	Fc1ccc(cc1)C(=O)N1CCCN(CC1)Cc1noc(n1)c1ccccc1	26.7	33.4	not tested	not tested	26.7	33.4
SN00780622	SC011787	C=CCc1ccc(OCC(O)CN2C(=O)NC(C)(c3ccccn3)C2=O)c(OC)c1	26.5	48.2	not tested	not tested	26.5	48.2
SN00801852	CM0986	Clc1ccc(s1)C(=O)Nc1ccc(nc1)N1CCN(C2CCCCC2)C1=O	26.4	26.3	not tested	not tested	26.4	26.3
SN00791262	CL6301	CCC1N(Cc2ccccc2n2cccc12)C(=O)NCCc1ccccc1	26.1	18.8	not tested	not tested	26.1	18.8
SN00794458	CL2258	Brc1ccc(cc1)NC(=O)c1ccc(F)c(c1)S(=O)(=O)NCCc1ccccc1	25.9	3.9	not tested	not tested	25.9	3.9
SN00785985	CL6446	Cc1ccc(cc1)CNC(=O)c1ccc2c(CCN2S(=O)(=O)c2ccc(C)cc2)c1	25.8	38.5	not tested	not tested	25.8	38.5
SN00785210	CL3462	CC1CC(C)CN(C1)CC(=O)c1ccc2c(CCN2C(=O)C)c1	25.4	19.3	not tested	not tested	25.4	19.3
SN00776128	SC003068	Cc1nc2sccn2c1c1scc(n1)c1cccc2ccccc12	24.8	37.4	not tested	not tested	24.8	37.4
SN00792200	CL4869	Clc1cccc(c1)C1=NC2(CCN(CC2)C(=O)c2ccc(cc2)C(C)(C)C)NC1=O	24.6	32.1	not tested	not tested	24.6	32.1
SN00799926	CM0279	CSc1ccc(cc1)CNC(=O)c1cn2cc(C)ccc2n1	23.8	26.2	not tested	not tested	23.8	26.2
SN00796327	CL9131	Cc1ccc(NC(=O)Nc2ccc3CN(Cc4ccccc4)Cc3c2)c(C)c1	23.6	41.2	not tested	not tested	23.6	41.2
SN00802425	CM3115	CCOc1ccccc1CNC(=O)c1nc(oc1C)C(C)C	23.3	24.0	not tested	not tested	23.3	24.0
SN00799827	CL7444	CCOc1ccc(cc1)NC(=O)C1(CCCN(CC)CC1)c1ccccc1	23.3	23.8	not tested	not tested	23.3	23.8
SN00782656	SC015211	CCc1nnc2CN(CCn12)Cc1csc(n1)c1cscc1	23.3	24.2	not tested	not tested	23.3	24.2
SN00785914	CL5635	Fc1ccc(cc1)C1=NC(=Nc2cc(Cl)ccc2N1)c1cccs1	23.0	0.0	not tested	not tested	23.0	0.0
SN00787795	CL4252	CCCn1cc(c(=O)c2cc(F)c(cc12)N1CCC(C)CC1)S(=O)(=O)c1ccc(OC)cc1	22.7	0.0	not tested	not tested	22.7	0.0
SN00798581	CL9575A	COCCn1cc2CCN(Cc3cccc1c23)C(=O)Nc1ccccc1F	22.7	22.0	not tested	not tested	22.7	22.0
SN00773266	SC015438	O=C(C1CC1)N1CCCCCC1C1CCN(CC1)c1nccc(n1)C(F)(F)F	22.7	15.0	not tested	not tested	22.7	15.0
SN00794684	CL2385	CCOc1ccc(cc1)c1oc(C)c(CSCC(=O)N2CCN(CC2)c2ccc(Cl)cc2)n1	22.4	0.0	not tested	not tested	22.4	0.0
SN00784957	CM2505	CN(C)c1ncc(C(=O)O)c(C)n1	22.3	3.0	not tested	not tested	22.3	3.0
SN00795886	CL8682	Fc1cccc(CNC(=O)C2(C)CCCN(C2)C(=O)Cc2cccs2)c1	22.1	29.2	not tested	not tested	22.1	29.2
SN00788939	CL7566	Fc1cccc(c1)Cn1c(nc2ccccc12)c1ccc(C)o1	22.1	16.3	not tested	not tested	22.1	16.3
SN00790473	CL8372A	Fc1cccc(c1)NC(=O)c1nnc(s1)C1CCCN1C(=O)Nc1cccc(c1)C(F)(F)F	22.0	8.5	not tested	not tested	22.0	8.5
SN00775722	SC002397	Cc1cnc(CN2CCOC(CNc3cccnn3)C2)o1	22.0	13.5	not tested	not tested	22.0	13.5
SN00784824	CL2508	O=C(Nc1ccc(Br)c(C)c1)C1CCN(CC1)S(=O)(=O)c1c(C)n[nH]c1C	21.9	11.8	not tested	not tested	21.9	11.8
SN00794224	CL9262	CCCNC1=C(C(=O)Nc2ccc(F)cc2)S(=O)(=O)c2ccccc12	21.9	46.5	not tested	not tested	21.9	46.5
SN00790865	CL8415	Cc1ccc(cc1)CNC(=O)C1CCN(CC1)c1ncccc1n1cccc1	21.9	0.0	not tested	not tested	21.9	0.0
SN00785776	CL5363	Cc1ccc(cc1N1CC(=O)C(=C1N)c1nc2ccccc2[nH]1)N(=O)=O	21.6	21.1	not tested	not tested	21.6	21.1
SN00770261	SC001787	Cc1ccccc1C(=O)N1CCN(CC1)C(=O)c1[nH]nc2ccccc12	21.5	25.4	not tested	not tested	21.5	25.4

Compound ID	Scaffold	Smiles	% Inhibition n=1		% Inhibition n=2		Average % Inhibition	
			24h	48h	24h	48h	24h	48h
SN00799956	CM0284	CC(C)Cn1ccc2sc(C)c(C(=O)NCCN3CCCCC3)c2c1=O	21.5	23.1	not tested	not tested	21.5	23.1
SN00792985	CL7319	O=C(NCCCN1CCCC1)Cn1c(=O)c(=O)n(Cc2ccccc2)c2ccccc12	21.5	20.3	not tested	not tested	21.5	20.3
SN00776436	SC003253	CCN(c1ccc(cc1)c1csc(n1)Cc1nnc2CCCCCn12)S(=O)(=O)C	21.3	17.3	not tested	not tested	21.3	17.3
SN00793887	CL8308A	CCc1onc(C)c1C1CCCN1S(=O)(=O)c1ccc(cc1)CC(C)C	21.2	26.3	not tested	not tested	21.2	26.3
SN00793320	CL8425	Cc1onc(n1)c1ccc(cc1)C(=O)N1CCCC1C(=O)Nc1ccc(C)c(Cl)c1	21.1	16.2	not tested	not tested	21.1	16.2
SN00779378	SC009943	O=C(Nc1cccnc1)C1=NN(Cc2ccccc2)C(=O)CC1	21.1	15.0	not tested	not tested	21.1	15.0
SN00786766	CL7519	COc1ccc(cc1)NC(=O)c1nn(C)c(c1)C(F)(F)F	21.0	0.0	not tested	not tested	21.0	0.0
SN00783239	SC014207	CC(=O)NCc1ccc(cc1)C(=O)N1CCCCC(C1)N1CCCC1	21.0	4.5	not tested	not tested	21.0	4.5
SN00792995	CL6673	COc1ccc(OC)c(c1)NC(=O)N1CCC2(CC1)Nc1ccccc1n1cccc21	20.9	15.0	not tested	not tested	20.9	15.0
SN00782455	SC008797	CCn1ncc(CN2CCCN(CC2)C(=O)c2snnc2CC)c1	20.8	0.0	not tested	not tested	20.8	0.0
SN00777844	SC007674	O=C(COc1cccc(C)c1C)N1CCN(CC1)Cc1nnsc1Cl	20.5	35.2	not tested	not tested	20.5	35.2
SN00786098	CL7090	CCS(=O)(=O)c1ncc(N(Cc2ccco2)Cc2ccccc2F)c(n1)C(=O)Nc1ccccc1	20.5	17.4	not tested	not tested	20.5	17.4
SN00781212	SC013651	Fc1ccc(cc1)Oc1ccc(cn1)/C(=N\OCc1ncc(o1)c1cccs1)/N	20.4	41.6	not tested	not tested	20.4	41.6
SN00797669	CL6113	COc1cc(CCNS(=O)(=O)c2cccc(c2)c2onc(n2)c2ccc(C)cc2)ccc1OC	20.4	6.7	not tested	not tested	20.4	6.7
SN00775453	SC001667	COc1ccc(cc1)CN1CCCN(CC1)c1ncnc2ccccc12	20.1	0.0	not tested	not tested	20.1	0.0
SN00788624	CL7499	COc1cccc(c1)C(=O)n1nc(nc1NCc1ccc(C)cc1)c1cccnc1	20.0	24.5	not tested	not tested	20.0	24.5
SN00793864	CL8306A	O=N(=O)c1cccc(c1)S(=O)(=O)N1CCCCCC1c1cccs1	20.0	25.8	not tested	not tested	20.0	25.8
SN00797617	CL9960	COc1ccc(cc1)CNC(=O)c1sc(Nc2ccc(F)cn2)nc1C	19.9	12.4	not tested	not tested	19.9	12.4
SN00783379	CL1623	O=C(Nc1cc(Oc2cccnc2)cc(c1)N(=O)=O)Cn1nc(cc1C)C(F)(F)F	19.9	22.1	not tested	not tested	19.9	22.1
SN00794395	CL1545	Fc1ccc(cc1)n1c(=O)n(CC(=O)Nc2cccc(C)c2)c2sc(C(=O)N(C)C)c(C)c2c1=O	19.7	21.9	not tested	not tested	19.7	21.9
SN00802291	CM1835	CCCCN1CCN(CC1)C(=O)c1nn2c(c1)c(=O)n(CC(C)C)c1ccccc21	19.6	12.0	not tested	not tested	19.6	12.0
SN00794830	CL8582	O=C(N1CCN(CC1)c1ccccc1)c1csc(n1)c1ccncc1	19.5	15.8	not tested	not tested	19.5	15.8
SN00794033	CL8310	Brc1cccc(c1)C(=O)NCc1ccc(cc1)c1nn2c(C)nnc2s1	19.5	41.7	not tested	not tested	19.5	41.7
SN00790058	CL8242A	O=C(NCCc1c(C)noc1C)C1CCN(CC1)c1nc2cccnc2n(C)c1=O	19.3	15.0	not tested	not tested	19.3	15.0
SN00781241	SC013809	Nc1n[nH]c(c1)C1CCNCC1	19.3	7.2	not tested	not tested	19.3	7.2
SN00807837	SC012433	Cc1ccc(c(=O)[nH]1)C(=O)N1CCCN(CC1)S(=O)(=O)C	19.3	19.8	not tested	not tested	19.3	19.8
SN00788230	CL3220	CCN(Cc1ccccc1)C(=O)c1[nH]nc(c1)c1ccccc1	19.1	36.1	not tested	not tested	19.1	36.1
SN00787412	CL4156	CCn1cc2CCc3oc(C(=O)Nc4ccc(F)cc4)c(C)c3c2n1	19.1	6.9	not tested	not tested	19.1	6.9
SN00770035	SC001404	O=C(/C=C/c1cccc(c1)N(=O)=O)Nc1ccc2c(c1)nc1CCCCCn21	18.9	0.0	not tested	not tested	18.9	0.0
SN00794883	CL8126	Clc1ccc(cc1)CS(=O)(=O)c1nc2ccccc2nc1N1CCC2(OCCO2)CC1	18.9	15.2	not tested	not tested	18.9	15.2
SN00789011	CL2349A	CC(=O)c1ccc(cc1)Nc1ncnc2c1c(C)c(C)n2c1ccccc1	18.9	48.7	not tested	not tested	18.9	48.7
SN00785555	CL7522	CSc1ccc(cc1)C1NCc2ccccc2n2cccc12	18.8	3.9	not tested	not tested	18.8	3.9
SN00784677	CL2503	Fc1cccc(c1)C(=O)N1C2CC(C)(Oc3ccccc23)N(c2ccccc2)C1=O	18.7	0.0	not tested	not tested	18.7	0.0
SN00800883	CM1851	Cc1ccc(cc1)CNC(=O)N1CCN(CC1)c1ncn2C(C)CN=c2c1	18.7	11.9	not tested	not tested	18.7	11.9
SN00798647	CL9614	COc1ccccc1c1onc(n1)C1CCCN(C1)C(=O)c1ccc(cc1)C(C)C	18.7	0.0	not tested	not tested	18.7	0.0
SN00797660	CL9406	CCOc1ccc(cc1)C(=O)Nc1ccc2Oc3ccccc3C(=O)N(C(C)C)c2c1	18.7	33.8	not tested	not tested	18.7	33.8
SN00777547	SC007482	Cc1nnc(s1)NC(=O)c1ccc2c(c1)nc1CCCCCn1c2=O	18.6	37.7	not tested	not tested	18.6	37.7
SN00789526	CL3979	COc1ccc(cc1)Cn1c(=O)c2nccnc2n(Cc2cccc(C)c2)c1=O	18.6	0.0	not tested	not tested	18.6	0.0
SN00785585	CL3592	NCCCN(C)C(=O)c1ccc2c(c1)nc1CCCCn1c2=O	18.6	25.4	not tested	not tested	18.6	25.4
SN00792977	CL7319	CSc1ccc(cc1)CNC(=O)Cn1c(=O)c(=O)n(CC)c2ccccc12	18.5	16.0	not tested	not tested	18.5	16.0
SN00782244	SC016317	COc1ccccc1/C=C/CNCC1CCOC1c1cnn(CC)c1	18.5	28.6	not tested	not tested	18.5	28.6
SN00802402	CM2665	Cc1ccc(cc1)CNC(=O)N1CCCN(CC1)C1CCN(CC2CC2)C1=O	18.4	5.4	not tested	not tested	18.4	5.4
SN00802800	CL1665	CCN(CC)C(=O)c1cc(nc2cc(C)c(C)cc12)c1cccs1	18.4	16.9	not tested	not tested	18.4	16.9
SN00796606	CL9302	COc1ccc2oc(c3noc(n3)C(NC(=O)c3ccco3)C(C)C)c(C)c2c1	18.3	9.9	not tested	not tested	18.3	9.9
SN00797488	CL9352B	Cc1cccc(c1)c1noc(n1)C1CCCN1S(=O)(=O)c1cc(C)c(C)cc1C	18.3	0.0	not tested	not tested	18.3	0.0
SN00794007	CL2023	CCOC(=O)c1ccc(cc1)Nc1c(nc2[nH]cnn12)c1ccccc1	18.2	0.0	not tested	not tested	18.2	0.0

Compound ID	Scaffold	Smiles	% Inhibition n=1		% Inhibition n=2		Average % Inhibition	
			24h	48h	24h	48h	24h	48h
SN00787159	CL4175	O=C(NCCC1=CCCCC1)c1oc2CCc3cn[nH]c3c2c1C	18.2	24.4	not tested	not tested	18.2	24.4
SN00783937	CL0031	O=C(NCCc1ccccc1)Cn1cnc(n1)N(=O)=O	18.2	33.8	not tested	not tested	18.2	33.8
SN00801430	CM3061	COc1ccc(cc1)C1CCC(=O)N1CCN1CCOCC1	17.9	34.6	not tested	not tested	17.9	34.6
SN00800980	CM2380A	O=C(N[C@H]1C[C@@H]2C[C@H]1CN2S(=O)(=O)c1ccc2CCCCc2c1)c1cccnc1	17.6	0.6	not tested	not tested	17.6	0.6
SN00802614	CL2573	CCCN(C)C(=O)Cn1c(=O)n(c2ccc(F)cc2)c(=O)c2ccccc12	17.3	32.9	not tested	not tested	17.3	32.9
SN00802548	CM1616	COc1cccc(c1)c1noc(n1)C1CCCCN1C(=O)Nc1ccc(Cl)cc1Cl	17.2	5.7	not tested	not tested	17.2	5.7
SN00784290	CL1665	O=C(NCCCN1CCCCC1)c1cc(nc2ccccc12)c1cccs1	17.2	13.3	not tested	not tested	17.2	13.3
SN00802952	CL1666	O=C(NC1CCCCC1)c1cc(nc2ccccc12)c1ccco1	17.2	20.8	not tested	not tested	17.2	20.8
SN00799943	CM0284	CCCn1ccc2sc(C)c(C(=O)NC3CCCCC3)c2c1=O	17.0	16.0	not tested	not tested	17.0	16.0
SN00803168	CL9065	Cc1ccc(cc1)n1nc(C)c2sc(nc12)N1CCCN(CC1)C(=O)c1ccc2OCOc2c1	16.8	0.0	not tested	not tested	16.8	0.0
SN00801115	CM2362	O=C(N1CCC(C1)c1onc(n1)c1ccccc1)c1cccc2[nH]ccc12	16.7	23.0	not tested	not tested	16.7	23.0
SN00785171	CL5800	CCCN1C(Nc2ccccc2OC)c2ccc(cc2C1=O)C(=O)Nc1ccccc1OC	16.7	21.9	not tested	not tested	16.7	21.9
SN00786288	CL1351	O=C(NCc1cccc(c1)C(F)(F)F)c1[nH]cnc1C(=O)NCc1ccccc1	16.6	20.4	not tested	not tested	16.6	20.4
SN00795497	CL8117	CCOC(=O)N1CCc2c(C1)nc(c1ccc(C)cc1)n(CC(=O)Nc1ccc(Cl)cc1)c2=O	16.6	41.5	not tested	not tested	16.6	41.5
SN00781237	SC013655	CC(COCCOC)NC(=O)Cc1csc(N2CCCNC2=O)n1	16.5	11.2	not tested	not tested	16.5	11.2
SN00786222	CL5430	Cc1ccc(cc1)CNc1nc(nn1C(=O)c1cccc(c1)N(=O)=O)c1ccco1	16.5	6.6	not tested	not tested	16.5	6.6
SN00798864	CL9718	Clc1ccc(cc1)CNS(=O)(=O)c1ccc(s1)c1scc(n1)C(C)C	16.5	0.0	not tested	not tested	16.5	0.0
SN00798558	CL9575	COCCn1cc2CCN(Cc3cccc1c23)C(=O)c1cccc(Cl)c1	16.4	26.2	not tested	not tested	16.4	26.2
SN00800540	CM1417	Cc1nnc(o1)c1nnc(NCc2ccccc2)c2ccccc12	16.4	1.6	not tested	not tested	16.4	1.6
SN00798578	CL9575A	O=C(NC(C)(C)C)N1CCc2cn(C)c3cccc(C1)c23	16.3	7.2	not tested	not tested	16.3	7.2
SN00792731	CL4869	COc1cccc(c1)C1=NC2(CCN(CC2)C(=O)CC)NC1=O	16.3	18.9	not tested	not tested	16.3	18.9
SN00793208	CL7156	COc1ccc(cc1)c1nn2ccn(Cc3cccc(F)c3)c(=O)c2c1	16.3	0.0	not tested	not tested	16.3	0.0
SN00794141	CL8331	COC(=O)c1cn(CC(=O)NCc2cc3OCCCOc3cc2C)c2ccccc12	16.2	14.4	not tested	not tested	16.2	14.4
SN00796215	CL8797A	COc1ccccc1c1onc(n1)C1CCN(CC1)C(=O)COc1ccccc1	16.2	15.9	not tested	not tested	16.2	15.9
SN00787674	CL4851	CC1CC(C)CN(CCCNC(=O)C2CC3CCCN3C32C(=O)Nc2ccccc32)C1	16.2	38.8	not tested	not tested	16.2	38.8
SN00799709	CM0615A	CCOCCn1c(=O)cc(C)nc1N1CCCCC1	16.1	33.8	not tested	not tested	16.1	33.8
SN00784489	CL6277	COc1ccc(CCNC(=O)c2noc(c2)c2cccs2)cc1OC	16.1	33.2	not tested	not tested	16.1	33.2
SN00786260	CL7201	CN1CCN(CC1)C1(CCCCC1)CNC(=O)COc1ccc(C)cc1	16.0	9.4	not tested	not tested	16.0	9.4
SN00794214	CL9260	CCCn1nnc(NCc2ccc(cc2)OCc2ccccc2)n1	16.0	16.1	not tested	not tested	16.0	16.1
SN00794046	CL8310	CCc1nnc2sc(nn12)c1ccc(cc1)CNC(=O)c1ccc(Br)o1	15.9	48.2	not tested	not tested	15.9	48.2
SN00781639	SC014597	COc1ccc(cc1)n1ccn2c(CCC(=O)Nc3cc(C)cc(C)c3)nnc2c1=O	15.9	16.2	not tested	not tested	15.9	16.2
SN00793267	CL4111	Cc1cccc(c1)Cn1c(CNS(=O)(=O)c2ccccc2)nc2cccnc12	15.7	9.9	not tested	not tested	15.7	9.9
SN00797819	CL9384	O=C(N1CCC(CC1)N1CCCCC1)c1cc2c(ccn(C)c2=O)n1C	15.7	18.7	not tested	not tested	15.7	18.7
SN00797239	CL9346	CCN(c1cccc(C)c1)S(=O)(=O)c1cc(sc1C)c1sc(C)c(C)n1	15.6	10.7	not tested	not tested	15.6	10.7
SN00790499	CL8375	CCOc1ccc(cc1)n1c(=O)n(CC(=O)NC2CCCCC2)c2c3cccnc3sc2c1=O	15.6	13.5	not tested	not tested	15.6	13.5
SN00790667	CL8421A	Brc1ccc(cc1)c1noc(n1)c1ccc(o1)CS(=O)(=O)Cc1ccccc1C	15.5	0.0	not tested	not tested	15.5	0.0
SN00789791	CL8042	O=C1CC(CN1c1cccc(c1)c1noc(n1)C1CCC1)C(=O)N1CCN(CC1)c1ccccc1F	15.4	5.7	not tested	not tested	15.4	5.7
SN00802558	CL3392	COC(=O)C(C)n1cnc2n(nnc2c1=O)Cc1ccccc1F	15.4	11.4	not tested	not tested	15.4	11.4
SN00788445	CL6088	Cc1nc2oc(C)c(C(=O)N3CCCC3)c2c(n1)N1CCOCC1	15.4	0.0	not tested	not tested	15.4	0.0
SN00781138	SC013375	O=C(NCCc1noc(n1)c1ccccn1)Cc1ccccc1Cl	15.4	21.7	not tested	not tested	15.4	21.7
SN00800844	CM1524	CCOC(=O)CCNC(=O)N1CCc2c(C1)nc1cc([nH]n1c2=O)c1ccccc1Cl	15.4	13.6	not tested	not tested	15.4	13.6
SN00777931	SC007715	CCc1scc(CN2CCCN(CC2)C(=O)C2CCC2)n1	15.2	3.9	not tested	not tested	15.2	3.9
SN00798186	CL9523	O=C(NC(C)c1ccccc1)c1cc2ccncc2[nH]1	15.0	4.2	not tested	not tested	15.0	4.2
SN00797723	CL8156	CCCOc1ccc(cc1)c1noc(Cc2scc(n2)c2cccc(OC)c2)n1	15.0	4.6	not tested	not tested	15.0	4.6
SN00791279	CL6301	CCOCCCNC(=O)N1Cc2ccccc2n2cccc2C1c1ccccc1	15.0	0.0	not tested	not tested	15.0	0.0
SN00786086	CL7090	O=C(Nc1ccccc1C)c1nc(ncc1N(Cc1ccco1)Cc1sccc1C)S(=O)(=O)C	14.9	11.7	not tested	not tested	14.9	11.7

Compound ID	Scaffold	Smiles	% Inhibition n=1		% Inhibition n=2		Average % Inhibition	
			24h	48h	24h	48h	24h	48h
SN00789967	CL8380	COc1cc(ccc1OC)NC(=O)c1cn(nc1C)c1ccc(cc1)c1noc(n1)C1CCCC1	14.9	25.5	not tested	not tested	14.9	25.5
SN00790723	CL8287	CCCc1nnc(s1)C(=O)Nc1cccc(c1)C(=O)NCc1c[nH]nc1C	14.9	0.0	not tested	not tested	14.9	0.0
SN00801222	CM2676	O=C(c1ccco1)N1CC[C@](O)(c2ccccc2)[C@@H](C1)N(C)C	14.9	3.1	not tested	not tested	14.9	3.1
SN00799218	CL9885	Cc1ccccc1C(=O)N1CCC(CC1)Cc1nnc(o1)c1ccccc1	14.8	12.0	not tested	not tested	14.8	12.0
SN00786292	CL1351	COCCNC(=O)c1nc[nH]c1C(=O)NC(C)C	14.8	8.3	not tested	not tested	14.8	8.3
SN00803240	CL1459	CCN1CCN(CC1)c1ccc2nnc(C(C)C)n2n1	14.8	0.4	not tested	not tested	14.8	0.4
SN00801724	CM1591	Cc1ccc(cc1)C1=NOC2(CCN(CC2)C(=O)c2ccc[nH]2)N1	14.7	0.0	not tested	not tested	14.7	0.0
SN00781610	SC014472	COCCN1CCN(CC1)c1nc(nc2CCCc12)c1ccncc1	14.7	15.0	not tested	not tested	14.7	15.0
SN00801306	CM2426	O=C1CC[C@@]2(CCC[C@H]2N1C)CNC(=O)C1(CC1)c1ccccc1	14.6	21.4	not tested	not tested	14.6	21.4
SN00797759	CL8534	COc1cccc(CNC(=O)c2nsc(C(=O)NCc3ccccc3F)c2N)c1	14.5	13.9	not tested	not tested	14.5	13.9
SN00788425	CL6088	Cc1ccccc1NC(=O)c1c(C)oc2ncnc(N3CCCCC3)c12	14.5	15.9	not tested	not tested	14.5	15.9
SN00794693	CL2560	COCCNC(=O)c1c[nH]c2ccc(cc2c1=O)S(=O)(=O)N(CC)c1ccccc1	14.5	10.4	not tested	not tested	14.5	10.4
SN00802641	CL6284	CCn1nc(cc1c1cc(C)sc1C)C(=O)NCc1ccccc1F	14.5	9.7	not tested	not tested	14.5	9.7
SN00803192	CL9110	Fc1ccc(CNc2ncnc3c(sc(C)c23)C(=O)N2CCCC2)cc1Cl	14.4	23.9	not tested	not tested	14.4	23.9
SN00785355	CL6666	COCCNC(=O)N1Cc2c(C)nn(c3ccccc3)c2n2ccc2C1c1cccc(C)c1	14.4	10.0	not tested	not tested	14.4	10.0
SN00794159	CL1240	COc1ccc(cc1)n1nnnc1C1(CCCCC1)N1CCOCC1	14.4	0.0	not tested	not tested	14.4	0.0
SN00789282	CL7759	COC(=O)c1ccc(cc1)Cn1c(cc2ccccc12)c1nnc(o1)C(C)C	14.4	4.4	not tested	not tested	14.4	4.4
SN00795823	CL5144A	Fc1ccc(cc1)c1nc2sc(nn2c1NC(C)(C)C)N1CCN(CC1)Cc1ccccn1	14.3	32.2	not tested	not tested	14.3	32.2
SN00794256	CL1358	O=C(N1CCCCCC1)c1nc2nc(cc(n2n1)C(F)(F)F)c1ccco1	14.3	19.8	not tested	not tested	14.3	19.8
SN00795352	CL8949	COCCNc1c(nc2sc3cc(C)ccc3n12)c1ccc(OCC)cc1	14.3	0.0	not tested	not tested	14.3	0.0
SN00786962	CL9260	CCn1nnc(NCc2ccc(cc2)c2ccccc2)n1	14.1	14.2	not tested	not tested	14.1	14.2
SN00784546	CL3225	COc1ccc(cc1)c1nc2sccn2c1Nc1ccc2OCOc2c1	13.9	0.0	not tested	not tested	13.9	0.0
SN00786071	CL7202	Fc1ccc(cc1)n1nnc(c1)C(=O)Nc1cc2OCOc2cc1C(=O)C	13.8	7.4	not tested	not tested	13.8	7.4
SN00802824	CL7474	COc1ccc(cc1)C1NC(=O)NC(=C1C(=O)c1ccccc1)C	13.8	12.9	not tested	not tested	13.8	12.9
SN00788526	CL6093	CCCCN(CCCC)S(=O)(=O)c1ccc2NC(=O)CCCc2c1	13.8	27.2	not tested	not tested	13.8	27.2
SN00801392	CM2247	O=C(CCC1CCCC1)N1CCCC(C)(C1)c1onc(n1)C1CC1	13.8	3.8	not tested	not tested	13.8	3.8
SN00798552	CL9575	CC(C)Cn1cc2CCN(Cc3cccc1c23)C(=O)Cc1ccs1	13.7	35.1	not tested	not tested	13.7	35.1
SN00791772	CL3568	COc1ccc(cc1)CNc1onc(n1)c1cccc(OC)c1	13.6	0.0	not tested	not tested	13.6	0.0
SN00787724	CL5093	COc1ccc(cc1)c1noc(CC(=O)N2CCN(CC2)c2ccccn2)n1	13.5	23.4	not tested	not tested	13.5	23.4
SN00777515	SC007474	CC(=O)Nc1ccc(s1)C(=O)OCc1noc(c1)c1cccs1	13.5	14.0	not tested	not tested	13.5	14.0
SN00785276	CL3385	COc1ccc(cc1)c1[nH]c(nc1S(=O)(=O)CC(=O)Nc1ccc(F)cc1)c1ccc(OC)cc1	13.5	12.5	not tested	not tested	13.5	12.5
SN00795955	CL8922	CCCC(=O)Nc1cc(F)c2nc(ccc2c1)N1CCCCC1	13.5	0.0	not tested	not tested	13.5	0.0
SN00796543	CL8891A	Clc1ccc(cc1)n1nnc(C(=O)N2CCCCC2)c1c1cccnc1	13.5	20.4	not tested	not tested	13.5	20.4
SN00789328	CL8003	CCn1nnc2cc(ccc12)C(=O)N(C)CC(=O)NCc1cccc(C)c1	13.5	14.4	not tested	not tested	13.5	14.4
SN00785932	CL5587	CCCN(C(=O)c1cccc(NC(=O)[C@H]2[C@H]3C[C@H]4OC(=O)[C@@H]2[C@H]4C3)c1)c1ccccc1F	13.4	2.8	not tested	not tested	13.4	2.8
SN00801138	CM2941	CCn1ncc(CN2CCC(CC2)OCc2onc(n2)C2CC2)c1	13.3	0.0	not tested	not tested	13.3	0.0
SN00784818	CL2506	CC(=O)c1cccc(c1)NS(=O)(=O)c1c(C)n[nH]c1C	13.3	5.0	not tested	not tested	13.3	5.0
SN00784983	CL2762	CCc1cccc(c1)NC(=O)C1CCCN(C1)S(=O)(=O)c1c(C)noc1C	13.3	0.0	not tested	not tested	13.3	0.0
SN00781548	SC014215	Cc1csc(n1)C1CCCCN1	13.3	6.1	not tested	not tested	13.3	6.1
SN00801505	CM2368	CNC(=O)N1C[C@@H](CN(C)C)[C@H](C1)C1CCN(CCC(C)C)CC1	13.1	0.0	not tested	not tested	13.1	0.0
SN00800077	CM0328	CN(C)C(=O)c1ccc(nc1)OC1CCN(CC1)C(=O)c1ccco1	13.1	13.6	not tested	not tested	13.1	13.6
SN00786799	CL1131A	Cc1ccc(cc1)C1=CC(c2ccccc2)n2nnnc2N1CC(=O)N1CCN(CC1)c1ccccc1	13.0	34.8	not tested	not tested	13.0	34.8
SN00781270	SC013929	C1NCC2CCCN2CC1	12.9	3.1	not tested	not tested	12.9	3.1
SN00794188	CL9259A	O=C(Cc1ccccc1F)N1CCCN(CC1)Cc1noc(n1)c1ccccc1	12.9	11.3	not tested	not tested	12.9	11.3
SN00785631	CL5700A	O=C(CN(C)c1nn2c(nc(C)cc2=O)s1)NC1CCCc2ccccc12	12.9	35.0	not tested	not tested	12.9	35.0
SN00775388	SC001417	CCOC(=O)c1sc2nc(CN3CCCCC3)nc(NCCC(=O)OC)c2c1C	12.8	0.0	not tested	not tested	12.8	0.0

Compound ID	Scaffold	Smiles	% Inhibition n=1		% Inhibition n=2		Average % Inhibition	
			24h	48h	24h	48h	24h	48h
SN00802785	CL2346	Cc1ccc(c(C)c1)n1nc2CS(=O)(=O)Cc2c1NC(=O)COc1ccccc1	12.8	16.6	not tested	not tested	12.8	16.6
SN00789595	CL4272B	CC(C)c1ccc(cc1)S(=O)(=O)N1CCN(CC1)c1cc(=O)n2nc(sc2n1)c1ccccc1	12.8	0.0	not tested	not tested	12.8	0.0
SN00774089	SC000765	Cc1nn(Cc2csc(n2)c2ccsc2)c(C)c1S(=O)(=O)N1CCc2ccccc12	12.8	7.1	not tested	not tested	12.8	7.1
SN00792043	CL7144	O=S(=O)(N1CCCCC1)c1ccc(cc1)c1cnc(o1)C1CC1	12.8	8.3	not tested	not tested	12.8	8.3
SN00786646	CL9247	Cc1csc(n1)n1nc(C)c(CC(=O)N2CCN(CC2)c2ccccc2)c1O	12.8	34.3	not tested	not tested	12.8	34.3
SN00802455	CL7521	COc1cc(ccc1OC)c1ccnc2c(cnn12)C(=O)NCc1ccc2OCOc2c1	12.7	16.8	not tested	not tested	12.7	16.8
SN00783829	CL6785C	Cc1ccc(cc1)c1cc(C(F)F)n2ncc(C(=O)NCc3cnn(C)c3C)c2n1	12.7	0.0	not tested	not tested	12.7	0.0
SN00786878	CL9434	COc1ccc(cc1)NC(=O)N1CCN(CC1)c1cc(C)nc(n1)N1CCOCC1	12.7	44.4	not tested	not tested	12.7	44.4
SN00774920	SC000986	O=c1[nH]c2ccccc2c(c1)C(=O)N1CCN(CC1)Cc1ccc2OCOc2c1	12.7	0.0	not tested	not tested	12.7	0.0
SN00798700	CL9653	CCn1cc(C(=O)Nc2ccc(F)cc2)c(n1)S(=O)(=O)N1CCC(C)CC1	12.6	0.0	not tested	not tested	12.6	0.0
SN00781153	SC013399	O=S(=O)(Cc1ccc(cc1)C(F)(F)F)Cc1noc(n1)C1CC1	12.6	0.0	not tested	not tested	12.6	0.0
SN00799416	CL4965A	COc1ccc(nn1)c1cccc(c1)NC(=O)c1ccccc1Br	12.5	16.0	not tested	not tested	12.5	16.0
SN00796597	CL9302	Cc1ccc2oc(c3noc(n3)C3CC(=O)N(C3)c3ccccc3)c(C)c2c1	12.5	2.9	not tested	not tested	12.5	2.9
SN00797236	CL9346	Cc1nc(sc1C)c1sc(C)c(c1)S(=O)(=O)N1CCCCCC1	12.5	11.8	not tested	not tested	12.5	11.8
SN00802904	CL6517	Cc1ccc(cc1)Cn1ccc2nc(ncc2c1=O)N1CCCCC1	12.4	15.1	not tested	not tested	12.4	15.1
SN00775741	SC002601	CCOc1cc(ccc1OC(F)F)C(=O)Nc1ccc(cc1)c1csc2=NCCCn12	12.4	4.5	not tested	not tested	12.4	4.5
SN00799546	CL9527	Cc1cccc(c1)c1nc2sc(nn2c1NC(=O)C1CC1)N1CCCCC1	12.4	23.2	not tested	not tested	12.4	23.2
SN00796291	CL9005	O=C(Nc1ccc2c(CCN2S(=O)(=O)C)c1)Nc1cccc(F)c1C	12.4	21.1	not tested	not tested	12.4	21.1
SN00788415	CL5991	O=C(Nc1ccc(nc1)S(=O)(=O)N)c1c(C)oc2ncn(C(C)c3ccccc3)c(=O)c12	12.3	0.0	not tested	not tested	12.3	0.0
SN00784896	CL3088	CSc1cccc(c1)NC(=O)CN1C(=O)C(C)N=C(c2ccccc2)c2ccccc12	12.2	0.0	not tested	not tested	12.2	0.0
SN00784397	CL7855	CCOc1ccc(cc1)NS(=O)(=O)c1n[nH]c(c1)C(=O)Nc1cc(C)ccc1C	12.2	15.3	not tested	not tested	12.2	15.3
SN00797319	CL9333	Cc1cccc(c1)CC(=O)N1CCN(CC1)c1nc2cccnc2n(Cc2ccccc2)c1=O	12.1	38.8	not tested	not tested	12.1	38.8
SN00789223	CL6673B	Clc1ccc2NC3(CCN(CC3)S(=O)(=O)c3ccccc3C)c3cccn3c2c1	12.0	20.1	not tested	not tested	12.0	20.1
SN00789358	CL3664	CCOc1c(cccc1OCC)CC(=O)Nc1ccc2c(c1)c(=O)n(C)c1=NCCn21	12.0	35.6	not tested	not tested	12.0	35.6
SN00785782	CL1320	CN(C)CCn1c(=O)c2[nH]cnc2n(C)c1=O	12.0	13.4	not tested	not tested	12.0	13.4
SN00795483	CL8117	Fc1cccc(c1)NC(=O)Cn1c(C)nc2CN(CCc2c1=O)Cc1ccccc1	12.0	0.0	not tested	not tested	12.0	0.0
SN00801060	CM3105	O=C(Cc1ccccc1F)N1CCC(Nc2cccnc2)C1	11.9	18.5	not tested	not tested	11.9	18.5
SN00801147	CM2941	O=C(N1CCC(OCc2onc(n2)c2ccncc2)CC1)c1ccco1	11.9	18.6	not tested	not tested	11.9	18.6
SN00782463	SC008887	Cc1noc(c1)C(=O)N1CCCN(CC1)Cc1cnn(C)c1	11.9	0.0	not tested	not tested	11.9	0.0
SN00797828	CL9384	Cc1ccc(cc1)NC(=O)c1cc2c(ccn(CC(C)C)c2=O)n1C	11.9	17.5	not tested	not tested	11.9	17.5
SN00792212	CL4121	COc1ccc(cc1)CN1CCC2(CC1)NC(=O)C(=N2)c1ccc(C)cc1	11.8	16.2	not tested	not tested	11.8	16.2
SN00807951	SC015097	Cc1ncc2CCN(Cc2n1)C(C)c1onc(n1)c1cccs1	11.8	13.5	not tested	not tested	11.8	13.5
SN00800322	CM1254	O=C(N1CCN(CC1)c1ccccc1)c1cccc(n1)N1CCNC(C1)c1ccccc1	11.8	13.5	not tested	not tested	11.8	13.5
SN00798137	CL9487	COc1ccc(cc1)n1nnc(c2nsc(Nc3ccc(Cl)cc3)n2)c1C	11.8	11.6	not tested	not tested	11.8	11.6
SN00792113	CL6984B	CCOC(=O)c1ccccc1NC(=O)NC(c1ccccc1)c1nnc(o1)c1ccccc1	11.8	0.0	not tested	not tested	11.8	0.0
SN00784190	CL3664	O=C(Nc1ccc2c(c1)c(=O)n(C)c1=NCCn21)c1ccc2OCOc2c1	11.7	31.6	not tested	not tested	11.7	31.6
SN00792767	CL7220	CN1CCN(CCNC(=O)CN2C(=O)C3CCCCN3c3ccc(cc23)C(=O)N2CCCC2)CC1	11.7	13.0	not tested	not tested	11.7	13.0
SN00790682	CL7232	Fc1ccc(cc1)NC(=O)CC1COc2ccccc2N1C	11.6	7.6	not tested	not tested	11.6	7.6
SN00797895	CL9438	Cc1ccc(cc1)n1nc(C)c2c(=O)n(ccc12)[CH]C(=O)NCCC1=CCCCC1	11.6	0.0	not tested	not tested	11.6	0.0
SN00802831	CL7971	CCOC(=O)c1csc(n1)n1nc(CC(C)C)cc1C(F)(F)F	11.5	29.0	not tested	not tested	11.5	29.0
SN00801690	CM1468	COc1ccc(cc1)C1(CC1)C(=O)N1CCC(CC1)Cc1onc(n1)c1ccc(F)cc1	11.4	0.0	not tested	not tested	11.4	0.0
SN00795100	CL8526	O=S(=O)(NCCC1=CCCCC1)c1ccc(s1)c1cnc(o1)C1CCCC1	11.4	27.9	not tested	not tested	11.4	27.9
SN00798474	CL9915	COc1cc(ccc1OC)C(=O)Nc1cc(sc1C)c1scc(C)n1	11.4	27.2	not tested	not tested	11.4	27.2
SN00793004	CL6673	COc1ccc(cc1)NC(=O)N1CCC2(CC1)Nc1cc(C)ccc1n1cccc21	11.3	10.6	not tested	not tested	11.3	10.6
SN00789565	CL7994	Cc1ccc(cc1)NC(=O)Cn1c2cccnc2c(=O)n(Cc2ccc3OCOc3c2)c1=O	11.3	0.0	not tested	not tested	11.3	0.0
SN00797922	CL7988	CCS(=O)(=O)c1nc2ccc(cc2s1)NS(=O)(=O)c1ccc2OCCOc2c1	11.3	0.0	not tested	not tested	11.3	0.0

Compound ID	Scaffold	Smiles	% Inhibition n=1		% Inhibition n=2		Average % Inhibition	
			24h	48h	24h	48h	24h	48h
SN00771952	SC009997	CSc1ccc(Cl)c(c1)C(=O)N1CCCN(CC1)C(=O)c1ccncc1	11.3	45.6	not tested	not tested	11.3	45.6
SN00788220	CL3220	COc1ccc(cc1)c1n[nH]c(c1)C(=O)N(C)Cc1ccco1	11.3	44.3	not tested	not tested	11.3	44.3
SN00786016	CL6860	CCCOc1ccc(cc1)C1OCCn2c1c1n(C)c(=O)n(C)c(=O)c1c2c1ccc(OC)cc1	11.3	14.8	not tested	not tested	11.3	14.8
SN00784101	CL7511	COc1cccc(c1)C(=O)NC1(C(=O)N(Cc2ccccc2)C(=C1C(=O)C)C)C(F)(F)F	11.2	2.9	not tested	not tested	11.2	2.9
SN00792956	CL7291	COc1ccccc1c1n[nH]c(c1)C(=O)Nc1sc(Nc2ccccc2)nc1C(=O)N	11.2	11.1	not tested	not tested	11.2	11.1
SN00798670	CL9618	Fc1ccc(cc1)C(=O)NCc1ccc2c(nnn2C)c1	11.2	3.3	not tested	not tested	11.2	3.3
SN00792172	CL6075	O=C(Nc1ccccc1)CS(=O)(=O)c1ccc2NC=NS(=O)(=O)c2c1	11.2	15.6	not tested	not tested	11.2	15.6
SN00793330	CL2572	Fc1ccc(cc1)N1CCN(CC1)S(=O)(=O)c1ccc2c(CCCN2C(=O)C)c1	11.2	0.2	not tested	not tested	11.2	0.2
SN00781438	SC014188	O=C(Cn1c(=O)onc1c1ncccn1)N1CCCCC1	11.1	1.7	not tested	not tested	11.1	1.7
SN00792912	CL6070	Cc1ccc(cc1)NC(=O)Cn1c(C)cc(C)c(c2onc(n2)c2ccc3OCOc3c2)c1=O	11.1	3.6	not tested	not tested	11.1	3.6
SN00785456	CL3544	O=C(Nc1c(C)cccc1C)Cn1c(=O)n(CCc2c[nH]c3ccccc23)c(=O)c2cccnc12	11.1	0.5	not tested	not tested	11.1	0.5
SN00800335	CM1204A	Clc1ccc(cc1)c1n[nH]c(c1)N1CCN(CC1)C(=O)c1ccccc1	11.1	14.4	not tested	not tested	11.1	14.4
SN00796500	CL4472	CCc1nc(sc1C(=O)Nc1ccc(C)c(C)c1)N1CCCC1=O	11.1	0.0	not tested	not tested	11.1	0.0
SN00798903	CL9733	CCC(C)NC(=O)C1CCN(CC1)c1cnc(nc1)N1CCSCC1	11.0	12.6	not tested	not tested	11.0	12.6
SN00785973	CL6446	O=C(Nc1ccccc1C)c1ccc2c(CCN2S(=O)(=O)C)c1	10.8	31.2	not tested	not tested	10.8	31.2
SN00783826	CL4751	CCCNC(=O)c1cnn2c(cc(nc12)c1ccc(C)cc1)C(F)F	10.8	40.8	not tested	not tested	10.8	40.8
SN00791007	CL7785B	COc1ccccc1c1n[nH]c(c1)C(=O)N1CCCN(CC1)c1ccc(=O)n(n1)c1ccc(C)c(C)c1	10.8	8.7	not tested	not tested	10.8	8.7
SN00791866	CL5759	Fc1ccc(cc1)C1=NCC(=O)N(CC(=O)Nc2ccc(Cl)cc2)c2sc(C)c(C)c12	10.8	0.0	not tested	not tested	10.8	0.0
SN00794982	CL5601	Clc1ccc(cc1)Cn1c(=O)c2nc(nn2c2ccc(C)cc12)C(=O)NCCc1cccs1	10.7	29.9	not tested	not tested	10.7	29.9
SN00802718	CL1320	Fc1ccc(cc1)Cn1cnc2c1c(=O)n(C1CCCC1)c(=O)n2c1ccccc1	10.7	6.1	not tested	not tested	10.7	6.1
SN00769384	SC000391	COc1ccc(cc1)c1scc(CN2C(=O)N(C(C)C)C(=O)C2=O)n1	10.7	0.0	not tested	not tested	10.7	0.0
SN00801268	CM2348	CN(C)CC1CN(CC21CCOCC2)S(=O)(=O)C1CC1	10.7	16.1	not tested	not tested	10.7	16.1
SN00793251	CL4110	CC(C)CC(=O)NCc1nc2cccnc2n1Cc1ccc(F)cc1	10.6	8.4	not tested	not tested	10.6	8.4
SN00796451	CL9209A	Cc1ccc(COCc2nn(C)c3CCN(Cc23)S(=O)(=O)c2ccc(F)c(C)c2)cc1	10.6	2.1	not tested	not tested	10.6	2.1
SN00790679	CL7232	O=C(CC1COc2ccccc2N1Cc1ccccc1)N1CCc2ccccc2C1	10.6	0.0	not tested	not tested	10.6	0.0
SN00790039	CL8242A	O=C(NCCC1=CCCCC1)C1CCN(CC1)c1nc2cccnc2n(Cc2ccccc2)c1=O	10.6	11.4	not tested	not tested	10.6	11.4
SN00775659	SC002205	O=C(NCC(N(C)C)c1ccccc1)C1CCN(CC1)S(=O)(=O)c1c(C)noc1C	10.5	0.0	not tested	not tested	10.5	0.0
SN00790732	CL8287	CCOc1ccc(cc1)CNC(=O)c1cccc(c1)NC(=O)c1nnc(s1)C1CC1	10.5	0.0	not tested	not tested	10.5	0.0
SN00802559	CL2849	Clc1ccc(cc1)C(=O)N1CCCC(C1)C(=O)N[C@@H]1CCCC[C@H]1NC(=O)c1ccccc1C	10.4	3.9	not tested	not tested	10.4	3.9
SN00794728	CL3961	CCOC(=O)c1cc(c2ccccc2)n(CCC(=O)N2CCn3c2nc2ccccc32)c1C	10.4	10.1	not tested	not tested	10.4	10.1
SN00800013	CM0322	CN1CCC(CC1)Oc1ncccc1C(=O)N1CCOCC1	10.4	15.5	not tested	not tested	10.4	15.5
SN00786283	CL2028	O=C(Nc1ccc(C)c(C)c1)c1noc(c1)c1ccc2OCCOc2c1	10.4	2.9	not tested	not tested	10.4	2.9
SN00787260	CL3683	O=C(Nc1nnc(s1)S(=O)(=O)N1CCc2ccccc12)c1ccccc1	10.4	37.8	not tested	not tested	10.4	37.8
SN00802034	CM1159	CC(C)n1cc2sc(cc2c1)C(=O)NCCCN1CCCc2ccccc12	10.4	3.6	not tested	not tested	10.4	3.6
SN00798971	CL9739	CCCn1c(cc(=O)n2nccc12)C1CCN(CC1)C(=O)Nc1ccc(F)cc1	10.3	19.1	not tested	not tested	10.3	19.1
SN00800535	CM1467	Cc1ccc(cc1)CNC(=O)N1CCCN(CC1)c1ncccc1c1onc(n1)C1CC1	10.3	34.3	not tested	not tested	10.3	34.3
SN00782468	SC009077	Cn1ncc2c(ncnc12)N1CCCN(CC1)C1CC1	10.3	9.8	not tested	not tested	10.3	9.8
SN00789029	CL7690	COc1ccccc1C(=O)n1ccn(c1=O)S(=O)(=O)c1ccc(C)cc1	10.3	6.3	not tested	not tested	10.3	6.3
SN00786247	CL7201	O=C(COc1ccc(Br)cc1)NCC1(CCCCC1)N(C)C	10.3	5.1	not tested	not tested	10.3	5.1
SN00790089	CL7825A	COc1cc(OC)cc(c1)NC(=O)C1CCCN(C1)c1nc2ccccc2[nH]c1=O	10.2	0.0	not tested	not tested	10.2	0.0
SN00790594	CL8392	Cc1ccc(cc1)Cn1nnc2c(=O)n(cnc12)Cc1onc(n1)c1ccccc1	10.2	2.1	not tested	not tested	10.2	2.1
SN00800575	CM1424	O=C(COc1ccccc1)Nc1ccc2c(c1)Sc1ncccc1C(=O)N2C	10.2	15.1	not tested	not tested	10.2	15.1
SN00794732	CL5794	Cc1ccc(cc1)NS(=O)(=O)c1ccc(cc1)n1cccn1	10.2	6.0	not tested	not tested	10.2	6.0
SN00795318	CL8523A	O=C(N(C)Cc1ccccc1)c1cc(cnc1N1CCNCC1)NC(=O)c1ccccc1C	10.2	15.2	not tested	not tested	10.2	15.2
SN00778835	SC008770	Cc1ccc(cc1)S(=O)(=O)C1(CCC1)C(=O)Nc1c[nH]nc1	10.2	0.0	not tested	not tested	10.2	0.0
SN00782469	SC009077	CCN1CCCN(CC1)c1ncnc2n(C)nc(Br)c12	10.1	4.4	not tested	not tested	10.1	4.4

Compound ID	Scaffold	Smiles	% Inhibition n=1		% Inhibition n=2		Average % Inhibition	
			24h	48h	24h	48h	24h	48h
SN00791365	CL6564	COCCN1C(=O)c2oc3ccccc3c2OCC1(C)C(=O)NCc1ccccc1OC	10.1	21.5	not tested	not tested	10.1	21.5
SN00798517	CL9569	COc1ccc(cc1)CNC(=O)c1[nH]c2cc(C)sc2c1CN1CCCC1	10.1	35.5	not tested	not tested	10.1	35.5
SN00797693	CL7650	Cc1ccc(cc1)c1nnc(o1)c1cccnc1N1CCCC1	10.0	0.0	not tested	not tested	10.0	0.0
SN00792238	CL6617	Cc1ccc(cc1C)S(=O)(=O)Nc1ccc2CCCN(C(=O)c3ccco3)c2c1	9.9	12.2	not tested	not tested	9.9	12.2
SN00777029	SC006675	Cc1ccc(cc1)NC(=O)Cc1scc(n1)c1ccc2c(c1)CC(=O)N2C	9.8	10.0	not tested	not tested	9.8	10.0
SN00793559	CL6605C	COc1ccc(cc1S(=O)(=O)N1CCN(CC1)c1nc(C)cc(n1)N1CCOCC1)C(C)(C)C	9.8	5.9	not tested	not tested	9.8	5.9
SN00796018	CL8222	COc1cccc(c1)n1ccn2cc(nc2c1=O)c1ccc(OC)c(OC)c1	9.8	36.9	not tested	not tested	9.8	36.9
SN00793821	CL8307	CCc1noc(c1)C1CCCN1C(=O)c1cnn2c1nc(cc2C(F)(F)F)c1cccs1	9.7	1.7	not tested	not tested	9.7	1.7
SN00771236	SC007963	CCC1N(CCc2sccc12)C(=O)C1CCN(CC1)S(=O)(=O)c1ccc(Cl)c(c1)N(=O)=O	9.7	27.8	not tested	not tested	9.7	27.8
SN00807797	SC012695	Cn1ncc2c(=O)[nH]c(CN3CCN(CC3)c3cnccn3)nc12	9.7	0.0	not tested	not tested	9.7	0.0
SN00798629	CL9613	Clc1ccc(cc1)S(=O)(=O)Nc1cc(sc1C)c1nnc(C)o1	9.7	2.8	not tested	not tested	9.7	2.8
SN00774045	SC000748	Cc1noc(n1)CN1C(=O)NC(C)(c2ccccc2)C1=O	9.7	0.0	not tested	not tested	9.7	0.0
SN00780570	SC011706	CCOc1ccccc1OC1CCN(CC1)c1ncnc2nc[nH]c12	9.7	31.1	not tested	not tested	9.7	31.1
SN00783344	CL1650	NC(=O)c1nn2c(nc(cc2C(F)(F)F)c2ccccc2)c1Br	9.6	38.7	not tested	not tested	9.6	38.7
SN00801518	CM2918	CN(C1CCN(CC1)S(=O)(=O)C(C)C)C1CCOCC1	9.6	0.0	not tested	not tested	9.6	0.0
SN00797950	CL7988	CCCS(=O)(=O)c1nc2ccc(cc2s1)NS(=O)(=O)CCc1ccccc1	9.6	20.2	not tested	not tested	9.6	20.2
SN00774154	SC000773	O=C(Nc1cnn(c1)Cc1nc2ccccn2c1)Nc1ccc(F)cc1F	9.6	7.2	not tested	not tested	9.6	7.2
SN00778504	SC008248	Fc1ccc(cc1)S(=O)(=O)N1CCCN(CC1)C(=O)c1sc(C)nc1c1ccccc1	9.6	9.7	not tested	not tested	9.6	9.7
SN00787811	CL3220A	COc1ccccc1N1CCN(CC1)C(=O)c1[nH]nc(c1)C1CC1	9.6	0.0	not tested	not tested	9.6	0.0
SN00797707	CL7650	COc1ccc(cc1)c1nnc(o1)c1cccnc1N1CCN(CC1)C(=O)c1cccc(OC)c1	9.6	2.3	not tested	not tested	9.6	2.3
SN00775178	SC001115	COC(=O)c1cccc2N(CCCc12)C(=O)C1CC(=O)N(CCc2ccccc2)C1	9.6	0.0	not tested	not tested	9.6	0.0
SN00794761	CL8472	O=C(NCc1ccccc1)c1ccc(cc1)c1ncnc(c1)N1CCOCC1	9.5	12.2	not tested	not tested	9.5	12.2
SN00800269	CM1091	O=C(CCc1ccccc1)N1CCc2n[nH]c(C(=O)N(C)C)c2C1	9.5	0.0	not tested	not tested	9.5	0.0
SN00783378	CL2139	O=C1CN(c2nc(nc(n2)N(C)C)N(C)C)C(=N)N1c1ccc(Cl)c(Cl)c1	9.5	16.0	not tested	not tested	9.5	16.0
SN00788993	CL5326	O=C(NCCC1=CCCCC1)c1cccn1c1cccnc1	9.5	14.0	not tested	not tested	9.5	14.0
SN00785656	CL4403A	CCCCCCNC(=O)Cc1c(C)nc2n(nc(C)c2c2ccccc2)c1C	9.5	17.0	not tested	not tested	9.5	17.0
SN00790579	CL8391	Fc1cccc(c1)c1noc(n1)Cn1ccn2nc(cc2c1=O)c1cccs1	9.5	8.1	not tested	not tested	9.5	8.1
SN00793567	CL7356	COc1cccc(CNC(=O)Cn2nc(ccc2=O)C(=O)Nc2ccccc2)c1	9.4	4.7	not tested	not tested	9.4	4.7
SN00784128	CL1655	O=C(Nc1cccc(c1)C(F)(F)F)c1cnn2c1nc(cc2C(F)(F)F)c1ccco1	9.4	9.2	not tested	not tested	9.4	9.2
SN00796748	CL9208A	CCc1ccc(cc1)NC(=O)N1CCc2n(C)nc(c3onc(C)n3)c2C1	9.4	19.4	not tested	not tested	9.4	19.4
SN00782691	SC016130	CCC(OC1CCCC1)C(=O)N1CCN(CC1)c1nccn1C	9.4	0.0	not tested	not tested	9.4	0.0
SN00789606	CL4272B	COc1ccc(cc1)S(=O)(=O)N1CCN(CC1)c1cc(=O)n2nc(sc2n1)c1ccccc1C	9.4	10.6	not tested	not tested	9.4	10.6
SN00774624	SC000953	O=C(NCCN1CCCCCC1=O)C1CCCN1C(=O)C1CCCC1	9.3	0.0	not tested	not tested	9.3	0.0
SN00784585	CL2049	CC(C)CCNC(=O)C1(C)CCC(=O)N1CCCN1CCN(CC1)c1cccc(Cl)c1	9.3	25.4	not tested	not tested	9.3	25.4
SN00798692	CL9653	CCCNC(=O)c1cn(C)nc1S(=O)(=O)N1CCC(C)CC1	9.3	3.5	not tested	not tested	9.3	3.5
SN00802568	CL3391	Clc1ccc(cc1)Cn1nnc2c(=O)n(cnc12)CC(=O)NC1CCCC1	9.3	16.4	not tested	not tested	9.3	16.4
SN00794771	CL8480	O=C(NCCc1ccccc1)C1CCCN1C(=O)c1cccc(c1)NC(=O)c1cccs1	9.3	3.7	not tested	not tested	9.3	3.7
SN00776112	SC003062	CCn1c2ccccc2nc1C1NC(=Nc2nc3ccccc3n12)N	9.2	5.4	not tested	not tested	9.2	5.4
SN00795093	CL8526	O=S(=O)(N1CCC2(OCCO2)CC1)c1ccc(s1)c1cnc(o1)C1CCC1	9.2	7.0	not tested	not tested	9.2	7.0
SN00790613	CL8392	COc1ccccc1c1noc(n1)Cn1cnc2n(nnc2c1=O)Cc1ccccc1	9.2	6.5	not tested	not tested	9.2	6.5
SN00793629	CL5710	CC(C)OCCCNC(=O)Cn1c(=O)c(=O)n(Cc2ccncc2)c2ncccc12	9.2	13.1	not tested	not tested	9.2	13.1
SN00783884	CL0192	COc1ccc(OCCn2c(NC(=O)c3ccc(OC)cc3)nc3ccccc23)cc1	9.2	19.5	not tested	not tested	9.2	19.5
SN00799772	CM0219	O=C(Nc1cnc(nc1)c1ccccc1)c1ccccc1C(F)(F)F	9.1	0.0	not tested	not tested	9.1	0.0
SN00797600	CL9224	O=C(NCC1CCCO1)Nc1ccc2sc(nc2c1)NS(=O)(=O)c1ccccc1	9.1	23.5	not tested	not tested	9.1	23.5
SN00786904	CL9235	O=C(Nc1ccccc1)c1cc(c2cccs2)n(n1)C1CCS(=O)(=O)C1	9.1	1.7	not tested	not tested	9.1	1.7
SN00796355	CL8891	Clc1ccccc1n1nnc(C(=O)N2CCc3ccccc3C2)c1c1ccncc1	9.1	30.9	not tested	not tested	9.1	30.9

Compound ID	Scaffold	Smiles	% Inhibition n=1		% Inhibition n=2		Average % Inhibition	
			24h	48h	24h	48h	24h	48h
SN00789838	CL7967	O=C(C1CCCC1)N1CCc2n(C)nc(C(=O)NCc3ccc4OCOc4c3)c2C1	9.1	7.0	not tested	not tested	9.1	7.0
SN00783837	CL6785B	COc1ccc(cc1)c1cc(C(F)F)n2ncc(C(=O)N(C)Cc3cn(CC)nc3C)c2n1	9.1	29.0	not tested	not tested	9.1	29.0
SN00799590	CL5620	O=C(N1CCN(CC1)c1ccccc1)c1cc2CN(CCc2n1C)Cc1ccccc1	9.0	1.6	not tested	not tested	9.0	1.6
SN00791196	CL6082	O=C(NC1CCCC1)Cn1c2ncccc2c(=O)n1c1ccccc1	9.0	18.2	not tested	not tested	9.0	18.2
SN00790417	CL7771A	Cc1ccc(cc1)CN1CCN(C(=O)C2CCC2)c2cccnc12	9.0	4.2	not tested	not tested	9.0	4.2
SN00785773	CL5363	Cc1ccc2nc([nH]c2c1)C1=C(N)N(CC1=O)C1CCCCCC1	9.0	29.0	not tested	not tested	9.0	29.0
SN00771916	SC009352	Cn1ncc2c(ncnc12)NC1CCN(CC1)Cc1ccncc1	8.9	0.0	not tested	not tested	8.9	0.0
SN00774555	SC000912	O=C(Cc1csc(n1)c1cccnc1)N(Cc1ccccc1)C1CCCN(C1)C(=O)OC(C)(C)C	8.9	0.0	not tested	not tested	8.9	0.0
SN00792575	CL4264	CCn1cc(C(=O)NCc2ccccc2)c(=O)c2ccc(C)nc12	8.9	6.1	not tested	not tested	8.9	6.1
SN00782363	SC007099	COc1ccc(cn1)CNC(=O)N1CCN(C)CC1c1ccccc1	8.9	0.0	not tested	not tested	8.9	0.0
SN00788081	CL5043	O=C(Nc1ccc(C)c(C)c1)Cn1nnc(c2onc(n2)c2ccccc2)c1N	8.9	2.5	not tested	not tested	8.9	2.5
SN00788641	CL7412	O=c1c2c(c3ccccc3)c(Nc3ccccc3)oc2n(C)c(=O)n1C	8.8	9.4	not tested	not tested	8.8	9.4
SN00801641	CM2805	CCc1ccc(cc1)NC(=O)Cn1nc2CCCCn2c1=O	8.8	9.1	not tested	not tested	8.8	9.1
SN00787900	CL5562	Fc1ccc(cc1)N1C(=O)CN(CC1(C)C(=O)NC1CCCCCCC1)S(=O)(=O)C	8.7	11.9	not tested	not tested	8.7	11.9
SN00801264	CM2348	CN(C)CC1CN(CC21CCOCC2)S(=O)(=O)c1ccccc1	8.7	0.0	not tested	not tested	8.7	0.0
SN00799453	CL4071	Cc1ccc(cc1)NC(=O)c1cnc2c(c1)c(=O)n(C)c(=O)n2c1ccccc1	8.7	0.0	not tested	not tested	8.7	0.0
SN00802737	CL4868A	O=C1NC2(CCN(CC2)S(=O)(=O)c2ccc(cc2)C(C)(C)C)Nc2c(C)cccc12	8.7	13.3	not tested	not tested	8.7	13.3
SN00784647	CL2048	COc1ccc(cc1)N1C(=O)NC2CC1(C)Oc1ccc(OC)cc21	8.7	11.2	not tested	not tested	8.7	11.2
SN00797402	CL9351A	COc1ccc(cc1)CNC(=O)c1ccc(cc1)c1noc(n1)C1CCCN(C1)C(=O)c1ccccc1	8.7	14.7	not tested	not tested	8.7	14.7
SN00794411	CL2352	CCCNc1ncnc2c1cnn2c1ccc(C)cc1	8.6	0.0	not tested	not tested	8.6	0.0
SN00788055	CL5555	CC1CC(C)CN(C1)C(=O)Cn1cnc2c(nc3CCCCn23)c1=O	8.6	0.0	not tested	not tested	8.6	0.0
SN00798224	CL9549	CCN1CCc2[nH]c3ccc(NC(=O)c4ccn(CC)n4)cc3c2C1	8.6	1.1	not tested	not tested	8.6	1.1
SN00785806	CL7499	O=C1c2ccccc2C(=O)N1CC(=O)n1nc(nc1NCc1cccs1)c1cccnc1	8.6	3.6	not tested	not tested	8.6	3.6
SN00799110	CL9760	CCCN(C)C(=O)c1ccc(cc1)c1ccnc(n1)N1CCCCC1	8.6	0.0	not tested	not tested	8.6	0.0
SN00783529	CM2887	COc1cc(cc(OC)c1OC)C(=O)NCC1(CCOCC1)c1ccccc1	8.5	6.5	not tested	not tested	8.5	6.5
SN00807940	SC015096	CCc1ncc2CCN(Cc3csc(n3)c3ncccn3)Cc2n1	8.5	9.1	not tested	not tested	8.5	9.1
SN00788529	CL6093	O=C1CCCc2cc(ccc2N1)S(=O)(=O)N1CCN(CC1)c1ccccc1	8.5	19.1	not tested	not tested	8.5	19.1
SN00782472	SC009212	O=C(COc1ccccc1C)N1CCN(CC1)Cc1nnc2CCCCCn12	8.5	0.5	not tested	not tested	8.5	0.5
SN00799511	CL6598	Fc1ccc(cc1)OCC(=O)N1CCN(CC1)c1scc(CN2CCCC2)n1	8.5	1.1	not tested	not tested	8.5	1.1
SN00787421	CL4156	Clc1ccc(cc1)Cn1cc2CCc3oc(C(=O)NCCCN4CCOCC4)c(C)c3c2n1	8.5	1.0	not tested	not tested	8.5	1.0
SN00786609	CL9233	O=S1(=O)C[C@H](NCc2ccccn2)[C@H](C1)S(=O)(=O)c1ccccc1	8.4	0.0	not tested	not tested	8.4	0.0
SN00799000	CL9740	COc1ccc(cc1)c1ncc2CN(CCc2n1)C(=O)c1ccccc1C(F)(F)F	8.4	0.0	not tested	not tested	8.4	0.0
SN00800120	CM0325	CN(C)C(=O)c1ccc(nc1)OC1CCN(C1)Cc1ccccn1	8.4	6.6	not tested	not tested	8.4	6.6
SN00802781	CL2346	COc1ccc(cc1)n1nc2CS(=O)(=O)Cc2c1NC(=O)c1ccc(cc1)N(=O)=O	8.3	12.4	not tested	not tested	8.3	12.4
SN00807792	SC012695	O=C(N(C)C)N1CCN(CC1)Cc1[nH]c(=O)c2cnn(C)c2n1	8.3	0.0	not tested	not tested	8.3	0.0
SN00797832	CL9388	CCC(C)NC(=O)c1cc2c(ccn(Cc3ccccc3)c2=O)s1	8.3	11.3	not tested	not tested	8.3	11.3
SN00785076	CL2596	COc1ccc(cc1)n1nc(C(=O)NCc2ccc(C)cc2)c2c(c1=O)n(C)c1ccccc21	8.3	5.1	not tested	not tested	8.3	5.1
SN00807767	CL1058	COc1ccc(c(OC)c1)N1C(=O)c2ccncc2C1=O	8.3	28.6	not tested	not tested	8.3	28.6
SN00779817	SC010465	CCc1ncncc1C(=O)N1CCN(CC1)Cc1onc(n1)C(C)C	8.3	26.6	not tested	not tested	8.3	26.6
SN00780571	SC011706	Cc1ccc(C)c(OC2CCN(CC2)c2ncnc3nc[nH]c23)c1	8.2	12.5	not tested	not tested	8.2	12.5
SN00799810	CL7439	O=C(NCc1cccs1)c1cc2CCCN(C)c2s1	8.2	0.0	not tested	not tested	8.2	0.0
SN00781149	SC013375	CNC(=O)Cc1noc(n1)c1cccc(n1)n1cccn1	8.1	4.9	not tested	not tested	8.1	4.9
SN00777957	SC007717	O=C(CN1CCCN(CC1)Cc1nnc(o1)c1c(C)onc1c1ccccc1)NC(C)(C)C	8.1	2.0	not tested	not tested	8.1	2.0
SN00786922	CL9243	Brc1cccc(c1)C(=O)N1CCC(CC1)c1nc2ccccc2[nH]1	8.1	0.0	not tested	not tested	8.1	0.0
SN00807762	CL1135B	COc1ccccc1CNC(=O)CCN1C(=O)c2ccccc2C1=O	8.1	30.7	not tested	not tested	8.1	30.7
SN00802004	CM1047	COCCn1c(nc2cccnc12)C1CCN(C1)S(=O)(=O)c1cccs1	8.0	5.3	not tested	not tested	8.0	5.3

Compound ID	Scaffold	Smiles	% Inhibition n=1		% Inhibition n=2		Average % Inhibition	
			24h	48h	24h	48h	24h	48h
SN00776140	SC003076	COc1cc(ccc1OC)c1csc(n1)n1nccc1C(F)(F)F	8.0	27.9	not tested	not tested	8.0	27.9
SN00777400	SC007239	O=C(N1CCN(CC1)C(=O)c1ccccc1)c1ccc2noc(c3ccccc3)c2c1	8.0	15.8	not tested	not tested	8.0	15.8
SN00790174	CL7867	O=C(NCC1CCCO1)C1CCN(CC1)c1ccc(=O)n(n1)c1ccc(C)c(Cl)c1	8.0	0.0	not tested	not tested	8.0	0.0
SN00777034	SC006675	NC(=Nc1scc(n1)c1ccc2c(c1)CC(=O)N2C)N	8.0	0.0	not tested	not tested	8.0	0.0
SN00791816	CL4597	O=C(NCCc1nc2ccccc2n1C)c1n[nH]c2c1COc1ccccc21	7.9	0.0	not tested	not tested	7.9	0.0
SN00792874	CL5227A	O=C(NCCCN1CCc2ccccc2C1)c1cc2cc(ccc2n1C)S(=O)(=O)N(C)C	7.9	0.0	not tested	not tested	7.9	0.0
SN00801793	CM1696	COc1ccc(cc1)N1CC(CC1=O)c1noc(n1)c1ccc(cc1)C(C)(C)C	7.9	0.0	not tested	not tested	7.9	0.0
SN00800160	CL5534	COc1cccc(c1)N1CCN(CC1)C(=O)C1CCN(CC1)c1snc(NC(=O)C)n1	7.9	16.2	not tested	not tested	7.9	16.2
SN00797413	CL9352	Cc1ccc(cc1)N1CC(CC1=O)C(=O)N1CCCC1c1onc(n1)c1cccc(C)c1	7.8	0.0	not tested	not tested	7.8	0.0
SN00770781	SC006546	NC/C(=C/c1cc(C)n(c1C)c1ccnn1C(C)C)/c1scc(C)n1	7.8	5.0	not tested	not tested	7.8	5.0
SN00783639	CL2385	O=C(NC(C)c1ccccc1)Cn1nc(c2ccccc2)c2ccccc2c1=O	7.8	3.0	not tested	not tested	7.8	3.0
SN00796526	CL8582	Clc1ccc(cc1)N1CCN(CC1)C(=O)c1csc(n1)c1ccc(C)s1	7.8	7.8	not tested	not tested	7.8	7.8
SN00796247	CL9066A	Cc1ccc(cc1)n1nc(C)c2sc(nc12)N1CCCC(C1)C(=O)NCc1ccc2OCOc2c1	7.8	0.0	not tested	not tested	7.8	0.0
SN00769722	SC000926	O=C(N1CCN(CC1)Cc1cccs1)C1CCCN(C1)c1cnccn1	7.7	17.2	not tested	not tested	7.7	17.2
SN00783147	SC012705	CCc1nnc(o1)C1Cc2ccccc2CN1CC(=C)C	7.7	0.0	not tested	not tested	7.7	0.0
SN00790589	CL8391	Brc1ccc(cc1)c1noc(n1)Cn1ccn2nc(cc2c1=O)c1cccc2ccccc12	7.7	10.0	not tested	not tested	7.7	10.0
SN00779777	SC010366	O=C(c1ccc2nncn2c1)N1CCc2[nH]nc(c2C1)C(F)(F)F	7.7	3.6	not tested	not tested	7.7	3.6
SN00790442	CL8366	Fc1ccc(cc1)S(=O)(=O)N1CCN=C1N1CCCCC1	7.7	15.7	not tested	not tested	7.7	15.7
SN00789027	CL7690	Cc1ccc(cc1)S(=O)(=O)n1ccn(C(=O)c2ccccc2C)c1=O	7.7	0.0	not tested	not tested	7.7	0.0
SN00793051	CL2023A	CCc1ccc(cc1)C1=Nc2ncnn2/C/1=N/Cc1ccc(F)cc1	7.7	1.3	not tested	not tested	7.7	1.3
SN00783577	CL2183	CC1=NN(C(=O)COc2ccc(C)c(C)c2)C(O)(C1)C(F)(F)F	7.6	0.0	not tested	not tested	7.6	0.0
SN00801545	CM2931	CN1CCC(CC1)OC1CCN(CC1)C(=O)CC1CCCC1	7.6	26.4	not tested	not tested	7.6	26.4
SN00799120	CL9766	CCC(C)NC(=O)CC1Sc2ncccc2N(C)C1=O	7.6	11.7	not tested	not tested	7.6	11.7
SN00799441	CL9679	O=C(Cn1nc2cc(nc(C)n2c1=O)c1ccccn1)N1CCC(CC1)Cc1ccccc1	7.6	4.9	not tested	not tested	7.6	4.9
SN00775640	SC002186	CC(C)C(C)NC(=O)C1CCN(CC1)c1n(C)nc(C)c1N(=O)=O	7.6	7.0	not tested	not tested	7.6	7.0
SN00787460	CL3343	Fc1ccc(cc1)c1noc(c1)C(=O)Nc1cc(Cl)ccc1O	7.5	3.6	not tested	not tested	7.5	3.6
SN00786280	CL6277	O=C(Nc1ccc(cc1)S(=O)(=O)N1CCCCC1C)c1noc(c1)c1cccs1	7.5	10.1	not tested	not tested	7.5	10.1
SN00783847	CL1789	CCOC(=O)c1c(NC(=O)CN2C(=O)COc3ccc(Cl)cc23)sc2CCCCc12	7.4	26.0	not tested	not tested	7.4	26.0
SN00785845	CL5371	Cc1nc2nncn2c(c1)N1CCN(CC1)c1ccccc1F	7.4	30.4	not tested	not tested	7.4	30.4
SN00793836	CL8307	CCc1noc(c1)C1CCCN1C(=O)Cc1ccccc1F	7.4	10.7	not tested	not tested	7.4	10.7
SN00784268	CL2153	CCOC(=O)N1CCN(CC1)c1nc(cc(n1)C(F)(F)F)c1cccs1	7.3	21.6	not tested	not tested	7.3	21.6
SN00776836	SC006267	Brc1ccc(cc1)Cn1c(nc2c1c(=O)[nH]c(=O)n2C)N1CCNCC1	7.3	0.0	not tested	not tested	7.3	0.0
SN00782395	SC007741	CCn1ncc(CN2CCCN(CC2)C(=O)C(C)n2cncn2)c1	7.3	0.0	not tested	not tested	7.3	0.0
SN00782152	SC015980	COc1ccc(cc1)C(NC(=O)CN1CCN(CC1)Cc1n[nH]c(C)n1)C(C)C	7.3	0.6	not tested	not tested	7.3	0.6
SN00787284	CL3682	Fc1ccc(cc1)CNC(=O)c1cc2c(nc3ccccn3c2=O)n1C	7.2	0.0	not tested	not tested	7.2	0.0
SN00798182	CL9523	CCc1ccc(cc1)CNC(=O)c1cc2ccncc2n1C	7.2	20.1	not tested	not tested	7.2	20.1
SN00791699	CL6877	CCc1ccc(cc1)CNC(=O)c1c[nH]nc1c1ccc(Cl)cc1	7.2	7.3	not tested	not tested	7.2	7.3
SN00772456	SC012627	O=N(=O)c1cc(ccc1N1CCC(CC1)c1nnc2CCCn12)S(=O)(=O)NC(C)(C)C	7.2	4.6	not tested	not tested	7.2	4.6
SN00784759	CL5454	Cc1ccc(cc1)S(=O)(=O)Nc1cc2c(cc1NCc1ccccc1)n(C)c(=O)n2C	7.2	14.3	not tested	not tested	7.2	14.3
SN00793908	CL8308B	CCCOc1ccc(cc1)CN1CCCC1c1c(C)noc1C	7.2	16.7	not tested	not tested	7.2	16.7
SN00777576	SC007509	O=C(Nc1n(C)nc2CCCc12)c1cccc(c1)NC(=O)C(C)(C)C	7.1	0.0	not tested	not tested	7.1	0.0
SN00795395	CL8280A	Cc1ccc(cc1)S(=O)(=O)N1CCN(C)c2ccccc12	7.1	8.6	not tested	not tested	7.1	8.6
SN00785473	CL3589	COc1ccc(cc1)S(=O)(=O)N1CCC(=CC1)C(=O)Nc1ccc(Cl)cc1	7.1	0.0	not tested	not tested	7.1	0.0
SN00797916	CL9438	COc1ccccc1OCCNC(=O)[CH]n1ccc2c(c(C)nn2c2ccccc2)c1=O	7.1	27.1	not tested	not tested	7.1	27.1
SN00770617	SC004496	OC(COc1ccccc1F)Cn1c(=O)c2ccccc2n2nc(C)cc12	7.1	17.7	not tested	not tested	7.1	17.7
SN00789585	CL7994	COc1ccc(CCn2c(=O)c3ncccc3n(Cc3ccc(C)cc3)c2=O)cc1OC	7.0	1.7	not tested	not tested	7.0	1.7
SN00774002	SC000746	Cc1ccc(cc1)C1(C)NC(=O)N(Cc2ncc(o2)c2ccccc2)C1=O	7.0	0.0	not tested	not tested	7.0	0.0

Compound ID	Scaffold	Smiles	% Inhibition n=1		% Inhibition n=2		Average % Inhibition	
			24h	48h	24h	48h	24h	48h
SN00797837	CL9388	COc1ccc(cc1)CNC(=O)c1cc2c(ccn(Cc3ccccc3)c2=O)s1	7.0	0.0	not tested	not tested	7.0	0.0
SN00786959	CL7525	COc1ccccc1OC(C)C(=O)N1N=C2CCCCC2C1(O)C(F)(F)F	7.0	13.1	not tested	not tested	7.0	13.1
SN00797041	CL9040	Cc1ccc(cc1)S(=O)(=O)N(C)c1ccsc1C(=O)Nc1ccc(C)c(C)c1	7.0	12.0	not tested	not tested	7.0	12.0
SN00786012	CL6860	CCOc1cc(ccc1O)C1OCCn2c1c1n(C)c(=O)n(C)c(=O)c1c2c1ccccc1	7.0	8.5	not tested	not tested	7.0	8.5
SN00802205	CM1646	O=C1CCc2c(C)nc(nc2N1CCC1=CCCCC1)c1ccncc1	7.0	0.0	not tested	not tested	7.0	0.0
SN00779739	SC010301	COc1cc(cc(OC)c1OC)c1noc(n1)c1[nH]nc(c1)C1CC1	7.0	13.7	not tested	not tested	7.0	13.7
SN00801332	CM3034	COCC1CN(CC21CCOCC2)C(=O)c1ccc(OC)cc1	6.9	24.3	not tested	not tested	6.9	24.3
SN00781078	SC012973	CCCCC(=O)N1CCN(CC1)S(=O)(=O)c1c[nH]c2ncccc12	6.9	2.4	not tested	not tested	6.9	2.4
SN00784186	CL5991	CCCCN(CCCNC(=O)c1c(C)oc2nc[nH]c(=O)c12)Cc1ccccc1	6.9	16.8	not tested	not tested	6.9	16.8
SN00788633	CL7919	O=C1N=C(NC(C1)C(=O)Nc1ccc(cc1)C(=O)C)N1CCN(CC1)Cc1ccc2OCOc2c1	6.9	6.8	not tested	not tested	6.9	6.8
SN00790783	CL7515	O=C(N1CCN(CC1)S(=O)(=O)c1c(C)nn(C(C)C)c1C)c1cccs1	6.8	3.8	not tested	not tested	6.8	3.8
SN00795482	CL7521	O=C(NC1CC1)c1cnn2c(ccnc12)c1ccccn1	6.8	0.0	not tested	not tested	6.8	0.0
SN00773666	SC000202	C=CCn1c(SCC(=O)N2CCCCC2)nc2scc(c3ccco3)c2c1=O	6.8	5.6	not tested	not tested	6.8	5.6
SN00801154	CM2946	Cc1noc(COC2CCCN(C2)C(=O)c2nn(C)c3ccccc23)n1	6.8	0.0	not tested	not tested	6.8	0.0
SN00785201	CL3462	O=C(CN1CCN(CC1)c1ccc(cc1N(=O)=O)C(F)(F)F)c1ccc2c(CCN2C(=O)C)c1	6.8	3.8	not tested	not tested	6.8	3.8
SN00801692	CM1468	CCc1ccc(cc1)C(=O)N1CCC(CC1)Cc1onc(n1)c1ccc(OC)cc1	6.8	0.0	not tested	not tested	6.8	0.0
SN00791377	CL6597	Fc1ccc(c(F)c1)N1C(=O)CSc2c(c(C)nn2c2ccccc2)C1C(=O)NC1CCCC1	6.8	1.8	not tested	not tested	6.8	1.8
SN00782685	SC015547	CCCCS(=O)(=O)N1CCC2(CC1)CC2c1onc(n1)c1cccc(Cl)c1	6.8	0.0	not tested	not tested	6.8	0.0
SN00775639	SC002186	Cc1cccc(n1)NC(=O)C1CCCN(C1)c1n(C)nc(C)c1N(=O)=O	6.8	0.0	not tested	not tested	6.8	0.0
SN00785483	CL3749	O=C(NCCc1ccccc1)C1CCCN(C1)C(=O)c1cc2sccc2n1C	6.8	4.8	not tested	not tested	6.8	4.8
SN00795110	CL3358	CCOc1ccc(cc1)N/C(=N\S(=O)(=O)C)/Nc1cc(C)c(Cl)cc1OC	6.7	18.1	not tested	not tested	6.7	18.1
SN00790958	CL8429	Cc1ccc(cc1)c1ncc(C(=O)NC2CCCCCC2)c(C)n1	6.7	0.0	not tested	not tested	6.7	0.0
SN00799155	CL9812	Clc1cccc(c1)N1CCN(CC1)C(=O)[CH]n1c(=O)c2nncn2c2ncccc12	6.7	17.9	not tested	not tested	6.7	17.9
SN00772050	SC010703	Cc1ccc(nn1)N1CCCN(CC1)C(=O)CN(C)C1CC1	6.7	11.9	not tested	not tested	6.7	11.9
SN00775259	SC001221	NC(=O)CC1CCN(CC1)C(=O)c1nn2c(C)cc(C)nc2n1	6.7	0.0	not tested	not tested	6.7	0.0
SN00780182	SC011001	COc1ccc(OC)cc1C1N(c2noc(C)c2)C(=O)C(=C1C(=O)c1ccco1)O	6.7	21.6	not tested	not tested	6.7	21.6
SN00784502	CL6291	Oc1cccc(c1)NC(=O)c1noc(c1)c1cccnc1	6.6	24.5	not tested	not tested	6.6	24.5
SN00771233	SC007889	Cc1nn2c(c1)nc(Sc1nnnn1CC1CCCO1)c1ccccc21	6.6	16.5	not tested	not tested	6.6	16.5
SN00778239	SC007966	CCC1N(CCc2sccc12)C(=O)c1[nH]cc(c1)c1csc(C)n1	6.6	3.8	not tested	not tested	6.6	3.8
SN00785300	CL7467	CCc1noc2nc(C)nc(N3CCCC(C3)C(=O)O)c12	6.6	0.0	not tested	not tested	6.6	0.0
SN00789545	CL7310	COc1ccc(NC(=O)C2CCn3c(C2)nc2ccccc32)cc1Cl	6.5	0.0	not tested	not tested	6.5	0.0
SN00798744	CL7308	O=C(Nc1ccccc1)c1cnc(nc1N)N1CCN(CC1)C(=O)c1ccco1	6.5	0.0	not tested	not tested	6.5	0.0
SN00786601	CL9232	O=C(Cc1scnc1c1cccs1)N1CCN(CC1)c1ccccn1	6.5	0.0	not tested	not tested	6.5	0.0
SN00801526	CM2918	CCC(=O)N(C1CCN(CC1)Cc1ccccc1)C1CCOCC1	6.5	3.6	not tested	not tested	6.5	3.6
SN00772032	SC010703	O=C(COc1ccccc1)N1CCCN(CC1)c1cccnn1	6.5	1.9	not tested	not tested	6.5	1.9
SN00799624	CM0164	Cc1csc(n1)c1ccc(cc1)CNC(=O)c1n[nH]c2ccccc12	6.5	25.2	not tested	not tested	6.5	25.2
SN00778763	SC008738	CN(C)C(=O)CN1CCCN(CC1)Cc1ncc(o1)C(C)(C)C	6.5	1.9	not tested	not tested	6.5	1.9
SN00797487	CL9352B	Cc1cccc(c1)c1noc(n1)C1CCCN1S(=O)(=O)c1ccc(cc1)C(C)(C)C	6.5	0.0	not tested	not tested	6.5	0.0
SN00799070	CL9740B	Fc1ccc(cc1)c1ncc2CN(CCc2n1)S(=O)(=O)c1cccc2cccnc12	6.5	3.1	not tested	not tested	6.5	3.1
SN00783317	CL7474	Clc1ccc(cc1)C1NC(=O)NC(=C1C(=O)C)C	6.4	2.8	not tested	not tested	6.4	2.8
SN00801211	CM2676	Fc1cccc(c1)C(=O)N1CC[C@@](C)(O)[C@@H](C1)N1CCCC1	6.4	0.0	not tested	not tested	6.4	0.0
SN00785320	CL3417	COc1ccc(cc1OC)S(=O)(=O)n1nc(cc1N)c1cc(OC)c(OC)c(OC)c1	6.4	21.7	not tested	not tested	6.4	21.7
SN00799224	CL9898A	Fc1cccc(COCC2CCN(CC2)C(=O)C2(CC2)c2ccccc2)c1	6.4	0.0	not tested	not tested	6.4	0.0
SN00802246	CM1830	CCNC(=O)c1c(C)oc2CCNCc12	6.4	10.3	not tested	not tested	6.4	10.3
SN00777586	SC007509	COC(=O)c1cc(cc(c1)N(=O)=O)C(=O)Nc1n(C)nc2CCCc12	6.4	12.1	not tested	not tested	6.4	12.1
SN00773273	SC015439	O=C(NC(C1CC1)c1ccccc1)c1ccc2n3ncnc3NS(=O)(=O)c2c1	6.4	0.0	not tested	not tested	6.4	0.0
SN00802787	CL6428	O=C1NCCCCC1NC(=O)c1cc(nc2cc(nn12)C(C)(C)C)c1ccccc1	6.4	0.0	not tested	not tested	6.4	0.0

Compound ID	Scaffold	Smiles	% Inhibition n=1		% Inhibition n=2		Average % Inhibition	
			24h	48h	24h	48h	24h	48h
SN00789241	CL6673A	COc1ccc2c(c1)NC1(CCN(CC1)C(=O)Cc1ccc(F)cc1)c1cccn21	6.4	5.9	not tested	not tested	6.4	5.9
SN00778442	SC008196	c1cnc(nc1)N1CCCN(CC1)c1nc2ccccc2o1	6.3	32.5	not tested	not tested	6.3	32.5
SN00797738	CL8156	COc1ccc2cc(c3csc(Cc4onc(n4)c4ccccn4)n3)c(=O)oc2c1	6.3	2.5	not tested	not tested	6.3	2.5
SN00794269	CL7291	COc1ccc(cc1)C(=O)Nc1sc(NC)nc1C(=O)N	6.3	25.8	not tested	not tested	6.3	25.8
SN00791042	CL8456	Cc1c(oc2ccc(cc12)S(=O)(=O)N1CCOCC1)c1onc(n1)c1cccnc1	6.3	1.1	not tested	not tested	6.3	1.1
SN00793348	CL2572	O=C(C1CCC1)N1CCCc2cc(ccc12)S(=O)(=O)Nc1ccccc1	6.3	0.0	not tested	not tested	6.3	0.0
SN00774751	SC000973	COc1cc(ccc1OC)S(=O)(=O)N1CCN(CC1)C(=O)c1cc(C)n(C(C)C)c1C	6.2	14.4	not tested	not tested	6.2	14.4
SN00794983	CL5601	Cc1ccc(cc1)NC(=O)c1nn2c(n1)c(=O)n(Cc1ccccc1C)c1cc(C)ccc21	6.2	3.1	not tested	not tested	6.2	3.1
SN00783666	CL2139	OC(=O)c1cccc(Nc2nc(Nc3ccccc3)nc(n2)N2CCCC2)c1	6.2	0.3	not tested	not tested	6.2	0.3
SN00790989	CL8494	COc1ccc(cc1)CCC(=O)N1CCc2cc(ccc12)c1noc(CC)n1	6.2	14.1	not tested	not tested	6.2	14.1
SN00797676	CL6113	Cc1ccc(cc1)c1noc(n1)c1cccc(c1)S(=O)(=O)N1CCN(CC1)c1ccc(F)cc1	6.2	0.0	not tested	not tested	6.2	0.0
SN00788187	CL3699	COc1cccc(c1)Nc1nc(nc2n(C)ncc12)N1CCN(CC1)c1ccccc1	6.1	5.8	not tested	not tested	6.1	5.8
SN00775834	SC002899	Clc1ccc(s1)C(=O)Cn1nnc(n1)c1cccs1	6.1	2.4	not tested	not tested	6.1	2.4
SN00774985	SC001031	O=C(C1CCCN(C1)S(=O)(=O)c1ccccc1)N1CCCC(C1)C(F)(F)F	6.1	7.6	not tested	not tested	6.1	7.6
SN00795513	CL9139	COCCNC(=O)C1(CCC1)c1ccc(cc1)NC(=O)c1ccc(Cl)cc1	6.1	0.0	not tested	not tested	6.1	0.0
SN00783267	SC015099	CC(N1CCc2cncnc2C1)c1nnc(o1)c1cccs1	6.1	12.9	not tested	not tested	6.1	12.9
SN00800411	CM1209	O=C(NC1CCN(C1)c1[nH]nc(c1)c1cccs1)c1ccc2[nH]cnc2c1	6.0	29.6	not tested	not tested	6.0	29.6
SN00799325	CL9946	Fc1ccc(cc1)N1CCN(CC1)S(=O)(=O)c1c(nc2sc(C)cn12)C(C)C	6.0	19.4	not tested	not tested	6.0	19.4
SN00783817	CL4751	COc1ccc(CCNC(=O)c2cnn3c(cc(nc23)c2ccccc2)C(F)F)cc1	6.0	0.0	not tested	not tested	6.0	0.0
SN00773952	SC000734	Brc1ccc(o1)c1nnc(o1)Cn1ccccc1=O	6.0	8.9	not tested	not tested	6.0	8.9
SN00774568	SC000927	Clc1cccc(CN2CCN(CC2)C(=O)c2ccc3[nH]cnc3c2)c1	6.0	0.0	not tested	not tested	6.0	0.0
SN00785272	CL3385	Cc1ccc(cc1)c1[nH]c(nc1S(=O)(=O)CC(=O)Nc1ccccc1)c1ccccc1	6.0	11.7	not tested	not tested	6.0	11.7
SN00773497	SC016401	CCCn1nc2CCN(Cc3onc(n3)c3ccco3)Cc2cc1=O	5.9	0.0	not tested	not tested	5.9	0.0
SN00792412	CL7194	O=C(CN1C(=O)CCn2nc(cc12)c1cn(C)c2ccccc12)N1CCC2(OCCO2)CC1	5.9	10.8	not tested	not tested	5.9	10.8
SN00771230	SC007889	C1COC(C1)Cn1nnnc1Sc1nc(nc2ccccc12)c1cccnc1	5.9	11.8	not tested	not tested	5.9	11.8
SN00783691	CL1666	Cc1ccc(o1)c1nc2ccccc2c(c1)C(=O)N1CC(C)CC(C)C1	5.9	7.2	not tested	not tested	5.9	7.2
SN00807889	SC015171	O=C(c1cnc(s1)c1ccccc1)N1CCn2c(nnc2C)C1	5.8	22.7	not tested	not tested	5.8	22.7
SN00782268	SC016324	NCC1(CCC1)C(=O)N1CC(C1)S(=O)(=O)N1CCN(CC1)C(=O)OC(C)(C)C	5.8	1.1	not tested	not tested	5.8	1.1
SN00789817	CL7744	COCCNC(=O)C1(C)Cn2c(cc3ccsc23)C(=O)N1c1ccc(OC)c(OC)c1	5.8	0.0	not tested	not tested	5.8	0.0
SN00796570	CL8836	Cc1ccc(cc1)CNC(=O)c1ccc(cc1)n1cnc(NC(=O)C(C)C)c1	5.8	29.7	not tested	not tested	5.8	29.7
SN00779257	SC009478	Cn1c2nc3oc(cn3c2c(=O)n(CC(=O)C(C)(C)C)c1=O)C(C)(C)C	5.8	18.6	not tested	not tested	5.8	18.6
SN00798241	CL9551	O=C(Nc1ccccc1)N1CCC(CC1)c1sc(c(C)n1)C(=O)N1CCCCCC1	5.8	0.0	not tested	not tested	5.8	0.0
SN00792660	CL6390	Cc1ccc(NS(=O)(=O)c2nc(C)n(C)c2)c(Br)c1	5.7	25.8	not tested	not tested	5.7	25.8
SN00795279	CL8091	COc1ccc(cc1)c1c(nn2c(C)cc(=O)[nH]c12)C(=O)Nc1cccc(Cl)c1	5.7	7.0	not tested	not tested	5.7	7.0
SN00774438	SC000898	CC(=O)NC1CCCN(C1)C(=O)c1cc2c(C)nn(Cc3ccc(F)cc3)c2s1	5.7	0.0	not tested	not tested	5.7	0.0
SN00784121	CL1652	Clc1ccc(cc1)c1nc2cc(nn2c(c1)C(F)(F)F)C(=O)N(C)CCc1ccccn1	5.7	0.0	not tested	not tested	5.7	0.0
SN00792679	CL6292	CC1Cc2ccccc2N1C(=O)c1n[nH]c(c1)c1cccnc1	5.6	19.0	not tested	not tested	5.6	19.0
SN00781689	SC014777	COCC(=O)Nc1ccc(cc1)NC(=O)c1cc2nc(C)ccc2o1	5.6	14.1	not tested	not tested	5.6	14.1
SN00777618	SC007544	O=C(Nc1ccc(cc1)N(=O)=O)CN1C(=O)CC2(CCCC2)C1=O	5.5	47.2	not tested	not tested	5.5	47.2
SN00781466	SC014207	O=C(NC1CCCc2n(C)ncc12)N1CCCCC(C1)N1CCCC1	5.5	13.9	not tested	not tested	5.5	13.9
SN00807846	SC012373	O=C(CSc1ccccc1)NCC1(CCOCC1)N1CCCCC1	5.4	1.8	not tested	not tested	5.4	1.8
SN00802783	CL6284	CCn1nc(cc1c1cc(C)sc1C)C(=O)NCCOc1ccccc1	5.4	0.0	not tested	not tested	5.4	0.0
SN00771924	SC009489	O=C(N1CCCC(C1)c1nnc2CCCCCn12)C(c1ccccc1)c1ccccc1	5.4	8.5	not tested	not tested	5.4	8.5
SN00778393	SC008075	O=C(CN1C(=O)NC2(CCc3ccccc23)C1=O)N1CCCC(C1)c1nnc2ccccn12	5.4	0.0	not tested	not tested	5.4	0.0
SN00788236	CL4805B	O=C(NCCCc1ccccc1)c1nn2CC(C)(C(=O)NC3CCCC3)N(C)C(=O)c2c1	5.4	0.6	not tested	not tested	5.4	0.6
SN00786431	CL8245	Cc1nc(cc(n1)N1CCCCC1)N1CCN(CC1)C(=O)c1cccc(F)c1	5.4	7.0	not tested	not tested	5.4	7.0

Compound ID	Scaffold	Smiles	% Inhibition n=1		% Inhibition n=2		Average % Inhibition	
			24h	48h	24h	48h	24h	48h
SN00789262	CL7759	CCc1nnc(o1)c1cc2ccccc2n1CC(=O)N1CCCc2ccccc12	5.4	0.0	not tested	not tested	5.4	0.0
SN00779979	SC010660	O=c1n(cnc2sccc12)Cc1noc(n1)c1ccsc1	5.3	0.0	not tested	not tested	5.3	0.0
SN00792328	CL6845	Fc1ccc(cc1)CN1CCCn2nc(cc2C1=O)C(=O)NCc1ccccc1Cl	5.3	0.0	not tested	not tested	5.3	0.0
SN00771379	SC008231	COc1ccc(cc1)CN1CCCN(CC1)C(=O)c1nc2cc(C)ccn2c1	5.3	0.0	not tested	not tested	5.3	0.0
SN00796250	CL9066A	Cc1ccc(cc1)n1nc(C)c2sc(nc12)N1CCCC(C1)C(=O)NCC1CCCO1	5.3	2.9	not tested	not tested	5.3	2.9
SN00790077	CL7825A	CCn1c(=O)c(nc2ccccc12)N1CCCC(C1)C(=O)NCCc1c[nH]c2ccccc12	5.3	9.8	not tested	not tested	5.3	9.8
SN00790111	CL8279	O=C(Cc1cccs1)Nc1ccc(cc1C(=O)O)N1CCN(CC1)c1ccccc1F	5.3	0.0	not tested	not tested	5.3	0.0
SN00785926	CL5587	O=C1O[C@@H]2C[C@H]3C[C@@H]2[C@@H]1[C@H]3C(=O)Nc1ccc(cc1)C(=O)N1CCC(=CC1)c1ccccc1	5.3	11.8	not tested	not tested	5.3	11.8
SN00790031	CL8110	CCc1ccc(cc1)c1n[nH]c(c1)C(=O)N1CCN(CC1)c1c(C)n(c2ccccc2)c(=O)n(C)c1=O	5.3	0.0	not tested	not tested	5.3	0.0
SN00775947	SC002957	O=C(Nc1ccnn1Cc1ccccn1)c1cccnc1Oc1ccccc1	5.2	2.6	not tested	not tested	5.2	2.6
SN00778718	SC008707	Cc1ccc(cc1)c1nc(C)sc1CC(=O)N1CCN(CC1)Cc1nc(N)c2ccccc2n1	5.2	9.0	not tested	not tested	5.2	9.0
SN00784460	CL2028	CCCNC(=O)c1noc(c1)c1ccc(F)cc1	5.2	0.0	not tested	not tested	5.2	0.0
SN00784503	CL2031	Clc1ccc(cc1)CNC(=O)c1noc2CCCCc12	5.1	0.0	not tested	not tested	5.1	0.0
SN00799885	CM0272	CCOc1ccc2nc(cn2n1)C(=O)N1CCN(CC1)c1cccc(C)c1C	5.1	9.3	not tested	not tested	5.1	9.3
SN00780701	SC011877	Cc1ccc(C)c(c1)NC(=O)N1CCC(CC1)c1c[nH]c2ncccc12	5.1	0.0	not tested	not tested	5.1	0.0
SN00779507	SC010119	Fc1ccc(cc1)C(C)NC(=O)Cc1csc(n1)Cc1ccccn1	5.1	0.0	not tested	not tested	5.1	0.0
SN00784068	CL1193	COc1cc(CNCCNC(=O)c2nonc2N)ccc1OCc1ccc(F)cc1	5.1	0.0	not tested	not tested	5.1	0.0
SN00802927	CL0948	CCn1cc(C(=O)Nc2ccccc2O)c(=O)c2cc(F)c(cc12)N1CCN(CC1)C(=O)C	5.1	0.0	not tested	not tested	5.1	0.0
SN00800991	CM2380A	CSc1ccc(cc1)NC(=O)N1C[C@@H]2C[C@H]1C[C@@H]2NC(=O)c1cccs1	5.1	17.8	not tested	not tested	5.1	17.8
SN00773389	SC015832	O=C(NCc1ccco1)N1CC2C3CCC(C3)C12	5.1	2.6	not tested	not tested	5.1	2.6
SN00771716	SC008911	COC(=O)CNC(=O)c1nnn(c1)c1ccc(cn1)C(F)(F)F	5.0	0.0	not tested	not tested	5.0	0.0
SN00778747	SC008719	c1ccc(cc1)c1noc(CN2CCOC(Cn3cncn3)C2)c1	5.0	6.8	not tested	not tested	5.0	6.8
SN00783803	CL0341	COc1cccc(c1)[C@@H]1Nc2c(cnn2[C@@H](C1)C(F)(F)F)C(=O)N1CCCCC1	5.0	0.0	not tested	not tested	5.0	0.0
SN00794609	CL5141	CC1CCN(CCCNC(=O)c2ccc(c(NC(=O)C)c2)S(=O)(=O)c2ccc(Cl)cc2)CC1	5.0	23.9	not tested	not tested	5.0	23.9
SN00801093	CM3131	COc1nccnc1N1CCC(C1)NC(=O)c1ccnn1CC	5.0	0.0	not tested	not tested	5.0	0.0
SN00788935	CL4722	Cc1ccsc1CCNC(=O)c1cnn2c1n(C)c1CCN(Cc3ccccc3)Cc1c2=O	5.0	17.0	not tested	not tested	5.0	17.0
SN00796091	CL8537	CCCNC(=O)N1CCN(CC1)Cc1nc2cccnc2n1c1ccc(CC)cc1	5.0	0.0	not tested	not tested	5.0	0.0
SN00783193	SC013651	CCC(C)(NCc1ncc(o1)c1cccs1)c1nccs1	5.0	26.6	not tested	not tested	5.0	26.6
SN00779481	SC010036	O=c1[nH]c(C)c(s1)S(=O)(=O)N1CCN(CC1)c1ccccc1N(=O)=O	4.9	0.0	not tested	not tested	4.9	0.0
SN00772712	SC013592	O=c1onc(c2cccnc2)n1Cc1noc(n1)C1CC1	4.9	0.0	not tested	not tested	4.9	0.0
SN00775605	SC002131	O=c1[nH]c(CN2CCCCC2)nc2scc(c3cccs3)c12	4.9	0.0	not tested	not tested	4.9	0.0
SN00795412	CL8921	O=C(Nc1cc(F)c2[nH]c(=O)ccc2c1)Nc1cccc(C)c1C	4.9	0.0	not tested	not tested	4.9	0.0
SN00783226	SC014139	O=C(N1CCC(C1)N1CCCC1)c1cccc(c1)NC(=O)c1ccco1	4.9	0.0	not tested	not tested	4.9	0.0
SN00798786	CL9687	Cc1cc(C)c(cc1C)S(=O)(=O)N1CCc2[nH]cnc2C1	4.9	0.0	not tested	not tested	4.9	0.0
SN00794110	CL3544A	Brc1ccc(cc1)NC(=O)Cn1c(=O)c2c(C)cc(C)nc2n(C)c1=O	4.9	0.0	not tested	not tested	4.9	0.0
SN00773041	SC014895	O=C(NC1CCCCC1)Cc1noc(n1)c1cocc1	4.9	3.1	not tested	not tested	4.9	3.1
SN00787657	CL4851	O=C(N1CCN(CC1)c1ccccc1)[C@H]1C[C@H]2CCCN2[C@]21C(=O)Nc1ccccc21	4.9	16.0	not tested	not tested	4.9	16.0
SN00777604	SC007512	O=C(N1CC2CCC1C2)c1cc(nc2ccccc12)C1CC1	4.9	18.4	not tested	not tested	4.9	18.4
SN00776412	SC003252	c1ccc(cc1)c1cn2nc(c3ccccc3)n3CCCCc1c23	4.9	0.0	not tested	not tested	4.9	0.0
SN00792717	CL4870	Cc1cccc(c1)C1=NC2(CCN(CC2)C(=O)NC2CCCCC2)NC1=O	4.9	13.8	not tested	not tested	4.9	13.8
SN00780706	SC011877	c1ccc(nc1)CCN1CCC(CC1)c1c[nH]c2ncccc12	4.9	11.5	not tested	not tested	4.9	11.5
SN00791850	CL3311	C=CCNC(=O)CCN1CCN(Cc2ccccc2)C1=O	4.9	0.0	not tested	not tested	4.9	0.0
SN00800180	CL5534	CC(=O)Nc1nsc(n1)N1CCCC(C1)C(=O)NCc1ccccc1Cl	4.9	5.9	not tested	not tested	4.9	5.9
SN00797075	CL8111A	O=C(Nc1ccc(C)c(C)c1)Cn1c(=O)nc(NC2CCCC2)c2ccccc12	4.8	0.0	not tested	not tested	4.8	0.0
SN00798508	CL9568	Fc1ccc(cc1)N1CCN(CC1)C(=O)c1cc2sc(C)c(CN3CCOCC3)c2[nH]1	4.8	27.9	not tested	not tested	4.8	27.9
SN00789162	CL5683	COc1ccc(cc1)n1ccn(CC(=O)Nc2ccc(OC)cc2OC)c1=O	4.8	0.0	not tested	not tested	4.8	0.0

Compound ID	Scaffold	Smiles	% Inhibition n=1		% Inhibition n=2		Average % Inhibition	
			24h	48h	24h	48h	24h	48h
SN00769948	SC001347	O=C(N1CCC(=CC1)c1ccccc1)c1ccc(Cl)c(c1)S(=O)(=O)NCC1CCCO1	4.8	6.8	not tested	not tested	4.8	6.8
SN00801182	CM2967	COc1cccc(c1)C(=O)N(CC1COCC1)C1CC1	4.8	0.0	not tested	not tested	4.8	0.0
SN00801277	CM2392	OC[C@@H]1CCN(Cc2ccc3[nH]ccc3c2)C[C@H]1N(C)C	4.7	6.2	not tested	not tested	4.7	6.2
SN00795351	CL8949	COCCNc1c(nc2sc3cc(C)ccc3n12)c1ccc(O)cc1	4.7	0.0	not tested	not tested	4.7	0.0
SN00794777	CL8480	CCNC(=O)C1CCCN1C(=O)c1cccc(NC(=O)c2ccc(Br)cc2)c1	4.7	0.0	not tested	not tested	4.7	0.0
SN00796120	CL8537A	CCc1ccc(cc1)n1c(CN2CCN(CC2)S(=O)(=O)C)nc2cccnc12	4.7	7.0	not tested	not tested	4.7	7.0
SN00796435	CL9209A	Cc1ccc(COCc2nn(C)c3CCN(Cc23)C(=O)Nc2ccccc2)cc1	4.7	0.0	not tested	not tested	4.7	0.0
SN00779264	SC009635	O=C(c1ccc(=O)n(C)c1)N1CCCC1C1CCCCC1	4.7	13.1	not tested	not tested	4.7	13.1
SN00784704	CL4134	Cc1ccc(NC(=O)CN2CC(CC2=O)c2ccccc2)c(C)c1	4.7	6.2	not tested	not tested	4.7	6.2
SN00775126	SC001100	CSc1ccc(cc1)N1CC(CC1=O)C(=O)N1C(C)Cc2ccccc12	4.7	0.0	not tested	not tested	4.7	0.0
SN00800228	CL8338	CCc1nn(CC)c(=O)c2cc(cn12)S(=O)(=O)N1CCOCC1	4.7	11.8	not tested	not tested	4.7	11.8
SN00788648	CL7412	COc1cc(cc(OC)c1OC)c1c(Nc2ccccc2)oc2c1c(=O)n(C)c(=O)n2C	4.7	0.0	not tested	not tested	4.7	0.0
SN00783261	SC015007	COCCN1C(=O)CCC(C(=O)O)C1c1cnn(c1)c1ccccc1	4.7	0.0	not tested	not tested	4.7	0.0
SN00779194	SC009466	CCCn1c(=O)c2n3CCN(c4ccc(CC)cc4)c3nc2n(C)c1=O	4.6	0.0	not tested	not tested	4.6	0.0
SN00773871	SC000350	Clc1ccc2c(c1)nc(CN1CCN(CC1)C(=O)C)n2C	4.6	0.0	not tested	not tested	4.6	0.0
SN00798231	CL9549	COC(=O)c1ccccc1NC(=O)Nc1ccc2[nH]c3CCN(Cc3c2c1)C(C)C	4.6	4.3	not tested	not tested	4.6	4.3
SN00799900	CM0279	O=C(N1CCN(CC1)c1ccccc1F)c1nc2ccccn2c1	4.6	4.9	not tested	not tested	4.6	4.9
SN00791893	CL5763	O=C(N1CCCCC1)C1Cc2cc(ccc2N1C(=O)C)S(=O)(=O)N1CCCCC1	4.6	0.0	not tested	not tested	4.6	0.0
SN00784765	CL5454	COc1cc(ccc1OC)S(=O)(=O)Nc1cc2c(cc1NCc1ccccc1)n(C)c(=O)n2C	4.6	14.1	not tested	not tested	4.6	14.1
SN00779496	SC010119	O=C(Cc1csc(n1)Cc1ccccn1)Nc1ccc2nc(sc2c1)N1CCOCC1	4.5	2.9	not tested	not tested	4.5	2.9
SN00796396	CL9186	COc1c(OC)cccc1c1noc(n1)c1cc(nc2ccccc12)N1CCN(CC1)C(=O)C	4.5	7.7	not tested	not tested	4.5	7.7
SN00798959	CL9737	CCc1c(N)onc1C1CCN(CC1)C(=O)c1cnccn1	4.4	12.1	not tested	not tested	4.4	12.1
SN00802274	CM1834	CCC1(CCN(C1)S(=O)(=O)c1ccccc1)N1CCN(C)CC1	4.4	2.6	not tested	not tested	4.4	2.6
SN00796298	CL8825	COc1ccc(cc1)CNC(=O)c1c(C)sc2cc(oc12)c1ccccc1	4.4	29.4	not tested	not tested	4.4	29.4
SN00785962	CL5632	CCCCS(=O)(=O)N1CCC(CC1)c1nnnn1c1ccc(C)cc1	4.4	0.0	not tested	not tested	4.4	0.0
SN00781334	SC014110	O=C(Nc1cc(ccc1C)N(=O)=O)N1CCC(CC1)n1cncn1	4.4	0.0	not tested	not tested	4.4	0.0
SN00789651	CL8016	CCCNS(=O)(=O)c1scc(c1)C(=O)N1CCCC1	4.3	0.0	not tested	not tested	4.3	0.0
SN00783298	SC015495	CC1CCN(C(=O)CCn2c(=O)sc(C)c2C)C(C1)c1ccco1	4.3	0.0	not tested	not tested	4.3	0.0
SN00781228	SC013655	FC(F)Oc1ccccc1NC(=O)Cc1csc(n1)N1CCCNC1=O	4.3	8.7	not tested	not tested	4.3	8.7
SN00796395	CL9186	CCOC(=O)N1CCN(CC1)c1nc2ccccc2c(c1)c1onc(n1)c1cccc(OC)c1OC	4.3	18.1	not tested	not tested	4.3	18.1
SN00774466	SC000900	CC(C)CN1CC(CC1=O)C(=O)N1CCC(CC1)Nc1cccnn1	4.3	0.0	not tested	not tested	4.3	0.0
SN00791689	CL4521	COc1ccc(cc1)N(CC)S(=O)(=O)c1csc(c1)C(=O)N(C)C	4.3	2.7	not tested	not tested	4.3	2.7
SN00802538	CL3392	COC(=O)C(C)n1cnc2n(nnc2c1=O)Cc1ccc(C)cc1	4.3	3.2	not tested	not tested	4.3	3.2
SN00785745	CL1071	Brc1ccc(cc1)S(=O)(=O)c1nc(oc1N1CCOCC1)c1ccccc1	4.2	4.0	not tested	not tested	4.2	4.0
SN00789013	CL2349A	CC1CCN(CC1)c1ncnc2c1c(C)c(C)n2c1ccccc1	4.2	35.2	not tested	not tested	4.2	35.2
SN00770081	SC001432	c1ccc(cc1)n1nnnc1N1CCN(CC1)C1CC1	4.2	0.0	not tested	not tested	4.2	0.0
SN00776975	SC006580	COCCCc1scc(n1)c1cc2CCC(=O)Nc2cc1F	4.2	0.0	not tested	not tested	4.2	0.0
SN00802991	CL4666	Cc1cc(C)cc(c1)N1CCN(CCCNC(=O)c2onc(n2)c2ccncn2)CC1	4.2	0.0	not tested	not tested	4.2	0.0
SN00799013	CL9740	Fc1ccc(cc1)c1ncc2CN(CCc2n1)C(=O)c1cccc(Br)c1	4.2	13.8	not tested	not tested	4.2	13.8
SN00791149	CL3586C	CCCN1CCN(CC1)C(=O)C1=C(C)C(=O)OC21CCN(CC2)S(=O)(=O)CC	4.2	0.0	not tested	not tested	4.2	0.0
SN00797785	CL9393	CCCCOc1ccc(cc1)CNC(=O)C1CCN(CC1)c1nccc2sc(C)cc12	4.2	27.9	not tested	not tested	4.2	27.9
SN00783272	SC015103	CCc1ncc2CCN(Cc3nc(oc3C)c3ccco3)Cc2n1	4.2	0.0	not tested	not tested	4.2	0.0
SN00799594	CL5620	Cc1cccc(c1)CN1CCc2c(C1)cc(C(=O)NC(C)(C)C)n2C	4.2	0.0	not tested	not tested	4.2	0.0
SN00783777	CL6990	CC(=O)N1N=C2C3CCN(CC3)[C@@H]2[C@@H]1c1cccc2ccccc12	4.2	0.0	not tested	not tested	4.2	0.0
SN00792862	CL5227A	CCN(CC)S(=O)(=O)c1ccc2c(cc(C(=O)NC3CCCC3)n2C)c1	4.2	0.0	not tested	not tested	4.2	0.0
SN00774966	SC001011	Brc1ccc(cc1)S(=O)(=O)N1CCCC1C(=O)N1CCCCC1	4.1	3.8	not tested	not tested	4.1	3.8

Compound ID	Scaffold	Smiles	% Inhibition n=1		% Inhibition n=2		Average % Inhibition	
			24h	48h	24h	48h	24h	48h
SN00791426	CL6014A	Cc1[nH]c(C)c(c1C(=O)N1CCCCC1)S(=O)(=O)N1CCCc2ccccc12	4.1	0.0	not tested	not tested	4.1	0.0
SN00782563	SC013398	OC(CN1CCCN(CC1)C1CC1)c1ccccc1C(F)(F)F	4.1	5.9	not tested	not tested	4.1	5.9
SN00774178	SC000816	O=C1C(=O)N(C2CCCC2)C(=O)N1Cc1onc(n1)c1ccsc1	4.1	0.0	not tested	not tested	4.1	0.0
SN00800379	CM1205	COc1ccc(cc1)c1n[nH]c(c1)N1CCC(CC1)NC(=O)Cc1ccccc1	4.1	0.0	not tested	not tested	4.1	0.0
SN00779415	SC009945	CSCCC(NS(=O)(=O)c1ccc(C)cc1)C(=O)Nc1nc(C)c(s1)c1ccccn1	4.1	0.0	not tested	not tested	4.1	0.0
SN00795072	CL8587	Fc1ccc(cc1)N1CCN(CC1)C(=O)C1CCN(CC1)c1cc(C)nc2cc(nn12)c1ccccc1	4.1	0.0	not tested	not tested	4.1	0.0
SN00774511	SC000907	CC1CCN(CC1)C(=O)c1n[nH]c(=O)c2ccccc12	4.0	0.0	not tested	not tested	4.0	0.0
SN00770479	SC002933	O=C(N1CCC(CC1)NS(=O)(=O)c1cccs1)c1cc(nc2c1cnn2C(C)C)C1CC1	4.0	0.0	not tested	not tested	4.0	0.0
SN00797440	CL9352A	O=C(NCCc1ccccc1)N1CCCC1c1onc(n1)c1ccccc1	4.0	0.0	not tested	not tested	4.0	0.0
SN00784980	CL2762	O=C(Nc1cccc(c1)C(=O)C)C1CCCN(C1)S(=O)(=O)c1c(C)noc1C	4.0	0.0	not tested	not tested	4.0	0.0
SN00791881	CL5759	COc1ccc(cc1)C1=NCC(=O)N(CC(=O)N(CC)CC)c2sc3CCCCc3c12	4.0	0.0	not tested	not tested	4.0	0.0
SN00787372	CL4805	COc1ccc(cc1)N1C(=O)c2cc(nn2CC1(C)C(=O)NC(C)(C)C)c1ccco1	4.0	16.8	not tested	not tested	4.0	16.8
SN00793909	CL8307B	Cc1noc(c1)C1CCCN1Cc1sccc1C	3.9	2.5	not tested	not tested	3.9	2.5
SN00788730	CL6477	Cc1cccc(c1)N(C)c1ccc2cc(ccc2n1)S(=O)(=O)N1CCCC1	3.9	7.2	not tested	not tested	3.9	7.2
SN00782653	SC014997	Cc1nsc(n1)N1CCCN(CC1)C(=O)NCc1ccccc1C	3.9	0.0	not tested	not tested	3.9	0.0
SN00786137	CL5015B	CCn1c(Cn2cccn2)nc2cc(ccc12)NC(=O)C	3.9	11.6	not tested	not tested	3.9	11.6
SN00786652	CL9247	Fc1ccc(cc1)N1CCN(CC1)C(=O)Cc1c(C)nn(c2scc(n2)c2ccccc2)c1O	3.9	0.0	not tested	not tested	3.9	0.0
SN00789403	CL7811	CCCCn1c(=O)[nH]c2cc(ccc2c1=O)c1onc(n1)c1ccc(F)cc1	3.9	0.3	not tested	not tested	3.9	0.3
SN00798982	CL9739	O=C(Nc1ccccc1F)N1CCC(CC1)c1cc(=O)n2nccc2n1C	3.9	3.2	not tested	not tested	3.9	3.2
SN00800876	CM1544	COc1ccc(cc1)c1[nH]n2c(c1)nc1CCNCc1c2=O	3.9	0.0	not tested	not tested	3.9	0.0
SN00798784	CL9687	O=S(=O)(c1cccs1)N1CCc2[nH]cnc2C1	3.9	0.0	not tested	not tested	3.9	0.0
SN00785721	CL4038	COc1ccc(cc1)C(=O)c1c(N)c(c2scc(n2)c2cc(OC)ccc2OC)c2ccccn12	3.8	0.0	not tested	not tested	3.8	0.0
SN00799344	CL9947	COc1ccc(cc1)CNS(=O)(=O)c1c(C)nc2sc(C)nn12	3.8	4.3	not tested	not tested	3.8	4.3
SN00787436	CL4811	O=C1c2cc3ccccc3n2CC(C)(C(=O)NC2CCCCC2)N1Cc1ccco1	3.8	0.0	not tested	not tested	3.8	0.0
SN00795853	CL9007A	COc1ccc(C)cc1NC(=O)Nc1c([nH]c2c(Cl)cccc12)c1ccncc1	3.8	0.0	not tested	not tested	3.8	0.0
SN00780166	SC010993	S=c1[nH]cccc1C(=O)N1CCCC(CN2CCN(CC2)c2ccccn2)C1	3.8	0.0	not tested	not tested	3.8	0.0
SN00774977	SC001011	Clc1ccc(cc1)c1noc(n1)C1CCCN(C1)C(=O)C1CCCN1C(=O)C1CCCCC1	3.8	0.0	not tested	not tested	3.8	0.0
SN00792278	CL7010A	Brc1cccc(CNC(=O)c2ncoc2c2cccs2)c1	3.8	6.4	not tested	not tested	3.8	6.4
SN00782358	SC006833	Cc1cccc(c1)c1noc(Cn2cccn2)n1	3.8	0.0	not tested	not tested	3.8	0.0
SN00803088	CL6946A	COc1ccc(cc1)CNC(=O)N1CCC(CC1)c1ccn2ncc(C(=O)N3CCOCC3)c2c1	3.8	0.7	not tested	not tested	3.8	0.7
SN00792154	CL4105	O=C(NCCN1CCCCC1)CCc1nc2c(C)nn(c3ccccc3)c2n(C)c1=O	3.7	0.0	not tested	not tested	3.7	0.0
SN00799184	CL9831	CCn1nnc2c(C(=O)NCCC3=CCCCC3)c(C)sc2c1=O	3.7	2.6	not tested	not tested	3.7	2.6
SN00784919	CL3314	CCN1CCN(CC1)C(=O)c1ccc(cc1)N1CCCC1=O	3.7	8.8	not tested	not tested	3.7	8.8
SN00789781	CL8042	Cc1onc(n1)c1cccc(c1)N1CC(CC1=O)C(=O)NCc1ccccc1	3.7	0.0	not tested	not tested	3.7	0.0
SN00790270	CL7460	O=C(Nc1ccc2nc(cc(C(=O)O)c2c1)N1CCCN(CC1)c1ncccn1)c1ccccc1F	3.7	0.0	not tested	not tested	3.7	0.0
SN00776455	SC003494	O=C1C(=C(C(=O)c2ccccc2)C(c2ccncc2)N1CCN1CCOCC1)O	3.7	0.7	not tested	not tested	3.7	0.7
SN00797555	CL4671	COc1cc(ccc1OC)CNS(=O)(=O)c1ccc2onc(C(=O)N3CCCCC3)c2c1	3.7	0.0	not tested	not tested	3.7	0.0
SN00791300	CL6077	COc1ccc(cc1)c1noc(n1)c1[nH]nc(c1)c1cccs1	3.6	15.5	not tested	not tested	3.6	15.5
SN00781301	SC013994	CCCc1noc(n1)Cn1c(=O)onc1c1ccc(OC)c(OC)c1	3.6	0.0	not tested	not tested	3.6	0.0
SN00798240	CL9551	COCCCNC(=O)c1sc(nc1C)C1CCN(CC1)C(=O)NC	3.6	6.4	not tested	not tested	3.6	6.4
SN00773239	SC015371	COc1ccc(cn1)NC(=O)N1CCN(CC1)CC1CCCCN1C	3.6	0.0	not tested	not tested	3.6	0.0
SN00800416	CM1389	CC1CCN(CC1)c1nc(nc2n(C)ncc12)C(=O)NCCc1ccncc1	3.6	9.4	not tested	not tested	3.6	9.4
SN00802442	CM3115	Cc1oc(nc1C(=O)NC(C)c1ccccn1)C1CCC1	3.6	17.0	not tested	not tested	3.6	17.0
SN00795424	CL8921	CCOC(=O)CC(NC(=O)Nc1cc(F)c2[nH]c(=O)ccc2c1)c1ccccc1	3.5	4.9	not tested	not tested	3.5	4.9
SN00796410	CL9209	O=C(C(C)C)N1CCc2n(C)nc(COCc3ccccc3F)c2C1	3.5	0.0	not tested	not tested	3.5	0.0
SN00797364	CL9338	COc1cc(OC)ccc1c1oc(C)c(Cn2nnc(C(=O)Nc3ccc(F)cc3F)c2N)n1	3.5	3.5	not tested	not tested	3.5	3.5

Compound ID	Scaffold	Smiles	% Inhibition n=1		% Inhibition n=2		Average % Inhibition	
			24h	48h	24h	48h	24h	48h
SN00795320	CL8523A	Cc1cccc(c1)C(=O)Nc1cnc(N2CCNCC2)c(c1)C(=O)N(CC(C)C)CC(C)C	3.4	14.5	not tested	not tested	3.4	14.5
SN00776135	SC003076	FC(F)(F)c1ccnn1c1scc(n1)c1ccc(cc1)S(=O)(=O)C	3.4	7.7	not tested	not tested	3.4	7.7
SN00803046	CL6946	O=C(N1CCC(CC1)c1ccn2ncc(C(=O)N3CCCCC3)c2c1)c1ccccc1	3.4	0.0	not tested	not tested	3.4	0.0
SN00774186	SC000816	CCOc1ccccc1N1C(=O)C(=O)N(Cc2onc(C)n2)C1=O	3.4	14.0	not tested	not tested	3.4	14.0
SN00796424	CL9209	COCC(=O)N1CCc2n(C)nc(COCc3ccc(C)cc3)c2C1	3.4	0.0	not tested	not tested	3.4	0.0
SN00798797	CL9705	COc1c(oc2ccc(NC(=O)C3CCCCC3)cc12)C(=O)Nc1ccccc1	3.4	3.8	not tested	not tested	3.4	3.8
SN00796358	CL8891	O=C(NCC1CC1)c1nnn(c2ccccc2)c1c1ccncc1	3.4	0.0	not tested	not tested	3.4	0.0
SN00769595	SC000885	Brc1ccc(s1)S(=O)(=O)N1CCN(CC1)C(=O)c1nc(oc1C)c1ccccc1	3.4	23.5	not tested	not tested	3.4	23.5
SN00798764	CL7308	COc1ccc(cc1)NC(=O)c1cnc(nc1N)N1CCN(CC1)CC(=O)N1CCCC1	3.4	0.0	not tested	not tested	3.4	0.0
SN00783289	SC015404	CS(=O)(=O)c1nccn1Cc1ccccn1	3.3	1.6	not tested	not tested	3.3	1.6
SN00770541	SC003349	CN1CCN(CC1)Cc1nc2ccccc2c(=O)n1Cc1scc(n1)c1cccc(c1)N(=O)=O	3.3	11.4	not tested	not tested	3.3	11.4
SN00787370	CL4805	Fc1ccc(cc1)CNC(=O)C1(C)Cn2nc(cc2C(=O)N1Cc1ccccc1)c1ccco1	3.3	13.6	not tested	not tested	3.3	13.6
SN00798831	CL9717	CC(=O)N1CCc2cc(C)c(cc12)S(=O)(=O)NC1CC1	3.3	4.3	not tested	not tested	3.3	4.3
SN00797379	CL9351	CC1CCC(CC1)NC(=O)c1ccc(cc1)c1noc(n1)C1CCN(CC1)C(=O)c1ccccc1	3.3	0.0	not tested	not tested	3.3	0.0
SN00799189	CL9882	CCCNC(=O)c1cncc(c1)c1cccc(NC(=O)CC)c1	3.3	0.0	not tested	not tested	3.3	0.0
SN00792891	CL6070	Cc1ccc(cc1)CNC(=O)Cn1c(C)cc(C)c(c2onc(n2)c2ccccc2)c1=O	3.2	0.0	not tested	not tested	3.2	0.0
SN00800452	CM1392	CCOC(=O)c1ccc(cc1)NC(=O)c1nc(C)n(C2CCN(CC2)C(=O)C)c1C	3.2	13.2	not tested	not tested	3.2	13.2
SN00784094	CL7511	COC(=O)C1=C(C)N(C(C)C)C(=O)C1(NC(=O)C1CCCCC1)C(F)(F)F	3.2	0.0	not tested	not tested	3.2	0.0
SN00782719	SC000900	O=C(C1CC(=O)N(C1)C1CCCC1)N1CCCCC1Cn1cccn1	3.2	0.0	not tested	not tested	3.2	0.0
SN00784661	CL2503	COc1c(OC)cc(cc1OC)C(=O)N1C2CC(C)(Oc3ccccc23)N(c2ccc(F)cc2)C1=O	3.2	3.1	not tested	not tested	3.2	3.1
SN00785642	CL5700A	CCCc1cc(=O)n2nc(sc2n1)N(C)CC(=O)NCCCN1CCCC1=O	3.2	4.1	not tested	not tested	3.2	4.1
SN00791289	CL6077	COc1c(OC)cc(cc1OC)c1noc(n1)c1[nH]nc(c1)c1ccco1	3.2	2.3	not tested	not tested	3.2	2.3
SN00784252	CL2153	Fc1ccc(cc1)c1nc(NCc2ccco2)nc(c1)C(F)(F)F	3.2	2.3	not tested	not tested	3.2	2.3
SN00800487	CM1476	CCc1noc(n1)C1(CCCCC1)NC(=O)c1[nH]nc(c1)c1ccccc1	3.2	0.0	not tested	not tested	3.2	0.0
SN00789336	CL8884	Clc1ccc2OCC(=O)N(Cc3onc(n3)Cn3cnc4ccccc4c3=O)c2c1	3.2	0.0	not tested	not tested	3.2	0.0
SN00779057	SC009334	CC(C)N1CCN(CC1)C(=O)c1cnc2onc(C)c2c1	3.2	0.0	not tested	not tested	3.2	0.0
SN00795461	CL3964	Cc1ccc(C)c(c1)S(=O)(=O)Nc1ccc2nnc(c3ccccc3)n2n1	3.2	0.0	not tested	not tested	3.2	0.0
SN00798214	CL9545	O=C(C1CCC1)N1CCc2[nH]c3ccc(cc3c2C1)C(=O)NCCc1ccccn1	3.2	0.0	not tested	not tested	3.2	0.0
SN00791775	CL3568	COc1cccc(c1)c1noc(Nc2cccc(C)c2)n1	3.1	0.0	not tested	not tested	3.1	0.0
SN00796109	CL8537	Cc1cccc(c1)n1c(CN2CCN(CC2)C(=O)NCC2CCCO2)nc2cccnc12	3.1	0.7	not tested	not tested	3.1	0.7
SN00782700	SC000069	COc1cccc(c1)C1(C)NC(=O)N(Cc2csc(n2)c2ccccc2)C1=O	3.1	0.0	not tested	not tested	3.1	0.0
SN00773547	SC000009	O=C(COC(=O)C1(O)CCCCC1)c1cc(C)n(c1C)c1c(C)n(C)n(c2ccccc2)c1=O	3.1	1.1	not tested	not tested	3.1	1.1
SN00783032	SC010366	O=C(CCN1CCc2[nH]nc(c2C1)C(F)(F)F)NC(C)(C)C	3.0	9.6	not tested	not tested	3.0	9.6
SN00792083	CL3699	CCCNc1nc(N2CCN(CC)CC2)c2cnn(C)c2n1	3.0	10.1	not tested	not tested	3.0	10.1
SN00800558	CM1417	CCc1nnc(o1)c1nnc(NCc2ccc3OCOc3c2)c2ccccc12	3.0	0.0	not tested	not tested	3.0	0.0
SN00790428	CL7771B	COc1ccc(cc1)S(=O)(=O)N1CCN(Cc2ccc(C)cc2)c2ncccc12	3.0	10.3	not tested	not tested	3.0	10.3
SN00783737	CL6785B	Cn1cc(CNC(=O)c2cc(nc3ccccc23)c2ccc(C)c(C)c2)c(C)n1	3.0	0.0	not tested	not tested	3.0	0.0
SN00798503	CL9568	O=C(NC(C)(C)C)c1cc2sc(C)c(CN3CCOCC3)c2[nH]1	3.0	2.4	not tested	not tested	3.0	2.4
SN00785855	CL5371	CN(Cc1ccccc1)c1c2CCCc2nc2nncn12	3.0	14.3	not tested	not tested	3.0	14.3
SN00795713	CL8811A	Fc1ccc(cc1)c1noc(n1)c1cccc(c1)C(=O)N1CCN(CC1)c1ccccc1	3.0	0.9	not tested	not tested	3.0	0.9
SN00791620	CL6374	COc1c(OC)ccc(CN2CCN(CC2)C(=O)c2c(C)oc3ncn4CCN=c4c23)c1OC	3.0	7.4	not tested	not tested	3.0	7.4
SN00786616	CL9233	Cc1ccc(cc1)S(=O)(=O)[C@H]1CS(=O)(=O)C[C@@H]1N1CCC(CC1)C(=O)N	3.0	0.0	not tested	not tested	3.0	0.0
SN00799373	CL9952	O=C(CN1CCC(CC1)c1onc(n1)c1ccccc1)Nc1cccc(c1)C(=O)C	2.9	0.0	not tested	not tested	2.9	0.0
SN00792263	CL6617	CCC(=O)N1CCCc2ccc(cc12)NS(=O)(=O)c1ccc(F)c(C)c1	2.9	17.5	not tested	not tested	2.9	17.5
SN00787857	CL3586B	CC(=O)N1CCC2(CC1)OC(=O)C(=C2C(=O)NC1CC1)C	2.9	0.0	not tested	not tested	2.9	0.0
SN00783571	CL0385	CC(=O)Oc1ccc(cc1)C(=O)Nc1ccc2nc(oc2c1)c1ccc(Cl)cc1	2.9	0.0	not tested	not tested	2.9	0.0

Compound ID	Scaffold	Smiles	% Inhibition n=1		% Inhibition n=2		Average % Inhibition	
			24h	48h	24h	48h	24h	48h
SN00773335	SC015547	Clc1cccc(c1)c1noc(n1)C1CC21CCN(CC2)c1ncc(cc1Cl)C(=O)N	2.9	0.0	not tested	not tested	2.9	0.0
SN00787045	CL9708	Cc1ccc2nc([nH]c2c1)c1ccc(C)c(c1)NC(=O)c1cccnc1	2.9	8.2	not tested	not tested	2.9	8.2
SN00802413	CM2665	COCCN1CCC(N2CCCN(CC2)C(=O)NCCc2ccccc2)C1=O	2.9	1.7	not tested	not tested	2.9	1.7
SN00791845	CL7223A	O=C(CN1C(=O)COc2ccc(cc12)C(=O)NCCCN1CCCC1)N1CCCC1	2.9	0.0	not tested	not tested	2.9	0.0
SN00800474	CL2726A	O=C(N1CCN(CC1)c1ccccc1)C1CN(CCc2ccccc2)C(=O)C1	2.8	6.2	not tested	not tested	2.8	6.2
SN00799558	CM0152A	O=C(N(C)Cc1ccccc1)c1cccc2N(CCc12)C(=O)Nc1ccccc1	2.8	0.0	not tested	not tested	2.8	0.0
SN00775403	SC001417	CC1CCN(CC1)Cc1nc2sc(C)c(C)c2c(n1)N(C)Cc1nc2ccsc2c(=O)[nH]1	2.8	11.1	not tested	not tested	2.8	11.1
SN00801929	CM0954	O=C(N1CCCCC1)c1nc2n(ccnc2c2ccccc2)c1	2.8	30.6	not tested	not tested	2.8	30.6
SN00796867	CL9207B	CCCS(=O)(=O)N1CCc2n(C)nc(c3nnc(o3)c3ccc(F)cc3)c2C1	2.8	0.0	not tested	not tested	2.8	0.0
SN00801209	CM2967	O=C(Cc1ccccc1)N(C)CC1COCC1	2.8	0.0	not tested	not tested	2.8	0.0
SN00788097	CL5043	Cc1ccc(cc1)NC(=O)Cn1nnc(c2onc(n2)c2ccncc2)c1N	2.7	7.5	not tested	not tested	2.7	7.5
SN00798392	CL9640	Cc1nnc(o1)c1ccc2ccn(CC(=O)NC3CC3)c2c1	2.7	0.0	not tested	not tested	2.7	0.0
SN00807910	SC015171	Cc1ccc(o1)c1sc(c(C)n1)C(=O)N1CCn2c(nnc2C)C1	2.7	15.8	not tested	not tested	2.7	15.8
SN00773551	SC000010	Nc1nc(nc(n1)Nc1ccccc1C)CN1C(=O)NC(C)(C1=O)c1cccc2ccccc12	2.7	21.3	not tested	not tested	2.7	21.3
SN00797595	CL9224	O=C(Nc1ccc2sc(nc2c1)NS(=O)(=O)c1ccccc1)Nc1ccccc1Cl	2.7	4.6	not tested	not tested	2.7	4.6
SN00798663	CL9618	Cc1cc(C)cc(c1)C(=O)NCc1ccc2c(nnn2C)c1	2.7	0.0	not tested	not tested	2.7	0.0
SN00781992	SC015279	Cn1cnc2CN(CCc12)Cc1csc(n1)c1ccco1	2.7	24.5	not tested	not tested	2.7	24.5
SN00780656	SC011804	COC(=O)c1c(C(=O)OC)c2CCCn2c1c1ccc(cc1)NS(=O)(=O)c1ccc(C)s1	2.6	9.3	not tested	not tested	2.6	9.3
SN00788969	CL5809	Cc1onc(C)c1c1ccc(C)c(c1)S(=O)(=O)N1CCC(=CC1)c1ccccc1	2.6	0.0	not tested	not tested	2.6	0.0
SN00795015	CL7935	Cc1ccnc(n1)N1CCCC(C1)C(=O)NC1CCCC1	2.5	10.9	not tested	not tested	2.5	10.9
SN00783684	CL0192	Clc1ccc(OCCCC(=O)Nc2nc3ccccc3[nH]2)c(C)c1	2.5	27.8	not tested	not tested	2.5	27.8
SN00784837	CL3458	CSCCC1COC(=N1)c1cccn1C	2.5	0.0	not tested	not tested	2.5	0.0
SN00772233	SC012053	O=C(CCN1C(=O)CCC1=O)N1CCCC(C1)N1CCCCCC1	2.5	7.1	not tested	not tested	2.5	7.1
SN00783696	CL6785C	CN(Cc1cnn(C)c1C)C(=O)c1cc2nc(cc(n2n1)C(F)(F)F)c1cccs1	2.5	0.0	not tested	not tested	2.5	0.0
SN00802962	CL6785A	CCOc1ccc(cc1)S(=O)(=O)N(C)Cc1c(C)nn(C)c1C	2.5	2.3	not tested	not tested	2.5	2.3
SN00776353	SC003155	O=C1CC(=C(N)N1C1CC1)c1nc2ccccc2s1	2.5	0.0	not tested	not tested	2.5	0.0
SN00803147	CL9064	COc1ccc(cc1)CC(=O)NC1CCN(CC1)c1sc2c(C)nn(c3cccc(C)c3)c2n1	2.4	1.5	not tested	not tested	2.4	1.5
SN00802630	CL7967	Fc1ccc(cc1)N1CCN(CC1)C(=O)c1n[nH]c2CCN(Cc12)C(=O)c1ccc(F)cc1	2.4	0.9	not tested	not tested	2.4	0.9
SN00797030	CL9040	COc1ccc(cc1)S(=O)(=O)N(C)c1ccsc1C(=O)NCCC(C)C	2.4	10.4	not tested	not tested	2.4	10.4
SN00781423	SC014186	O=C(NCC1CCCO1)Cn1c(=O)onc1c1cccnc1	2.4	0.0	not tested	not tested	2.4	0.0
SN00788598	CL7467A	O=C(NC1CCCCCC1)C1CCCN(C1)c1ncnc2onc(C)c12	2.4	0.0	not tested	not tested	2.4	0.0
SN00784528	CL2003	Fc1ccc(cc1)C1NC(Cc2ccccc2)(C(=O)O)C2C1C(=O)N(c1ccc(F)cc1)C2=O	2.4	0.7	not tested	not tested	2.4	0.7
SN00780535	SC011610	O=C(CCNS(=O)(=O)c1cccnc1)N1CCCC(C1)c1nc2ccccc2o1	2.3	0.0	not tested	not tested	2.3	0.0
SN00783996	CL7522	CCOc1cc2c(CNC(c3cccc(c3)N(=O)=O)c3cccn23)cc1OCC	2.3	10.3	not tested	not tested	2.3	10.3
SN00783508	CL1995	CCOC(=O)c1sc2nc(C)nc(N3CCCCC3C)c2c1C	2.3	0.0	not tested	not tested	2.3	0.0
SN00789757	CL7900	O=C(N1CCCCC1)c1cccc(c1)c1noc(n1)c1cc2ccccc2oc1=O	2.3	9.8	not tested	not tested	2.3	9.8
SN00784702	CL4134	Cc1cccc(c1)NC(=O)CN1CC(CC1=O)c1ccccc1	2.3	10.4	not tested	not tested	2.3	10.4
SN00781319	SC014022	O=C(NCC1OCC2CCCN2C1)c1ccccc1n1cnnn1	2.3	0.0	not tested	not tested	2.3	0.0
SN00778148	SC007870	O=C(N1CCCN(CC1)C(=O)C)c1n[nH]c(c1)C1CC1	2.3	1.1	not tested	not tested	2.3	1.1
SN00799415	CL9953	O=C(NCc1ccc(F)cc1)CCn1ccn2nc(cc2c1=O)c1ccccc1Cl	2.2	0.0	not tested	not tested	2.2	0.0
SN00784607	CL2039	Clc1ccc(cc1)S(=O)(=O)C1(CC1)C(=O)N1CCN(CC1)c1cccc(Cl)c1	2.2	0.0	not tested	not tested	2.2	0.0
SN00784775	CL6984B	O=C(Nc1ccccc1)NC(c1ccccc1)c1nnc(o1)c1ccccc1Cl	2.2	0.0	not tested	not tested	2.2	0.0
SN00783149	SC012705	Fc1ccc(OCCN2Cc3ccccc3CC2c2nnc(C)o2)cc1	2.2	0.0	not tested	not tested	2.2	0.0
SN00777269	SC006996	COc1cc(ccc1OC)C(=O)N1C(C)CC(=O)Nc2ccccc12	2.2	0.0	not tested	not tested	2.2	0.0
SN00786562	CL8274	O=C(NC1CCCCC1)Nc1ccc(cc1)N1CCn2c(C1)nc1cc(Cl)ccc21	2.2	10.0	not tested	not tested	2.2	10.0
SN00787408	CL4469B	Fc1cccc(CNC(=O)C2CCCN(C2)c2nc3ccccc3n3cccc23)c1	2.2	0.0	not tested	not tested	2.2	0.0

Compound ID	Scaffold	Smiles	% Inhibition n=1		% Inhibition n=2		Average % Inhibition	
			24h	48h	24h	48h	24h	48h
SN00782451	SC008797	CCc1nnsc1C(=O)N1CCCN(CC1)Cc1ccccc1F	2.1	0.0	not tested	not tested	2.1	0.0
SN00787244	CL4597	O=C(NCCCN1CCN(CC1)c1ccccc1)c1nn(C)c2c1COc1ccccc21	2.1	0.0	not tested	not tested	2.1	0.0
SN00797416	CL9352	Cc1ccc(cc1)C(=O)N1CCCC1c1onc(n1)c1cccc(C)c1	2.1	0.0	not tested	not tested	2.1	0.0
SN00772110	SC011460	Fc1ccc(cc1)CNC(=O)N1CCCCC1c1onc(n1)c1ccccc1	2.1	0.0	not tested	not tested	2.1	0.0
SN00800668	CM1452	Fc1ccc(cc1C)NS(=O)(=O)c1cc(oc1C)c1[nH]ncc1C(=O)N1CCCC1	2.1	0.0	not tested	not tested	2.1	0.0
SN00771943	SC009489	COc1cccc(CCC(=O)N2CCCC(C2)c2nnc3CCCCCn23)c1	2.1	2.6	not tested	not tested	2.1	2.6
SN00776477	SC003542	Cc1oc(C)c(c1)c1cc(C(=O)Nc2c(C)n(C)n(c3ccccc3)c2=O)c2cnn(C(C)C)c2n1	2.0	0.0	not tested	not tested	2.0	0.0
SN00782373	SC007569	CCCCn1nnnc1CN1CCCC1Cn1cncn1	2.0	0.0	not tested	not tested	2.0	0.0
SN00795227	CL8798	Cc1ccc(cc1)S(=O)(=O)NC1CCN(CC1)C(=O)Cc1ccc(Br)cc1	2.0	3.1	not tested	not tested	2.0	3.1
SN00793315	CL8425	Cc1onc(n1)c1ccc(cc1)C(=O)N1CCCC1C(=O)Nc1ccc(cc1)C(C)C	2.0	0.0	not tested	not tested	2.0	0.0
SN00801485	CM1393	CCC(=O)N1CCc2c(C1)sc1ncnc(N(C)C3CCN(C)CC3)c21	2.0	0.0	not tested	not tested	2.0	0.0
SN00802522	CL2032	CCN(CC)CCCC(C)NC(=O)c1noc(c1)c1ccco1	2.0	3.7	not tested	not tested	2.0	3.7
SN00795207	CL8595	Cc1ccc2oc(c3noc(n3)C(=O)NCC3CCCO3)c(C)c2c1	2.0	0.0	not tested	not tested	2.0	0.0
SN00795042	CL6301A	Cc1ccc(cc1)C1N(Cc2ccccc2n2cccc12)C(=O)c1cccnc1	2.0	0.0	not tested	not tested	2.0	0.0
SN00781429	SC014186	CCn1c(Cn2c(=O)onc2c2cccnc2)nc2c(F)cccc12	1.9	0.0	not tested	not tested	1.9	0.0
SN00788609	CL7467A	O=C(N1CCCCCC1)C1CCCN(C1)c1ncnc2onc(C)c12	1.9	0.0	not tested	not tested	1.9	0.0
SN00784820	CL2506	Cc1ccc(cc1)NS(=O)(=O)c1c(C)n[nH]c1C	1.9	8.8	not tested	not tested	1.9	8.8
SN00782289	SC016338	O=C(CCc1cnn(c1)c1ccccc1)Nc1n[nH]c(c1)C1CCOCC1	1.9	8.0	not tested	not tested	1.9	8.0
SN00787062	CL1392	COc1ccccc1CCC(=O)N1CCN(CC1)c1ncnc2[nH]cnc12	1.8	0.0	not tested	not tested	1.8	0.0
SN00794266	CL1358	Fc1ccc(cc1)NC(=O)c1nc2nc(cc(n2n1)C(F)(F)F)c1cccs1	1.8	18.7	not tested	not tested	1.8	18.7
SN00797622	CL9960	Cc1ccnc(c1)Nc1nc(C)c(s1)C(=O)NC1CC1	1.7	13.3	not tested	not tested	1.7	13.3
SN00778131	SC007869	CC(=O)N1CCCN(CC1)C(=O)c1cnn(c1C)C(C)(C)C	1.7	0.0	not tested	not tested	1.7	0.0
SN00787709	CL5114	O=C1c2ccc(c3ccco3)n2CC(C)(C(=O)NCc2ccccc2)N1CCc1ccco1	1.7	0.0	not tested	not tested	1.7	0.0
SN00776382	SC003225	O=C(NC1CCCCCC1)c1c(C)c(C)sc1n1cccc1	1.7	0.0	not tested	not tested	1.7	0.0
SN00798195	CL9545	COc1ccc(OC)cc1CNC(=O)c1ccc2[nH]c3CCN(Cc3c2c1)C(=O)C	1.6	0.0	not tested	not tested	1.6	0.0
SN00794807	CL8624	COc1cc(OC)c(Cl)cc1NC(=O)N1CCN(CC1)c1nc2ccccc2n1C	1.6	0.0	not tested	not tested	1.6	0.0
SN00791700	CL6877	Clc1ccc(cc1)c1n[nH]cc1C(=O)N1CCN(CC1)c1ccccc1	1.6	26.6	not tested	not tested	1.6	26.6
SN00774529	SC000912	Cc1ccc(cc1)c1nc(sc1CC(=O)Nc1ccc2nc(C)sc2c1)c1cccnc1	1.6	0.0	not tested	not tested	1.6	0.0
SN00774718	SC000971	Cc1cnc(cn1)C(=O)N1CCN(C(C)C1)C(=O)OC(C)(C)C	1.6	0.0	not tested	not tested	1.6	0.0
SN00776123	SC003068	O=C1COc2ccc(cc2N1)C(=O)c1sc(nc1c1ccccc1)c1c(C)nc2sccn12	1.6	7.0	not tested	not tested	1.6	7.0
SN00800151	CM0330A	O=C(N1CCC(Oc2nccnc2N2CCc3ccccc23)C1)c1ccoc1	1.5	22.8	not tested	not tested	1.5	22.8
SN00786472	CL8245A	Cc1ccc(cc1)Nc1nc(C)nc(c1)N1CCN(CC1)S(=O)(=O)c1ccc(C)c(C)c1	1.5	20.7	not tested	not tested	1.5	20.7
SN00792818	CL7253A	Fc1cccc(COC2CN(C2)S(=O)(=O)c2cccc(F)c2)c1	1.5	10.0	not tested	not tested	1.5	10.0
SN00791727	CL6874	O=C(N1CCN(CC1)C(=O)c1ccco1)c1noc(CN2CCCC2)c1	1.5	0.0	not tested	not tested	1.5	0.0
SN00791146	CL3586C	CCS(=O)(=O)N1CCC2(CC1)OC(=O)C(=C2C(=O)Nc1ccc(C)cc1C)C	1.5	0.0	not tested	not tested	1.5	0.0
SN00798844	CL9717	CC(C)NS(=O)(=O)c1cc2c(CCN2C(=O)C2CC2)cc1C	1.4	15.5	not tested	not tested	1.4	15.5
SN00770003	SC001352	Fc1ccc(cc1)C1=CCN(CC1)C(=O)C1CCN(CC1)C(=O)C(C)(C)C	1.4	0.0	not tested	not tested	1.4	0.0
SN00795593	CL6768A	O=C(NCCc1c[nH]c2ccccc12)CCC(=O)N1CCCN(CC1)c1sc2ncccc2n1	1.4	0.0	not tested	not tested	1.4	0.0
SN00793181	CL1995	OC(=O)c1sc2ncnc(NC3CCCCCC3)c2c1C	1.4	3.6	not tested	not tested	1.4	3.6
SN00784814	CL3311	Clc1ccc(cc1)CNC(=O)CN1CCN(Cc2ccccc2)C1=O	1.4	0.0	not tested	not tested	1.4	0.0
SN00783222	SC014110	O=C(Cc1ccc2CCCc2c1)N1CCC(CC1)n1cncn1	1.4	6.3	not tested	not tested	1.4	6.3
SN00793573	CL7356	Cc1ccc(cc1)NC(=O)c1ccc(=O)n(CCN2CCOCC2)n1	1.4	2.3	not tested	not tested	1.4	2.3
SN00781984	SC015279	O=C(Cc1scc(n1)CN1CCc2c(ncn2C)C1)N(C)C	1.4	0.0	not tested	not tested	1.4	0.0
SN00769908	SC001339	O=C(NC(C)c1scc(n1)c1cccnc1)C1CCOCC1	1.3	0.0	not tested	not tested	1.3	0.0
SN00773517	SC016401	CC(C)n1nc2CCN(Cc3onc(n3)c3cocc3)Cc2cc1=O	1.3	10.7	not tested	not tested	1.3	10.7
SN00798811	CL9705	COc1c(oc2ccc(NC(=O)c3ccc(C)s3)cc12)C(=O)Nc1ccc(C)cc1	1.3	19.9	not tested	not tested	1.3	19.9

Compound ID	Scaffold	Smiles	% Inhibition n=1		% Inhibition n=2		Average % Inhibition	
			24h	48h	24h	48h	24h	48h
SN00792712	CL4870	CCOC(=O)Cc1ccc(cc1)NC(=O)N1CCC2(CC1)NC(=O)C(=N2)c1ccc(F)cc1	1.3	14.3	not tested	not tested	1.3	14.3
SN00773912	SC000646	Clc1ccc(F)c(c1)NC(=O)Cn1c(=O)c2n(cnc2n(Cc2ccccc2)c1=O)C1CCCC1	1.3	0.0	not tested	not tested	1.3	0.0
SN00800353	CM1204A	COc1ccc(cc1)c1n[nH]c(c1)N1CCN(CC1)C(=O)Cc1ccc(F)cc1	1.2	0.0	not tested	not tested	1.2	0.0
SN00799991	CM0324	O=S(=O)(Nc1ccc(nc1)OC1CCNCC1)c1ccccc1	1.2	0.0	not tested	not tested	1.2	0.0
SN00798340	CL9596	CN(C)CCN(Cc1cccs1)S(=O)(=O)c1cc(sc1C)c1[nH]nc(C)c1	1.2	13.6	not tested	not tested	1.2	13.6
SN00782604	SC013675	COCCS(=O)(=O)N1CCCC1Cn1cncc1	1.2	0.0	not tested	not tested	1.2	0.0
SN00799393	CL9952	O=C(NCc1ccccc1)CN1CCC(CC1)c1onc(n1)c1ccc(F)cc1	1.2	0.0	not tested	not tested	1.2	0.0
SN00791583	CL5797A	CCCN1C(=O)c2n(cnc2C(=O)NCCc2ccccc2)CC1(C)C(=O)NCCC(C)C	1.2	9.0	not tested	not tested	1.2	9.0
SN00797407	CL9351A	Cc1ccc(cc1)NC(=O)c1ccc(cc1)c1noc(n1)C1CCCN(C1)C(=O)c1ccccc1	1.2	5.6	not tested	not tested	1.2	5.6
SN00772435	SC012606	CCOC(=O)c1ccsc1NC(=O)CN1CCNC(=O)CC1c1ccccc1	1.2	0.0	not tested	not tested	1.2	0.0
SN00801675	CM1450	O=C(c1cnccn1)N1CCCC1c1onc(n1)c1ccccc1	1.2	0.0	not tested	not tested	1.2	0.0
SN00784679	CL4006	Clc1ccc(cc1)Nc1nc2ccccc2n2nnnc12	1.2	4.0	not tested	not tested	1.2	4.0
SN00792604	CL4965A	CCOc1ccc(cc1)S(=O)(=O)Nc1cccc(c1)c1ccc(OC)nn1	1.1	10.8	not tested	not tested	1.1	10.8
SN00785530	CL4805A	CCOC(=O)c1nn2CC(C)(C(=O)NCCC(C)C)N(CCc3cccs3)C(=O)c2c1	1.1	6.6	not tested	not tested	1.1	6.6
SN00793191	CL9791	O=C(Nc1ccc(nc1)S(=O)(=O)N)c1c(C)sc2ncn(CC3CCCO3)c(=O)c12	1.1	0.0	not tested	not tested	1.1	0.0
SN00797953	CL9439A	COc1cccc(CNC(=O)C2CCCN(C2)c2nccc3c(C)nn(C)c23)c1	1.1	0.0	not tested	not tested	1.1	0.0
SN00798718	CL9678	O=C(Cn1c(=O)cc(c2onc(n2)C(C)C)c2ccccc12)N1CCCC1	1.1	0.0	not tested	not tested	1.1	0.0
SN00798481	CL5449A	COc1ccc(c2onc(n2)CS(=O)(=O)c2ccc(C)cc2)c(C)c1	1.1	11.0	not tested	not tested	1.1	11.0
SN00779129	SC009460	Brc1ccc(cc1)S(=O)(=O)N1CCN(CC1)C(=O)C1CC(=O)Nc2cc(F)ccc12	1.1	0.0	not tested	not tested	1.1	0.0
SN00771886	SC009223	C1CCc2nnc(C3CCCN3c3nc4ccccc4s3)n2CC1	1.1	12.0	not tested	not tested	1.1	12.0
SN00778765	SC008738	Cc1oc(CN2CCNCCC2)nc1C	1.1	0.0	not tested	not tested	1.1	0.0
SN00778709	SC008677	CCn1ncc(CN2CCCN(CC2)C(=O)c2noc(c2)C2CC2)c1	1.1	0.0	not tested	not tested	1.1	0.0
SN00791741	CL6846	Clc1ccc(cc1)N1CCN(CC1)C(=O)CN1CCCn2nc(C)cc12	1.1	2.4	not tested	not tested	1.1	2.4
SN00797257	CL9349	Cc1noc(c1)c1csc(c1)S(=O)(=O)Nc1ccc(Cl)c(c1)C(F)(F)F	1.1	0.0	not tested	not tested	1.1	0.0
SN00780594	SC011739	Cc1scc(CN2CCN(CC2)C(=O)c2c(=O)[nH]nc(C)c2C)n1	1.1	0.0	not tested	not tested	1.1	0.0
SN00774484	SC000902	CCOC(=O)C1CCN(CC1)C(=O)c1ccc2C(=O)N(C(=O)c2c1)c1ccccc1C	1.1	0.0	not tested	not tested	1.1	0.0
SN00798707	CL9678	CCOC(=O)c1ccc(cc1)NC(=O)Cn1c(=O)cc(c2onc(C)n2)c2ccccc12	1.1	2.1	not tested	not tested	1.1	2.1
SN00770936	SC006833	NC(=O)c1cn(Cc2onc(n2)c2cocc2)nc1c1ccccc1	1.1	0.0	not tested	not tested	1.1	0.0
SN00790501	CL8375	CCOc1ccc(cc1)n1c(=O)n(CC(=O)N2CCCCC2)c2c3cccnc3sc2c1=O	1.0	5.1	not tested	not tested	1.0	5.1
SN00781291	SC013994	Cc1ccccc1c1noc(n1)Cn1c(=O)onc1c1ccccn1	1.0	0.0	not tested	not tested	1.0	0.0
SN00782137	SC015980	Cc1[nH]nc(CN2CCN(CC2)C(=O)c2ccc(cc2)Cn2nc(C)cc2C)n1	1.0	1.5	not tested	not tested	1.0	1.5
SN00790483	CL8372A	Cc1ccc(cc1)NC(=O)c1nnc(s1)C1CCCN1C(=O)NC1CCCC1	1.0	4.4	not tested	not tested	1.0	4.4
SN00769994	SC001352	O=C(C1CCN(CC1)S(=O)(=O)c1ccccc1)N1CCC(=CC1)c1ccccc1	1.0	0.0	not tested	not tested	1.0	0.0
SN00784617	CL1545	CCOc1ccc(cc1)n1c(=O)c2c3CCCc3sc2n(Cc2cccnc2)c1=O	1.0	0.0	not tested	not tested	1.0	0.0
SN00774515	SC000907	CC(C)Cn1nc(C(=O)N2CCCCC2c2nc3ccccc3s2)c2ccccc2c1=O	1.0	2.8	not tested	not tested	1.0	2.8
SN00798102	CL9469	Cc1ccc(cc1)c1noc(n1)c1cccn2c1nn(CC(=O)Nc1cc(C)ccc1C)c2=O	1.0	23.0	not tested	not tested	1.0	23.0
SN00787754	CL4881	Cc1ccc(cc1)n1nnc(c2onc(n2)c2ccc3OCOc3c2)c1N	1.0	0.0	not tested	not tested	1.0	0.0
SN00791374	CL6597	COCCCN1C(=O)CSc2c(c(C)nn2c2ccccc2)C1C(=O)NCc1ccccc1OC	1.0	0.0	not tested	not tested	1.0	0.0
SN00795932	CL8682B	COc1ccc(cc1)S(=O)(=O)N1CCCC(C)(C1)C(=O)NCc1ccc(F)cc1	1.0	8.4	not tested	not tested	1.0	8.4
SN00782901	SC008030	COCCN1CCCN(CC1)C(=O)C1CCCN(C1)S(=O)(=O)C	0.9	15.1	not tested	not tested	0.9	15.1
SN00795313	CL5144A	CC(C)NC(=O)N1CCN(CC1)c1nn2c(s1)nc(c1cccc(C)c1)c2NC(C)(C)C	0.9	0.0	not tested	not tested	0.9	0.0
SN00785951	CL3359	O=C1CC(CN1C)c1nc2ccccc2[nH]1	0.8	0.0	not tested	not tested	0.8	0.0
SN00789389	CL7658	O=C(NCc1cccs1)CN1C(=O)C2CCCC2=Nc2cc(C)c(C)cc12	0.8	11.1	not tested	not tested	0.8	11.1
SN00780555	SC011610	O=C1OCCN1CCCN1CCCC(C1)c1nc2ccccc2o1	0.8	0.0	not tested	not tested	0.8	0.0
SN00795530	CL9139	CNC(=O)Nc1ccc(cc1)C1(CCC1)C(=O)Nc1ccc(F)c(C)c1	0.8	0.0	not tested	not tested	0.8	0.0
SN00787839	CL3586A	CCCCN(CC)CCNC(=O)C1=C(C)C(=O)OC21CCN(CC)CC2	0.7	0.0	not tested	not tested	0.7	0.0

Compound ID	Scaffold	Smiles	% Inhibition n=1		% Inhibition n=2		Average % Inhibition	
			24h	48h	24h	48h	24h	48h
SN00782733	SC000953	O=C(C1CCCC1)N1CCC(C1)c1ccccc1C	0.7	7.3	not tested	not tested	0.7	7.3
SN00781376	SC014183	O=C(Cn1c(=O)onc1c1ccccn1)N1CCc2sccc2C1	0.7	0.0	not tested	not tested	0.7	0.0
SN00781524	SC014214	CCc1csc(n1)C1CCCN(C1)C(=O)c1ccc2OCOc2c1	0.7	0.0	not tested	not tested	0.7	0.0
SN00771493	SC008256	C/C=C/c1ccc(OCC(=O)N2CCCN(CC2)C(=O)C2CCCC2)c(OC)c1	0.7	16.5	not tested	not tested	0.7	16.5
SN00802129	CM1459	C1CCC(CN1)c1nnc([nH]1)c1nccnc1	0.7	2.3	not tested	not tested	0.7	2.3
SN00790002	CL8109	CC(C)Cn1c(=O)c(N2CCC(CC2)C(=O)NC2CCCCCC2)c(C)n(c2ccccc2)c1=O	0.7	9.5	not tested	not tested	0.7	9.5
SN00771426	SC008246	O=C(N1CCCN(CC1)c1cccnn1)c1cc(C)n(C(C)C)c1C	0.6	0.0	not tested	not tested	0.6	0.0
SN00796055	CL9297	Cc1ccc(c(C)c1)S(=O)(=O)N1CCC2(NCCN=C2NC2CCCCCC2)CC1	0.6	0.0	not tested	not tested	0.6	0.0
SN00789235	CL6673A	CCOc1ccc(CC(=O)N2CCC3(CC2)Nc2ccccc2n2cccc32)cc1OCC	0.6	16.8	not tested	not tested	0.6	16.8
SN00773167	SC015213	c1ccc(cc1)c1nnc(CN2CCn3c(nnc3C3CC3)C2)o1	0.6	0.0	not tested	not tested	0.6	0.0
SN00786185	CL7099	CC(C)NC(=O)c1sc(nc1C)n1nc(C)c(Cc2ccccc2Cl)c1C	0.6	0.0	not tested	not tested	0.6	0.0
SN00807954	SC015103	Cc1oc(nc1CN1CCc2cnc(nc2C1)C(C)C)c1ccco1	0.6	0.1	not tested	not tested	0.6	0.1
SN00800036	CM0327	Cc1ccc(c(C)c1)C(=O)N1CCC(Oc2ncccc2C(=O)N2CCCCC2)C1	0.6	0.0	not tested	not tested	0.6	0.0
SN00794535	CL2560	CCN(CC)S(=O)(=O)c1ccc2[nH]cc(C(=O)NCCCN3CCCC3)c(=O)c2c1	0.6	13.0	not tested	not tested	0.6	13.0
SN00773994	SC000743	COc1ccc(cc1)c1nnc(o1)CN1C(=O)NC(C)(CCC(C)C)C1=O	0.6	0.0	not tested	not tested	0.6	0.0
SN00797886	CL9391	COc1cccc(CNC(=O)[CH]n2ccc3sccc3c2=O)c1	0.5	6.6	not tested	not tested	0.5	6.6
SN00801741	CM1591	O=C(N1CCC2(CC1)ON=C(N2)c1ccccc1)c1ccc(cc1)C(C)(C)C	0.5	0.0	not tested	not tested	0.5	0.0
SN00801654	CM2805	O=C(NCc1ccccc1Br)Cn1nc2CCCCn2c1=O	0.4	6.0	not tested	not tested	0.4	6.0
SN00798020	CL9455	Brc1cccc(c1)Cn1nc2n(cccc2S(=O)(=O)N2CCOCC2)c1=O	0.4	0.0	not tested	not tested	0.4	0.0
SN00802202	CM1646	Cc1nc(C)c2CCC(=O)N(CCN(C)C3CCCCC3)c2n1	0.4	0.0	not tested	not tested	0.4	0.0
SN00785614	CL4062	O=C(Cn1cc2CC(C)CCc2n1)NC(C)c1ccccc1	0.4	0.0	not tested	not tested	0.4	0.0
SN00793974	CL8307C	CCc1noc(c1)C1CCCN1C(=O)Nc1cc(C)cc(C)c1	0.4	2.9	not tested	not tested	0.4	2.9
SN00775953	SC002957	Fc1ccc(CCC(=O)Nc2ccn(n2)Cc2ccccn2)cc1	0.4	0.0	not tested	not tested	0.4	0.0
SN00789400	CL7811	CSc1ccc(cc1)c1noc(n1)c1ccc2c(c1)[nH]c(=O)n(Cc1ccccc1)c2=O	0.3	6.5	not tested	not tested	0.3	6.5
SN00797576	CL9398A	COc1ccc(OC)cc1NC(=O)C1CC(=O)N(C1)c1cccc(NC(=O)C)c1	0.3	0.0	not tested	not tested	0.3	0.0
SN00787693	CL5114	Fc1cccc(c1)N1C(=O)c2ccc(c3ccco3)n2CC1(C)C(=O)NC1CCCC1	0.3	11.7	not tested	not tested	0.3	11.7
SN00779392	SC009943	Fc1ccc(cc1)CN1N=C(CCC1=O)C(=O)Nc1ccc(cc1)N(C)C	0.3	8.0	not tested	not tested	0.3	8.0
SN00787800	CL4252	CCCn1cc(c(=O)c2cc(F)c(cc12)N1CCOCC1)S(=O)(=O)c1ccc(Cl)cc1	0.3	0.0	not tested	not tested	0.3	0.0
SN00772921	SC014250	O=C(Nc1cccc(c1)c1noc(n1)C1CCOCC1)C1CC1	0.3	0.0	not tested	not tested	0.3	0.0
SN00790942	CL8429	CCOC(=O)c1cccc(c1)NC(=O)c1cnc(C)nc1C	0.3	0.0	not tested	not tested	0.3	0.0
SN00783840	CL2609	O=C(CC1(O)C(=O)Nc2ccccc12)c1c(C)coc1C	0.2	0.0	not tested	not tested	0.2	0.0
SN00795232	CL8798	Clc1ccc(cc1)S(=O)(=O)NC1CCN(CC1)C(=O)c1ccco1	0.2	0.0	not tested	not tested	0.2	0.0
SN00772783	SC013675	CCc1nccn1CC1CCCN1	0.2	0.0	not tested	not tested	0.2	0.0
SN00794671	CL4756	CN(CCCNC(=O)c1cc2c(C)nccc2[nH]c1=O)C1CCCCC1	0.2	0.0	not tested	not tested	0.2	0.0
SN00793699	CL7417	Cn1cnc2cc(ccc12)c1noc(n1)C1CCCO1	0.2	22.8	not tested	not tested	0.2	22.8
SN00786524	CL8267	CCOC(=O)Cn1c2nc3CC(C)(C)OCc3cc2c(=O)n(c2ccccc2)c1=O	0.2	18.7	not tested	not tested	0.2	18.7
SN00790900	CL8199B	CCC(C)NC(=O)N1CCc2c(C1)sc1nc(nn21)c1ccc(C)cc1	0.1	10.9	not tested	not tested	0.1	10.9
SN00775113	SC001098	O=C(N1CCc2ccccc12)c1cnn(c2ccccc2)c1C1CC1	0.1	0.5	not tested	not tested	0.1	0.5
SN00796222	CL4659	O=C(NCCc1cccs1)Cc1csc2nc(cn12)c1ccccc1	0.1	0.0	not tested	not tested	0.1	0.0
SN00791445	CL6014	Cn1c(C)c(C(=O)N2CCCCC2)c(c1C)S(=O)(=O)N1CCN(CC1)c1ccccn1	0.1	0.0	not tested	not tested	0.1	0.0
SN00769798	SC001283	O=c1oc2ccccc2cc1c1csc(n1)NC(=O)c1cccc(c1)C(F)(F)F	0.1	0.0	not tested	not tested	0.1	0.0
SN00789144	CL6279	Cc1noc(c1C)c1c(C)sc(C)c1S(=O)(=O)NCc1ccccc1	0.0	30.5	not tested	not tested	0.0	30.5
SN00782943	SC008677	COc1ccc(cc1)CCC(=O)N1CCCN(CC1)C(=O)c1noc(c1)c1cccc(Cl)c1	0.0	0.0	not tested	not tested	0.0	0.0
SN00797341	CL9333	C=CCn1c(=O)c(nc2cccnc12)N1CCC(CC1)NC(=O)NC1CCCC1	0.0	6.8	not tested	not tested	0.0	6.8
SN00777772	SC007616	Fc1ccc(cc1)Cn1nnn(c2ccccc2)c1=O	0.0	48.5	not tested	not tested	0.0	48.5
SN00771536	SC008739	CC(=O)N1CCCN(CC1)Cc1ncc(Cl)n1C	0.0	46.7	not tested	not tested	0.0	46.7

Compound ID	Scaffold	Smiles	% Inhibition n=1		% Inhibition n=2		Average % Inhibition	
			24h	48h	24h	48h	24h	48h
SN00775829	SC002899	Clc1ccc(cc1)CNC(=O)Cn1nnc(n1)c1cccs1	0.0	45.7	not tested	not tested	0.0	45.7
SN00778123	SC007869	Cc1ccc(cc1)S(=O)(=O)N1CCCN(CC1)C(=O)c1c[nH]nc1c1ccc(F)cc1	0.0	40.4	not tested	not tested	0.0	40.4
SN00790290	CL7943A	COc1ccccc1CN1CCN(CC1)c1nc2ccc(NC(=O)C)cc2c(c1)C(=O)O	0.0	39.8	not tested	not tested	0.0	39.8
SN00775781	SC002863	CCCCn1c(=O)[nH]c(=O)c2c1nc(c1ccc(o1)c1cccc(c1)N(=O)=O)n2CC	0.0	37.2	not tested	not tested	0.0	37.2
SN00776915	SC006480	CCCNC(=O)CCNC(=O)N1CCCC1c1nc2cc(C)ccc2[nH]1	0.0	37.1	not tested	not tested	0.0	37.1
SN00775216	SC001182	COc1ccccc1N1CCN(CC1)C(=O)c1ccc2[nH]c(C)c(C)c2c1	0.0	36.6	not tested	not tested	0.0	36.6
SN00777724	SC007577	Cc1noc(c1)C1CCCN1Cc1noc(c1)c1ccco1	0.0	35.3	not tested	not tested	0.0	35.3
SN00774591	SC000942	COc1ccc(cc1)C1CCCN1C(=O)c1cnn(c2ccc(cn2)C(F)(F)F)c1C	0.0	35.2	not tested	not tested	0.0	35.2
SN00786006	CL5141	O=C(NC1CCN(CC1)c1nc(C)cc(C)n1)Nc1cccc(C)c1	0.0	33.8	not tested	not tested	0.0	33.8
SN00781176	SC013513	CN1CCN(CC1)CC1CCN(CC1)c1ccc(cc1Cl)N(=O)=O	0.0	33.0	not tested	not tested	0.0	33.0
SN00778045	SC007829	CCCNC(=O)CN1CCN(CC1)c1nc2ccccn2c1N(=O)=O	0.0	31.4	not tested	not tested	0.0	31.4
SN00778114	SC007868	CCc1noc(C)c1C(=O)N1CCCN(CC1)Cc1csc(C)n1	0.0	29.4	not tested	not tested	0.0	29.4
SN00776900	SC006480	O=C(CCC(=O)c1ccccc1)N1CCCC1c1nc2ccccc2[nH]1	0.0	28.7	not tested	not tested	0.0	28.7
SN00778011	SC007767	CCN1C(=O)C(=O)N(Cc2coc(n2)c2ccc(C)cc2)C1=O	0.0	28.7	not tested	not tested	0.0	28.7
SN00772586	SC013363	O=C(CCN1CCC(CC1)c1n[nH]c(n1)C1CC1)Nc1ccccc1	0.0	28.6	not tested	not tested	0.0	28.6
SN00784165	CL4067	CCc1ccc(cc1)NC(=O)[C@H]1[C@@H]2CCCCN2C(=O)c2cc(OC)c(OC)cc12	0.0	28.1	not tested	not tested	0.0	28.1
SN00793357	CL1071	Brc1ccc(cc1)S(=O)(=O)c1nc(C)oc1NCc1ccccc1	0.0	27.4	not tested	not tested	0.0	27.4
SN00772601	SC013374	COC(=O)c1ccc(F)c(c1)S(=O)(=O)NC1(CCC1)c1scc(C)n1	0.0	27.1	not tested	not tested	0.0	27.1
SN00785395	CL4469D	Cc1ccc(cc1)C(=O)N1CCN(CC1)c1nc2ccccc2n2cccc12	0.0	25.5	not tested	not tested	0.0	25.5
SN00800900	CM1851	CCC1N=c2cc(ncn2C1)N1CCN(CC1)C(=O)CCc1ccccc1OC	0.0	25.4	not tested	not tested	0.0	25.4
SN00786331	CL7370	CCCCNc1ncccc1c1onc(n1)c1ccc(C)cc1	0.0	25.3	not tested	not tested	0.0	25.3
SN00772623	SC013389	O=C1SCCC1NC(=O)c1cc2c(n1Cc1ccccc1)n(C)c(=O)n(C)c2=O	0.0	25.0	not tested	not tested	0.0	25.0
SN00776030	SC003012	O=S1(=O)CCC(Nc2nc(nc3scc(c4ccccc4)c23)c2ccccn2)C1	0.0	24.9	not tested	not tested	0.0	24.9
SN00797520	CL9375	CCCNC(=O)Cn1cccc1c1scc(n1)c1ccc(Cl)cc1	0.0	24.8	not tested	not tested	0.0	24.8
SN00772581	SC013363	Fc1ccc(cc1)C(NC(=O)CN1CCC(CC1)c1n[nH]c(C)n1)c1cccs1	0.0	24.6	not tested	not tested	0.0	24.6
SN00775761	SC002718	COCCc1ccc(cc1)Oc1nnnn1c1ccccc1	0.0	24.6	not tested	not tested	0.0	24.6
SN00786842	CL9431	Cc1ccc2oc(nc2n1)N1CCN(CC1)C(=O)C(C)c1ccccc1	0.0	24.1	not tested	not tested	0.0	24.1
SN00782004	SC015319	NCC1OCCN(C1)C1CC1	0.0	23.7	not tested	not tested	0.0	23.7
SN00795676	CL8811	CC1CCN(CCCNC(=O)c2ccc(cc2)c2onc(n2)c2ccccc2)CC1	0.0	23.6	not tested	not tested	0.0	23.6
SN00785474	CL3589	COc1ccc(cc1)S(=O)(=O)N1CCC(=CC1)C(=O)NCc1ccc(C)cc1	0.0	23.4	not tested	not tested	0.0	23.4
SN00794352	CL1623	COC(=O)c1c(NC(=O)Cn2nc(c(Br)c2C)C(F)(F)F)sc2CCCCc12	0.0	23.2	not tested	not tested	0.0	23.2
SN00784242	CL1106	O=C(NCc1ccccc1Cl)[C@@H]1[C@H]2C=C[C@]3(CN(Cc4cccs4)C(=O)[C@H]13)O2	0.0	22.4	not tested	not tested	0.0	22.4
SN00779630	SC010215	CCc1nc(C)c2c(n1)sc(C(=O)N1CCN(CC1)c1ncccn1)c2C	0.0	22.3	not tested	not tested	0.0	22.3
SN00792030	CL7144A	COc1ccc(cc1S(=O)(=O)Nc1ccc(C)cc1)c1cnc(C)o1	0.0	22.0	not tested	not tested	0.0	22.0
SN00781381	SC014183	O=c1onc(c2ccccn2)n1CC(=O)C12CC3CC(CC(C3)C2)C1	0.0	21.7	not tested	not tested	0.0	21.7
SN00789527	CL7971	CCOC(=O)c1csc(n1)n1nc(cc1C(F)(F)F)c1cccc(OC(=O)C)c1	0.0	21.7	not tested	not tested	0.0	21.7
SN00772513	SC013041	Cn1ncc2c(=O)[nH]c(CN3CCCC3Cc3ccccc3)nc12	0.0	21.0	not tested	not tested	0.0	21.0
SN00772607	SC013374	Cc1csc(n1)C1(CCC1)NC(=O)Nc1ccc2OCCc2c1	0.0	20.7	not tested	not tested	0.0	20.7
SN00780810	SC012060	CCCc1cc(N2CCOCC2)n2nc(C)c(c3ccccc3)c2n1	0.0	20.2	not tested	not tested	0.0	20.2
SN00796296	CL8825	O=C(NCc1ccccc1)c1c(C)sc2cc(oc12)c1ccccc1	0.0	20.2	not tested	not tested	0.0	20.2
SN00791316	CL6359	COc1ccc(Cl)cc1n1nnc(c2nsc(NC(=O)c3ccccc3)n2)c1C	0.0	20.0	not tested	not tested	0.0	20.0
SN00782812	SC005774	CC(NCc1onc(n1)c1ccccn1)Cn1ncc(C)c1	0.0	19.9	not tested	not tested	0.0	19.9
SN00797883	CL9391	O=C(Nc1ccccc1C)[CH]n1ccc2sccc2c1=O	0.0	19.9	not tested	not tested	0.0	19.9
SN00775787	SC002863	CCCCn1c(=O)[nH]c(=O)c2c1nc(c1ccc(o1)c1ccc(cc1Cl)N(=O)=O)n2C	0.0	19.8	not tested	not tested	0.0	19.8
SN00784853	CL3458	COc1ccc(cc1)Cn1cccc1C1=NC(C)(C)CO1	0.0	19.2	not tested	not tested	0.0	19.2
SN00775768	SC002718	c1ccc(cc1)n1nnnc1Oc1ccc(cc1)c1nc2ccccc2s1	0.0	19.1	not tested	not tested	0.0	19.1

Compound ID	Scaffold	Smiles	% Inhibition n=1		% Inhibition n=2		Average % Inhibition	
			24h	48h	24h	48h	24h	48h
SN00800580	CM1424	CCn1ccc(n1)C(=O)Nc1ccc2c(c1)Sc1ncccc1C(=O)N2C	0.0	19.0	not tested	not tested	0.0	19.0
SN00777999	SC007767	Clc1ccc(cc1)c1occ(CN2C(=O)N(Cc3ccccc3)C(=O)C2=O)n1	0.0	18.6	not tested	not tested	0.0	18.6
SN00791607	CL6374	CCOc1cc(CCNC(=O)c2c(C)oc3ncn4CCN=c4c23)ccc1OCC	0.0	17.8	not tested	not tested	0.0	17.8
SN00783181	SC013513	CN1CCN(CC1)CC1CCN(CC1)C(=O)Cc1cccc2ccccc12	0.0	17.7	not tested	not tested	0.0	17.7
SN00776937	SC006559	Cc1cccc(C)c1NC(=O)c1cc(nc2n(ncc12)Cc1cccnc1)c1ccccc1	0.0	17.5	not tested	not tested	0.0	17.5
SN00793530	CL6605B	CN1CCN(CC1)c1cc(C)nc(n1)N1CCN(CC1)C(=O)Nc1cccc(C)c1	0.0	17.4	not tested	not tested	0.0	17.4
SN00785221	CL4240	CCn1c2ccccc2c(C2=CCN(CC2)S(=O)(=O)c2ccc(F)cc2)c1C	0.0	17.2	not tested	not tested	0.0	17.2
SN00789076	CL4852	Clc1ccc(C)c(c1)NC(=O)C1CC2CSCN2C21C(=O)Nc1ccccc21	0.0	17.2	not tested	not tested	0.0	17.2
SN00772247	SC012104	O=C(N1CCCC1c1[nH]cc(n1)c1cccc(c1)C(F)(F)F)c1[nH]c(C)c(C(=O)C)c1C	0.0	17.0	not tested	not tested	0.0	17.0
SN00772093	SC011213	O=C(Nc1ccccc1)N1CCCC1CN1CCCCC1	0.0	16.8	not tested	not tested	0.0	16.8
SN00772629	SC013389	CCCCOC(=O)c1ccc(cc1)NC(=O)c1cc2c(n1CCC)n(C)c(=O)n(C)c2=O	0.0	16.7	not tested	not tested	0.0	16.7
SN00777763	SC007582	CCC(=O)N1CCCC1C1CCCN1Cc1cn2c(C)cccc2n1	0.0	16.6	not tested	not tested	0.0	16.6
SN00782105	SC015739	O=C(Nc1ccc(cc1)N(=O)=O)NC1C2CCCOC2C1(C)C	0.0	16.3	not tested	not tested	0.0	16.3
SN00774604	SC000942	O=C(N1CCCC1Cn1cccn1)c1cnn(c2ccccc2)c1C	0.0	16.2	not tested	not tested	0.0	16.2
SN00774074	SC000758	Cn1ncc(c1)CN1C(=O)NC(C)(c2ccco2)C1=O	0.0	16.2	not tested	not tested	0.0	16.2
SN00795048	CL8587	Cc1ccc(cc1)c1cc2nc(C)cc(N3CCC(CC3)C(=O)NCCCN3CCCC3)n2n1	0.0	16.1	not tested	not tested	0.0	16.1
SN00770169	SC001552	O=C1NCCN(C1)c1ccc(cn1)NC(=O)c1[nH]cc(c1)C(=O)C	0.0	16.1	not tested	not tested	0.0	16.1
SN00784246	CL1106	CCCNC(=O)[C@@H]1[C@@H]2C=C[C@]3(O2)CN(Cc2ccccc2Cl)C(=O)[C@H]13	0.0	16.0	not tested	not tested	0.0	16.0
SN00771283	SC008058	O=C1NC(C)(c2ccco2)C(=O)N1NC(=O)c1sc2ccccc2c1Cl	0.0	16.0	not tested	not tested	0.0	16.0
SN00797123	CL8861A	O=C(NCc1cccc(c1)C(F)(F)F)C1CCCN(C1)c1nccnc1Oc1ccccc1	0.0	15.6	not tested	not tested	0.0	15.6
SN00783221	SC014022	O=C(NCC1OCC2CCCN2C1)NC(C)Cc1c(C)nn(C)c1C	0.0	15.6	not tested	not tested	0.0	15.6
SN00799308	CL9937	O=C(NC(C)(C)C)c1n[nH]c(c1)c1cccnc1	0.0	15.5	not tested	not tested	0.0	15.5
SN00769730	SC000926	O=C(C1CCCNC1)N1CCN(CC1)C(=O)OC(C)(C)C	0.0	15.1	not tested	not tested	0.0	15.1
SN00791532	CL6662	CCCNC(=O)N1Cc2c3CCCCc3sc2n2cccc2C1CC	0.0	15.0	not tested	not tested	0.0	15.0
SN00776616	SC005539	CCN(CC)CCN1C(c2ccc(C)o2)C(=C(O)C1=O)C(=O)c1cccs1	0.0	15.0	not tested	not tested	0.0	15.0
SN00779632	SC010215	CCc1nc(C)c2c(n1)sc(C(=O)N1CCN(CC1)Cc1ccc(OC)c(F)c1)c2C	0.0	14.8	not tested	not tested	0.0	14.8
SN00785390	CL8504	CC(C)CCNC(=O)C1(C)CCc2ccccc2C(=O)N1Cc1ccccc1F	0.0	14.6	not tested	not tested	0.0	14.6
SN00797220	CL9330A	CCC(=O)Nc1cccc(c1)c1noc(n1)C1CCCN(C1)C(=O)c1ccc(C)c(F)c1	0.0	14.6	not tested	not tested	0.0	14.6
SN00776642	SC005539	CN(C)CCCN1C(=O)C(=C(C1c1ccco1)C(=O)c1cc(Cl)ccc1O)O	0.0	14.6	not tested	not tested	0.0	14.6
SN00783283	SC015319	Cc1ncccc1CNCC1OCCN(C1)C1CC1	0.0	14.5	not tested	not tested	0.0	14.5
SN00790822	CL5098	CCN1CCN(CCCNC(=O)c2nc(Nc3ccc(OC)cc3)c3ccoc3c2)CC1	0.0	14.5	not tested	not tested	0.0	14.5
SN00782980	SC009940	O=C(N1CCCC(CN2CCOCC2)C1)C1(C)CC1(Cl)Cl	0.0	14.3	not tested	not tested	0.0	14.3
SN00796798	CL9208B	Fc1cccc(c1)CS(=O)(=O)N1CCc2n(C)nc(c3onc(C)n3)c2C1	0.0	14.3	not tested	not tested	0.0	14.3
SN00774391	SC000873	O=C(N1CCN(CC1)C(=O)c1cc2c(C)nn(Cc3ccccc3)c2s1)N1CCCCC1	0.0	14.0	not tested	not tested	0.0	14.0
SN00785687	CL6990	CCC(=O)N1N=C2C3CCN(CC3)[C@@H]2[C@@H]1c1cccc(OC)c1OC	0.0	13.8	not tested	not tested	0.0	13.8
SN00795130	CL7780	COc1ccc(cc1)CC(=O)N1CCN(CC1)Cc1nc2cc(ccc2n1C)C(=O)N1CCCC1	0.0	13.8	not tested	not tested	0.0	13.8
SN00796116	CL8537A	COc1ccc(cc1)n1c(CN2CCN(CC2)S(=O)(=O)c2cc(C)ccc2C)nc2cccnc12	0.0	13.7	not tested	not tested	0.0	13.7
SN00807918	SC015099	Cc1ncc2CCN(Cc2n1)C(C)c1nnc(o1)c1ccccc1	0.0	13.7	not tested	not tested	0.0	13.7
SN00799309	CL9937	O=C(N1CCCC1)c1n[nH]c(c1)c1cccnc1	0.0	13.7	not tested	not tested	0.0	13.7
SN00793355	CL3957	Cc1ccc(cc1)c1nc2ncccn2c1NC(=O)c1ccc(cc1)C(F)(F)F	0.0	13.4	not tested	not tested	0.0	13.4
SN00785169	CL5800	CCCN1C(=O)c2cc(ccc2C1Nc1ccc(F)cc1)C(=O)Nc1ccc(F)cc1	0.0	13.3	not tested	not tested	0.0	13.3
SN00802145	CM2338A	CCC(=O)N1CC(C(=O)NCCN(C)C)C2(CCOCC2)C1	0.0	13.1	not tested	not tested	0.0	13.1
SN00771276	SC008058	O=C(CN1C(=O)NC(C)(c2ccco2)C1=O)c1ccccc1	0.0	12.8	not tested	not tested	0.0	12.8
SN00792852	CL7285A	CSc1ccc(CCNC(=O)c2ccc3c(c2)nc2COCCn32)cc1	0.0	12.8	not tested	not tested	0.0	12.8
SN00792562	CL4121	Cc1cccc(CN2CCC3(CC2)N=C(c2ccc(C)c(C)c2)C(=O)N3)c1	0.0	12.3	not tested	not tested	0.0	12.3
SN00778360	SC008047	CSc1ccc(cc1)c1csc(CN2CCN(CC2)C(=O)C)n1	0.0	12.1	not tested	not tested	0.0	12.1

Compound ID	Scaffold	Smiles	% Inhibition n=1		% Inhibition n=2		Average % Inhibition	
			24h	48h	24h	48h	24h	48h
SN00783856	CL2892	CCCN(c1ccccc1C(=O)O)S(=O)(=O)c1ccc2[nH]c(=O)[nH]c2c1	0.0	12.1	not tested	not tested	0.0	12.1
SN00776389	SC003225	CCc1nnc(s1)NC(=O)c1ccsc1n1c(C)ccc1C	0.0	11.7	not tested	not tested	0.0	11.7
SN00798922	CL9736	CCCC(=O)Nc1ccc2CC(N(Cc2c1)C(=O)Cc1ccccc1C)C(=O)N(C)C	0.0	11.7	not tested	not tested	0.0	11.7
SN00790551	CL6286	CSc1ccc(cc1)CNC(=O)C1CCN(CC1)S(=O)(=O)c1ccc(o1)c1[nH]ncc1	0.0	11.7	not tested	not tested	0.0	11.7
SN00793072	CL4660A	O=C(C1CCN(CC1)c1onc(n1)c1ccc(cc1)C(C)C)N1CCN(CC1)C1CCCCC1	0.0	11.6	not tested	not tested	0.0	11.6
SN00780682	SC011809	O=C(NCCCC(=O)N1CCCN(CC1)Cc1noc(n1)C(C)(C)C)NC1CCCCC1	0.0	11.6	not tested	not tested	0.0	11.6
SN00784605	CL2039	Cc1ccc(cc1)CNC(=O)C1(CC1)S(=O)(=O)c1ccc(Cl)cc1	0.0	11.5	not tested	not tested	0.0	11.5
SN00794326	CL5859	COc1ccccc1NC(=O)c1cn2CCc3cc(Br)cc(c1=O)c23	0.0	11.4	not tested	not tested	0.0	11.4
SN00799967	CM0323	CNc1cccnc1OC1CCN(C1)C(=O)CCc1nc2ccccc2[nH]1	0.0	11.3	not tested	not tested	0.0	11.3
SN00779695	SC010300	Clc1ccc(cc1)S(=O)(=O)N1CCC(CC1)c1onc(n1)c1c(Cl)cccc1Cl	0.0	11.2	not tested	not tested	0.0	11.2
SN00786221	CL5430	Cc1ccc(cc1)CNc1nc(nn1C(=O)C(C)C)c1ccco1	0.0	11.1	not tested	not tested	0.0	11.1
SN00784417	CL4264	Cc1ccc2c([nH]cc(C(=O)N3CCSCC3)c2=O)n1	0.0	11.0	not tested	not tested	0.0	11.0
SN00771476	SC008255	CCn1ccnc1CN1CCCN(CC1)C(=O)c1cnc(C)cn1	0.0	10.9	not tested	not tested	0.0	10.9
SN00788031	CL3961	CC1Cc2cc(ccc2N1C(=O)C1CCC1)S(=O)(=O)N1CCn2c3ccccc3nc12	0.0	10.8	not tested	not tested	0.0	10.8
SN00773025	SC014747	COc1ccc2CN(CCCc2c1)C(=O)c1nnc2ccccc2c1N	0.0	10.7	not tested	not tested	0.0	10.7
SN00772279	SC012158	CC(N1CCN(CC1)c1n(C)nc(C)c1N(=O)=O)c1nc2ccccc2s1	0.0	10.6	not tested	not tested	0.0	10.6
SN00774390	SC000873	Brc1ccc(cc1)S(=O)(=O)N1CCN(CC1)C(=O)c1sc2n(C)nc(C)c2c1	0.0	10.6	not tested	not tested	0.0	10.6
SN00775816	SC002885	O=c1cc(COC(=O)C2CCCN2S(=O)(=O)C)nc2sc3CCCCc3n12	0.0	10.4	not tested	not tested	0.0	10.4
SN00785371	CL6673B	CCN1c2cc(C)ccc2n2cccc2C21CCN(CC2)S(=O)(=O)c1ccc(C)cc1	0.0	10.4	not tested	not tested	0.0	10.4
SN00791557	CL6662A	CCOC(=O)c1ccccc1NC(=O)N1Cc2c3CCN(C)Cc3sc2n2cccc2C1CC	0.0	10.1	not tested	not tested	0.0	10.1
SN00771296	SC008104	CCc1ccccc1Nc1nc(CN2CCCN(CC2)S(=O)(=O)c2ccc(C)cc2)nc(N)n1	0.0	10.0	not tested	not tested	0.0	10.0
SN00773766	SC000244	CCn1c(CN2Cc3ccccc3C[C@H]2C(=O)N)nc2cc(ccc12)S(=O)(=O)N1CCCCC1	0.0	10.0	not tested	not tested	0.0	10.0
SN00788706	CL4247	COc1cc2ccccc2cc1C(=O)Nc1sc2CN(CCc2n1)Cc1ccccc1	0.0	9.9	not tested	not tested	0.0	9.9
SN00791505	CL6497	COc1ccc(OC)c(CNC(=O)c2ccc(s2)N2CCOc3ccccc23)c1	0.0	9.9	not tested	not tested	0.0	9.9
SN00797311	CL9350	CCCn1nccc1c1csc(c1)S(=O)(=O)NC(C)C	0.0	9.7	not tested	not tested	0.0	9.7
SN00788368	CL2031	O=C(NCCCN1CCN(CC1)c1ccccc1)c1noc2CCCCCc12	0.0	9.7	not tested	not tested	0.0	9.7
SN00780038	SC010798	O=C1CCCN1CC1CCCCN1c1cc(Cl)c(cc1N(=O)=O)C(F)(F)F	0.0	9.6	not tested	not tested	0.0	9.6
SN00799688	CM0489	CSc1ccc(cc1)NC(=O)c1cn(C)c2ncccc12	0.0	9.5	not tested	not tested	0.0	9.5
SN00776806	SC005969	O=C(Nc1nnc([nH]1)c1ccccn1)C1CC(=O)N(C1)CC(F)(F)F	0.0	9.5	not tested	not tested	0.0	9.5
SN00774731	SC000972	O=C(Cc1ccccc1N(=O)=O)N1CCN(CC1)C(=O)c1ccc2OCOc2c1	0.0	9.4	not tested	not tested	0.0	9.4
SN00780071	SC010810	O=C(COc1ccccc1c1ccccc1)NNC(=O)C1CC(=O)N(C)C1c1cccnc1	0.0	9.4	not tested	not tested	0.0	9.4
SN00795250	CL7767A	Cc1cccc(c1)c1onc(n1)c1ccc(cc1)NS(=O)(=O)C	0.0	9.2	not tested	not tested	0.0	9.2
SN00801778	CM1696	Fc1ccc(cc1)c1onc(n1)C1CC(=O)N(C1)c1ccc(F)cc1	0.0	9.0	not tested	not tested	0.0	9.0
SN00782098	SC015739	Cc1ncc(s1)C(=O)NC1C2CCCOC2C1(C)C	0.0	9.0	not tested	not tested	0.0	9.0
SN00797750	CL8534	NC(=O)C1CCN(CC1)C(=O)c1snc(C(=O)NCc2ccccc2)c1N	0.0	8.9	not tested	not tested	0.0	8.9
SN00800192	CL4868B	CCN1c2cc(Cl)ccc2C(=O)NC21CCN(CC2)C(=O)Nc1ccc2OCOc2c1	0.0	8.9	not tested	not tested	0.0	8.9
SN00797099	CL9311	CCc1ccccc1NC(=O)c1cnn2c1NC(=CC2c1cccs1)C(=O)O	0.0	8.9	not tested	not tested	0.0	8.9
SN00785479	CL3765	CCC1CCCCN1C(=O)Cn1c(cc2occc12)C(=O)OC	0.0	8.8	not tested	not tested	0.0	8.8
SN00774707	SC000971	O=C(N1CCN(CC1)C(=O)C1CCCC1)c1nccnc1	0.0	8.7	not tested	not tested	0.0	8.7
SN00787599	CL5111A	CC(C(=O)NC1CCCCCC1)n1c(=O)c2cccn2c2cccnc12	0.0	8.7	not tested	not tested	0.0	8.7
SN00798930	CL9736	CC(=O)Nc1ccc2CC(C(=O)N3CCCCC3)N(Cc2c1)C(=O)C1CCCCC1	0.0	8.7	not tested	not tested	0.0	8.7
SN00784739	CL4006	O=C(NCCc1ccccc1)c1ccc(cc1)Nc1nc2ccccc2n2nnnc12	0.0	8.5	not tested	not tested	0.0	8.5
SN00781474	SC014211	Cc1csc(n1)C1CCN(CC1)C(=O)c1ccccc1N(=O)=O	0.0	8.2	not tested	not tested	0.0	8.2
SN00794287	CL9522	CSc1ccccc1NC(=O)C1CCN(c2ccccc2O1)S(=O)(=O)C	0.0	8.2	not tested	not tested	0.0	8.2
SN00775416	SC001437	O=c1c(Br)c(cnn1c1ccccc1)N1CCCCC1	0.0	8.1	not tested	not tested	0.0	8.1
SN00794417	CL2352	OCCNc1ncnc2c1cnn2c1cc(Cl)ccc1C	0.0	8.1	not tested	not tested	0.0	8.1

Compound ID	Scaffold	Smiles	% Inhibition n=1		% Inhibition n=2		Average % Inhibition	
			24h	48h	24h	48h	24h	48h
SN00775270	SC001221	Cc1cc(C)n2nc(nc2n1)C(=O)N1CCCC(C1)n1cncn1	0.0	8.1	not tested	not tested	0.0	8.1
SN00792341	CL6666	CCOC(=O)CNC(=O)N1Cc2c(C)nn(c3ccccc3)c2n2cccc2C1c1cccc(F)c1	0.0	8.0	not tested	not tested	0.0	8.0
SN00799502	CL2046	Fc1ccc(cc1)NC(=O)C1=NN(CC(=O)Nc2ccc(C)c(F)c2)C(=O)CC1	0.0	8.0	not tested	not tested	0.0	8.0
SN00777799	SC007630	Clc1ccc(SCCNC(=O)c2[nH]cc(c2)S(=O)(=O)N2CCOCC2)cc1	0.0	7.9	not tested	not tested	0.0	7.9
SN00793589	CL3702A	O=C(NCc1ccc2c(CCN2C(=O)c2ccccc2)c1)C1CCN(CC1)S(=O)(=O)N1CCCC1	0.0	7.9	not tested	not tested	0.0	7.9
SN00782611	SC013678	O=C(CCc1cnn(C)c1)N1CCCC(C1)c1n[nH]c(C)c1	0.0	7.8	not tested	not tested	0.0	7.8
SN00772546	SC013240	Cc1ccc(cc1)S(=O)(=O)N1CCC[C@H]1C(=O)N1CCN(CC1)CC(=O)Nc1cccc(C)c1C	0.0	7.6	not tested	not tested	0.0	7.6
SN00781077	SC012973	O=C(C1CCC=CC1)N1CCN(CC1)S(=O)(=O)c1c[nH]c2ncccc12	0.0	7.5	not tested	not tested	0.0	7.5
SN00784337	CL3544	O=c1n(Cc2cc(=O)n3ccccc3n2)c2ncccc2c(=O)n1CCc1ccccc1	0.0	7.3	not tested	not tested	0.0	7.3
SN00791654	CL6154	O=C(N1CCN(CC1)c1ccccn1)c1noc2ncnc(N3CCCC3)c12	0.0	7.3	not tested	not tested	0.0	7.3
SN00786536	CL8267	O=c1[nH]c2nc3CC(C)(C)OCc3cc2c(=O)n1Cc1ccco1	0.0	7.3	not tested	not tested	0.0	7.3
SN00796635	CL9305	Cc1ccc(cc1)c1noc(n1)c1ccc(nc1)N1CCN(CC1)Cc1ccc2OCOc2c1	0.0	7.2	not tested	not tested	0.0	7.2
SN00777047	SC006676	Nc1scc(n1)c1ccc2c(CCCN2S(=O)(=O)C)c1	0.0	7.2	not tested	not tested	0.0	7.2
SN00782090	SC015667	CCOc1cc(F)ccc1NC(=O)N1CCCC(C1)N1CCCC1=O	0.0	7.0	not tested	not tested	0.0	7.0
SN00790923	CL8114A	CCNC(=O)N1CCCC(C1)c1cccc(c1)C(=O)NCCC1=CCCCC1	0.0	6.9	not tested	not tested	0.0	6.9
SN00799076	CL9740B	COc1ccc(cc1)S(=O)(=O)N1CCc2nc(ncc2C1)c1ccccc1	0.0	6.9	not tested	not tested	0.0	6.9
SN00776656	SC005543	CCOC(=O)c1sc(nc1C)N1C(c2ccc(OC)cc2)C(=C(O)C1=O)C(=O)c1ccco1	0.0	6.9	not tested	not tested	0.0	6.9
SN00770616	SC004496	OC(COc1ccccc1)Cn1c(=O)c2ccccc2n2nc(C)cc12	0.0	6.9	not tested	not tested	0.0	6.9
SN00790421	CL7771A	COc1ccc(cc1)OCC(=O)N1CCN(Cc2ccc(C)cc2)c2ncccc12	0.0	6.7	not tested	not tested	0.0	6.7
SN00788861	CL3997A	O=C(CCc1nnc2n(CC(C)C)c(=O)c3sccc3n12)NC1CC1	0.0	6.7	not tested	not tested	0.0	6.7
SN00800945	CM2379A	O=C(Nc1ccc2CCCc2c1)N1C[C@H]2C[C@@H]1[C@H](C2)NC(=O)c1ccccc1	0.0	6.6	not tested	not tested	0.0	6.6
SN00793852	CL8308	CCc1onc(C)c1C1CCCN1C(=O)c1ccc2OCOc2c1	0.0	6.6	not tested	not tested	0.0	6.6
SN00794542	CL1790	COc1ccc(CCNC(=O)C2N(Cc3ccc(C)cc3)C(=O)c3ccccc23)cc1OC	0.0	6.3	not tested	not tested	0.0	6.3
SN00790813	CL8435	Cc1cccc(c1)N(C)S(=O)(=O)c1cc(sc1C)c1ccno1	0.0	6.1	not tested	not tested	0.0	6.1
SN00772979	SC014697	Cc1cccc(c1)c1noc(CN2CC(C2)n2ncc(C)c2)n1	0.0	6.1	not tested	not tested	0.0	6.1
SN00776157	SC003106	Cc1nn(c(c1)NC(=O)c1ccc(cc1)N(=O)=O)c1nc2ccccc2[nH]1	0.0	6.0	not tested	not tested	0.0	6.0
SN00774586	SC000927	C/C(=C/C(=O)N1CCN(CC1)C(=O)c1ccc2[nH]cnc2c1)/C1CC1	0.0	5.9	not tested	not tested	0.0	5.9
SN00791107	CL8463	CC(C)n1c2nn(cc2c(=O)n(C(C)C)c1=O)S(=O)(=O)c1ccccc1	0.0	5.9	not tested	not tested	0.0	5.9
SN00798376	CL9600	Cc1onc(n1)c1ccc2c(c1)n(CC(=O)NC1CCN(CC1)Cc1ccccc1)c(=O)n2C(C)C	0.0	5.7	not tested	not tested	0.0	5.7
SN00793591	CL3702A	O=C(c1ccccc1)N1CCc2cc(ccc12)CNC(=O)c1n[nH]c2c1CCc1ccccc21	0.0	5.7	not tested	not tested	0.0	5.7
SN00789753	CL7900	Cc1ccc(cc1)S(=O)(=O)NC(C)c1onc(n1)c1cccc(c1)C(=O)N1CCCC1	0.0	5.7	not tested	not tested	0.0	5.7
SN00784282	CL1215	CC(C)CCNC(=O)C1CCCCN1S(=O)(=O)c1ccc(F)cc1	0.0	5.5	not tested	not tested	0.0	5.5
SN00779590	SC010179	CC1COCCN1C(=O)c1nnn(c2cccc3CN(CCc23)Cc2ccccc2)c1C	0.0	5.5	not tested	not tested	0.0	5.5
SN00802642	CL1367	CN(Cc1ccco1)C(=O)c1nn(c(CCc2ccccc2)n1)c1ccccc1C	0.0	5.4	not tested	not tested	0.0	5.4
SN00802675	CL5459A	CCOC(=O)CC(=O)Nc1ccc(cc1)c1onc2c1CCc1cc(OC)ccc21	0.0	5.3	not tested	not tested	0.0	5.3
SN00791744	CL6846	Clc1cccc(c1)N1CCN(CC1)C(=O)CN1CCCn2nc(C)cc12	0.0	5.3	not tested	not tested	0.0	5.3
SN00789919	CL7893	Cc1ccc(cc1)NS(=O)(=O)c1ccc(s1)c1nnc(C)o1	0.0	5.1	not tested	not tested	0.0	5.1
SN00783957	CL6690	COc1ccc(c(OC)c1)C1NC(Cc2c1[nH]c1ccccc21)C(=O)O	0.0	5.0	not tested	not tested	0.0	5.0
SN00776590	SC005367	O=C(Nc1ccc2OCOc2c1)C1CCN(CC1)Cc1nnnn1c1ccccc1	0.0	4.9	not tested	not tested	0.0	4.9
SN00798039	CL9457	CCc1ccc(cc1)n1ccc(=O)c(n1)c1onc(n1)c1cccc(Cl)c1	0.0	4.9	not tested	not tested	0.0	4.9
SN00798018	CL9455	COc1cccc(c1)Cn1nc2n(cccc2S(=O)(=O)N2CCOCC2)c1=O	0.0	4.9	not tested	not tested	0.0	4.9
SN00800001	CM0322	COCC(=O)N1CCC(CC1)Oc1ncccc1C(=O)N(C)C	0.0	4.8	not tested	not tested	0.0	4.8
SN00784930	CL3315	CCCCNC(=O)c1cccc(c1)N1CCCC1=O	0.0	4.7	not tested	not tested	0.0	4.7
SN00776554	SC005037	CC1OC(C)CN(C1)c1nc2n(C)c(=O)[nH]c(=O)c2n1CC(=O)O	0.0	4.6	not tested	not tested	0.0	4.6
SN00775052	SC001092	Cc1n[nH]c(c1)C(=O)N1CCC(CC1)Oc1ccccc1	0.0	4.5	not tested	not tested	0.0	4.5
SN00801593	CM3040	CNC(=O)N1CCC(C1)C1CCN(CC1)C(=O)c1ccncc1	0.0	4.5	not tested	not tested	0.0	4.5

Compound ID	Scaffold	Smiles	% Inhibition n=1		% Inhibition n=2		Average % Inhibition	
			24h	48h	24h	48h	24h	48h
SN00802933	CL6492	COc1ccc(CCNC(=O)c2sc(nc2C)c2c(F)cccc2OC)cc1	0.0	4.5	not tested	not tested	0.0	4.5
SN00786364	CL7376	COc1ccc(cc1)OCC(=O)N1CCCC(C1)c1nc2ccccc2[nH]1	0.0	4.5	not tested	not tested	0.0	4.5
SN00794088	CL3544A	CCCCNC(=O)Cn1c(=O)c2c(C)cc(C)nc2n(c2ccccc2)c1=O	0.0	4.4	not tested	not tested	0.0	4.4
SN00771045	SC007571	Clc1ccc(cc1)c1oc(CN2CCC(Nc3ccccc3)C2)nc1C	0.0	4.4	not tested	not tested	0.0	4.4
SN00787653	CL5161	Cc1ccc(cc1)CNC(=O)C1(C)Cn2c3ccccc3nc2C(=O)N1Cc1cccc(C)c1	0.0	4.3	not tested	not tested	0.0	4.3
SN00787611	CL5111B	O=C(CCn1c(=O)c2cccn2c2cccnc12)NC1CCCC1	0.0	4.3	not tested	not tested	0.0	4.3
SN00774994	SC001031	O=C(N1CCCC(C1)c1nncn1C)C1CCCN(C1)c1ncccc1C(F)(F)F	0.0	4.2	not tested	not tested	0.0	4.2
SN00802895	CL6511	COC(=O)c1cc2c(ccn(CC3CCCO3)c2=O)nc1C	0.0	4.2	not tested	not tested	0.0	4.2
SN00781067	SC012765	O=C1NC(C)(C)C(=O)N1CCCN1CCOc2ccccc2C1	0.0	4.2	not tested	not tested	0.0	4.2
SN00801825	CM0270	O=C(N1CCOCC1)c1ccc2c(c1)n(Cc1ccccc1)c(=O)c1ccnn21	0.0	4.0	not tested	not tested	0.0	4.0
SN00788837	CL7488	Cc1cccc(c1)/N=C(/NC(=O)c1ccccc1)\N1CCN(CC1)c1ccccc1	0.0	3.9	not tested	not tested	0.0	3.9
SN00779484	SC010036	CCOc1ccc(cc1OCC)S(=O)(=O)N1CCN(CC1)S(=O)(=O)c1sc(=O)[nH]c1C	0.0	3.9	not tested	not tested	0.0	3.9
SN00775033	SC001048	COc1ccc(cc1)c1noc(CN2CCN(CC2)C(=O)c2nn(nc2C)c2cccc(Cl)c2)n1	0.0	3.9	not tested	not tested	0.0	3.9
SN00786118	CL5015A	CCCn1c(Cn2ccnc2C)nc2cc(ccc12)NC(=O)c1ccco1	0.0	3.8	not tested	not tested	0.0	3.8
SN00782631	SC014697	c1ccc(cc1)OC1CN(C1)Cc1onc(n1)C1CC1	0.0	3.8	not tested	not tested	0.0	3.8
SN00799257	CL4844	COc1cc(ccc1OC)C(=O)Nc1cnn(C)c(=O)c1N1CCOCC1	0.0	3.6	not tested	not tested	0.0	3.6
SN00793436	CL6945A	COc1ccc(cc1)c1nn2c(n1)sc(CCNC(=O)C(C)(C)C)c2C	0.0	3.5	not tested	not tested	0.0	3.5
SN00783265	SC015097	c1ncc2CCN(Cc3onc(n3)C3CC3)Cc2n1	0.0	3.1	not tested	not tested	0.0	3.1
SN00784184	CL5508A	CCN(c1ccccc1)C(=O)Cn1cnc2onc(c3ccc(Cl)cc3)c2c1=O	0.0	3.1	not tested	not tested	0.0	3.1
SN00777609	SC007544	OC(COc1ccccc1)CN1C(=O)CC2(CCCC2)C1=O	0.0	3.0	not tested	not tested	0.0	3.0
SN00799674	CM0194	CN1CCC(CC1)NC(=O)c1ccc(cc1)n1ncc(CNC(=O)C2CCC2)c1	0.0	2.9	not tested	not tested	0.0	2.9
SN00782301	SC016338	O=C(Nc1n[nH]c(c1)C1CCOCC1)c1cn[nH]c1C	0.0	2.9	not tested	not tested	0.0	2.9
SN00771001	SC007517	Cc1cccc(c1)CC(=O)N1CCN(CC1)C(=O)c1c[nH]c(=O)[nH]1	0.0	2.9	not tested	not tested	0.0	2.9
SN00774943	SC000998	O=c1c2cccc3cccc(c23)n1CN1CCC(CC1)c1nc2ccccc2o1	0.0	2.8	not tested	not tested	0.0	2.8
SN00794493	CL3765	Fc1ccc(cc1)c1nnc(N)s1	0.0	2.8	not tested	not tested	0.0	2.8
SN00793092	CL5923	O=C(NCc1n[nH]c(=O)c2ccccc12)c1ccc(cc1)S(=O)(=O)N1CCOCC1	0.0	2.8	not tested	not tested	0.0	2.8
SN00785011	CL2764	Clc1ccc(CCNC(=O)C2CCCN(C2)S(=O)(=O)c2c(C)nn(C)c2C)cc1	0.0	2.8	not tested	not tested	0.0	2.8
SN00788008	CL3220A	COc1ccc(cc1)CC(=O)N1CCN(CC1)C(=O)c1[nH]nc(c1)C1CC1	0.0	2.8	not tested	not tested	0.0	2.8
SN00785085	CL2596	Cc1ccc(cc1)n1nc(C(=O)NC2CCCC2)c2c(c1=O)n(C)c1ccccc21	0.0	2.8	not tested	not tested	0.0	2.8
SN00784731	CL1162B	CCc1nn2c(cc(C)nc2n1)Nc1cc(C)ccc1C	0.0	2.8	not tested	not tested	0.0	2.8
SN00784307	CL0892	Clc1ccc(cc1)N1CCN(CC1)C(=O)Cc1ccc(cc1)n1c(=O)[nH]c2ccsc2c1=O	0.0	2.7	not tested	not tested	0.0	2.7
SN00801973	CM1028	O=C(Nc1ccccc1)c1nc2csc(C)c2c(n1)N1CCSCC1	0.0	2.7	not tested	not tested	0.0	2.7
SN00792021	CL7144A	Clc1ccc(NS(=O)(=O)c2cc(ccc2C)c2cnc(C)o2)c(F)c1	0.0	2.6	not tested	not tested	0.0	2.6
SN00782125	SC015928	c1ccc(cc1)N1CCC(CNc2nccn3cnnc23)C1	0.0	2.6	not tested	not tested	0.0	2.6
SN00802702	CL2849	CCCCC(O)CN1CCN(CC1)Cc1occ(n1)C(=O)N1CCCC(C1)C(=O)N(CC)CC	0.0	2.5	not tested	not tested	0.0	2.5
SN00794841	CL8689	Fc1ccc(cc1)CNC(=O)c1sc(nc1C)c1nnn(c2ccccc2)c1C	0.0	2.5	not tested	not tested	0.0	2.5
SN00793666	CL7067C	Fc1cccc(c1)NC(=O)N1CCC(CC1)N1CCN(Cc2cccc(F)c2)C(=O)C1=O	0.0	2.5	not tested	not tested	0.0	2.5
SN00777237	SC006854	CC(C)c1c(cnn1c1ccccc1)C(=O)Nc1sc(c(C)n1)c1nccn1C	0.0	2.5	not tested	not tested	0.0	2.5
SN00781678	SC014751	CCN1C(=O)C2CN(CCN2C1=O)C(=O)c1ccc2[nH]c(C)c(C)c2c1	0.0	2.4	not tested	not tested	0.0	2.4
SN00789851	CL7967A	Cc1cc(ccc1C)S(=O)(=O)N1CCc2n(C)nc(C(=O)NCc3ccc4OCOc4c3)c2C1	0.0	2.4	not tested	not tested	0.0	2.4
SN00794388	CL4855	O=C(NCCc1cccs1)CC(C)CC1=NS(=O)(=O)c2ccccc2N1	0.0	2.4	not tested	not tested	0.0	2.4
SN00799207	CL9885	O=C(COc1ccccc1)N1CCC(CC1)Cc1nnc(o1)c1ccccc1	0.0	2.3	not tested	not tested	0.0	2.3
SN00794962	CL8934	CCOC(=O)c1ccc(cc1)S(=O)(=O)Nc1cccc(c1)c1nnc(o1)c1ccccc1	0.0	2.3	not tested	not tested	0.0	2.3
SN00788003	CL3222	CCCS(=O)(=O)N1CCN(CC1)C(=O)c1[nH]nc(c1)c1ccc(Br)cc1	0.0	2.3	not tested	not tested	0.0	2.3
SN00788258	CL4805B	Fc1ccc(cc1)CNC(=O)c1nn2CC(C)(C(=O)NCc3ccc(F)cc3)N(C)C(=O)c2c1	0.0	2.3	not tested	not tested	0.0	2.3
SN00782203	SC016288	Fc1ccc(SCCC(=O)N2CCC(CC2)C2(C)NC(=O)NC2=O)cc1	0.0	2.1	not tested	not tested	0.0	2.1

Compound ID	Scaffold	Smiles	% Inhibition n=1		% Inhibition n=2		Average % Inhibition	
			24h	48h	24h	48h	24h	48h
SN00788841	CL7488	O=CN1CCN(CC1)/C(=N\c1ccccc1C)/NC(=O)c1ccccc1	0.0	2.1	not tested	not tested	0.0	2.1
SN00770052	SC001432	c1ccc(cc1)n1nnnc1N1CCN(CC1)Cc1nc2ccccc2s1	0.0	2.0	not tested	not tested	0.0	2.0
SN00786889	CL9402A	O=C(Nc1ccccc1)Cc1c(C)n(C)c2nc(nn2c1=O)c1ccccc1	0.0	1.9	not tested	not tested	0.0	1.9
SN00800999	CM2380B	O=C(N1C[C@@H]2C[C@H]1C[C@@H]2NS(=O)(=O)c1ccccc1)c1n[nH]c2ccccc12	0.0	1.8	not tested	not tested	0.0	1.8
SN00797278	CL9349	CCc1noc(c1)c1csc(c1)S(=O)(=O)N(C)c1ccc(C)c(C)c1	0.0	1.7	not tested	not tested	0.0	1.7
SN00782741	SC000972	CN1CCN(C(C1)c1ccccc1)C(=O)c1ccc2OCOc2c1	0.0	1.7	not tested	not tested	0.0	1.7
SN00788972	CL5809	CCc1cccc(c1)NS(=O)(=O)c1cc(ccc1C)c1c(C)noc1C	0.0	1.7	not tested	not tested	0.0	1.7
SN00799461	CL4071	COc1ccc(NC(=O)c2cnc3c(c2)c(=O)n(C)c(=O)n3c2ccccc2)cc1Cl	0.0	1.6	not tested	not tested	0.0	1.6
SN00776087	SC003030	Fc1cc(F)cc(c1)NC(=O)C1CCCCN1Cc1ccno1	0.0	1.6	not tested	not tested	0.0	1.6
SN00800219	CL8338	COc1ccc(cc1)NS(=O)(=O)c1cn2c(CC)n[nH]c(=O)c2c1	0.0	1.6	not tested	not tested	0.0	1.6
SN00790978	CL8494	COc1cc(OC)cc(c1)CC(=O)N1CCc2cc(ccc12)c1noc(C)n1	0.0	1.6	not tested	not tested	0.0	1.6
SN00782988	SC009941	CCOc1ncccc1NC(=O)N1CCC(CC1)CN1CCCC1	0.0	1.6	not tested	not tested	0.0	1.6
SN00791062	CL8457	COc1ccc(cc1)n1nnc(c2scc(n2)c2ccccc2C)c1N	0.0	1.5	not tested	not tested	0.0	1.5
SN00777678	SC007572	CCc1onc(n1)C1CCCN1Cc1c(C)noc1C	0.0	1.5	not tested	not tested	0.0	1.5
SN00784197	CL1042	CCN(CC)CCNC(=O)NC1(Oc2ccccc2O1)C(F)(F)F	0.0	1.5	not tested	not tested	0.0	1.5
SN00786116	CL5015A	CCCn1c(Cn2ccnc2C)nc2cc(ccc12)NC(=O)c1cc(OC)c(OC)c(OC)c1	0.0	1.5	not tested	not tested	0.0	1.5
SN00788520	CL6065	CCOc1ccc(cc1)N1C(=O)CS(=O)(=O)C21C(=O)N(Cc1cccc(Cl)c1)c1ccccc21	0.0	1.4	not tested	not tested	0.0	1.4
SN00787631	CL5161	Clc1ccc(cc1)CN1C(=O)c2nc3ccccc3n2CC1(C)C(=O)NC1CCCCC1	0.0	1.4	not tested	not tested	0.0	1.4
SN00788760	CL6291	O=C(Nc1ccc(cc1)C(C)C)c1noc(c1)c1cccnc1	0.0	1.4	not tested	not tested	0.0	1.4
SN00778432	SC008196	NC(=O)CN1CCCN(CC1)c1nccc(n1)C(F)(F)F	0.0	1.4	not tested	not tested	0.0	1.4
SN00774764	SC000973	Cc1ccc(C)c(c1)S(=O)(=O)N1CCN(CC1)C(=O)c1c[nH]cc1c1ccccc1	0.0	1.3	not tested	not tested	0.0	1.3
SN00786722	CL7986	COc1ccc(OCCN2CC(=O)N(c3ccc(C)cc3)[C@@H]3CS(=O)(=O)C[C@H]23)cc1	0.0	1.3	not tested	not tested	0.0	1.3
SN00793632	CL2049	COCCCNC(=O)C1(C)CCC(=O)N1c1ccc(C)cc1C	0.0	1.3	not tested	not tested	0.0	1.3
SN00779856	SC010596	Cc1cc(C(=O)Nc2cc(Cl)c(N)c(Cl)c2)c2nc3CCCCCn3c2n1	0.0	1.3	not tested	not tested	0.0	1.3
SN00795807	CL4111	CN(C)S(=O)(=O)NCc1nc2cccnc2n1C1CCCCC1	0.0	1.2	not tested	not tested	0.0	1.2
SN00782986	SC009941	COCCCN1CCC(CC1)CN1CCCC1	0.0	1.2	not tested	not tested	0.0	1.2
SN00785329	CL3439	Cc1ccc(cc1)NC(=O)c1oc2ccccc2c1NC(=O)c1ccccc1	0.0	1.2	not tested	not tested	0.0	1.2
SN00782612	SC013680	Cc1cc(C)n(CC2CCNCC2)n1	0.0	1.1	not tested	not tested	0.0	1.1
SN00776743	SC005830	FC(F)(F)c1cccc(c1)n1ncc(c1)c1cnn(Cc2ccccc2)c1	0.0	1.1	not tested	not tested	0.0	1.1
SN00782783	SC002397	CC1OC(C)CN(C1)Cc1ncc(o1)c1ccc(F)cc1	0.0	1.1	not tested	not tested	0.0	1.1
SN00790915	CL8114A	CCOc1ccc(cc1)NC(=O)N1CCCC(C1)c1cccc(c1)C(=O)NCCc1ccco1	0.0	1.1	not tested	not tested	0.0	1.1
SN00784208	CL1042	COC(=O)c1ccccc1NC(=O)NC1(Oc2cc(Cl)ccc2O1)C(F)(F)F	0.0	1.0	not tested	not tested	0.0	1.0
SN00780452	SC011311	O=C1COc2cc(ccc2N1)C(=O)Nc1cnccc1n1cncc1	0.0	0.9	not tested	not tested	0.0	0.9
SN00800430	CM1389	O=C(N1CCN(CC1)Cc1ccccc1)c1nc(N2CCCCC2)c2cnn(C)c2n1	0.0	0.9	not tested	not tested	0.0	0.9
SN00777084	SC006680	CCOc1ccc(CCC2CCN(CC2)C(=O)c2[nH]cnc2)cc1	0.0	0.9	not tested	not tested	0.0	0.9
SN00770428	SC002445	COc1ccc(cc1)C1CCCN1Cc1[nH]c(=O)c2sc(cc2n1)c1ccc(OC)cc1	0.0	0.9	not tested	not tested	0.0	0.9
SN00800405	CM1209	COc1ccc(cc1)C(=O)NC1CCN(C1)c1[nH]nc(c1)c1cccs1	0.0	0.8	not tested	not tested	0.0	0.8
SN00794283	CL9522	O=C(NC1CC1)C1CCN(c2ccccc2O1)S(=O)(=O)C	0.0	0.8	not tested	not tested	0.0	0.8
SN00783850	CL7741	COc1ccc(cc1)NC(=O)CC1NCCNC1=O	0.0	0.7	not tested	not tested	0.0	0.7
SN00793881	CL8308A	CCCS(=O)(=O)N1CCCC1c1c(C)noc1CC	0.0	0.7	not tested	not tested	0.0	0.7
SN00798349	CL9596	Cc1c(C)n[nH]c1c1sc(C)c(c1)S(=O)(=O)N1CCc2ccccc2C1	0.0	0.7	not tested	not tested	0.0	0.7
SN00770777	SC006354	Clc1ccc(Cl)c(c1)S(=O)(=O)N1CCN=C1c1ccccc1	0.0	0.7	not tested	not tested	0.0	0.7
SN00785326	CL3417	CCOc1cc(cc(OCC)c1OCC)c1cc(N)n(n1)S(=O)(=O)c1ccc(C)cc1	0.0	0.6	not tested	not tested	0.0	0.6
SN00793354	CL3957	Cc1ccc(cc1)c1nc2ncccn2c1NC(=O)c1cccc(c1)C(F)(F)F	0.0	0.6	not tested	not tested	0.0	0.6
SN00802854	CL7567	CN1CCN(CC1)Cc1nc(Nc2ccc(cc2)Oc2ccccc2)nc(N)n1	0.0	0.6	not tested	not tested	0.0	0.6
SN00799836	CL7449	O=C(NCCC1=CCCCC1)CC1c2ncccc2C(=O)N1c1ccccc1	0.0	0.6	not tested	not tested	0.0	0.6

Compound ID	Scaffold	Smiles	% Inhibition n=1		% Inhibition n=2		Average % Inhibition	
			24h	48h	24h	48h	24h	48h
SN00785788	CL1392	c1cnc(nc1)N1CCN(CC1)c1ncnc2[nH]cnc12	0.0	0.6	not tested	not tested	0.0	0.6
SN00785094	CL2879	CCn1c(CN2CCN(CC2)Cc2ccccc2)nc2c1c(=O)n(C)c(=O)n2C	0.0	0.5	not tested	not tested	0.0	0.5
SN00791991	CL7144	COCCCNS(=O)(=O)c1ccc(cc1)c1cnc(C)o1	0.0	0.5	not tested	not tested	0.0	0.5
SN00777937	SC007716	Cc1ccc(cc1)S(=O)(=O)N1CCCN(CC1)Cc1onc(n1)c1ccccc1	0.0	0.5	not tested	not tested	0.0	0.5
SN00780288	SC011097	COC(=O)c1[nH]c(C)c(C(=O)C2=C(O)C(=O)N(CCCn3ccnc3)C2c2ccncc2)c1C	0.0	0.5	not tested	not tested	0.0	0.5
SN00782169	SC016049	CC(C)c1oc(nc1c1ccccc1)C1CCCN1S(=O)(=O)c1cn(C)c(c1)C(=O)N	0.0	0.5	not tested	not tested	0.0	0.5
SN00785231	CL3343	COc1cc(ccc1OC)NC(=O)c1onc(C)c1	0.0	0.4	not tested	not tested	0.0	0.4
SN00796804	CL9208B	CCCS(=O)(=O)N1CCc2n(C)nc(c3onc(n3)c3ccc(F)cc3)c2C1	0.0	0.4	not tested	not tested	0.0	0.4
SN00780522	SC011527	Clc1ccc(CCC(=O)N2CCCC(C2)c2nnc[nH]2)c(Cl)c1	0.0	0.4	not tested	not tested	0.0	0.4
SN00795455	CL3964	COc1ccc(Cl)cc1S(=O)(=O)Nc1ccc2nnc(C)n2n1	0.0	0.3	not tested	not tested	0.0	0.3
SN00792661	CL6390	Cc1cc(ccc1C)NS(=O)(=O)c1nc(C)n(C)c1	0.0	0.3	not tested	not tested	0.0	0.3
SN00788868	CL3997A	CCCn1c2nnc(CCC(=O)NCCc3ccccc3)n2c2ccsc2c1=O	0.0	0.3	not tested	not tested	0.0	0.3
SN00773982	SC000743	CCCC1NC(=O)N(Cc2nnc(o2)c2ccc(Br)o2)C1=O	0.0	0.3	not tested	not tested	0.0	0.3
SN00791672	CL6783	COc1ccccc1CNC(=O)c1noc(C)c1CN1CCOCC1	0.0	0.2	not tested	not tested	0.0	0.2
SN00773968	SC000734	O=c1n(cccc1C(F)(F)F)Cc1nnc(Cc2ccccc2)o1	0.0	0.2	not tested	not tested	0.0	0.2
SN00782248	SC016317	CCn1ncc(c1)C1OCCC1CNCc1nccn1CC	0.0	0.1	not tested	not tested	0.0	0.1
SN00773066	SC015169	O=C(N1CCn2c(nnc2C)C1)c1cnn(c2ccccc2)c1C	0.0	0.1	not tested	not tested	0.0	0.1
SN00782953	SC009216	CN1CCCC1C1CCCN1C(=O)c1cnc2c(cnn2C(C)C)c1	0.0	0.1	not tested	not tested	0.0	0.1
SN00782274	SC016324	O=C(NCc1ccco1)N1CC(C1)S(=O)(=O)N1CCN(CC1)C(=O)OC(C)(C)C	0.0	0.1	not tested	not tested	0.0	0.1
SN00769920	SC001340	O=C(Nc1scc(n1)c1ccncc1)c1cc(nc2ccccc12)C1CC1	0.0	0.1	not tested	not tested	0.0	0.1
SN00802307	CM1855	O=C1CC[C@@]2(CCCC[C@H]2N1C)C(=O)NC1CCCC1	0.0	0.1	not tested	not tested	0.0	0.1
SN00770125	SC001484	NC/C(=c/1\[nH]c2ccccc2n1C)/c1csc(n1)N1CCCCC1	0.0	0.1	not tested	not tested	0.0	0.1
SN00799435	CL9679	COCc1cc2nn(CC(=O)N(C)c3ccccc3)c(=O)n2c(n1)c1ccccc1	0.0	0.1	not tested	not tested	0.0	0.1
SN00789950	CL8380	COc1ccc(OC)c(c1)NC(=O)c1cn(nc1C)c1ccc(cc1)c1noc(C)n1	0.0	0.1	not tested	not tested	0.0	0.1
SN00800621	CM1426	CCN1CCCn2cc(cc2C1=O)S(=O)(=O)NCCCc1ccccc1	0.0	0.0	not tested	not tested	0.0	0.0
SN00774401	SC000896	O=C(Nc1cccc(c1)C(C)C)C1CCN(CC1)C(=O)c1cnoc1C	0.0	0.0	not tested	not tested	0.0	0.0
SN00783493	CL7525	O=N(=O)c1cccc(c1)C(=O)N1N=C2CCCCC2C1(O)C(F)(F)F	0.0	0.0	not tested	not tested	0.0	0.0
SN00793767	CL5102	COc1ccc(OC)cc1NC(=O)Cc1n(C)cc(C)c1C(=O)O	0.0	0.0	not tested	not tested	0.0	0.0
SN00794898	CL9106	Brc1ccc(cc1)c1noc(n1)c1cccn(CC(=O)N2CCCc3ccccc23)c1=O	0.0	0.0	not tested	not tested	0.0	0.0
SN00794923	CL8795	Cc1ccc(cc1)Cn1c(=O)c2ccccc2n(Cc2onc(n2)c2ccc(Cl)cc2)c1=O	0.0	0.0	not tested	not tested	0.0	0.0
SN00785384	CL8504	COc1cc(OC)cc(c1)N1C(=O)c2ccccc2CCC1(C)C(=O)NCCC(C)C	0.0	0.0	not tested	not tested	0.0	0.0
SN00782003	SC015304	O1CCC(CC1)n1nnnc1Sc1cnc2ccccc2n1	0.0	0.0	not tested	not tested	0.0	0.0
SN00782082	SC015667	CCCCS(=O)(=O)N1CCCC(C1)N1CCCC1=O	0.0	0.0	not tested	not tested	0.0	0.0
SN00781966	SC015250	Cc1cccc(c1)c1nnc(o1)C(C)N1CC(C1)n1nc(C)cc1C	0.0	0.0	not tested	not tested	0.0	0.0
SN00772249	SC012104	CCC(C)C(=O)N1CCCC1c1[nH]cc(n1)c1ccccc1Cl	0.0	0.0	not tested	not tested	0.0	0.0
SN00790279	CL7943A	Fc1ccc(cc1)C(=O)Nc1ccc2nc(cc(C(=O)O)c2c1)N1CCN(CC1)c1ccccc1	0.0	0.0	not tested	not tested	0.0	0.0
SN00782999	SC010158	O=C(NCc1nnc2CCCn12)C1CC(=O)N(Cc2ccccn2)C1	0.0	0.0	not tested	not tested	0.0	0.0
SN00784363	CL1530	Cc1ccc(cc1)c1nc(Cn2nnc(C(=O)NCc3ccc(F)cc3)c2N)c(C)o1	0.0	0.0	not tested	not tested	0.0	0.0
SN00773313	SC015442	CC(C)(C)c1onc(CN2CCC3(CC2)CC3c2nnc(o2)c2ccccc2)n1	0.0	0.0	not tested	not tested	0.0	0.0
SN00796936	CL9360	CCOc1ccc(cc1)NC(=O)Cn1c(=O)n(CC=C)c(=O)c2sc(nc12)N1CCCCC1	0.0	0.0	not tested	not tested	0.0	0.0
SN00790320	CL7708B	COc1ccc(NC(=O)C2CCCN(C2)c2ccnc(n2)c2ccc(C)cc2)c(OC)c1	0.0	0.0	not tested	not tested	0.0	0.0
SN00790235	CL7966B	O=C(NCc1ccc2OCOc2c1)C1(C)CCN(CC1)S(=O)(=O)c1cccs1	0.0	0.0	not tested	not tested	0.0	0.0
SN00793422	CL6945A	COc1ccc(cc1)c1nn2c(n1)sc(CCNC(=O)c1ccco1)c2C	0.0	0.0	not tested	not tested	0.0	0.0
SN00784400	CL5887	CCn1nc(C)c2[nH]c(=O)n(Cc3ccc(F)cc3)c(=O)c12	0.0	0.0	not tested	not tested	0.0	0.0
SN00782074	SC015501	CC(=O)NC(C)(C)c1noc(n1)c1cnc(s1)C(C)(C)C	0.0	0.0	not tested	not tested	0.0	0.0
SN00791653	CL6154	O=C(NCc1cccnc1)c1noc2ncnc(N3CCCC3)c12	0.0	0.0	not tested	not tested	0.0	0.0

Compound ID	Scaffold	Smiles	% Inhibition n=1		% Inhibition n=2		Average % Inhibition	
			24h	48h	24h	48h	24h	48h
SN00793647	CL2286	Cc1nc2sc(C(=O)N3CCOCC3)c(C)c2c(n1)N1CCCCC1	0.0	0.0	not tested	not tested	0.0	0.0
SN00770147	SC001495	Fc1ccc(cc1)N1CCN(CC1)C1=NS(=O)(=O)c2ccccc12	0.0	0.0	not tested	not tested	0.0	0.0
SN00787823	CL3586A	COc1ccccc1NC(=O)C1=C(C)C(=O)OC21CCN(CC2)Cc1ccccc1	0.0	0.0	not tested	not tested	0.0	0.0
SN00798245	CL9556	CCN1CCN([CH]C(=O)N2CCCCC2)C(=O)CC1	0.0	0.0	not tested	not tested	0.0	0.0
SN00778965	SC009242	Fc1ccc(cc1)n1nc(C(=O)N2CCN(CC2)Cc2ccc3OCCc3c2)c2CCCc12	0.0	0.0	not tested	not tested	0.0	0.0
SN00771253	SC007963	Fc1ccc(cc1)S(=O)(=O)N1CCC(CC1)C(=O)N1CCc2sccc2C1	0.0	0.0	not tested	not tested	0.0	0.0
SN00791588	CL5797A	O=C(N1CCc2ccccc12)c1ncn2CC(C)(C(=O)NCc3ccccc3)N(C3CC3)C(=O)c12	0.0	0.0	not tested	not tested	0.0	0.0
SN00793995	CL8308C	CCc1onc(C)c1C1CCCN1C(=O)Nc1ccccc1C	0.0	0.0	not tested	not tested	0.0	0.0
SN00796012	CL8222	COc1cccc(c1)c1cn2ccn(c3ccccc3OC)c(=O)c2n1	0.0	0.0	not tested	not tested	0.0	0.0
SN00790456	CL8366	Clc1ccc(s1)S(=O)(=O)N1CCN=C1N1CCN(CC1)c1ccccc1F	0.0	0.0	not tested	not tested	0.0	0.0
SN00793752	CL1132	Fc1ccccc1[C@H]1C[C@H](Nc2nnnn12)c1cccs1	0.0	0.0	not tested	not tested	0.0	0.0
SN00798476	CL5449A	Cc1ccc(cc1)S(=O)(=O)Cc1noc(n1)C1CCN(CC1)S(=O)(=O)c1ccc(Cl)cc1	0.0	0.0	not tested	not tested	0.0	0.0
SN00775559	SC002126	COc1ccc(CCC2CCN(CC2)Cc2cc(=O)n3ccccc3n2)cc1	0.0	0.0	not tested	not tested	0.0	0.0
SN00791110	CL8463	Brc1ccc(s1)S(=O)(=O)n1cc2c(n1)n(C(C)C)c(=O)n(C(C)C)c2=O	0.0	0.0	not tested	not tested	0.0	0.0
SN00782616	SC013680	OC(CN1CCC(CC1)Cn1cccn1)CN(C)Cc1ccccc1	0.0	0.0	not tested	not tested	0.0	0.0
SN00793616	CL5710	Clc1ccc(CCNC(=O)Cn2c(=O)c(=O)n(Cc3ccccc3)c3ncccc23)cc1	0.0	0.0	not tested	not tested	0.0	0.0
SN00775677	SC002262	CC(=O)c1ccc(cc1)N1CCN(CC1)S(=O)(=O)c1c(C)nn(C)c1C	0.0	0.0	not tested	not tested	0.0	0.0
SN00784390	CL4868A	CC(=O)c1ccc(cc1)S(=O)(=O)N1CCC2(CC1)NC(=O)c1ccccc1N2	0.0	0.0	not tested	not tested	0.0	0.0
SN00782116	SC015928	CC(Nc1nccn2cnnc12)c1ccc(cc1)S(=O)(=O)C	0.0	0.0	not tested	not tested	0.0	0.0
SN00779579	SC010179	CCC1OCCN(C1)C(=O)c1nnn(c1)c1ccc2OCCOc2c1	0.0	0.0	not tested	not tested	0.0	0.0
SN00790143	CL8253	CCC(=O)Nc1nc2c(OC)cc(cc2s1)NS(=O)(=O)c1cc(Cl)ccc1OC	0.0	0.0	not tested	not tested	0.0	0.0
SN00796551	CL8891A	COc1ccc(cc1)n1nnc(C(=O)NCc2ccccc2F)c1c1cccnc1	0.0	0.0	not tested	not tested	0.0	0.0
SN00782167	SC016049	CC(C)c1oc(nc1c1ccccc1)C1CCCN1C(=O)c1nc2ccccc2c(=O)[nH]1	0.0	0.0	not tested	not tested	0.0	0.0
SN00791903	CL5763	O=C(NCCN1CCOCC1)C1Cc2cc(ccc2N1C(=O)C)S(=O)(=O)N1CCOCC1	0.0	0.0	not tested	not tested	0.0	0.0
SN00778539	SC008338	Fc1ccc(cc1)OCc1scc(n1)Cn1nc(oc1=O)c1ccc(F)cc1	0.0	0.0	not tested	not tested	0.0	0.0
SN00789460	CL7925	CCc1nnc2c(=O)n(CC(=O)NCc3ccc(Cl)cc3)c3ccccc3n12	0.0	0.0	not tested	not tested	0.0	0.0
SN00780481	SC011330	CC(C)COC(=O)N1CCCN(CC1)C(=O)c1ccccn1	0.0	0.0	not tested	not tested	0.0	0.0
SN00781445	SC014188	Cc1cccc(c1)Cn1[nH]cc/c/1=N\C(=O)Cn1c(=O)onc1c1ncccn1	0.0	0.0	not tested	not tested	0.0	0.0
SN00789141	CL6279	Cc1noc(c1C)c1c(C)sc(C)c1S(=O)(=O)N1CCC(=CC1)c1ccccc1	0.0	0.0	not tested	not tested	0.0	0.0
SN00777910	SC007680	Cn1c(=O)[nH]c(=O)c2c1nc(CN1CCN(CC1)C1CCCC1)n2C	0.0	0.0	not tested	not tested	0.0	0.0
SN00786054	CL6892	CCc1ccc(cc1)N1CC(CC1=O)c1nnc(s1)NC(=O)c1c(C)onc1c1ccccc1	0.0	0.0	not tested	not tested	0.0	0.0
SN00793723	CL7417	Brc1ccc(o1)c1onc(n1)c1ccc2c(ncn2C2CCCC2)c1	0.0	0.0	not tested	not tested	0.0	0.0
SN00783607	CL1388	Cc1ccc(cc1)S(=O)(=O)N1Cc2ccccc2CC1C(=O)N(C)C	0.0	0.0	not tested	not tested	0.0	0.0
SN00773738	SC000244	COc1cccc(OC)c1C(=O)OCc1nc2cc(ccc2n1CC)S(=O)(=O)N1CCCCC1	0.0	0.0	not tested	not tested	0.0	0.0
SN00789106	CL7757	CCc1onc(n1)c1sc(C)c(c1)S(=O)(=O)NCc1ccccc1	0.0	0.0	not tested	not tested	0.0	0.0
SN00770153	SC001495	CC(C)NC(=O)N1CCN(CC1)C1=NS(=O)(=O)c2ccccc12	0.0	0.0	not tested	not tested	0.0	0.0
SN00773019	SC014747	COc1ccc2CN(CCCc2c1)C(=O)NCCN1CCOCC1	0.0	0.0	not tested	not tested	0.0	0.0
SN00782323	SC001309	O=C(N1CCN(CC1)CC1CCCO1)C1CCC1	0.0	0.0	not tested	not tested	0.0	0.0
SN00797300	CL9350	CCn1nccc1c1csc(c1)S(=O)(=O)Nc1ccc(cc1)N1CCOCC1	0.0	0.0	not tested	not tested	0.0	0.0
SN00782524	SC012053	C1CCC(OC1)CN1CCCC(C1)N1CCCCCC1	0.0	0.0	not tested	not tested	0.0	0.0
SN00778366	SC008047	CC(N1CCN(CC1)Cc1sc(C)c(C)n1)C(=O)NC1CC1	0.0	0.0	not tested	not tested	0.0	0.0
SN00797463	CL9352A	COc1ccccc1c1noc(n1)C1CCCN1C(=O)NCc1ccco1	0.0	0.0	not tested	not tested	0.0	0.0
SN00772465	SC012627	CC(=CC1C(C(=O)N2CCC(CC2)c2nnc3CCCn23)C1(C)C)C	0.0	0.0	not tested	not tested	0.0	0.0
SN00772129	SC011466	O=C(Cc1ccc2CCCc2c1)N1CCC(CC1)c1nncn1C	0.0	0.0	not tested	not tested	0.0	0.0
SN00788354	CL2541	COc1ccc(NC(=O)c2nnn(CC(=O)Nc3ccc(C)cc3)c2N)c(OC)c1	0.0	0.0	not tested	not tested	0.0	0.0
SN00778477	SC008232	Clc1c[nH]c(c1)C(=O)N1CCCN(CC1)Cc1cscn1	0.0	0.0	not tested	not tested	0.0	0.0

Compound ID	Scaffold	Smiles	% Inhibition n=1		% Inhibition n=2		Average % Inhibition	
			24h	48h	24h	48h	24h	48h
SN00771302	SC008104	Cc1ccc(cc1)S(=O)(=O)N1CCCN(CC1)C(C)c1nc(N)nc(n1)N(C)C	0.0	0.0	not tested	not tested	0.0	0.0
SN00802102	CM1459	CC(C)CCN1CCCC(C1)c1nnc([nH]1)c1cnccn1	0.0	0.0	not tested	not tested	0.0	0.0
SN00786159	CL5015B	CCn1c(Cn2nc(C)cc2C)nc2cc(ccc12)NC(=O)c1ccco1	0.0	0.0	not tested	not tested	0.0	0.0
SN00780854	SC012255	Cc1cc(NCc2ccccc2CN2CCCC2)nc(n1)c1ccncc1	0.0	0.0	not tested	not tested	0.0	0.0
SN00794607	CL2785	CCS(=O)(=O)N1CCCC(C1)C(=O)NC(C)c1ccccc1	0.0	0.0	not tested	not tested	0.0	0.0
SN00802470	CL5459A	COc1ccc2c(CCc3c(onc23)c2ccc(cc2)NC(=O)c2cc(C)nn2C)c1	0.0	0.0	not tested	not tested	0.0	0.0
SN00797374	CL9351	O=C(NCc1ccco1)c1ccc(cc1)c1noc(n1)C1CCN(CC1)C(=O)c1ccccc1	0.0	0.0	not tested	not tested	0.0	0.0
SN00779672	SC010287	O=c1cc(C)n2nc(sc2n1)N1CCN(CC1)C(=O)c1ccccn1	0.0	0.0	not tested	not tested	0.0	0.0
SN00777430	SC007286	CCCCn1nnnc1CN1C(=O)NC(C)(c2ccc3OCOc3c2)C1=O	0.0	0.0	not tested	not tested	0.0	0.0
SN00781962	SC015250	CCc1sc(cc1C)c1nnc(CN2CC(C2)n2cccn2)o1	0.0	0.0	not tested	not tested	0.0	0.0
SN00774412	SC000896	CCc1noc(C)c1C(=O)N1CCCCC1CNS(=O)(=O)C	0.0	0.0	not tested	not tested	0.0	0.0
SN00795801	CL7202	Clc1ccc(cc1)CNC(=O)c1nnn(c1)c1cccc(F)c1	0.0	0.0	not tested	not tested	0.0	0.0
SN00792010	CL6285	CCN1CCN(CC1)S(=O)(=O)c1ccc(o1)c1onc(C)c1	0.0	0.0	not tested	not tested	0.0	0.0
SN00784329	CL2003	COc1c(OC)ccc(C2NC(C(=O)O)C3C(=O)N(c4ccc(F)cc4)C(=O)C23)c1OC	0.0	0.0	not tested	not tested	0.0	0.0
SN00791440	CL6014	O=C(N1CCCCC1)c1c(C)n(C)c(C)c1S(=O)(=O)NCCC1=CCCCC1	0.0	0.0	not tested	not tested	0.0	0.0
SN00785537	CL5306	Cc1ccc(NC(=O)N2CCCn3nc(C)cc23)c(c1)N(=O)=O	0.0	0.0	not tested	not tested	0.0	0.0
SN00790669	CL8421A	Cc1ccccc1CS(=O)(=O)Cc1ccc(o1)c1onc(n1)c1ccccn1	0.0	0.0	not tested	not tested	0.0	0.0
SN00782180	SC016121	CCOC1CC(N(C)C(=O)Cn2ccc(=O)[nH]c2=O)C21CCCCC2	0.0	0.0	not tested	not tested	0.0	0.0
SN00786216	CL7184	CCC(=O)N1[C@H]2CCCC[C@H]2C2(CCCCC2)n2nc(nc12)c1ccco1	0.0	0.0	not tested	not tested	0.0	0.0
SN00796565	CL8836	CCOC(=O)c1cccc(c1)NC(=O)c1ccc(cc1)n1cnc(NC(=O)C)c1	0.0	0.0	not tested	not tested	0.0	0.0
SN00782318	SC000925	CCN1CCN(CC1)C(=O)c1nn(c(C)n1)c1ccccc1C(C)C	0.0	0.0	not tested	not tested	0.0	0.0
SN00783854	CL4994	Cc1ccc(OCCn2c3ncnc(N)c3nc2S(=O)(=O)O)cc1	0.0	0.0	not tested	not tested	0.0	0.0
SN00774109	SC000772	Cc1nn(Cc2coc(n2)c2cccs2)c(C)c1S(=O)(=O)N1CCCCC1	0.0	0.0	not tested	not tested	0.0	0.0
SN00772685	SC013514	CN(Cc1ccc2OCCOc2c1)Cn1nccc1c1ccncc1	0.0	0.0	not tested	not tested	0.0	0.0
SN00797367	CL9338	Cc1cccc(c1)c1oc(C)c(Cn2nnc(C(=O)Nc3ccc4OCCOc4c3)c2N)n1	0.0	0.0	not tested	not tested	0.0	0.0
SN00786047	CL6892	CCc1ccccc1N1CC(CC1=O)c1nnc(NC(=O)C)s1	0.0	0.0	not tested	not tested	0.0	0.0
SN00776325	SC003137	O=C(NN1CC(=O)C(=C1N)c1nc2ccccc2[nH]1)c1ccccc1	0.0	0.0	not tested	not tested	0.0	0.0
SN00772408	SC012477	Cc1ccc(cc1)c1c[nH]c(n1)C1CCCCN1C(=O)c1cnccn1	0.0	0.0	not tested	not tested	0.0	0.0
SN00769368	SC000304	CC(C)CCCC(C)NC(=O)CN1C(=O)NC(Cc2c[nH]c3ccccc23)C1=O	0.0	0.0	not tested	not tested	0.0	0.0
SN00775447	SC001667	CN1CCCN(CC1)c1ncnc2cc(Cl)ccc12	0.0	0.0	not tested	not tested	0.0	0.0
SN00798957	CL9737	CCc1c(N)onc1C1CCN(CC1)C(=O)C1COc2ccccc2O1	0.0	0.0	not tested	not tested	0.0	0.0
SN00787901	CL5562	COc1ccc(cc1)N1C(=O)CN(CC1(C)C(=O)NC1CCCCCC1)S(=O)(=O)CC	0.0	0.0	not tested	not tested	0.0	0.0
SN00780345	SC011156	O=C(CSc1nnc(c(=O)[nH]1)c1cc(Cl)sc1Cl)Nc1noc(C)c1	0.0	0.0	not tested	not tested	0.0	0.0
SN00787175	CL4582	COc1cccc(c1)C1=NS(=O)(=O)N(C)C(=C1)C(=O)N1CCCCC1C	0.0	0.0	not tested	not tested	0.0	0.0
SN00782182	SC016121	CCCN(C(=O)CN(CCC)C1CC(OCC)C21CCCCC2)C1CCS(=O)(=O)C1	0.0	0.0	not tested	not tested	0.0	0.0
SN00771362	SC008198	Cc1nc(C)nc(c1)N1CCCN(CC1)c1scc(C)n1	0.0	0.0	not tested	not tested	0.0	0.0
SN00789469	CL7925	CCCc1nnc2c(=O)n(CC(=O)Nc3cccc(SC)c3)c3ccccc3n12	0.0	0.0	not tested	not tested	0.0	0.0
SN00781052	SC012714	NCCCCCN1CCCN(CC1)c1nc2ccccc2[nH]1	0.0	0.0	not tested	not tested	0.0	0.0
SN00784655	CL1729	Clc1ccc2nc(c3cscc3)c(Nc3ccc4OCOc4c3)n2c1	0.0	0.0	not tested	not tested	0.0	0.0
SN00777690	SC007575	COc1ccc(cc1)C1CCCN1Cc1nc(oc1C)c1ccccc1	0.0	0.0	not tested	not tested	0.0	0.0
SN00783626	CL1281	Cn1c(=O)n(C)c(=O)c2c1nc(Oc1ccccc1)n2C	0.0	0.0	not tested	not tested	0.0	0.0
SN00791675	CL6783	Cc1onc(C(=O)NC2CCN(CC2)Cc2ccccc2)c1CN1CCOCC1	0.0	0.0	not tested	not tested	0.0	0.0
SN00793942	CL8308B	CCc1onc(C)c1C1CCCN1Cc1cn(C)nc1C	0.0	0.0	not tested	not tested	0.0	0.0
SN00773403	SC016053	O=C1NC(=O)C2(CCN(C2)C(=O)CCCCCc2cccs2)N1	0.0	0.0	not tested	not tested	0.0	0.0
SN00783652	CL2120	COc1ccc(Cl)cc1Nc1nc(CC(=O)CSc2ccc(C)cc2)nc(N)n1	0.0	0.0	not tested	not tested	0.0	0.0
SN00787181	CL4582	CCN(CC)C(=O)C1=CC(=NS(=O)(=O)N1C)c1ccc(F)cc1	0.0	0.0	not tested	not tested	0.0	0.0

Compound ID	Scaffold	Smiles	% Inhibition n=1		% Inhibition n=2		Average % Inhibition	
			24h	48h	24h	48h	24h	48h
SN00795134	CL7780	Fc1cccc(c1)CC(=O)N1CCN(CC1)Cc1nc2cc(ccc2n1C)C(=O)N1CCCC1	0.0	0.0	not tested	not tested	0.0	0.0
SN00786000	CL6492	Cc1nc(C)c(s1)C(=O)Nc1scc(n1)c1ccc(cc1)c1ccccc1	0.0	0.0	not tested	not tested	0.0	0.0
SN00782966	SC009938	O=C(NCC(F)(F)F)CN1CCCC(CN2CCCCCC2)C1	0.0	0.0	not tested	not tested	0.0	0.0
SN00793896	CL8307B	Brc1cccc(c1)c1n[nH]cc1CN1CCCC1c1onc(C)c1	0.0	0.0	not tested	not tested	0.0	0.0
SN00783133	SC011954	O=N(=O)c1cccnc1NC1CCCN(Cc2ccccc2)C1	0.0	0.0	not tested	not tested	0.0	0.0
SN00773301	SC015442	c1ccc(cc1)c1nnc(o1)C1CC21CCN(CC2)c1nc2ccccc2o1	0.0	0.0	not tested	not tested	0.0	0.0
SN00796171	CL5888	O=C(Nc1ccc2OCCOc2c1)Cn1c(=O)n(Cc2ccccc2)c(=O)c2nn(C)cc12	0.0	0.0	not tested	not tested	0.0	0.0
SN00769541	SC000869	O=c1ccc(n[nH]1)C(=O)N1CCN(CC1)c1ccc(cc1)C(=O)C	0.0	0.0	not tested	not tested	0.0	0.0
SN00770998	SC007517	O=c1[nH]cc([nH]1)C(=O)N1CCN(CC1)C(=O)c1ccc(C)c(Cl)c1	0.0	0.0	not tested	not tested	0.0	0.0
SN00776849	SC006282	COC(=O)c1ccc(nn1)Nc1ccc(cc1)OC1CCCC1	0.0	0.0	not tested	not tested	0.0	0.0
SN00781057	SC012714	O=C(Nc1nncs1)N1CCCN(CC1)c1nc2ccccc2[nH]1	0.0	0.0	not tested	not tested	0.0	0.0
SN00788929	CL4722	CCN1CCN(CC1)C(=O)c1cnn2c1n(CC)c1CCN(C)Cc1c2=O	0.0	0.0	not tested	not tested	0.0	0.0
SN00786941	CL9243	O=C(CC(C)(C)C)N1CCC(CC1)c1nc2ccccc2[nH]1	0.0	0.0	not tested	not tested	0.0	0.0
SN00783252	SC014212	CC(=O)OC(c1onc(n1)C1CCN(CC1)C(=O)C1CCCC1)c1ccccc1	0.0	0.0	not tested	not tested	0.0	0.0
SN00787090	CL7919	Clc1cccc(c1)NC(=O)C1CC(=O)N=C(N1)N1CCCCC1	0.0	0.0	not tested	not tested	0.0	0.0
SN00783361	CL0031	O=C(Nc1cccc(c1)C(=O)C)Cn1cnc(n1)N(=O)=O	0.0	0.0	not tested	not tested	0.0	0.0
SN00792518	CL5449B	COc1cc(ccc1OC(C)C)c1noc(n1)C1CCCN(C1)S(=O)(=O)c1cccs1	0.0	0.0	not tested	not tested	0.0	0.0
SN00770480	SC002933	O=C(N1CCC(CC1)c1nnc2ccccn12)c1cc(nc2c1cnn2C(C)C)c1ccco1	0.0	0.0	not tested	not tested	0.0	0.0
SN00783312	CL4994	OCC1OC(C(O)C1O)n1cnc2c(N)n(=O)cnc12	0.0	0.0	not tested	not tested	0.0	0.0
SN00772942	SC014342	CCCc1noc(n1)CN1CCCC1c1nncn1CC(C)C	0.0	0.0	not tested	not tested	0.0	0.0
SN00784933	CL3315	CCCCN(C)CCCNC(=O)c1cccc(c1)N1CCCC1=O	0.0	0.0	not tested	not tested	0.0	0.0
SN00788997	CL5326	O=C(NC1CCCCCCC1)C1CCN(CC1)C(=O)c1cccn1c1cccnc1	0.0	0.0	not tested	not tested	0.0	0.0
SN00792384	CL6785A	COc1ccc(OC)c(c1)S(=O)(=O)NCc1c(C)nn(C)c1C	0.0	0.0	not tested	not tested	0.0	0.0
SN00785484	CL3749	CC1CCC(CC1)NC(=O)C1CCCN(C1)C(=O)c1cc2sccc2n1C	0.0	0.0	not tested	not tested	0.0	0.0
SN00778321	SC008040	O=N(=O)c1cc(ccc1N1CCCCCC1)C(=O)N1CCCC1c1nnc2ccccn12	0.0	0.0	not tested	not tested	0.0	0.0
SN00781130	SC013362	O=C(C1CCOC1)N1CCN(CC1CNC(=O)C1CCOC1)C(=O)OC(C)(C)C	0.0	0.0	not tested	not tested	0.0	0.0
SN00793142	CL4469C	COC(=O)c1sccc1NC(=O)N1CCN(CC1)c1nc2cc(F)ccc2n2cccc12	0.0	0.0	not tested	not tested	0.0	0.0
SN00772493	SC012743	CCNC(=O)NC(=O)CN1CC2CC3CC(C2)CC1C3	0.0	0.0	not tested	not tested	0.0	0.0
SN00798601	CL9613	Cc1nnc(o1)c1cc(NC(=O)c2sc(C)nc2C)c(C)s1	0.0	0.0	not tested	not tested	0.0	0.0
SN00776347	SC003137	COc1cccc(c1)N1CC(=O)C(=C1N)c1nc2ccccc2n1C	0.0	0.0	not tested	not tested	0.0	0.0
SN00783599	CL2609	Cc1ccc(cc1)C(=O)CC1(O)C(=O)Nc2ccc(C)cc12	0.0	0.0	not tested	not tested	0.0	0.0
SN00791998	CL6285	CCC(C)NS(=O)(=O)c1ccc(o1)c1onc(C)c1	0.0	0.0	not tested	not tested	0.0	0.0
SN00800083	CM0328	O=C(C1CCC1)N1CCC(CC1)Oc1ccc(cn1)C(=O)N1CCOCC1	0.0	0.0	not tested	not tested	0.0	0.0
SN00774097	SC000765	NC(=O)c1cn(Cc2csc(n2)C(C)C)nc1c1ccccc1	0.0	0.0	not tested	not tested	0.0	0.0
SN00781168	SC013399	O=C(Nc1ccc(cc1)OCc1noc(n1)C1CC1)NC(C)C(C)C	0.0	0.0	not tested	not tested	0.0	0.0
SN00770406	SC002418	CC(N1CCN(CC1)Cc1nc2ccccn2c1)C(=O)c1[nH]c(C)c(C(=O)C)c1C	0.0	0.0	not tested	not tested	0.0	0.0
SN00776843	SC006267	CCC(=O)N1CCN(CC1)c1nc2c(c(=O)[nH]c(=O)n2C)n1CC	0.0	0.0	not tested	not tested	0.0	0.0
SN00784645	CL2048	Oc1cc(cc2[C@@H]3NC(=O)N(c4ccc(C)c(C)c4)[C@](C)(C3)Oc12)C(C)(C)C	0.0	0.0	not tested	not tested	0.0	0.0
SN00792821	CL7253A	CCS(=O)(=O)N1CC(OCc2ccc(F)cc2Cl)C1	0.0	0.0	not tested	not tested	0.0	0.0
SN00795898	CL8682A	Cc1ccc(cc1)NC(=O)N1CCCC(C)(C1)C(=O)NCc1ccc(F)cc1	0.0	0.0	not tested	not tested	0.0	0.0
SN00798389	CL9640	O=C(Cn1ccc2ccc(cc12)c1nnc(C)o1)N1CCCc2ccccc12	0.0	0.0	not tested	not tested	0.0	0.0
SN00793362	CL1388	O=C(Nc1ccccc1)C1Cc2ccccc2CN1S(=O)(=O)c1cccs1	0.0	0.0	not tested	not tested	0.0	0.0
SN00789523	CL3979	O=C(NC1CCCC1)Cn1c2nccnc2c(=O)n(CCc2ccccc2)c1=O	0.0	0.0	not tested	not tested	0.0	0.0
SN00781354	SC014139	O=C(Cc1c(C)nn(C)c1C)N1CCC(C1)N1CCCC1	0.0	0.0	not tested	not tested	0.0	0.0
SN00770271	SC001787	CC(N1CCN(CC1)C(=O)c1[nH]nc2ccccc12)C(=O)Nc1cccc2ccccc12	0.0	0.0	not tested	not tested	0.0	0.0
SN00770286	SC001884	Clc1ccc(OCCN2CCN(CC2)CN2C(=O)NC3(CCCCC3)C2=O)cc1	0.0	0.0	not tested	not tested	0.0	0.0
SN00771361	SC008198	COc1cccc(c1)C(=O)N1CCCN(CC1)c1cc(C)nc(C)n1	0.0	0.0	not tested	not tested	0.0	0.0

Compound ID	Scaffold	Smiles	% Inhibition n=1		% Inhibition n=2		Average % Inhibition	
			24h	48h	24h	48h	24h	48h
SN00771376	SC008231	Cc1ccn2cc(nc2c1)C(=O)N1CCCN(CC1)C(=O)C	0.0	0.0	not tested	not tested	0.0	0.0
SN00771391	SC008243	Clc1ccc(cc1)c1n[nH]c(c1)C(=O)N1CCCN(CC1)C(=O)C	0.0	0.0	not tested	not tested	0.0	0.0
SN00771399	SC008243	Clc1ccc(cc1)CN1CCCN(CC1)C(=O)c1[nH]ncc1	0.0	0.0	not tested	not tested	0.0	0.0
SN00772449	SC012606	FC(F)Oc1ccc(cc1)NC(=O)N1CCNC(=O)CC1c1ccccc1	0.0	0.0	not tested	not tested	0.0	0.0
SN00772499	SC012743	S=c1[nH]cccc1C(=O)N1CC2CC3CC(C2)CC1C3	0.0	0.0	not tested	not tested	0.0	0.0
SN00773485	SC016316	CC(C)c1n[nH]c(n1)C1OCCN(CCc2ccncc2)C1	0.0	0.0	not tested	not tested	0.0	0.0
SN00773541	SC000009	CN(Cc1ccc2OCOc2c1)CC(=O)c1cc(C)n(c1C)c1c(C)n(C)n(c2ccccc2)c1=O	0.0	0.0	not tested	not tested	0.0	0.0
SN00774479	SC000902	O=C(N1CCCCC1)c1ccc2C(=O)N(C(=O)c2c1)c1ccccc1C	0.0	0.0	not tested	not tested	0.0	0.0
SN00775650	SC002205	C=CCN(c1scc(n1)c1ccccc1)C(=O)C1CCN(CC1)S(=O)(=O)c1c(C)noc1C	0.0	0.0	not tested	not tested	0.0	0.0
SN00775678	SC002262	CC(=O)N1CCN(CC1)S(=O)(=O)c1c(C)n[nH]c1C	0.0	0.0	not tested	not tested	0.0	0.0
SN00775739	SC002601	O=C(CCCc1cccs1)Nc1ccc(cc1)c1csc2=NCCCn12	0.0	0.0	not tested	not tested	0.0	0.0
SN00776845	SC006282	c1ccc(cc1)CNc1ccc(nn1)Nc1ccccc1	0.0	0.0	not tested	not tested	0.0	0.0
SN00777823	SC007630	O=C(NCc1ccnc(c1)n1ccnc1C)c1[nH]cc(c1)S(=O)(=O)N1CCOCC1	0.0	0.0	not tested	not tested	0.0	0.0
SN00777835	SC007674	Clc1snnc1CN1CCN(CC1)Cc1ccco1	0.0	0.0	not tested	not tested	0.0	0.0
SN00770225	SC001665	Cc1ccc(cc1)c1csc2ncnc(N3CCCN(CC3)CC(=O)NC(C)(C)C)c12	0.0	0.0	not tested	not tested	0.0	0.0
SN00770244	SC001665	NC(=O)N1CCCN(CC1)c1nc(nc2scc(c3ccccc3)c12)C1CC1	0.0	0.0	not tested	not tested	0.0	0.0
SN00770304	SC001884	CCC(C)NC(=O)CN1CCN(CC1)CN1C(=O)NC2(CCCCC2)C1=O	0.0	0.0	not tested	not tested	0.0	0.0
SN00770320	SC002229	Fc1ccc(OCCCCNC(=O)C2CCCN2S(=O)(=O)c2c(C)noc2C)cc1	0.0	0.0	not tested	not tested	0.0	0.0
SN00770323	SC002229	Cc1onc(C)c1S(=O)(=O)N1CCCC1Cc1ccccc1	0.0	0.0	not tested	not tested	0.0	0.0
SN00770341	SC002336	CCc1ccccc1NC(=O)c1cncc(c1)c1cnn(C)c1	0.0	0.0	not tested	not tested	0.0	0.0
SN00770361	SC002336	Cn1ncc(c1)c1cncc(c1)C(=O)Nc1ccc(cc1)c1ncc[nH]1	0.0	0.0	not tested	not tested	0.0	0.0
SN00770375	SC002407	CCOC(=O)C1=C(CN2CCN(CCOc3ccc(OC)cc3)CC2)NC(=O)NC1	0.0	0.0	not tested	not tested	0.0	0.0
SN00770391	SC002407	CCOC(=O)C1=C(CN2CCN(CC2)Cc2cscn2)NC(=O)NC1	0.0	0.0	not tested	not tested	0.0	0.0
SN00770402	SC002418	CC1CCc2c(C1)sc1ncnc(N3CCN(CC3)Cc3nc4ccccn4c3)c21	0.0	0.0	not tested	not tested	0.0	0.0
SN00770427	SC002445	Clc1ccc(cc1)c1sc2c(=O)[nH]c(nc2c1)CN1CCCC1c1cccn1C	0.0	0.0	not tested	not tested	0.0	0.0
SN00771427	SC008246	O=C(N1CCCN(CC1)c1cccnn1)c1cc(C)n(C2CC2)c1C	0.0	0.0	not tested	not tested	0.0	0.0
SN00771448	SC008253	O=C(NC1CCCCC1)N1CCCN(CC1)C(=O)c1ccsc1	0.0	0.0	not tested	not tested	0.0	0.0
SN00771449	SC008253	O=C(Cc1c(F)cccc1Cl)N1CCCN(CC1)C(=O)c1ccsc1	0.0	0.0	not tested	not tested	0.0	0.0
SN00771461	SC008255	Fc1ccc(cc1)S(=O)(=O)N1CCCN(CC1)C(=O)c1nccnc1c1nc2ccccc2s1	0.0	0.0	not tested	not tested	0.0	0.0
SN00771505	SC008256	NC(=O)CN1CCCN(CC1)C(=O)C1(CCCC1)c1cccc(F)c1	0.0	0.0	not tested	not tested	0.0	0.0
SN00771513	SC008386	Brc1ccc(cc1)SCC(=O)N1CC(=O)NC(=O)C21CCCCC2	0.0	0.0	not tested	not tested	0.0	0.0
SN00771515	SC008386	O=C1NC(=O)C2(CCCCC2)N(C1)C(=O)Cn1cc(cc(Cl)c1=O)C(F)(F)F	0.0	0.0	not tested	not tested	0.0	0.0
SN00776880	SC006388	OC1=C(C(c2ccncc2)N(CCN2CCOCC2)C1=O)C(=O)c1c(C)nc2ccccn12	0.0	0.0	not tested	not tested	0.0	0.0
SN00777928	SC007715	O=C(N1CCCN(CC1)Cc1cscn1)C(C)NS(=O)(=O)C	0.0	0.0	not tested	not tested	0.0	0.0
SN00777952	SC007716	CCOC(C)c1noc(CN2CCCN(CC2)C(C)C(=O)N(C)Cc2ccccc2)n1	0.0	0.0	not tested	not tested	0.0	0.0
SN00777966	SC007717	Fc1ccc(cc1)N1CCCN(CC1)Cc1nnc(C)o1	0.0	0.0	not tested	not tested	0.0	0.0
SN00777983	SC007731	Cc1cc(c2nnc(o2)CN2CCc3sccc3C2c2cccs2)c(C)o1	0.0	0.0	not tested	not tested	0.0	0.0
SN00778049	SC007829	O=N(=O)c1c(nc2ccccn12)N1CCN(CCCc2onc(n2)c2cccs2)CC1	0.0	0.0	not tested	not tested	0.0	0.0
SN00779721	SC010300	CC1CCCN(CCN2CCC(CC2)c2onc(n2)c2ccccn2)C1	0.0	0.0	not tested	not tested	0.0	0.0
SN00779744	SC010301	COc1ccc(cc1)c1noc(n1)c1[nH]nc(c1)c1ccc(OC)cc1OC	0.0	0.0	not tested	not tested	0.0	0.0
SN00779762	SC010358	CCOC1CC(NC(=O)c2cccnc2O)C21CCC2	0.0	0.0	not tested	not tested	0.0	0.0
SN00779819	SC010465	Cc1ncncc1C(=O)N1CCN(CC1)CC(O)C(C)(C)C	0.0	0.0	not tested	not tested	0.0	0.0
SN00779824	SC010570	Cc1ccc(o1)C1CCCN1S(=O)(=O)CC1CCCO1	0.0	0.0	not tested	not tested	0.0	0.0
SN00779834	SC010570	Cn1ncc(c1)CN(C)S(=O)(=O)N1CCCC1c1ccc(C)o1	0.0	0.0	not tested	not tested	0.0	0.0
SN00780650	SC011804	COC(=O)c1c2CCCn2c(c2ccc(cc2)NC(=O)/C=C/c2ccccc2N(=O)=O)c1C(=O)OC	0.0	0.0	not tested	not tested	0.0	0.0
SN00780722	SC011885	CCOC(=O)c1ccc(o1)S(=O)(=O)N1CCN(CC1)c1cc(C)nc(C)n1	0.0	0.0	not tested	not tested	0.0	0.0

Compound ID	Scaffold	Smiles	% Inhibition n=1		% Inhibition n=2		Average % Inhibition	
			24h	48h	24h	48h	24h	48h
SN00780733	SC011885	CCOC(=O)c1ccc(o1)S(=O)(=O)N1CCN(CC1)C1CCOC1=O	0.0	0.0	not tested	not tested	0.0	0.0
SN00780751	SC011954	CCOC(=O)c1cccnc1NC1CCCN(C1)c1ncccn1	0.0	0.0	not tested	not tested	0.0	0.0
SN00780755	SC012049	Clc1ccc(s1)c1c[nH]cc1C(=O)NCc1ccccc1F	0.0	0.0	not tested	not tested	0.0	0.0
SN00780770	SC012049	Clc1ccc(s1)c1c[nH]cc1C(=O)NCCc1csc(C)n1	0.0	0.0	not tested	not tested	0.0	0.0
SN00780788	SC012058	CCOC(=O)c1cnn(c1N)c1cc(C)nc2c(C)c(nn12)c1ccccc1	0.0	0.0	not tested	not tested	0.0	0.0
SN00780807	SC012058	CCOC(=O)c1cnn(c1N)c1cc(C)nc2c(C)c(nn12)c1ccc(Cl)cc1	0.0	0.0	not tested	not tested	0.0	0.0
SN00780817	SC012060	Cc1cc(N2CCOCC2)n2nc(c3ccccc3)c(C)c2n1	0.0	0.0	not tested	not tested	0.0	0.0
SN00780834	SC012214	Cn1ncc(c1)N1CCC(NC(=O)c2ccc(cc2)C(C)(C)C)C1=O	0.0	0.0	not tested	not tested	0.0	0.0
SN00770130	SC001484	Cc1csc(n1)N1CCC(CC1)C(=O)Nc1ccc(F)cc1F	0.0	0.0	not tested	not tested	0.0	0.0
SN00771207	SC007875	CCn1nc(C(=O)N2CCCN(CC2)S(=O)(=O)c2ccc(C)cc2)c2ccccc2c1=O	0.0	0.0	not tested	not tested	0.0	0.0
SN00771211	SC007875	CCn1nc(C(=O)N2CCCN(CC2)S(=O)(=O)c2ccc(F)cc2)c2ccccc2c1=O	0.0	0.0	not tested	not tested	0.0	0.0
SN00771314	SC008114	C1CN(CCN(C1)Cc1nnnn1C1CC1)Cc1nnnn1C1CC1	0.0	0.0	not tested	not tested	0.0	0.0
SN00771331	SC008115	Clc1cccc(c1)C(=O)N1CCCN(CC1)Cc1noc(C)c1	0.0	0.0	not tested	not tested	0.0	0.0
SN00772410	SC012477	Fc1cccc(c1)c1cnc([nH]1)C1CCCCN1c1ccc(c(c1)N(=O)=O)S(=O)(=O)C	0.0	0.0	not tested	not tested	0.0	0.0
SN00772647	SC013398	CCCOc1ccc(CNC(=O)N2CCCN(CC2)C2CC2)cc1OC	0.0	0.0	not tested	not tested	0.0	0.0
SN00772648	SC013434	N1CCC(CC1)c1[nH]nc(n1)c1ccccc1	0.0	0.0	not tested	not tested	0.0	0.0
SN00773555	SC000010	CCCC1(CCC)NC(=O)N(Cc2nc(Nc3ccc(C)cc3)nc(N)n2)C1=O	0.0	0.0	not tested	not tested	0.0	0.0
SN00774419	SC000898	Fc1ccc(cc1)n1nc(C)c2cc(sc12)C(=O)N1CCCCC1C(=O)N	0.0	0.0	not tested	not tested	0.0	0.0
SN00774645	SC000960	COc1ccc(cc1)NC(=O)C1CCCN1C(=O)c1cn2ccc(C)cc2n1	0.0	0.0	not tested	not tested	0.0	0.0
SN00774659	SC000960	CCN1CCCC1C1CCCN1C(=O)c1cn2ccc(C)cc2n1	0.0	0.0	not tested	not tested	0.0	0.0
SN00774665	SC000969	O=C(CN1CCN(CC1)C(=O)C1CCC1)Nc1cccc(F)c1	0.0	0.0	not tested	not tested	0.0	0.0
SN00774674	SC000969	O=C(CCN1CCN(CC1)C(=O)C1CCC1)OC(C)(C)C	0.0	0.0	not tested	not tested	0.0	0.0
SN00775553	SC002126	O=C(C1CCN(CC1)Cc1cc(=O)n2cccc(C)c2n1)N1CCCCCC1	0.0	0.0	not tested	not tested	0.0	0.0
SN00775575	SC002128	O=c1[nH]c(CN2CCC(CC2)c2nc3ccccc3o2)nc2ccsc12	0.0	0.0	not tested	not tested	0.0	0.0
SN00775581	SC002128	CC1CCN(CC1)Cc1nc2ccsc2c(=O)[nH]1	0.0	0.0	not tested	not tested	0.0	0.0
SN00775875	SC002921	O=C(CN1CCN(CC1)Cc1c(nc2ccccn12)c1ccccc1)NC1CC1	0.0	0.0	not tested	not tested	0.0	0.0
SN00775878	SC002921	Cc1ccc2nc(c3ccccc3)c(CN3CCN(CC3)C3CCS(=O)(=O)C3)n2c1	0.0	0.0	not tested	not tested	0.0	0.0
SN00775891	SC002931	O=C(/C=C/c1nnc2n1ncc1ccccc21)Nc1cccc(C)c1C	0.0	0.0	not tested	not tested	0.0	0.0
SN00776777	SC005855	CCc1c([nH]nc1c1ccncc1)NC(=O)Cc1ccccc1	0.0	0.0	not tested	not tested	0.0	0.0
SN00776803	SC005969	O=C(CCN1C(=O)CSc2ccccc12)Nc1nnc([nH]1)c1ccccn1	0.0	0.0	not tested	not tested	0.0	0.0
SN00777870	SC007675	CCCCCCn1c(CN2CCCCC2CC)nc2c1c(=O)n(C)c(=O)n2C	0.0	0.0	not tested	not tested	0.0	0.0
SN00777873	SC007675	Fc1ccc(cc1)Cn1c(CN2CCC(CC2)C(=O)N)nc2c1c(=O)n(C)c(=O)n2C	0.0	0.0	not tested	not tested	0.0	0.0
SN00777905	SC007680	Cn1c(=O)[nH]c(=O)c2c1nc(CN1CCN(CC1)Cc1ccccc1)n2CCCc1ccccc1	0.0	0.0	not tested	not tested	0.0	0.0
SN00779612	SC010190	Cn1ncc(c1)C1CCCN1C(=O)c1nnn(c2ccccc2)c1C	0.0	0.0	not tested	not tested	0.0	0.0
SN00779667	SC010225	CCCCc1onc(n1)CN1CC(C(C)C1)N1CCN(CC1)C(=O)C	0.0	0.0	not tested	not tested	0.0	0.0
SN00779681	SC010287	O=c1cc(C)n2nc(sc2n1)N1CCN(CC1)C(=O)CC12CC3CC(CC(C3)C2)C1	0.0	0.0	not tested	not tested	0.0	0.0
SN00780637	SC011787	Fc1ccc(cc1)CN1C(=O)NC(C)(c2ccccn2)C1=O	0.0	0.0	not tested	not tested	0.0	0.0
SN00780907	SC012282	CC1CCN(CCCNC(=O)c2cnc(cn2)n2cccn2)CC1	0.0	0.0	not tested	not tested	0.0	0.0
SN00780919	SC012373	CN(C1CCN(CC1)C1CCOCC1)C(=O)OC(C)(C)C	0.0	0.0	not tested	not tested	0.0	0.0
SN00770166	SC001552	O=C1NCCN(C1)c1ccc(cn1)NC(=O)c1ccc(cc1)Cn1ncnc1	0.0	0.0	not tested	not tested	0.0	0.0
SN00770199	SC001584	O=C1CSc2ccc(cc2N1)C(=O)Nc1ccc(cn1)N1CCOCC1	0.0	0.0	not tested	not tested	0.0	0.0
SN00770214	SC001584	O=C(Nc1ccc(cn1)N1CCOCC1)Cc1csc(n1)NC(=O)c1ccoc1C	0.0	0.0	not tested	not tested	0.0	0.0
SN00775799	SC002885	CC(C)CCNc1scc(n1)C(=O)OCc1cc(=O)n2c(n1)sc1CCCCc21	0.0	0.0	not tested	not tested	0.0	0.0
SN00776663	SC005543	Clc1ccc(c(Cl)c1)C(=O)C1=C(O)C(=O)N(c2nccs2)C1c1ccccc1C(=O)O	0.0	0.0	not tested	not tested	0.0	0.0
SN00776889	SC006388	Cc1ccc(o1)C1N(CCCN2CCOCC2)C(=O)C(=C1C(=O)c1c(C)nc2ccccn12)O	0.0	0.0	not tested	not tested	0.0	0.0
SN00777984	SC007731	Cc1nnc(o1)CN1CCc2sccc2C1c1ccccc1	0.0	0.0	not tested	not tested	0.0	0.0

Compound ID	Scaffold	Smiles	% Inhibition n=1		% Inhibition n=2		Average % Inhibition	
			24h	48h	24h	48h	24h	48h
SN00778021	SC007824	Oc1ccccc1C(=O)c1cc(n(C)c1)c1nc2ccccc2c(=O)[nH]1	0.0	0.0	not tested	not tested	0.0	0.0
SN00770082	SC001441	Cc1sc2nc(CN3CCCC3)nc(NCc3nc4ccccn4c3)c2c1C	0.0	0.0	not tested	not tested	0.0	0.0
SN00770083	SC001441	Cc1c(C)sc2nc(CN3CCCC3)nc(Sc3nnc4ccccn34)c12	0.0	0.0	not tested	not tested	0.0	0.0
SN00771187	SC007867	O=C(N1CCCN(CC1)C(=O)C)C1CN(c2ccccc2)C(=O)C1	0.0	0.0	not tested	not tested	0.0	0.0
SN00771194	SC007867	COc1ccnc(n1)N1CCCN(CC1)C(=O)C1CN(C)C(=O)C1	0.0	0.0	not tested	not tested	0.0	0.0
SN00772273	SC012158	CN1CCN(CC1)c1n(C)nc(C)c1N(=O)=O	0.0	0.0	not tested	not tested	0.0	0.0
SN00772301	SC012203	O=C(CC(C)NC(=O)C1CCCC1)NC1CCN(C1)c1cnn(C)c1	0.0	0.0	not tested	not tested	0.0	0.0
SN00772305	SC012203	O=C(NCc1c(C)cc(C)cc1C)NC1CCN(C1)c1cnn(C)c1	0.0	0.0	not tested	not tested	0.0	0.0
SN00772337	SC012288	C[C@@H]1CN(CCN1Cc1nnc(C2CC2)n1C)Cc1ccccc1	0.0	0.0	not tested	not tested	0.0	0.0
SN00772350	SC012380	O=C(NCc1ccccc1)NC(=O)Cn1cc(ccc1=O)c1nnco1	0.0	0.0	not tested	not tested	0.0	0.0
SN00772356	SC012380	Fc1ccc(Cn2cc(ccc2=O)c2nnco2)c2ncccc12	0.0	0.0	not tested	not tested	0.0	0.0
SN00772374	SC012476	O=C(CCc1ccco1)N1CCC(CC1)c1ncc([nH]1)c1ccc(F)c(F)c1	0.0	0.0	not tested	not tested	0.0	0.0
SN00772754	SC013672	CS(=O)(=O)CCN1CCCCC1Cn1ncnc1	0.0	0.0	not tested	not tested	0.0	0.0
SN00773439	SC016130	COc1ccc(cc1)Oc1ncccc1NC(=O)N1CCN(CC1)c1nccn1C	0.0	0.0	not tested	not tested	0.0	0.0
SN00773448	SC016190	CCOC(=O)c1c(NC(=O)CN2CCC32CCC3)sc(C(=O)N(C)C)c1C	0.0	0.0	not tested	not tested	0.0	0.0
SN00773458	SC016190	CCOc1cc(C)ccc1NC(=O)N1CCC21CCC2	0.0	0.0	not tested	not tested	0.0	0.0
SN00773469	SC016316	CC(C)c1n[nH]c(n1)C1OCCN(C1)C(=O)c1cnc[nH]1	0.0	0.0	not tested	not tested	0.0	0.0
SN00774361	SC000870	CCOCC(O)CN1CCN(CC1)C(=O)c1cc(C)nn1CC	0.0	0.0	not tested	not tested	0.0	0.0
SN00775506	SC001740	Brc1ccc(cc1)Sc1ncccc1C(=O)N1CCNC(=O)C1	0.0	0.0	not tested	not tested	0.0	0.0
SN00775525	SC001766	Fc1ccc(cc1)C1CCCN1C(=O)c1cnccn1	0.0	0.0	not tested	not tested	0.0	0.0
SN00775531	SC001766	Cc1cnc(cn1)C(=O)N1CCC(C1)c1ccccc1C	0.0	0.0	not tested	not tested	0.0	0.0
SN00775611	SC002131	CC1CCN(CC1)Cc1nc2scc(c3ccc(Cl)cc3)c2c(=O)[nH]1	0.0	0.0	not tested	not tested	0.0	0.0
SN00775899	SC002931	CCc1nnc2c3ccccc3c(nn12)c1ccc(C)c(C)c1	0.0	0.0	not tested	not tested	0.0	0.0
SN00776675	SC005619	O=C(CN1C(=O)C2CCCN2C1=O)Nc1ccccc1Oc1ccccc1	0.0	0.0	not tested	not tested	0.0	0.0
SN00776693	SC005619	OC(COCc1cccs1)CN1C(=O)C2CCCN2C1=O	0.0	0.0	not tested	not tested	0.0	0.0
SN00776707	SC005774	O=C(NCCOc1ccccc1)CCc1onc(n1)c1ccccn1	0.0	0.0	not tested	not tested	0.0	0.0
SN00776964	SC006580	O=C(CCn1[nH]c(=O)ccc1=O)Nc1scc(n1)c1ccc2NC(=O)CCc2c1	0.0	0.0	not tested	not tested	0.0	0.0
SN00776991	SC006621	Fc1cccc(Cl)c1CN1CCN(CC1)C(=O)c1cccc2cccnc12	0.0	0.0	not tested	not tested	0.0	0.0
SN00776997	SC006621	NC(=O)CN1CCN(CC1)C(=O)c1cc(Cl)cc2cccnc12	0.0	0.0	not tested	not tested	0.0	0.0
SN00777165	SC006719	CCc1ccc(cc1)c1nn(cc1C(=O)N1CCC1)c1ccccc1C	0.0	0.0	not tested	not tested	0.0	0.0
SN00779539	SC010132	CCCNC(=O)C1CCN(CC1)Cc1[nH]c(=O)c2cnn(C)c2n1	0.0	0.0	not tested	not tested	0.0	0.0
SN00779560	SC010158	CCC(C)(C)NC(=O)C1CC(=O)N(Cc2ccccn2)C1	0.0	0.0	not tested	not tested	0.0	0.0
SN00780841	SC012214	O=C1C(CCN1c1cnn(C)c1)NC(=O)c1cccc(C)c1C	0.0	0.0	not tested	not tested	0.0	0.0
SN00780878	SC012255	Fc1cccc(c1)C(c1cnn(C)c1)N(C)c1nc(nc(c1)C(F)(F)F)c1ccncc1	0.0	0.0	not tested	not tested	0.0	0.0
SN00780889	SC012282	COCc1ccc(cc1)CNc1cncc(n1)n1cccn1	0.0	0.0	not tested	not tested	0.0	0.0
SN00769451	SC000448	O=C1C(=O)N(Cc2ccco2)C(=O)N1CC(=O)c1cc(C)n(c2nccs2)c1C	0.0	0.0	not tested	not tested	0.0	0.0
SN00769455	SC000448	O=C(NCC1COc2ccccc2O1)CN1C(=O)N(Cc2ccco2)C(=O)C1=O	0.0	0.0	not tested	not tested	0.0	0.0
SN00769488	SC000718	Clc1cnn(Cc2onc(n2)c2cscc2)c(=O)c1Cl	0.0	0.0	not tested	not tested	0.0	0.0
SN00769498	SC000718	NCc1c(C)c(C)nn(Cc2onc(Cc3ccccc3)n2)c1=O	0.0	0.0	not tested	not tested	0.0	0.0
SN00769519	SC000791	Brc1ccccc1c1nnc(o1)Cn1cnc2sccc2c1=O	0.0	0.0	not tested	not tested	0.0	0.0
SN00770604	SC004472	Fc1cccc(OCCN(C)C(=O)c2ccc(nn2)n2cncc2)c1	0.0	0.0	not tested	not tested	0.0	0.0
SN00770605	SC004472	COc1ccccc1C1(CNC(=O)c2ccc(nn2)n2cncc2)CC1	0.0	0.0	not tested	not tested	0.0	0.0
SN00771697	SC008893	O=C1NCCN(C(C)C1)c1ncnc2sc(cc12)c1ccccc1	0.0	0.0	not tested	not tested	0.0	0.0
SN00771708	SC008911	CC(C)C(C)NC(=O)c1nnn(c1)c1ccc(cn1)C(F)(F)F	0.0	0.0	not tested	not tested	0.0	0.0
SN00771735	SC009017	Fc1ccccc1c1nc2onc(C)c2c(c1)C(=O)N1CCCC1c1nnc2ccccn12	0.0	0.0	not tested	not tested	0.0	0.0
SN00772743	SC013672	O=C(NCc1ccco1)CN1CCCCC1Cn1cncn1	0.0	0.0	not tested	not tested	0.0	0.0

Compound ID	Scaffold	Smiles	% Inhibition n=1		% Inhibition n=2		Average % Inhibition	
			24h	48h	24h	48h	24h	48h
SN00772767	SC013674	O=S(=O)(CCN1CCCC1Cn1cncn1)c1ccccc1	0.0	0.0	not tested	not tested	0.0	0.0
SN00772773	SC013674	O=C(NC1CCN(CC1)C1CC1)CN1CCCC1Cn1cncn1	0.0	0.0	not tested	not tested	0.0	0.0
SN00772849	SC013920	COc1ccc(OC)c(c1)C1CCCN1Cc1ccn(n1)c1ccccc1	0.0	0.0	not tested	not tested	0.0	0.0
SN00773676	SC000202	NCCCCSc1nc2scc(c3ccco3)c2c(=O)n1CC	0.0	0.0	not tested	not tested	0.0	0.0
SN00773696	SC000212	Cc1ccc(cc1)n1ncc(c1)C(=O)c1cc(C)ccc1O	0.0	0.0	not tested	not tested	0.0	0.0
SN00773708	SC000214	Nc1nc(COC(=O)c2sc3nc4CCCCCn4c(=O)c3c2C)nc(n1)Nc1ccccc1C	0.0	0.0	not tested	not tested	0.0	0.0
SN00773711	SC000214	CCCNC(=O)c1sc2nc3CCCCCn3c(=O)c2c1C	0.0	0.0	not tested	not tested	0.0	0.0
SN00773770	SC000303	O=c1[nH]c2ccccc2c2nc(nn12)c1cccc(c1)S(=O)(=O)Nc1cccc(c1)C(F)(F)F	0.0	0.0	not tested	not tested	0.0	0.0
SN00773796	SC000303	O=c1[nH]c2ccccc2c2nc(nn12)c1ccco1	0.0	0.0	not tested	not tested	0.0	0.0
SN00774793	SC000974	COc1cc2CCN(C(CC(=O)O)c2cc1OC)C(=O)c1ccco1	0.0	0.0	not tested	not tested	0.0	0.0
SN00774798	SC000974	COc1cc2CN(CCc2cc1OC)C(=O)c1occc1c1ccccc1	0.0	0.0	not tested	not tested	0.0	0.0
SN00774803	SC000979	Cc1onc(c1)C(=O)N1CCN(CC1)S(=O)(=O)c1ccccc1	0.0	0.0	not tested	not tested	0.0	0.0
SN00774828	SC000979	CC(O)CN1CCN(CC1)C(=O)c1noc(c1)c1ccccc1F	0.0	0.0	not tested	not tested	0.0	0.0
SN00774835	SC000981	Fc1ccc(cc1)N1CCN(CC1)C(=O)c1ccc2OCCOc2c1	0.0	0.0	not tested	not tested	0.0	0.0
SN00774840	SC000981	O=C(N1CCN(CC1)S(=O)(=O)Cc1ccccc1)c1cc(Cl)c2OCCOc2c1	0.0	0.0	not tested	not tested	0.0	0.0
SN00774866	SC000983	O=C(N1CCN(CC1)c1ccccc1)C1Cc2ccccc2CN1C(=O)c1ccco1	0.0	0.0	not tested	not tested	0.0	0.0
SN00774872	SC000983	O=C(c1ccccc1)N1Cc2ccccc2CC1C(=O)N1CCN(CC1)c1ccccc1	0.0	0.0	not tested	not tested	0.0	0.0
SN00774906	SC000984	O=C(CN1CCN(CC1)C(=O)c1ccc2ncsc2c1)Nc1c(C)cccc1C	0.0	0.0	not tested	not tested	0.0	0.0
SN00774909	SC000984	Cc1ccc(cc1)c1noc(CCCN2CCN(CC2)C(=O)c2ccc3ncsc3c2)n1	0.0	0.0	not tested	not tested	0.0	0.0
SN00774919	SC000986	O=c1[nH]c2ccccc2c(c1)C(=O)N1CCN(CC1)c1ccccc1F	0.0	0.0	not tested	not tested	0.0	0.0
SN00775988	SC002978	Cc1cnn(c1)C1CCN(CC1)C(=O)c1cc(ccc1Cl)N(=O)=O	0.0	0.0	not tested	not tested	0.0	0.0
SN00776000	SC002981	CCOC(=O)c1c[nH]n(C2CCN(CC2)C(=O)C2(C)CCCC2)c1=N	0.0	0.0	not tested	not tested	0.0	0.0
SN00776025	SC003012	Cc1c(C)sc2nc(CN3CCOCC3)nc(NC3CCS(=O)(=O)C3)c12	0.0	0.0	not tested	not tested	0.0	0.0
SN00776040	SC003029	COc1cc(ccc1OC)c1noc(CN2CCNC(=O)C2)n1	0.0	0.0	not tested	not tested	0.0	0.0
SN00777121	SC006689	O=c1cc(C)c2ccc(OCc3nnc(o3)c3c(C)onc3c3ccccc3)cc2o1	0.0	0.0	not tested	not tested	0.0	0.0
SN00777129	SC006712	Fc1cc(ccc1N1CCN(CC1)C(=O)c1ccc[nH]c1=O)C(=O)C	0.0	0.0	not tested	not tested	0.0	0.0
SN00777148	SC006712	O=c1[nH]c(ccc1C(=O)N1CCN(CC1)c1ccccc1)c1ccco1	0.0	0.0	not tested	not tested	0.0	0.0
SN00777159	SC006719	Cc1ccc(c(C)c1)c1nn(cc1C(=O)N1CCC1)c1ccccc1	0.0	0.0	not tested	not tested	0.0	0.0
SN00777206	SC006761	OCCn1ncc(c1)Nc1ncnc2scc(c3ccc(C)c(C)c3)c12	0.0	0.0	not tested	not tested	0.0	0.0
SN00777225	SC006854	O=C(CCn1c(=O)oc2cc(Cl)ccc12)Nc1sc(c(C)n1)c1nccn1C(F)F	0.0	0.0	not tested	not tested	0.0	0.0
SN00778208	SC007962	CCN(CC)Cc1ccc(o1)C(=O)N1CCc2sccc2C1	0.0	0.0	not tested	not tested	0.0	0.0
SN00778287	SC008030	O=C(N1CCCN(CC1)c1nccs1)C1CCCN(C1)C(=O)C	0.0	0.0	not tested	not tested	0.0	0.0
SN00778293	SC008032	COCCn1nc(ccc1=O)C(=O)N1CCCN(CC1)c1ccc(cn1)C(F)(F)F	0.0	0.0	not tested	not tested	0.0	0.0
SN00778297	SC008032	CCCn1nc(ccc1=O)C(=O)N1CCCN(CC1)C(=O)C	0.0	0.0	not tested	not tested	0.0	0.0
SN00778945	SC009216	COc1ccc(OC)c(c1)C1CCCN1C(=O)c1cnc2c(cnn2C2CCCC2)c1	0.0	0.0	not tested	not tested	0.0	0.0
SN00780027	SC010798	O=C(CN1CCCCC1CN1CCCC1=O)N1CCc2ccccc12	0.0	0.0	not tested	not tested	0.0	0.0
SN00780056	SC010804	O=C(NC1CCCc2n(C)ncc12)N1CCCC1CN1CCCC1	0.0	0.0	not tested	not tested	0.0	0.0
SN00780061	SC010810	OC(=O)C1CC(=O)N(C)C1c1cccnc1	0.0	0.0	not tested	not tested	0.0	0.0
SN00769467	SC000452	Brc1ccc(cc1)C1CCCN1Cc1nnc(C)n1C	0.0	0.0	not tested	not tested	0.0	0.0
SN00769470	SC000452	Cc1onc(n1)C1CCCN1Cc1nnc(C2CC2)n1C	0.0	0.0	not tested	not tested	0.0	0.0
SN00769527	SC000791	CCc1nnc(o1)Cn1cnc2sccc2c1=O	0.0	0.0	not tested	not tested	0.0	0.0
SN00769605	SC000885	CN1CCN(C(=O)c2nc(oc2C)c2ccccc2)C(C1)c1ccccc1	0.0	0.0	not tested	not tested	0.0	0.0
SN00769632	SC000889	Fc1cc2c(=O)c(cn(C3CC3)c2cc1N1CCN(CC1)C(=O)c1c(C)noc1C)C(=O)O	0.0	0.0	not tested	not tested	0.0	0.0
SN00770510	SC002979	O=C(Nc1ccn(Cc2cccnc2)n1)c1ccc2[nH]nnc2c1	0.0	0.0	not tested	not tested	0.0	0.0
SN00770513	SC002979	O=C(Nc1ccn(Cc2cccnc2)n1)c1cnc2n[nH]c(C)c2c1	0.0	0.0	not tested	not tested	0.0	0.0
SN00770554	SC003349	O=c1c2ccccc2nc(CN2CCOCC2)n1Cc1scc(n1)c1ccc2CCCc2c1	0.0	0.0	not tested	not tested	0.0	0.0

Compound ID	Scaffold	Smiles	% Inhibition n=1		% Inhibition n=2		Average % Inhibition	
			24h	48h	24h	48h	24h	48h
SN00770562	SC003785	c1ccc(cc1)c1n[nH]cc1c1nc2ccccc2[nH]1	0.0	0.0	not tested	not tested	0.0	0.0
SN00770568	SC003785	Cn1ncc(c2nc3ccccc3[nH]2)c1N	0.0	0.0	not tested	not tested	0.0	0.0
SN00771636	SC008871	COc1ccc(cc1CN1CCCN(CC1)c1nccs1)C(=O)C	0.0	0.0	not tested	not tested	0.0	0.0
SN00771642	SC008871	O=C(N1CCCN(CC1)c1nccs1)C1Cc2ccccc2O1	0.0	0.0	not tested	not tested	0.0	0.0
SN00771664	SC008887	Cc1noc(c1)C(=O)N1CCCN(CC1)c1cccnn1	0.0	0.0	not tested	not tested	0.0	0.0
SN00771687	SC008893	Clc1ccc(cc1)c1csc2ncnc(N3CCNC(=O)CC3C)c12	0.0	0.0	not tested	not tested	0.0	0.0
SN00771729	SC009017	Cc1noc2nc(cc(C(=O)N3CCCC3c3nc4ccccc4s3)c12)c1ccccc1	0.0	0.0	not tested	not tested	0.0	0.0
SN00772730	SC013670	CC1CCC(CC1)NC(=O)CN1CCCC(C1)Cn1nc(C)nc1C	0.0	0.0	not tested	not tested	0.0	0.0
SN00772824	SC013678	CSC(C)C(=O)N1CCCC(C1)c1n[nH]c(C)c1	0.0	0.0	not tested	not tested	0.0	0.0
SN00773692	SC000212	COc1ccc(O)c(c1)C(=O)c1cnn(C(=S)Nc2ccccc2)c1N	0.0	0.0	not tested	not tested	0.0	0.0
SN00773810	SC000338	Clc1cccc(c1)N(C(=O)C)c1scc(Cn2nnc(n2)c2cccc(c2)C(F)(F)F)n1	0.0	0.0	not tested	not tested	0.0	0.0
SN00773834	SC000340	CCOC(=O)Nc1ccc2c(c1)oc(=O)cc2Cn1nnc(n1)c1ccc(C)cc1	0.0	0.0	not tested	not tested	0.0	0.0
SN00773838	SC000340	CCOC(=O)Nc1ccc2c(c1)oc(=O)cc2Cn1nnc(n1)c1ccc(Cl)cc1	0.0	0.0	not tested	not tested	0.0	0.0
SN00776009	SC002981	CCOC(=O)c1c[nH]n(C2CCN(CC2)C(C)c2ccccc2)c1=N	0.0	0.0	not tested	not tested	0.0	0.0
SN00778215	SC007962	O=C(c1ccc(o1)S(=O)(=O)NC(C)(C)C)N1CCc2sccc2C1C	0.0	0.0	not tested	not tested	0.0	0.0
SN00778229	SC007965	O=C(N1CCc2sccc2C1)c1ccc(s1)C(=O)C	0.0	0.0	not tested	not tested	0.0	0.0
SN00778236	SC007965	COC(=O)c1ccc(s1)C(=O)N1CCc2sccc2C1	0.0	0.0	not tested	not tested	0.0	0.0
SN00778256	SC007966	Fc1ccc(cc1)c1ccc(C(=O)N2CCc3sccc3C2C)n1C	0.0	0.0	not tested	not tested	0.0	0.0
SN00778258	SC007972	O=C1CC(CN1c1ccc2OCCOc2c1)C(=O)N1CCc2sccc2C1C	0.0	0.0	not tested	not tested	0.0	0.0
SN00778263	SC007972	COc1cc(OC)c(Cl)cc1N1CC(CC1=O)C(=O)N1CCc2sccc2C1	0.0	0.0	not tested	not tested	0.0	0.0
SN00778894	SC009014	OCCN1CCN(CC1)C(=O)c1cc(C)nc2onc(C)c12	0.0	0.0	not tested	not tested	0.0	0.0
SN00778909	SC009131	CCOc1ccc(cc1)C(=O)Nc1ccc(cc1)c1nnc2CCCCCn12	0.0	0.0	not tested	not tested	0.0	0.0
SN00778917	SC009131	CNS(=O)(=O)c1cc(ccc1Cl)C(=O)Nc1ccc(cc1)c1nnc2CCCCCn12	0.0	0.0	not tested	not tested	0.0	0.0
SN00778983	SC009261	NCc1c(Oc2ccccc2N(=O)=O)n(C)c(=O)n(C)c1=O	0.0	0.0	not tested	not tested	0.0	0.0
SN00779002	SC009261	NCc1c(Oc2ccc(C)cc2Br)n(C)c(=O)n(C)c1=O	0.0	0.0	not tested	not tested	0.0	0.0
SN00779014	SC009333	O=C(CN1CCC(CC1)c1c[nH]c2ccccc12)Nc1ccccc1C	0.0	0.0	not tested	not tested	0.0	0.0
SN00779030	SC009333	Cc1ccc2c([nH]cc2C2CCNCC2)c1	0.0	0.0	not tested	not tested	0.0	0.0
SN00779053	SC009334	OCCN1CCN(CC1)C(=O)c1cnc2onc(C)c2c1	0.0	0.0	not tested	not tested	0.0	0.0
SN00779083	SC009438	O=C(CCCc1nc2ccccc2s1)N1Cc2cc3OCCOc3cc2C2(CCCC2)C1	0.0	0.0	not tested	not tested	0.0	0.0
SN00780083	SC010890	CC1COCCN1Cc1noc(n1)c1ccco1	0.0	0.0	not tested	not tested	0.0	0.0
SN00780093	SC010890	Cc1cccc(c1)c1onc(CN2CCOC(C2)Cn2cncn2)n1	0.0	0.0	not tested	not tested	0.0	0.0
SN00780125	SC010916	O=C(NCc1ccc2OCOc2c1)N1CCCC1c1noc(C)c1	0.0	0.0	not tested	not tested	0.0	0.0
SN00780160	SC010993	NCCCNC(=O)CN1CCCC(CN2CCN(CC2)c2ccccn2)C1	0.0	0.0	not tested	not tested	0.0	0.0
SN00769535	SC000869	Clc1ccc(s1)S(=O)(=O)N1CCN(CC1)C(=O)c1ccc(=O)n(C)n1	0.0	0.0	not tested	not tested	0.0	0.0
SN00769560	SC000884	O=C(N1CCN(CC1)c1ncccn1)c1nc2ccccn2c1	0.0	0.0	not tested	not tested	0.0	0.0
SN00769576	SC000884	O=C(N1CCN(CC1)C1CCS(=O)(=O)C1)c1nc2ccccn2c1	0.0	0.0	not tested	not tested	0.0	0.0
SN00769646	SC000889	Fc1ccc(c(F)c1)C(=O)N1CCN(CC1)C(=O)c1c(C)noc1C	0.0	0.0	not tested	not tested	0.0	0.0
SN00769649	SC000917	O=C(N1CCCC1c1nc2ccccc2s1)c1nn(C)c(=O)c2ccccc12	0.0	0.0	not tested	not tested	0.0	0.0
SN00770646	SC005161	OC1CC2CCC(C1)N2C(=O)Cc1nc2sccn2c1	0.0	0.0	not tested	not tested	0.0	0.0
SN00770662	SC005161	O=C(Nc1cc(ccc1N(C)C)C(F)(F)F)N1C2CCC1CC(C)(O)C2	0.0	0.0	not tested	not tested	0.0	0.0
SN00770682	SC005386	O=c1cc(nc2ccccn12)N1CCN(CC1)S(=O)(=O)C	0.0	0.0	not tested	not tested	0.0	0.0
SN00770693	SC005386	Cc1noc(CN2CCN(CC2)c2cc(=O)n3ccccc3n2)n1	0.0	0.0	not tested	not tested	0.0	0.0
SN00770699	SC005734	c1ccc(cc1)N1CCN(CC1)Cc1ccn(n1)c1ccccc1	0.0	0.0	not tested	not tested	0.0	0.0
SN00770711	SC005734	Fc1cccc(c1)n1ccc(CN2CCN(CC2)c2cnccn2)n1	0.0	0.0	not tested	not tested	0.0	0.0
SN00770727	SC005765	O=C(CSc1n[nH]c(=O)n1C1CC1)N(C)c1ccccc1	0.0	0.0	not tested	not tested	0.0	0.0
SN00770738	SC005765	O=C(NCc1ccccc1)C(C)Sc1n[nH]c(=O)n1C1CC1	0.0	0.0	not tested	not tested	0.0	0.0

Compound ID	Scaffold	Smiles	% Inhibition n=1		% Inhibition n=2		Average % Inhibition	
			24h	48h	24h	48h	24h	48h
SN00770762	SC006354	COc1cc(c(OC)cc1Cl)S(=O)(=O)N1CCN=C1Cc1ccccc1	0.0	0.0	not tested	not tested	0.0	0.0
SN00771799	SC009089	Cc1nc(N)nc(c1)N1CCN(CC1)c1ccc2nncn2n1	0.0	0.0	not tested	not tested	0.0	0.0
SN00771803	SC009089	Cc1nnc2ccc(nn12)N1CCN(CC1)c1ncnc2[nH]ccc12	0.0	0.0	not tested	not tested	0.0	0.0
SN00771815	SC009130	O=C(CCCN1C(=O)c2ccccc2C1=O)N1CCCc2ncccc12	0.0	0.0	not tested	not tested	0.0	0.0
SN00771818	SC009130	Clc1ccc2OCC(=Cc2c1)C(=O)N1CCCc2ncccc12	0.0	0.0	not tested	not tested	0.0	0.0
SN00773636	SC000143	COc1ccc(cc1)CC1(C)NC(=O)N(Cc2c(C)noc2C)C1=O	0.0	0.0	not tested	not tested	0.0	0.0
SN00773812	SC000338	COc1ccc(Br)cc1c1scc(Cn2nnc(n2)c2cccc(F)c2)n1	0.0	0.0	not tested	not tested	0.0	0.0
SN00773861	SC000350	COc1ccc(cc1)n1c(CN2CCN(CC2)C(=O)C(C)C)nc2ccccc12	0.0	0.0	not tested	not tested	0.0	0.0
SN00774939	SC000998	COc1cccc(c1)CC(=O)N1CCC(CC1)c1nc2ccccc2o1	0.0	0.0	not tested	not tested	0.0	0.0
SN00775042	SC001092	COC1CCN(CC1)C(=O)c1cc(nn1c1ccccc1)C1CC1	0.0	0.0	not tested	not tested	0.0	0.0
SN00776174	SC003106	COC(=O)CCc1c(C)nn(c2nc3ccccc3[nH]2)c1O	0.0	0.0	not tested	not tested	0.0	0.0
SN00776180	SC003113	NCCCn1nc(C)c2c1nc(cc2C(F)(F)F)c1cccnc1	0.0	0.0	not tested	not tested	0.0	0.0
SN00776217	SC003124	COc1ccccc1c1noc(COC(=O)c2cc(nc3n(ncc23)C(C)C)c2ccco2)n1	0.0	0.0	not tested	not tested	0.0	0.0
SN00776242	SC003130	CCOc1ccccc1NC(=O)c1cc(nc2onc(C)c12)c1cccs1	0.0	0.0	not tested	not tested	0.0	0.0
SN00777243	SC006867	COc1ccc(cc1)OCC(O)Cn1nnc(n1)c1cccnc1	0.0	0.0	not tested	not tested	0.0	0.0
SN00777248	SC006867	Clc1ccc(COCC(O)Cn2nnc(n2)c2cccnc2)cc1	0.0	0.0	not tested	not tested	0.0	0.0
SN00778341	SC008040	CC(C)COCCCNC(=O)N1CCCC1c1nnc2ccccn12	0.0	0.0	not tested	not tested	0.0	0.0
SN00778372	SC008075	COc1cc(ccc1OC)C(=O)N1CCCC(C1)c1nnc2ccccn12	0.0	0.0	not tested	not tested	0.0	0.0
SN00779070	SC009438	O=C(N1Cc2cc3OCCOc3cc2C2(CCCC2)C1)c1cncn1c1ccccc1	0.0	0.0	not tested	not tested	0.0	0.0
SN00779128	SC009460	Fc1ccc2c(c1)NC(=O)CC2C(=O)N1CCN(CC1)S(=O)(=O)c1cc(C)ccc1C	0.0	0.0	not tested	not tested	0.0	0.0
SN00779156	SC009463	Cc1onc(n1)C1(CCCCC1)NC(=O)Cc1csc(n1)c1cccs1	0.0	0.0	not tested	not tested	0.0	0.0
SN00779158	SC009463	Cc1onc(n1)C1(CCCCC1)NC(=O)CCc1ncc(o1)c1ccccc1F	0.0	0.0	not tested	not tested	0.0	0.0
SN00780109	SC010916	O=C(NC(=O)N)CN1CCCC1c1noc(C)c1	0.0	0.0	not tested	not tested	0.0	0.0
SN00780138	SC010992	O=C(CCNC(=O)c1ccco1)N1CCCCC1CN1CCOCC1	0.0	0.0	not tested	not tested	0.0	0.0
SN00780144	SC010992	Fc1cccc2[nH]c(CN3CCCCC3CN3CCOCC3)nc12	0.0	0.0	not tested	not tested	0.0	0.0
SN00780218	SC011003	CCN(CC)CCCN1C(c2ccccn2)C(=C(O)C1=O)C(=O)c1ccc(C)o1	0.0	0.0	not tested	not tested	0.0	0.0
SN00780225	SC011003	CCOc1ccc(cc1)C(=O)C1=C(O)C(=O)N(CCCN2CCOCC2)C1c1ccccn1	0.0	0.0	not tested	not tested	0.0	0.0
SN00780242	SC011004	COc1cc2CCN(CCc2cc1OC)C(=O)Cc1c(C)onc1C	0.0	0.0	not tested	not tested	0.0	0.0
SN00780247	SC011004	COc1cc2CCN(CCc2cc1OC)C(=O)Cn1nc(C)cc1C	0.0	0.0	not tested	not tested	0.0	0.0
SN00769333	SC000256	O=C(NNC(=O)c1c[nH]c2ccccc12)CN1C(=O)NC(C)(C2CC2)C1=O	0.0	0.0	not tested	not tested	0.0	0.0
SN00769337	SC000256	CC(C)CC(CNC(=O)CN1C(=O)NC(C)(C2CC2)C1=O)N1CCOCC1	0.0	0.0	not tested	not tested	0.0	0.0
SN00769347	SC000304	O=C1NC(Cc2c[nH]c3ccccc23)C(=O)N1CC(=O)N(C(C)C)c1ccccc1	0.0	0.0	not tested	not tested	0.0	0.0
SN00771559	SC008739	Cc1[nH]ncc1C(=O)N1CCCN(CC1)Cc1nccn1C(C)C	0.0	0.0	not tested	not tested	0.0	0.0
SN00771590	SC008751	CC(C)N(Cc1nc2ccccc2c(=O)[nH]1)CC(=O)N1c2ccccc2NC(=O)C21CCCC2	0.0	0.0	not tested	not tested	0.0	0.0
SN00771605	SC008829	Fc1ccc(cc1)NC(=O)c1nnc(s1)CN1CCCC1c1ccccc1	0.0	0.0	not tested	not tested	0.0	0.0
SN00771615	SC008829	CCOc1nnc(CN2CCCC2c2cnn(C)c2)s1	0.0	0.0	not tested	not tested	0.0	0.0
SN00771862	SC009212	COc1ccccc1COCC(O)CN1CCN(CC1)Cc1nnc2CCCCCn12	0.0	0.0	not tested	not tested	0.0	0.0
SN00771896	SC009223	O=c1cc(ccn1C)CN1CCCC1c1nnc2CCCCCn12	0.0	0.0	not tested	not tested	0.0	0.0
SN00772860	SC013920	Fc1cccc(c1)n1ccc(CN2CCCC2Cn2cncn2)n1	0.0	0.0	not tested	not tested	0.0	0.0
SN00772874	SC014149	O=C(Nc1ncn(n1)Cc1ccccn1)c1cc2sc3ccccc3c2s1	0.0	0.0	not tested	not tested	0.0	0.0
SN00772879	SC014149	O=C(Nc1ncn(n1)Cc1ccccn1)c1ccc(Cl)c(Cl)c1	0.0	0.0	not tested	not tested	0.0	0.0
SN00772920	SC014250	COc1ccc(c2noc(n2)C2CCOCC2)c(OC)c1	0.0	0.0	not tested	not tested	0.0	0.0
SN00772928	SC014342	Clc1ccc(cc1)CC(=O)N1CCCC1c1nncn1CC(C)C	0.0	0.0	not tested	not tested	0.0	0.0
SN00772944	SC014592	Cc1ccc(cc1C)N1C(=O)CS(=O)(=O)C21C(=O)Nc1ccccc21	0.0	0.0	not tested	not tested	0.0	0.0
SN00772961	SC014592	Cc1cccc(c1)CN1c2ccccc2C2(C1=O)N(c1cc(C)cc(C)c1)C(=O)CS2(=O)=O	0.0	0.0	not tested	not tested	0.0	0.0

Compound ID	Scaffold	Smiles	% Inhibition n=1		% Inhibition n=2		Average % Inhibition	
			24h	48h	24h	48h	24h	48h
SN00773632	SC000143	O=C1NC(C)(c2cc3ccccc3o2)C(=O)N1Cc1c(C)onc1C	0.0	0.0	not tested	not tested	0.0	0.0
SN00773644	SC000195	O=c1cc(Cn2cc(ccc2=O)C(F)(F)F)c2ccc(C)c(C)c2o1	0.0	0.0	not tested	not tested	0.0	0.0
SN00773647	SC000195	O=c1cc(Cn2cc(cc(Cl)c2=O)C(F)(F)F)c2ccc(O)c(C)c2o1	0.0	0.0	not tested	not tested	0.0	0.0
SN00773893	SC000384	CCN(C(=O)CN1C(=O)NC(C)(c2ccc3OCOc3c2)C1=O)C1CCS(=O)(=O)C1	0.0	0.0	not tested	not tested	0.0	0.0
SN00776183	SC003113	OC(=O)c1cc(nc2c1cnn2C(C)C1CC1)c1cccnc1	0.0	0.0	not tested	not tested	0.0	0.0
SN00776227	SC003124	COc1ccc(OC)cc1NC(=O)c1cc(nc2n(C)nc(C)c12)c1ccco1	0.0	0.0	not tested	not tested	0.0	0.0
SN00777268	SC006996	O=C1Nc2ccccc2N(C(C)C1)C(=O)c1cccc(c1)S(=O)(=O)N(C)C	0.0	0.0	not tested	not tested	0.0	0.0
SN00777308	SC007036	O=C(Nc1cccc(c1)N(=O)=O)N1CCNC(=O)C1c1c(C)nn(c2ccccc2)c1C	0.0	0.0	not tested	not tested	0.0	0.0
SN00777311	SC007036	Cc1oc(nc1CN1CCNC(=O)C1c1c(C)nn(c2ccccc2)c1C)c1cccs1	0.0	0.0	not tested	not tested	0.0	0.0
SN00777323	SC007047	O=S(=O)(N1CCN(CC1)Cc1c(nc2sccn12)c1ccccc1)c1cccs1	0.0	0.0	not tested	not tested	0.0	0.0
SN00777325	SC007047	O=S(=O)(N1CCN(CC1)Cc1c(nc2sccn12)c1ccccc1)c1ccc2OCCOc2c1	0.0	0.0	not tested	not tested	0.0	0.0
SN00777359	SC007139	Cc1nn(Cc2noc(c2)c2ccco2)c(C)c1S(=O)(=O)N1CCc2ccccc12	0.0	0.0	not tested	not tested	0.0	0.0
SN00777364	SC007139	c1ccc(cc1)c1onc(Cn2ncc(c2)c2ccccc2)c1	0.0	0.0	not tested	not tested	0.0	0.0
SN00778162	SC007870	CCOC(=O)N1CCCN(CC1)C(=O)c1nn(cc1OC)c1ccccc1	0.0	0.0	not tested	not tested	0.0	0.0
SN00778173	SC007879	Brc1ccccc1SCc1noc(n1)c1cccs1	0.0	0.0	not tested	not tested	0.0	0.0
SN00778191	SC007879	O=C1c2ccccc2S(=O)(=O)N1Cc1noc(n1)c1cccs1	0.0	0.0	not tested	not tested	0.0	0.0
SN00778407	SC008076	CCN(Cc1cccs1)C(=O)CN1CCC(CC1)n1c(=O)[nH]c2ccccc12	0.0	0.0	not tested	not tested	0.0	0.0
SN00778427	SC008076	CCCNC(=O)N1CCC(CC1)n1c(=O)[nH]c2ccccc12	0.0	0.0	not tested	not tested	0.0	0.0
SN00779099	SC009453	Cn1ncc(c2onc(n2)C2CC2)c1N	0.0	0.0	not tested	not tested	0.0	0.0
SN00779105	SC009453	Fc1cccc(c1)C(=O)Nc1n(C)ncc1c1onc(n1)C1CC1	0.0	0.0	not tested	not tested	0.0	0.0
SN00779883	SC010604	OC(=O)Cn1nnc(n1)c1ccco1	0.0	0.0	not tested	not tested	0.0	0.0
SN00779890	SC010604	Fc1ccc(CCNC(=O)Cn2nnc(n2)c2ccco2)cc1	0.0	0.0	not tested	not tested	0.0	0.0
SN00779908	SC010648	O=C(N1CCC(CC1)c1ccccc1)c1cnn2cccnc12	0.0	0.0	not tested	not tested	0.0	0.0
SN00779929	SC010648	Cc1ccn2ncc(C(=O)N3CCC(CC3)Nc3cccnn3)c2n1	0.0	0.0	not tested	not tested	0.0	0.0
SN00779951	SC010659	O=c1n(cnc2ccccc12)Cc1noc(n1)c1ccco1	0.0	0.0	not tested	not tested	0.0	0.0
SN00772677	SC013434	CCS(=O)(=O)N1CCC(CC1)c1[nH]nc(n1)c1ccccc1	0.0	0.0	not tested	not tested	0.0	0.0
SN00772684	SC013514	Clc1ccc(cc1)NC(=O)Cn1nccc1c1ccncc1	0.0	0.0	not tested	not tested	0.0	0.0
SN00772703	SC013592	CCc1onc(n1)Cn1c(=O)onc1c1ccccc1C	0.0	0.0	not tested	not tested	0.0	0.0
SN00772717	SC013670	c1ncn(n1)CC1CCCN(C1)c1nc2ccccc2o1	0.0	0.0	not tested	not tested	0.0	0.0
SN00773362	SC015775	Cc1nn2CCN(Cc2n1)S(=O)(=O)c1cc(F)c(F)cc1F	0.0	0.0	not tested	not tested	0.0	0.0
SN00773383	SC015832	COc1cccc(c1)c1nnc(CN2CC3C4CCC(C4)C23)o1	0.0	0.0	not tested	not tested	0.0	0.0
SN00773606	SC000069	CCCc1scc(n1)CN1C(=O)NC(C)(c2ccc(C)o2)C1=O	0.0	0.0	not tested	not tested	0.0	0.0
SN00773910	SC000384	Cc1ccc(cc1)n1nc(C)cc1NC(=O)CN1C(=O)NC(C)(c2ccc3OCOc3c2)C1=O	0.0	0.0	not tested	not tested	0.0	0.0
SN00773937	SC000646	CCCCn1c2ncn(C3CCCC3)c2c(=O)n(CC(=O)Nc2sccc2C(=O)OCC)c1=O	0.0	0.0	not tested	not tested	0.0	0.0
SN00774292	SC000847	NCc1c(=O)n(CC(=O)Nc2ccc3OCCOc3c2)c(=O)n2CCCCCc12	0.0	0.0	not tested	not tested	0.0	0.0
SN00775028	SC001048	CCc1ccccc1NC(=O)CN1CCN(CC1)C(=O)c1cnn(n1)c1ccccc1	0.0	0.0	not tested	not tested	0.0	0.0
SN00775440	SC001437	Cn1ncc(N2CCCC(C2)c2nnc[nH]2)c(Cl)c1=O	0.0	0.0	not tested	not tested	0.0	0.0
SN00775472	SC001734	O=C(C1CCC1)N1CCCC(C1)C(F)(F)F	0.0	0.0	not tested	not tested	0.0	0.0
SN00775476	SC001734	Clc1cccc(c1)C1(CCC1)C(=O)N1CCCC(C1)NS(=O)(=O)C	0.0	0.0	not tested	not tested	0.0	0.0
SN00775915	SC002935	O=C(N1CCCC1c1cccs1)c1cc(nc2n(ncc12)Cc1ccc2OCOc2c1)c1ccco1	0.0	0.0	not tested	not tested	0.0	0.0
SN00775923	SC002935	O=C(N1CCCC1c1nc2ccccc2s1)c1cc(nc2n(C)nc(C)c12)c1ccccc1	0.0	0.0	not tested	not tested	0.0	0.0
SN00775970	SC002978	CN1CCC(CC1)n1nccc1NC(=O)C(NC(=O)Nc1ccccc1)C(C)C	0.0	0.0	not tested	not tested	0.0	0.0
SN00776546	SC005037	CCCCCCn1c(nc2c1c(=O)n(C)c(=O)n2C)N1CCOCC1	0.0	0.0	not tested	not tested	0.0	0.0
SN00776568	SC005151	O=C(Nc1cccc(c1)N(=O)=O)C(C)N1CCN(CC1)c1nc2ccccc2s1	0.0	0.0	not tested	not tested	0.0	0.0
SN00776584	SC005151	CC(O)CN1CCN(CC1C)c1nc2ccccc2s1	0.0	0.0	not tested	not tested	0.0	0.0
SN00776586	SC005367	Clc1cccc(c1)n1nnnc1C1(CCCCC1)N1CCCCC1	0.0	0.0	not tested	not tested	0.0	0.0

Compound ID	Scaffold	Smiles	% Inhibition n=1		% Inhibition n=2		Average % Inhibition	
			24h	48h	24h	48h	24h	48h
SN00777063	SC006676	CCCc1scc(n1)c1ccc2c(CCCN2S(=O)(=O)C)c1	0.0	0.0	not tested	not tested	0.0	0.0
SN00777069	SC006680	Fc1ccc(cc1)n1cncc1C(=O)N1CCC(CC1)n1c(=O)[nH]c2ccccc12	0.0	0.0	not tested	not tested	0.0	0.0
SN00777663	SC007568	Cc1onc(c1)CN1CCCCC1Cn1ncc(C)c1	0.0	0.0	not tested	not tested	0.0	0.0
SN00777677	SC007572	Cc1onc(n1)C1CCCN1Cc1c(C)noc1C	0.0	0.0	not tested	not tested	0.0	0.0
SN00777708	SC007575	Cc1nn(CC2CCCN2Cc2nc(oc2C)c2ccco2)c(C)n1	0.0	0.0	not tested	not tested	0.0	0.0
SN00777718	SC007577	COc1cc(OC)ccc1C1CCCN1Cc1noc(c1)c1cccs1	0.0	0.0	not tested	not tested	0.0	0.0
SN00777791	SC007616	COc1ccc(Cl)cc1Cn1nnn(c2ccccc2Cl)c1=O	0.0	0.0	not tested	not tested	0.0	0.0
SN00778785	SC008766	O=C(Cc1c[nH]c2ccccc12)N1CCN(CC1)S(=O)(=O)c1cn(C)c(c1)C(=O)N	0.0	0.0	not tested	not tested	0.0	0.0
SN00778803	SC008766	Cn1ccc(c1)S(=O)(=O)N1CCNCC1	0.0	0.0	not tested	not tested	0.0	0.0
SN00769387	SC000391	Cc1ccc(cc1)OCc1scc(n1)CN1C(=O)N(C(C)C)C(=O)C1=O	0.0	0.0	not tested	not tested	0.0	0.0
SN00769406	SC000433	O=C1NC2(CCSC2)C(=O)N1Cc1ccccc1F	0.0	0.0	not tested	not tested	0.0	0.0
SN00769421	SC000433	O=C1NC2(CCSC2)C(=O)N1Cc1ccc2ccccc2n1	0.0	0.0	not tested	not tested	0.0	0.0
SN00770448	SC002915	NCc1c(=O)n(CC(=O)N2CCN(CC2)S(=O)(=O)c2ccc(C)cc2)c(=O)n2CCCc12	0.0	0.0	not tested	not tested	0.0	0.0
SN00770455	SC002915	NCc1c(=O)n(CC(=O)N2C(C)Cc3ccccc23)c(=O)n2CCCc12	0.0	0.0	not tested	not tested	0.0	0.0
SN00772022	SC010365	Clc1ccc2c(c1)nc(CN1CCc3nc(C)ncc3C1)n2C	0.0	0.0	not tested	not tested	0.0	0.0
SN00773081	SC015169	CCc1nnc2CN(CCn12)C(=O)c1cnn(c1)C(C)(C)C	0.0	0.0	not tested	not tested	0.0	0.0
SN00773093	SC015175	Brc1c[nH]c(c1)C(=O)N1CCn2c(nnc2C)C1	0.0	0.0	not tested	not tested	0.0	0.0
SN00773108	SC015175	CCc1nnc2CN(CCn12)C(=O)c1cc(Cl)c(Cl)n1C	0.0	0.0	not tested	not tested	0.0	0.0
SN00773365	SC015775	Clc1ccc(cc1)S(=O)(=O)N1CCn2nc(C)nc2C1	0.0	0.0	not tested	not tested	0.0	0.0
SN00773401	SC016053	O=C1NC(=O)C2(CCN(C2)C(=O)CCn2ccc3ccccc23)N1	0.0	0.0	not tested	not tested	0.0	0.0
SN00774299	SC000847	NCc1c(=O)n(CC(=O)NCC(F)(F)F)c(=O)n2CCCCCc12	0.0	0.0	not tested	not tested	0.0	0.0
SN00774330	SC000859	COc1ccc(cc1)CN(C1CC1)C(=O)c1cc(O)nc2ccc(cc12)S(=O)(=O)N1CCOCC1	0.0	0.0	not tested	not tested	0.0	0.0
SN00774333	SC000859	Oc1nc2ccc(cc2c(c1)C(=O)Nc1cccc(c1)C(=O)NC1CC1)S(=O)(=O)N1CCOCC1	0.0	0.0	not tested	not tested	0.0	0.0
SN00775143	SC001114	Cc1ccc2c(CCCN2C(=O)C2CCN(CC2)C(=O)OC(C)(C)C)c1	0.0	0.0	not tested	not tested	0.0	0.0
SN00775501	SC001740	O=C1NCCN(C1)C(=O)c1cccnc1Nc1ccccc1F	0.0	0.0	not tested	not tested	0.0	0.0
SN00777453	SC007315	CC1CCC(CC1)NC(=O)C1CCN(CC1)Cc1nccn1C	0.0	0.0	not tested	not tested	0.0	0.0
SN00777456	SC007315	O=C(NC(C1CC1)c1ccccc1)C1CCN(CC1)Cc1nccn1C	0.0	0.0	not tested	not tested	0.0	0.0
SN00778829	SC008770	Cc1ccc(cc1)S(=O)(=O)C1(CCC1)C(=O)N(C)Cc1cccc2ccccc12	0.0	0.0	not tested	not tested	0.0	0.0
SN00778870	SC008972	Fc1ccc(cc1)C(C)(C)NCn1nc(C)c2c(C)onc2c1=O	0.0	0.0	not tested	not tested	0.0	0.0
SN00778892	SC009014	Cc1nc2onc(C)c2c(c1)C(=O)N1CCN(CC1)c1ncnc2n(C)ncc12	0.0	0.0	not tested	not tested	0.0	0.0
SN00779218	SC009471	C=CCn1c(nc2c1c(=O)[nH]c(=O)n2C)N1CCCC1	0.0	0.0	not tested	not tested	0.0	0.0
SN00779222	SC009471	Cn1c(=O)[nH]c(=O)c2c1nc(N1CCCC1)n2Cc1ccccc1	0.0	0.0	not tested	not tested	0.0	0.0
SN00779444	SC010002	CCCCC(=O)N1CCN(CC1)S(=O)(=O)c1cc2OCC(=O)Nc2cc1Cl	0.0	0.0	not tested	not tested	0.0	0.0
SN00779453	SC010002	O=C1COc2cc(c(Cl)cc2N1)S(=O)(=O)N1CCN(CC1)C(=O)C(C)(C)C	0.0	0.0	not tested	not tested	0.0	0.0
SN00779516	SC010132	CC1CCCN(C1)Cc1[nH]c(=O)c2cnn(c3ccccc3)c2n1	0.0	0.0	not tested	not tested	0.0	0.0
SN00779956	SC010659	O=C(NCc1noc(n1)c1ccco1)c1ccn(C)c(=O)c1	0.0	0.0	not tested	not tested	0.0	0.0
SN00779967	SC010660	Clc1ccccc1c1onc(n1)Cn1cnc2sc(c3ccccc3)c(C)c2c1=O	0.0	0.0	not tested	not tested	0.0	0.0
SN00780281	SC011097	CCN(CC)CCN1C(c2ccccc2)C(=C(O)C1=O)C(=O)c1c(C)[nH]c(C(=O)OC)c1C	0.0	0.0	not tested	not tested	0.0	0.0
SN00780308	SC011120	CN(Cc1onc(n1)c1ccc2OCOc2c1)CC(=O)NC(C)(C)C	0.0	0.0	not tested	not tested	0.0	0.0
SN00780317	SC011120	CC(NCc1onc(n1)c1ccc2OCOc2c1)Cn1ncc(C)c1	0.0	0.0	not tested	not tested	0.0	0.0
SN00769754	SC001187	CN1CCCC1C1CCCN1C(=O)c1[nH]nc(c1)c1cccs1	0.0	0.0	not tested	not tested	0.0	0.0
SN00769760	SC001187	CCc1onc(n1)C1CCCN1C(=O)c1cc(C)nn1CC	0.0	0.0	not tested	not tested	0.0	0.0
SN00770041	SC001404	O=C(CCn1[nH]c(=O)c2ccccc2c1=O)Nc1ccc2c(c1)nc1CCCCCn21	0.0	0.0	not tested	not tested	0.0	0.0
SN00771150	SC007741	Cn1ncc(CN2CCCN(CC2)C(=O)Nc2nnc(s2)C(C)(C)c2ccccc2)c1	0.0	0.0	not tested	not tested	0.0	0.0
SN00771162	SC007812	O=C(Nc1ccc(nc1)N1CCOCC1)N1CCN(Cc2ccccc2)C2(CCCCC2)C1	0.0	0.0	not tested	not tested	0.0	0.0
SN00771163	SC007812	CN1CCN(C(=O)CC2C=CCC2)C2(CCCCC2)C1	0.0	0.0	not tested	not tested	0.0	0.0

Compound ID	Scaffold	Smiles	% Inhibition n=1		% Inhibition n=2		Average % Inhibition	
			24h	48h	24h	48h	24h	48h
SN00772140	SC011466	CCn1cnnc1C1CCN(CC1)C(=O)C1CCOc2ccccc12	0.0	0.0	not tested	not tested	0.0	0.0
SN00772147	SC011506	CCCOc1c(Br)cc(cc1OC)C(=O)N1CCCC(C1)C1NC(=O)NC1=O	0.0	0.0	not tested	not tested	0.0	0.0
SN00772172	SC011506	O=C1NC(=O)C(C)(N1)C1CCCN(C1)C(=O)c1cc(ccc1C)S(=O)(=O)NC1CC1	0.0	0.0	not tested	not tested	0.0	0.0
SN00773140	SC015211	c1scc(c1)c1scc(CN2CCn3c(nnc3C3CC3)C2)n1	0.0	0.0	not tested	not tested	0.0	0.0
SN00773159	SC015213	CC(C)c1nnc(CN2CCn3c(nnc3C(C)C)C2)o1	0.0	0.0	not tested	not tested	0.0	0.0
SN00773179	SC015216	Cc1nnc2CN(CCn12)Cc1onc(n1)c1ccc2OCOc2c1	0.0	0.0	not tested	not tested	0.0	0.0
SN00775153	SC001114	O=C(/C=C/c1ccccc1)N1CCC(CC1)C(=O)N1CCCc2cccc(F)c12	0.0	0.0	not tested	not tested	0.0	0.0
SN00775190	SC001115	Fc1cc(F)c2c(CCCN2C(=O)C2CN(C(C)C)C(=O)C2)c1	0.0	0.0	not tested	not tested	0.0	0.0
SN00776414	SC003252	c1ccc(cc1)OCc1nn2cc(c3ccc4OCOc4c3)c3CCCCn1c23	0.0	0.0	not tested	not tested	0.0	0.0
SN00777520	SC007474	FC(F)(F)c1nc2ccccc2n1Cc1noc(c1)c1cccs1	0.0	0.0	not tested	not tested	0.0	0.0
SN00777534	SC007482	O=C(OCc1onc(n1)c1ccccc1Cl)c1ccc2c(c1)nc1CCCCCn1c2=O	0.0	0.0	not tested	not tested	0.0	0.0
SN00780600	SC011739	Cc1n[nH]c(=O)c(C(=O)N2CCN(CC2)CC2CCCCC2)c1C	0.0	0.0	not tested	not tested	0.0	0.0
SN00780606	SC011776	Cc1onc(n1)C1(CCC1)NS(=O)(=O)c1ccc2CCCc2c1	0.0	0.0	not tested	not tested	0.0	0.0
SN00780613	SC011776	Cc1onc(n1)C1(CCC1)NCc1c(C)nn(C)c1N(C)C	0.0	0.0	not tested	not tested	0.0	0.0
SN00769811	SC001283	NC/C(=C\c1cccs1)/c1scc(n1)c1cc2ccccc2oc1=O	0.0	0.0	not tested	not tested	0.0	0.0
SN00770808	SC006623	O=C(C1CCN(CC1)C(=O)Nc1ccccc1)N1CCCN(CC1)c1ccc(cn1)C(F)(F)F	0.0	0.0	not tested	not tested	0.0	0.0
SN00770829	SC006623	O=C(N1CCCN(CC1)c1nccs1)C1CCN(CC1)C(=O)C	0.0	0.0	not tested	not tested	0.0	0.0
SN00770842	SC006642	O=C1CC2(CCCC2)C(=O)N1CN1CCN(CC1)S(=O)(=O)c1cccs1	0.0	0.0	not tested	not tested	0.0	0.0
SN00770846	SC006642	Brc1ccc(s1)S(=O)(=O)N1CCN(CC1)CN1C(=O)CC2(CCCC2)C1=O	0.0	0.0	not tested	not tested	0.0	0.0
SN00770880	SC006746	O=C(N1CCN(CC1)c1cnccn1)C1(CCOCC1)c1ccccc1	0.0	0.0	not tested	not tested	0.0	0.0
SN00770891	SC006746	OC(CN1CCN(CC1)C(=O)C1(C)CCOCC1)C(C)(C)C	0.0	0.0	not tested	not tested	0.0	0.0
SN00770900	SC006748	O=c1cc(C)n(nc1C(=O)N1CCN(CC1)c1ccc(cn1)C(F)(F)F)c1ccccc1F	0.0	0.0	not tested	not tested	0.0	0.0
SN00770916	SC006748	Clc1ccc(cc1)n1nc(c(=O)cc1C)C(=O)N1CCN(CC1)C(=O)C	0.0	0.0	not tested	not tested	0.0	0.0
SN00771971	SC010012	CCOc1ccc(cc1)CN1CCN(CC1)C(=O)c1nnn(Cc2ccccc2)c1	0.0	0.0	not tested	not tested	0.0	0.0
SN00771975	SC010012	O=C(N1CCN(CC1)c1ccccc1F)c1nnn(c1)c1ccccc1	0.0	0.0	not tested	not tested	0.0	0.0
SN00772009	SC010365	c1ccc(cc1)c1ncc2CN(CCc2n1)c1ncnc2sc3CCCc3c12	0.0	0.0	not tested	not tested	0.0	0.0
SN00772073	SC010766	CCOc1ccccc1N1C(=O)C(=O)N(Cc2noc(C)n2)C1=O	0.0	0.0	not tested	not tested	0.0	0.0
SN00773221	SC015354	CC1CN(CCS(=O)(=O)C)CC1N1CCN(CC1)C(=O)C	0.0	0.0	not tested	not tested	0.0	0.0
SN00773269	SC015438	O=C(C1CC1)N1CCCCCC1C1CCN(CC1)Cc1noc(n1)C(C)(C)C	0.0	0.0	not tested	not tested	0.0	0.0
SN00774037	SC000748	COc1ccc(cc1)c1noc(n1)CN1C(=O)NC(C)(C1=O)c1ccccc1Cl	0.0	0.0	not tested	not tested	0.0	0.0
SN00774072	SC000758	O=C1NC(C)(c2ccco2)C(=O)N1Cc1cn(nc1c1ccccc1)c1ccccc1	0.0	0.0	not tested	not tested	0.0	0.0
SN00774132	SC000772	Cc1oc(nc1Cn1cccn1)c1cccs1	0.0	0.0	not tested	not tested	0.0	0.0
SN00774164	SC000773	CN1CCCCC1C(=O)Nc1cnn(c1)Cc1nc2ccccn2c1	0.0	0.0	not tested	not tested	0.0	0.0
SN00774207	SC000840	Cc1ccc(cc1)N1C(=O)C(=O)N(CC(=O)c2ccc(Cl)s2)C1=O	0.0	0.0	not tested	not tested	0.0	0.0
SN00774209	SC000840	COc1ccc(cc1)N1C(=O)NC(=O)C1=O	0.0	0.0	not tested	not tested	0.0	0.0
SN00775130	SC001100	COc1cc(ccc1OC)N1CC(CC1=O)C(=O)N1CCc2ccccc12	0.0	0.0	not tested	not tested	0.0	0.0
SN00775200	SC001166	CC1OC(C)CN(C1)C(=O)c1ccc(=O)n(n1)c1ccccc1	0.0	0.0	not tested	not tested	0.0	0.0
SN00775226	SC001182	O=C(C1CC1)N1CCN(CC1)C(=O)c1ccc2[nH]c(C)c(C)c2c1	0.0	0.0	not tested	not tested	0.0	0.0
SN00775276	SC001236	CC1OC(C)CN(C1)C(=O)c1cc(nc2n(ncc12)C(C)C)C1CC1	0.0	0.0	not tested	not tested	0.0	0.0
SN00775298	SC001236	CCC1OCCN(C1)C(=O)c1cc(nc2c1cnn2C(C)C)C1CC1	0.0	0.0	not tested	not tested	0.0	0.0
SN00775318	SC001261	CN(C)C(=O)C1Oc2ccccc2N(C1)C(=O)C1CCN(CC1)C1=NS(=O)(=O)c2ccccc12	0.0	0.0	not tested	not tested	0.0	0.0
SN00778522	SC008248	CC(C)CN1CCCN(CC1)C(=O)c1cnc(s1)c1cnn(C)c1	0.0	0.0	not tested	not tested	0.0	0.0
SN00778610	SC008596	CCN(CC)S(=O)(=O)c1ccc(=O)n(Cc2noc(n2)c2ccc(F)cc2)c1	0.0	0.0	not tested	not tested	0.0	0.0
SN00778624	SC008596	CCCc1onc(n1)Cn1cccc(c1=O)C(F)(F)F	0.0	0.0	not tested	not tested	0.0	0.0
SN00778647	SC008615	Cc1onc(n1)Cn1nnc(n1)c1ccc(cc1)C(C)C	0.0	0.0	not tested	not tested	0.0	0.0
SN00778650	SC008615	Fc1cccc(c1)c1nnn(Cc2noc(n2)C(C)(C)C)n1	0.0	0.0	not tested	not tested	0.0	0.0

Compound ID	Scaffold	Smiles	% Inhibition n=1		% Inhibition n=2		Average % Inhibition	
			24h	48h	24h	48h	24h	48h
SN00778666	SC008643	OC(COCc1ccccc1)CN1C(=O)C2CCCCN2C1=O	0.0	0.0	not tested	not tested	0.0	0.0
SN00778676	SC008643	OC(COc1cc(C)ccc1C)CN1C(=O)C2CCCCN2C1=O	0.0	0.0	not tested	not tested	0.0	0.0
SN00779254	SC009478	O=c1c2c(nc3oc(cn23)C(C)(C)C)n(C)c(=O)n1CCN1CCCCC1	0.0	0.0	not tested	not tested	0.0	0.0
SN00779274	SC009635	O=C(N1CCC(C1)c1ccccc1C)c1ccc(=O)n(C)c1	0.0	0.0	not tested	not tested	0.0	0.0
SN00779290	SC009701	Cc1nn(Cc2onc(C)n2)c(n1)c1ccc(Cl)c(Cl)c1	0.0	0.0	not tested	not tested	0.0	0.0
SN00779305	SC009912	O=C(NCC(F)(F)F)C1CCCN1c1nn2cc(nc2s1)c1ccccc1	0.0	0.0	not tested	not tested	0.0	0.0
SN00779315	SC009912	CCc1onc(n1)C1CCCN1c1nn2cc(C)nc2s1	0.0	0.0	not tested	not tested	0.0	0.0
SN00779341	SC009938	CCNC(=O)N1CCCC(CN2CCCCCC2)C1	0.0	0.0	not tested	not tested	0.0	0.0
SN00779347	SC009940	CSc1ccc(C)c(c1)C(=O)N1CCCC(CN2CCOCC2)C1	0.0	0.0	not tested	not tested	0.0	0.0
SN00780351	SC011156	O=C(CSc1nnc(c(=O)[nH]1)c1cc(Cl)sc1Cl)NCc1ccccc1F	0.0	0.0	not tested	not tested	0.0	0.0
SN00780363	SC011167	CCNC(=O)N1CCN(C)c2ccccc2C1	0.0	0.0	not tested	not tested	0.0	0.0
SN00780367	SC011167	O=C(N1CCN(C)c2ccccc2C1)c1ccc2[nH]cnc2c1	0.0	0.0	not tested	not tested	0.0	0.0
SN00780385	SC011234	C1CCN2CCN(CC2C1)c1nc2ccccc2o1	0.0	0.0	not tested	not tested	0.0	0.0
SN00780389	SC011234	O=C(Cn1nnc(n1)c1ccccc1)N1CCN2CCCCC2C1	0.0	0.0	not tested	not tested	0.0	0.0
SN00780410	SC011296	O=C(CN(C)S(=O)(=O)C)N1CCCCC1c1cc2ccccc2[nH]1	0.0	0.0	not tested	not tested	0.0	0.0
SN00780417	SC011296	O=C1CSCN1CC(=O)N1CCCCC1c1cc2ccccc2[nH]1	0.0	0.0	not tested	not tested	0.0	0.0
SN00769662	SC000917	CCOc1ccc(cc1)C1CCCN1C(=O)c1nn(CC)c(=O)c2ccccc12	0.0	0.0	not tested	not tested	0.0	0.0
SN00769685	SC000925	CCc1ccccc1NC(=O)CN1CCN(CC1)C(=O)c1nc(c2ccccc2)n(n1)c1ccccc1	0.0	0.0	not tested	not tested	0.0	0.0
SN00769833	SC001309	COc1cccc(c1)C(=O)N1CCN(CC1)CC1CCCO1	0.0	0.0	not tested	not tested	0.0	0.0
SN00769852	SC001321	Fc1ccc(cc1)n1nc(nc1C)C(=O)N1CCCC1c1nc2ccccc2[nH]1	0.0	0.0	not tested	not tested	0.0	0.0
SN00769862	SC001321	CCc1nc(nn1c1c(Cl)cccc1Cl)C(=O)N1CCCC1	0.0	0.0	not tested	not tested	0.0	0.0
SN00770791	SC006546	Cn1ncc(C(=O)NCCn2c(C)nc3ccccc23)c1n1cccc1	0.0	0.0	not tested	not tested	0.0	0.0
SN00770972	SC007099	CN1CCN(CCNC(=O)c2scnc2C)C(C1)c1ccccc1	0.0	0.0	not tested	not tested	0.0	0.0
SN00771906	SC009352	COc1ccc(cc1)S(=O)(=O)N1CCC(CC1)Nc1ncnc2n(C)ncc12	0.0	0.0	not tested	not tested	0.0	0.0
SN00772058	SC010766	CCN1C(=O)C(=O)N(Cc2noc(n2)c2cccc(C)c2)C1=O	0.0	0.0	not tested	not tested	0.0	0.0
SN00772100	SC011460	O=C(c1cocc1)N1CCCCC1c1onc(n1)c1ccccc1	0.0	0.0	not tested	not tested	0.0	0.0
SN00773028	SC014895	Clc1ccc2ncn(Cc3noc(n3)c3cocc3)c(=O)c2c1	0.0	0.0	not tested	not tested	0.0	0.0
SN00774018	SC000746	Cc1ccc(cc1)N1C(=O)N(Cc2ncc(o2)C(C)(C)C)C(=O)C1C	0.0	0.0	not tested	not tested	0.0	0.0
SN00776253	SC003130	Cc1ccc(s1)c1nc2onc(C)c2c(c1)C(=O)NC1CC1	0.0	0.0	not tested	not tested	0.0	0.0
SN00776279	SC003131	CC(C)C(NC(=O)c1cnc2c(c1)c(C)nn2c1ccccn1)c1ccccc1	0.0	0.0	not tested	not tested	0.0	0.0
SN00776281	SC003131	O=C(NCC(N(C)C)c1ccccc1Cl)c1cnc2c(c1)c(C)nn2c1ccccn1	0.0	0.0	not tested	not tested	0.0	0.0
SN00776306	SC003136	Clc1ccc(cc1)c1csc(n1)C1=C(N)N(CCc2ccccc2)CC1=O	0.0	0.0	not tested	not tested	0.0	0.0
SN00777385	SC007224	OCCn1ccc(Nc2ccc(cc2)S(=O)(=O)C(F)(F)F)n1	0.0	0.0	not tested	not tested	0.0	0.0
SN00777435	SC007286	CCCn1nnnc1CN1C(=O)NC(C)(Cc2ccc3OCOc3c2)C1=O	0.0	0.0	not tested	not tested	0.0	0.0
SN00777493	SC007383	COc1ccc2cc(ccc2c1)CN(C)C(=O)COC(=O)c1nccnc1c1nc2ccccc2s1	0.0	0.0	not tested	not tested	0.0	0.0
SN00777501	SC007383	Cc1csc(CNC(=O)c2nccnc2c2nc3ccccc3s2)n1	0.0	0.0	not tested	not tested	0.0	0.0
SN00778491	SC008241	CCNC(=O)CN1CCCN(CC1)C(=O)c1csc(n1)c1ccccc1	0.0	0.0	not tested	not tested	0.0	0.0
SN00778499	SC008241	Cc1ccc(nn1)N1CCCN(CC1)C(=O)c1csc(C)n1	0.0	0.0	not tested	not tested	0.0	0.0
SN00778544	SC008338	CCOC(=O)c1scc(n1)Cn1nc(oc1=O)c1ccc(F)cc1	0.0	0.0	not tested	not tested	0.0	0.0
SN00778556	SC008339	Fc1ccc(cc1)c1nn(Cc2onc(n2)c2ccccc2)c(=O)o1	0.0	0.0	not tested	not tested	0.0	0.0
SN00778558	SC008339	Fc1ccc(cc1)c1nn(Cc2onc(n2)c2cscc2)c(=O)o1	0.0	0.0	not tested	not tested	0.0	0.0
SN00778588	SC008566	O=C1CN(c2ccccc2)C(=O)N1Cc1noc(n1)c1ccco1	0.0	0.0	not tested	not tested	0.0	0.0
SN00778591	SC008566	CCCc1onc(n1)CN1C(=O)NC(C)(c2ccc3OCCOc3c2)C1=O	0.0	0.0	not tested	not tested	0.0	0.0
SN00779192	SC009466	CCCCCCn1c(=O)n(C)c2nc3N(CCn3c2c1=O)c1ccc(F)cc1	0.0	0.0	not tested	not tested	0.0	0.0
SN00780273	SC011033	O=C(NC(C)(C)c1noc(CN2CCC3(OCCO3)CC2)n1)OC(C)(C)C	0.0	0.0	not tested	not tested	0.0	0.0
SN00786468	CL8245A	CCOc1ccc(cc1)S(=O)(=O)N1CCN(CC1)c1nc(C)nc(c1)N1CCOCC1	0.0	0.0	not tested	not tested	0.0	0.0

Compound ID	Scaffold	Smiles	% Inhibition n=1		% Inhibition n=2		Average % Inhibition	
			24h	48h	24h	48h	24h	48h
SN00773062	SC014997	Cc1nsc(n1)N1CCCN(CC1)C(=O)NCC1CCC=CC1	0.0	0.0	not tested	not tested	0.0	0.0
SN00775081	SC001094	CC1Cc2ccccc2N1C(=O)C1CCN(CC1)S(=O)(=O)C	0.0	0.0	not tested	not tested	0.0	0.0
SN00775106	SC001098	O=C(N1CCc2ccccc12)c1cnn(c2cccc(c2)C(F)(F)F)c1C	0.0	0.0	not tested	not tested	0.0	0.0
SN00778480	SC008232	Clc1c[nH]c(c1)C(=O)N1CCCN(CC1)Cc1nccn1C	0.0	0.0	not tested	not tested	0.0	0.0
SN00778730	SC008707	CCCNC(=O)CN1CCN(CC1)Cc1nc(C)c2ccccc2n1	0.0	0.0	not tested	not tested	0.0	0.0
SN00778753	SC008719	Cc1cnn(CC2OCCN(C2)Cc2onc(c2)c2ccccc2)c1	0.0	0.0	not tested	not tested	0.0	0.0
SN00779434	SC009945	O=C(Nc1nc(C)c(s1)c1ccccn1)c1c[nH]c(c1)C(=O)N1CCCC1	0.0	0.0	not tested	not tested	0.0	0.0
SN00780524	SC011527	O=S(=O)(N1CCCC(C1)c1nnc[nH]1)c1cccc(c1)OC(F)(F)F	0.0	0.0	not tested	not tested	0.0	0.0
SN00781674	SC014751	CCN1C(=O)C2CN(CCN2C1=O)C(=O)C(C)Oc1ccc(cc1)c1ccccc1	0.0	0.0	not tested	not tested	0.0	0.0
SN00785335	CL2879	CCCCn1c(CN2CC(C)OC(C)C2)nc2n(C)c(=O)[nH]c(=O)c12	0.0	0.0	not tested	not tested	0.0	0.0
SN00799175	CL9812	O=C(NCCN1CCc2ccccc2C1)[CH]n1c(=O)c2nnc(C)n2c2ncccc12	0.0	0.0	not tested	not tested	0.0	0.0
SN00769964	SC001347	Cc1onc(COc2ccccc2C(=O)N2CCC(=CC2)c2c[nH]c3ccccc23)n1	0.0	0.0	not tested	not tested	0.0	0.0
SN00769975	SC001350	O=C(N1CCC(=CC1)c1ccccc1)c1ccc(s1)C(=O)C	0.0	0.0	not tested	not tested	0.0	0.0
SN00771075	SC007629	O=c1n(nnn1c1cccs1)Cc1nc2ccccc2[nH]1	0.0	0.0	not tested	not tested	0.0	0.0
SN00771076	SC007629	O=C(Cn1nnn(c2cccs2)c1=O)N1CCN(CC1)C1CC1	0.0	0.0	not tested	not tested	0.0	0.0
SN00771083	SC007650	Clc1c(cnn(c2ccc(cn2)C(F)(F)F)c1=O)n1cncc1	0.0	0.0	not tested	not tested	0.0	0.0
SN00771096	SC007650	Cc1cccc(c1)N1CCN(CCNc2cnn(c3ccc(cn3)C(F)(F)F)c(=O)c2Cl)CC1	0.0	0.0	not tested	not tested	0.0	0.0
SN00771111	SC007707	COc1cccc(c1)CC1CCCN1Cc1nccn1C	0.0	0.0	not tested	not tested	0.0	0.0
SN00771129	SC007707	CCc1cnc(o1)CN(C(=O)C)C1CCN(C1)Cc1nccn1CC	0.0	0.0	not tested	not tested	0.0	0.0
SN00772181	SC011798	O=C(Cc1csc(n1)NC(=O)c1ccco1)N1CCCC1c1onc(C)c1	0.0	0.0	not tested	not tested	0.0	0.0
SN00772200	SC011798	Fc1ccc(F)c(CCC(=O)N2CCCC2c2onc(C)c2)c1	0.0	0.0	not tested	not tested	0.0	0.0
SN00775088	SC001094	CC1Cc2ccccc2N1C(=O)C1CCN(CC1)S(=O)(=O)N	0.0	0.0	not tested	not tested	0.0	0.0
SN00775322	SC001261	CC1Oc2ccccc2N(C1)C(=O)C1CCN(CC1)C(=O)C(C)(C)C	0.0	0.0	not tested	not tested	0.0	0.0
SN00775324	SC001273	O=c1ccc(c[nH]1)C(=O)N1CCCC(C1)c1nc2ccccc2s1	0.0	0.0	not tested	not tested	0.0	0.0
SN00776471	SC003494	CCN(CC)CCCN1C(c2ccncc2)C(=C(O)C1=O)C(=O)c1cc2cccc(OC)c2o1	0.0	0.0	not tested	not tested	0.0	0.0
SN00776510	SC004527	O=C(NC1CCCC1)c1sccc1S(=O)(=O)N1CCOCC1	0.0	0.0	not tested	not tested	0.0	0.0
SN00776517	SC004527	COc1c(OC)cccc1CN(C)C(=O)c1sccc1S(=O)(=O)N1CCOCC1	0.0	0.0	not tested	not tested	0.0	0.0
SN00777647	SC007568	COC(=O)C1CCCCN1Cc1noc(c1)c1ccco1	0.0	0.0	not tested	not tested	0.0	0.0
SN00781686	SC014777	Cc1ccc2oc(cc2n1)C(=O)NC1(C)CCS(=O)(=O)C1	0.0	0.0	not tested	not tested	0.0	0.0
SN00781726	SC015007	CCc1csc(NC(=O)C2CCC(=O)N(C)C2c2cnn(C)c2)n1	0.0	0.0	not tested	not tested	0.0	0.0
SN00782799	SC003029	COC(=O)CC1C(=O)NCCN1Cc1onc(n1)C1CC1	0.0	0.0	not tested	not tested	0.0	0.0
SN00782802	SC003030	CNC1CCN(CC1)Cc1onc(C)c1	0.0	0.0	not tested	not tested	0.0	0.0
SN00782820	SC006689	CCOc1cc2CCN(Cc3nnc(o3)c3c(C)onc3c3ccccc3)Cc2cc1OCC	0.0	0.0	not tested	not tested	0.0	0.0
SN00782849	SC007582	C1CC(Cn2ncnc2)N(C1)Cc1nc2ccccn2c1	0.0	0.0	not tested	not tested	0.0	0.0
SN00784141	CL1050	COc1cc2c(cc1OC)C(=O)N(C)C(C2C(=O)O)c1ccccc1OC	0.0	0.0	not tested	not tested	0.0	0.0
SN00784163	CL4067	COc1cc2[C@H]([C@@H]3CCCCN3C(=O)c2cc1OC)C(=O)N(C)c1ccc(Cl)cc1	0.0	0.0	not tested	not tested	0.0	0.0
SN00793389	CL5116	COc1ccc(cc1)S(=O)(=O)N1CCOC21CCN(CC2)C(=O)c1ccc(Cl)cc1	0.0	0.0	not tested	not tested	0.0	0.0
SN00793398	CL5116	Fc1ccc(cc1)C(=O)N1CCC2(OCCN2S(=O)(=O)c2cccs2)CC1	0.0	0.0	not tested	not tested	0.0	0.0
SN00796866	CL9207A	O=C(NC1CCCCC1)N1CCc2n(C)nc(c3nnc(o3)c3ccccc3Cl)c2C1	0.0	0.0	not tested	not tested	0.0	0.0
SN00797961	CL9439A	O=C(NCc1ccc(F)cc1Cl)C1CCCN(C1)c1ncccc2c(C)nn(C)c12	0.0	0.0	not tested	not tested	0.0	0.0
SN00797970	CL9454	CCCn1c(SCC(=O)Nc2ccc3OCCOc3c2)nc2c(nsc2c1=O)c1ccccc1	0.0	0.0	not tested	not tested	0.0	0.0
SN00797974	CL9454	CCCn1c(SCC(=O)N)nc2c(nsc2c1=O)c1ccccc1	0.0	0.0	not tested	not tested	0.0	0.0
SN00799138	CL9766	CCCN1C(=O)C(CC(=O)NC2CCCCC2)Sc2ncccc12	0.0	0.0	not tested	not tested	0.0	0.0
SN00769877	SC001322	CCc1noc(C)c1C(=O)N1CCCC1c1c(C)nn(C)c1C	0.0	0.0	not tested	not tested	0.0	0.0
SN00769881	SC001322	COc1n(C)nc(C)c1C1CCCN1C(=O)c1c(C)noc1C(C)C	0.0	0.0	not tested	not tested	0.0	0.0
SN00769885	SC001339	O=C(Nc1scc(n1)c1cccnc1)Cn1cnc2ccccc2c1=O	0.0	0.0	not tested	not tested	0.0	0.0

Compound ID	Scaffold	Smiles	% Inhibition n=1		% Inhibition n=2		Average % Inhibition	
			24h	48h	24h	48h	24h	48h
SN00769940	SC001340	O=S(=O)(c1ccccc1NCCc1scc(n1)c1ccncc1)C(F)(F)F	0.0	0.0	not tested	not tested	0.0	0.0
SN00771017	SC007569	Cc1nn(C)c(C)c1C1CCCN1Cc1nnnn1c1ccccc1	0.0	0.0	not tested	not tested	0.0	0.0
SN00772209	SC011876	CCC(C)C(=O)N1CCCN(CC1)Cc1nc2ccc(Cl)cn2c1	0.0	0.0	not tested	not tested	0.0	0.0
SN00772216	SC011876	CCCCC(=O)N1CCCN(CC1)Cc1nc2ccc(Cl)cn2c1	0.0	0.0	not tested	not tested	0.0	0.0
SN00773298	SC015439	O=C(NCCCc1ccccc1)c1nc2NS(=O)(=O)c3ccccc3n2n1	0.0	0.0	not tested	not tested	0.0	0.0
SN00777605	SC007512	Cc1ccc(N2CCCC2)c(c1)C(=O)N1CC2CCC1C2	0.0	0.0	not tested	not tested	0.0	0.0
SN00780449	SC011311	Fc1ccc(F)c(c1)C(=O)Nc1cnccc1n1cncc1	0.0	0.0	not tested	not tested	0.0	0.0
SN00780458	SC011317	CN1CCN(CC1)S(=O)(=O)N1CCN(CC1)C(=O)c1ccc(C)o1	0.0	0.0	not tested	not tested	0.0	0.0
SN00780467	SC011317	COc1cc(Cl)c(C)cc1NC(=O)N1CCN(CC1)S(=O)(=O)N1CCN(C)CC1	0.0	0.0	not tested	not tested	0.0	0.0
SN00784155	CL1050	COc1cc2c(cc1OC)C(=O)N(C)C(c1cccc(F)c1)C2C(=O)O	0.0	0.0	not tested	not tested	0.0	0.0
SN00788780	CL3248	O=C(NC1CCCCC1)Cn1c2cc(C)c(C)cc2nc(c1=O)C(F)(F)F	0.0	0.0	not tested	not tested	0.0	0.0
SN00788781	CL3248	O=C(Nc1nccs1)Cn1c2cc(C)c(C)cc2nc(c1=O)C(F)(F)F	0.0	0.0	not tested	not tested	0.0	0.0
SN00788805	CL3096	COc1cccc(c1)NC(=O)C1CCCN(C1)c1nc2ccccc2nc1C(F)(F)F	0.0	0.0	not tested	not tested	0.0	0.0
SN00788811	CL3096	O=C(C(C)C)N1CCN(CC1)c1nc2ccccc2nc1C(F)(F)F	0.0	0.0	not tested	not tested	0.0	0.0
SN00789908	CL4240	Fc1cccc(c1)Cn1c(C)c(C2=CCN(CC2)S(=O)(=O)c2c(C)noc2C)c2ccccc12	0.0	0.0	not tested	not tested	0.0	0.0
SN00789938	CL7893	COc1ccc(Cl)cc1NS(=O)(=O)c1ccc(s1)c1nnc(o1)C1CCC1	0.0	0.0	not tested	not tested	0.0	0.0
SN00791054	CL8456	Cc1cccc(c1)NS(=O)(=O)c1ccc2oc(c3onc(n3)c3ccccn3)c(C)c2c1	0.0	0.0	not tested	not tested	0.0	0.0
SN00791078	CL8457	COc1ccc(cc1)c1csc(n1)c1nnn(c1N)c1cc(Cl)c(OC)cc1OC	0.0	0.0	not tested	not tested	0.0	0.0
SN00793374	CL4056	Cc1ccc(cc1)N1CC(NC(=O)Nc2nccs2)CC1=O	0.0	0.0	not tested	not tested	0.0	0.0
SN00793408	CL5923	O=C(NCc1n[nH]c(=O)c2ccccc12)c1ccccc1F	0.0	0.0	not tested	not tested	0.0	0.0
SN00794526	CL1655	O=C(N1CCCC1)c1cnn2c1nc(cc2C(F)(F)F)c1cccs1	0.0	0.0	not tested	not tested	0.0	0.0
SN00795671	CL8811	CCN1CCN(CC1)C(=O)c1ccc(cc1)c1onc(n1)c1ccccc1	0.0	0.0	not tested	not tested	0.0	0.0
SN00795708	CL8811A	O=C(NCCN1CCN(CC1)c1ccccc1F)c1cccc(c1)c1onc(n1)c1ccccc1	0.0	0.0	not tested	not tested	0.0	0.0
SN00796841	CL9207A	COc1cccc(c1)NC(=O)N1CCc2n(C)nc(c3nnc(o3)c3ccc(F)cc3)c2C1	0.0	0.0	not tested	not tested	0.0	0.0
SN00774234	SC000841	O=C(Nc1ccc2OCOc2c1)CN1C(=O)N(C(C)C2CC3CCC2C3)C(=O)C1=O	0.0	0.0	not tested	not tested	0.0	0.0
SN00774249	SC000841	O=C1NC(=O)C(=O)N1C(C)C1CC2CCC1C2	0.0	0.0	not tested	not tested	0.0	0.0
SN00774268	SC000844	Cc1noc(n1)Cn1cnc2sc(c3ccccc3)c(C)c2c1=O	0.0	0.0	not tested	not tested	0.0	0.0
SN00781741	SC015062	O=C(CN1CCOC(C1)Cn1cncn1)N(C)Cc1ccccc1	0.0	0.0	not tested	not tested	0.0	0.0
SN00781752	SC015062	O=C(NC1CCCCCC1)CN1CCOC(C1)Cn1nc(C)nc1C	0.0	0.0	not tested	not tested	0.0	0.0
SN00781773	SC015096	C1CCC(CC1)c1scc(CN2CCc3cncnc3C2)n1	0.0	0.0	not tested	not tested	0.0	0.0
SN00782886	SC007868	CCCN1CCCN(CC1)C(=O)c1c(C)noc1C(C)C	0.0	0.0	not tested	not tested	0.0	0.0
SN00784223	CL1159	O=c1[nH]c(nnc1Cc1ccccc1)NC1CCCCC1	0.0	0.0	not tested	not tested	0.0	0.0
SN00784238	CL1159	Clc1ccc(cc1)Cc1nnc(Nc2ccc(Cl)cc2)[nH]c1=O	0.0	0.0	not tested	not tested	0.0	0.0
SN00785405	CL4469D	Fc1ccc(cc1)C(=O)N1CCN(CC1)c1nc2cc(Br)ccc2n2cccc12	0.0	0.0	not tested	not tested	0.0	0.0
SN00785422	CL8280A	Cc1cc(C)c(c(C)c1)S(=O)(=O)N1CCN(C)c2ccccc12	0.0	0.0	not tested	not tested	0.0	0.0
SN00786516	CL7986	CCOc1ccc(cc1)N1C(=O)CN(Cc2ccc3OCOc3c2)[C@H]2CS(=O)(=O)C[C@@H]12	0.0	0.0	not tested	not tested	0.0	0.0
SN00787612	CL5111B	CCOc1ccc(cc1)NC(=O)CCn1c(=O)c2cccn2c2cccnc12	0.0	0.0	not tested	not tested	0.0	0.0
SN00793447	CL7354	Cc1ccc(cc1)n1nnc2c(ncnc12)N1CCN(CC1)S(=O)(=O)c1ccc(Cl)cc1	0.0	0.0	not tested	not tested	0.0	0.0
SN00793462	CL7354	COc1cccc(c1)n1nnc2c(ncnc12)N1CCN(CC1)S(=O)(=O)c1ccc(CC)s1	0.0	0.0	not tested	not tested	0.0	0.0
SN00793477	CL6605A	CCNc1cc(C)nc(n1)N1CCN(CC1)C(=O)c1cc(Cl)ccc1OC	0.0	0.0	not tested	not tested	0.0	0.0
SN00794612	CL2785	CCCS(=O)(=O)N1CCCC(C1)C(=O)NCCCN1CCC(CC1)Cc1ccccc1	0.0	0.0	not tested	not tested	0.0	0.0
SN00795657	CL8804	COc1ccc(cc1)c1onc(n1)c1ccc(cc1)c1csc(n1)C1CC1	0.0	0.0	not tested	not tested	0.0	0.0
SN00795732	CL9032	O=C(Cn1cccc1c1nnc(o1)C1CC1)N1CCc2ccccc2C1	0.0	0.0	not tested	not tested	0.0	0.0
SN00795733	CL9032	COc1ccc(OC)c(NC(=O)Cn2cccc2c2nnc(o2)C2CC2)c1	0.0	0.0	not tested	not tested	0.0	0.0
SN00796807	CL9207	Fc1ccc(cc1)c1nnc(o1)c1nn(C)c2CCN(Cc12)C(=O)c1ccccc1Br	0.0	0.0	not tested	not tested	0.0	0.0
SN00796922	CL9317	Cc1ccc(cc1)CNC(=O)c1ccc2nn(CC(=O)NCCC3=CCCCC3)c(=O)n2c1	0.0	0.0	not tested	not tested	0.0	0.0

Compound ID	Scaffold	Smiles	% Inhibition n=1		% Inhibition n=2		Average % Inhibition	
			24h	48h	24h	48h	24h	48h
SN00796924	CL9317	Cc1ccc(cc1)CNC(=O)c1ccc2nn(CC(=O)N(C)c3ccccc3)c(=O)n2c1	0.0	0.0	not tested	not tested	0.0	0.0
SN00798046	CL9457	COc1ccc(cc1)n1ccc(=O)c(n1)c1onc(n1)c1ccc(OC)c(OC)c1	0.0	0.0	not tested	not tested	0.0	0.0
SN00798077	CL7707A	Cc1ccc(cc1)c1ncnc(c1)N1CCN(CC1)C(=O)c1c(F)cccc1Cl	0.0	0.0	not tested	not tested	0.0	0.0
SN00799249	CL9898A	Fc1ccc(cc1)NC(=O)N1CCC(CCOc2ccccc2)CC1	0.0	0.0	not tested	not tested	0.0	0.0
SN00774280	SC000844	O=c1n(cnc2sccc12)Cc1onc(Cc2ccccc2)n1	0.0	0.0	not tested	not tested	0.0	0.0
SN00775344	SC001273	O=C(N1CCCC(C1)c1noc(n1)C(C)C)c1ccc(=O)n(C)c1	0.0	0.0	not tested	not tested	0.0	0.0
SN00775359	SC001382	Cc1ccc(cc1)Cn1nc(C)c(C(=O)N2CCNC(=O)C2)c1C	0.0	0.0	not tested	not tested	0.0	0.0
SN00775371	SC001382	O=C1NCCN(C1)C(=O)c1cnn(c1C1CC1)c1ccccc1C	0.0	0.0	not tested	not tested	0.0	0.0
SN00786569	CL8274	COc1cc(ccc1NC(=O)COc1ccccc1)N1CCn2c(C1)nc1cc(Cl)ccc21	0.0	0.0	not tested	not tested	0.0	0.0
SN00786575	CL9232	O=C(NCCC1=CCCCC1)Cc1sc(C)nc1c1cccs1	0.0	0.0	not tested	not tested	0.0	0.0
SN00787736	CL5093	COc1ccc(cc1)NC(=O)Cc1onc(n1)c1ccc(OC)cc1	0.0	0.0	not tested	not tested	0.0	0.0
SN00787776	CL4881	CCOc1ccc(cc1)c1noc(n1)c1nnn(c2ccc(Br)cc2)c1N	0.0	0.0	not tested	not tested	0.0	0.0
SN00788905	CL1215	Cc1ccc(cc1)NC(=O)C1CCCCN1S(=O)(=O)c1ccc(Br)cc1	0.0	0.0	not tested	not tested	0.0	0.0
SN00788909	CL7223A	O=C(NCc1ccccc1)CN1C(=O)COc2ccc(cc12)C(=O)N1CCCCC1	0.0	0.0	not tested	not tested	0.0	0.0
SN00790004	CL8109	Cc1ccnc(c1)NC(=O)C1CCN(CC1)c1c(C)n(c2ccccc2)c(=O)n(CC(C)C)c1=O	0.0	0.0	not tested	not tested	0.0	0.0
SN00790011	CL8110	CCC1Oc2ccc(Cl)cc2N(CC(=O)N2CCN(CC2)c2c(C)n(C)c(=O)n(C)c2=O)C1=O	0.0	0.0	not tested	not tested	0.0	0.0
SN00791122	CL6193	CCOC(=O)C1CCN(CC1)c1nn2nnnc2c2ccccc12	0.0	0.0	not tested	not tested	0.0	0.0
SN00791184	CL4521	COCCN(CCc1ccncc1)S(=O)(=O)c1csc(c1)C(=O)N	0.0	0.0	not tested	not tested	0.0	0.0
SN00792287	CL7010B	O=C(Nc1ccc(C)c(Cl)c1)c1ncoc1c1ccco1	0.0	0.0	not tested	not tested	0.0	0.0
SN00792294	CL7010B	CN(CCNC(=O)c1ncoc1c1ccco1)Cc1ccccc1	0.0	0.0	not tested	not tested	0.0	0.0
SN00792320	CL7010A	Cc1ccc(s1)c1ocnc1C(=O)NCc1ccccc1C	0.0	0.0	not tested	not tested	0.0	0.0
SN00792324	CL6845	Cc1ccc(cc1)CN1CCCn2nc(cc2C1=O)C(=O)N1CCC(CC1)N1CCCCC1	0.0	0.0	not tested	not tested	0.0	0.0
SN00795773	CL8976B	O=C(Nc1ccccc1)N1CCC[C@@H]1C(=O)NCc1csc(n1)c1cccs1	0.0	0.0	not tested	not tested	0.0	0.0
SN00795782	CL8976B	O=C(NCCc1ccccc1)N1CCC[C@@H]1C(=O)NCc1csc(n1)c1cccs1	0.0	0.0	not tested	not tested	0.0	0.0
SN00796896	CL9207B	CC(=O)Nc1ccc(C)cc1S(=O)(=O)N1CCc2n(C)nc(c3nnc(o3)c3ccccc3)c2C1	0.0	0.0	not tested	not tested	0.0	0.0
SN00798113	CL9469	Clc1ccc(cc1)c1noc(n1)c1cccn2c1nn(CC(=O)N1CCCC(C)C1)c2=O	0.0	0.0	not tested	not tested	0.0	0.0
SN00798131	CL6358	CSc1cccc(c1)NC(=O)Cn1cc(ccc1=O)c1onc(n1)C1CC1	0.0	0.0	not tested	not tested	0.0	0.0
SN00799182	CL9831	CN(C)CCNC(=O)c1c(C)sc2c1nnn(C)c2=O	0.0	0.0	not tested	not tested	0.0	0.0
SN00799190	CL9882	O=C(Nc1cccc(c1)c1cncc(c1)C(=O)NCc1ccncc1)C1CCC1	0.0	0.0	not tested	not tested	0.0	0.0
SN00799269	CL4844	CCn1ncc(NC(=O)c2ccc(F)cc2)c(N2CCOCC2)c1=O	0.0	0.0	not tested	not tested	0.0	0.0
SN00788056	CL5555	O=C(Cn1cnc2n3CCCCc3nc2c1=O)N1CCN(CC1)Cc1ccc2OCOc2c1	0.0	0.0	not tested	not tested	0.0	0.0
SN00789181	CL5777	COc1ccc(cc1OC)NC(=O)N1Cc2cccn2c2ccccc12	0.0	0.0	not tested	not tested	0.0	0.0
SN00789189	CL5777	CCOc1ccc(cc1)NC(=O)N1Cc2cccn2c2ccccc12	0.0	0.0	not tested	not tested	0.0	0.0
SN00790108	CL8279	O=C(CN1CCN(CC1)c1ccc(NC(=O)C(C)(C)C)c(c1)C(=O)O)N1CCCC1	0.0	0.0	not tested	not tested	0.0	0.0
SN00790329	CL7708B	CCCNC(=O)C1CCCN(C1)c1ccnc(n1)c1ccccc1	0.0	0.0	not tested	not tested	0.0	0.0
SN00790334	CL7801	O=C(NCc1ccccc1Cl)Cn1cc(c2nnco2)c2ccccc12	0.0	0.0	not tested	not tested	0.0	0.0
SN00790352	CL7801	CSc1ccc(cc1)CNC(=O)Cn1cc(c2nnc(CC)o2)c2ccccc12	0.0	0.0	not tested	not tested	0.0	0.0
SN00791217	CL6082	CCc1ccc(cc1)n1c(=O)c2cccnc2n1CC(=O)NCCC1=CCCCC1	0.0	0.0	not tested	not tested	0.0	0.0
SN00791222	CL6067	Clc1cccc(c1)n1nnc(c2onc(n2)c2ccccc2)c1C	0.0	0.0	not tested	not tested	0.0	0.0
SN00791231	CL6067	Cc1ccc(cc1)c1noc(n1)c1nnn(c2cccc(F)c2)c1C	0.0	0.0	not tested	not tested	0.0	0.0
SN00792370	CL7193	O=C(CCCN1C(=O)CCn2nc(cc12)c1cccn1C)NCc1cccc(C)c1	0.0	0.0	not tested	not tested	0.0	0.0
SN00792374	CL7193	O=C(CCCN1C(=O)CCn2nc(cc12)c1cccn1C)NCc1ccc(C)o1	0.0	0.0	not tested	not tested	0.0	0.0
SN00793550	CL6605C	Clc1ccc(cc1)S(=O)(=O)N1CCN(CC1)c1nc(C)cc(n1)N1CCCC1	0.0	0.0	not tested	not tested	0.0	0.0
SN00793781	CL5102	CCOc1ccc(cc1)NC(=O)Cc1n(CC)cc(C)c1C(=O)O	0.0	0.0	not tested	not tested	0.0	0.0
SN00794669	CL3088	O=C1CN=C(c2ccccc2)c2ccccc2N1Cc1nc(oc1C)c1ccccc1	0.0	0.0	not tested	not tested	0.0	0.0
SN00794890	CL9106	Cc1ccc(cc1)c1noc(n1)c1cccn(CC(=O)NCCc2ccccc2)c1=O	0.0	0.0	not tested	not tested	0.0	0.0

Compound ID	Scaffold	Smiles	% Inhibition n=1		% Inhibition n=2		Average % Inhibition	
			24h	48h	24h	48h	24h	48h
SN00794975	CL8661	CC(=O)Nc1[nH]nc(n1)S(=O)(=O)C	0.0	0.0	not tested	not tested	0.0	0.0
SN00794976	CL8661	OC(=O)CCS(=O)(=O)c1n[nH]c(NC(=O)C)n1	0.0	0.0	not tested	not tested	0.0	0.0
SN00795858	CL8682	COc1ccc(cc1)CNC(=O)C1(C)CCCN(C1)C(=O)Cc1cccs1	0.0	0.0	not tested	not tested	0.0	0.0
SN00796955	CL9360	CCC(C)NC(=O)Cn1c2nc(sc2c(=O)n(C(C)C)c1=O)N1CCC(C)CC1	0.0	0.0	not tested	not tested	0.0	0.0
SN00796961	CL9210A	CC(=O)N(Cc1nn(C)c2CCN(Cc12)C(=O)Nc1ccc(C)c(Cl)c1)c1ccccc1	0.0	0.0	not tested	not tested	0.0	0.0
SN00796983	CL9210A	Cc1cccc(c1)NC(=O)N1CCc2n(C)nc(CN(c3ccccc3)C(=O)c3ccccc3F)c2C1	0.0	0.0	not tested	not tested	0.0	0.0
SN00796987	CL9210B	CC(=O)N(Cc1nn(C)c2CCN(Cc12)S(=O)(=O)c1ccc2OCCOc2c1)c1ccccc1	0.0	0.0	not tested	not tested	0.0	0.0
SN00796989	CL9210B	CC(=O)N(Cc1nn(C)c2CCN(Cc12)S(=O)(=O)c1ccccc1)c1ccccc1	0.0	0.0	not tested	not tested	0.0	0.0
SN00782030	SC015449	Fc1ccc(cc1)C1(CCOCC1)c1nnc(o1)c1ccccc1	0.0	0.0	not tested	not tested	0.0	0.0
SN00782049	SC015449	Brc1ccc(cc1)C1(CCOCC1)c1nnc(Cc2ccccc2F)o1	0.0	0.0	not tested	not tested	0.0	0.0
SN00782054	SC015495	O=C(CCc1c[nH]c2ccccc12)N1CCCCC1c1ccco1	0.0	0.0	not tested	not tested	0.0	0.0
SN00782081	SC015501	Fc1ccc(cc1)c1nc(C)c(s1)c1onc(n1)C1COCC1	0.0	0.0	not tested	not tested	0.0	0.0
SN00783386	CL1650	COc1ccc(cc1)c1nc2c(Cl)c(nn2c(c1)C(F)(F)F)C(=O)N1CCOCC1	0.0	0.0	not tested	not tested	0.0	0.0
SN00783408	CL2614	CCCc1[nH]nc2OC(=C(CN)C(c3ccccc3)c12)N	0.0	0.0	not tested	not tested	0.0	0.0
SN00783413	CL0706	Clc1ccc(nc1)NC(=O)Cn1cnc2c1c(=O)n(C)c(=O)n2C	0.0	0.0	not tested	not tested	0.0	0.0
SN00783437	CL3225	O=C(Nc1c(nc2sccn12)c1ccccc1)c1ccc(cc1)C(C)(C)C	0.0	0.0	not tested	not tested	0.0	0.0
SN00783467	CL0706	COc1ccccc1NC(=O)Cn1cnc2c1c(=O)n(C)c(=O)n2C	0.0	0.0	not tested	not tested	0.0	0.0
SN00783517	CL7566	CC(=O)Nc1nonc1c1nc2ccccc2n1Cc1ccccc1C	0.0	0.0	not tested	not tested	0.0	0.0
SN00789157	CL5683	COc1ccc(cc1)n1ccn(CC(=O)Nc2cccc(F)c2)c1=O	0.0	0.0	not tested	not tested	0.0	0.0
SN00791408	CL6014A	Clc1cccc(c1)N1CCN(CC1)S(=O)(=O)c1c(C)[nH]c(C)c1C(=O)N1CCCC1	0.0	0.0	not tested	not tested	0.0	0.0
SN00792619	CL7041	Fc1ccc(cc1)CNC(=O)c1cc2cccnc2n(Cc2ccccc2)c1=O	0.0	0.0	not tested	not tested	0.0	0.0
SN00793734	CL7419	Cc1onc(n1)c1ccc(C)c(c1)S(=O)(=O)N1CCN(CC1)c1ccccc1	0.0	0.0	not tested	not tested	0.0	0.0
SN00793743	CL7419	CCc1onc(n1)c1ccc(C)c(c1)S(=O)(=O)Nc1cc(Cl)ccc1OC	0.0	0.0	not tested	not tested	0.0	0.0
SN00794869	CL8689	COc1cccc(c1)n1nnc(c2nc(C)c(s2)C(=O)NCCC2=CCCCC2)c1C	0.0	0.0	not tested	not tested	0.0	0.0
SN00794871	CL8126	CN(Cc1ccccc1)c1nc2ccccc2nc1S(=O)(=O)C(C)C	0.0	0.0	not tested	not tested	0.0	0.0
SN00794932	CL8795	CCn1c(=O)n(Cc2onc(n2)c2ccc(cc2)OC(C)C)c2ccccc2c1=O	0.0	0.0	not tested	not tested	0.0	0.0
SN00796031	CL8162A	Clc1cccc(c1)Cn1c(cc2ccsc12)C(=O)N1CCCC(C1)C(=O)NCc1ccccn1	0.0	0.0	not tested	not tested	0.0	0.0
SN00796033	CL8162A	O=C(NCc1ccco1)C1CCCN(C1)C(=O)c1cc2ccsc2n1Cc1cccc(Cl)c1	0.0	0.0	not tested	not tested	0.0	0.0
SN00796076	CL6601	COc1ccccc1CNC(=O)C1(C)Cc2ccccc2C(=O)N1CC1CC1	0.0	0.0	not tested	not tested	0.0	0.0
SN00783165	SC013362	CC(O)CN1CCN(CC1)C(=O)C1COCC1	0.0	0.0	not tested	not tested	0.0	0.0
SN00783197	SC013929	FC(F)Oc1ccc(Br)cc1CN1CCCN2CCCC2C1	0.0	0.0	not tested	not tested	0.0	0.0
SN00784553	CL2023	CSc1ccc(cc1)c1nc2[nH]cnn2c1Nc1ccc(C)cc1	0.0	0.0	not tested	not tested	0.0	0.0
SN00785672	CL4403A	Clc1ccc(CCNC(=O)Cc2c(C)nc3cc(nn3c2C)c2ccccc2)cc1	0.0	0.0	not tested	not tested	0.0	0.0
SN00786801	CL1131A	CCOc1ccc(cc1)NC(=O)CN1C(=CC(c2ccccc2)n2nnnc12)c1ccc(C)cc1	0.0	0.0	not tested	not tested	0.0	0.0
SN00786850	CL9431	Cc1ccc2oc(nc2n1)N1CCCN(CC1)C(=O)CCN1C(=O)c2ccccc2C1=O	0.0	0.0	not tested	not tested	0.0	0.0
SN00790368	CL7771	CCOCCCNC(=O)N1CCN(Cc2ccccc2)c2ncccc12	0.0	0.0	not tested	not tested	0.0	0.0
SN00790373	CL7771	CCN1CCN(C(=O)Nc2ccc(Cl)cc2)c2cccnc12	0.0	0.0	not tested	not tested	0.0	0.0
SN00791461	CL6241	Cc1n[nH]c(c1)c1ccc(s1)S(=O)(=O)N1CCN(CC1)c1cccc(C)c1C	0.0	0.0	not tested	not tested	0.0	0.0
SN00791469	CL6241	Cc1n[nH]c(c1)c1ccc(s1)S(=O)(=O)NCC1CCN(CC1)Cc1ccccc1F	0.0	0.0	not tested	not tested	0.0	0.0
SN00791495	CL6497	O=C(NCCc1ccc(cc1)S(=O)(=O)N)c1ccc(s1)N1CCOc2ccccc12	0.0	0.0	not tested	not tested	0.0	0.0
SN00791541	CL6662	CC(C)NC(=O)N1Cc2c3CCCCc3sc2n2cccc2C1C	0.0	0.0	not tested	not tested	0.0	0.0
SN00792636	CL7041	Fc1ccccc1Cn1c(=O)c(cc2cccnc12)C(=O)NCc1ccccc1Cl	0.0	0.0	not tested	not tested	0.0	0.0
SN00792690	CL6292	O=C(NC1CC1)C1CCCN(C1)C(=O)c1n[nH]c(c1)c1cccnc1	0.0	0.0	not tested	not tested	0.0	0.0
SN00793680	CL7416	CCn1nnc2cc(ccc12)c1noc(n1)c1ccc(Cl)cc1	0.0	0.0	not tested	not tested	0.0	0.0
SN00793694	CL7416	O=C(NCc1onc(n1)c1ccc2c(nnn2C2CCCC2)c1)Cc1ccccc1	0.0	0.0	not tested	not tested	0.0	0.0
SN00796063	CL9297	CCc1ccc(cc1)S(=O)(=O)N1CCC2(NCCN=C2NC2CCCCCC2)CC1	0.0	0.0	not tested	not tested	0.0	0.0

Compound ID	Scaffold	Smiles	% Inhibition n=1		% Inhibition n=2		Average % Inhibition	
			24h	48h	24h	48h	24h	48h
SN00796075	CL6601	COc1ccccc1CNC(=O)C1(C)Cc2ccccc2C(=O)N1Cc1ccc(cc1)C(F)(F)F	0.0	0.0	not tested	not tested	0.0	0.0
SN00797166	CL9330	CC(=O)Nc1ccc(cc1)c1noc(n1)C1CCCN(C1)C(=O)c1ccc(Br)o1	0.0	0.0	not tested	not tested	0.0	0.0
SN00797171	CL9330	CC(=O)Nc1ccc(cc1)c1noc(n1)C1CCCN(C1)C(=O)/C=C/c1ccc(C)cc1	0.0	0.0	not tested	not tested	0.0	0.0
SN00797212	CL9330A	CCC(=O)Nc1cccc(c1)c1noc(n1)C1CCCN(C1)C(=O)COc1cccc(C)c1	0.0	0.0	not tested	not tested	0.0	0.0
SN00798370	CL9600	COc1ccccc1N1CCN(CC1)C(=O)Cn1c(=O)n(C(C)C)c2ccc(cc12)c1noc(C)n1	0.0	0.0	not tested	not tested	0.0	0.0
SN00783097	SC011033	CCc1noc(CN2CCC3(OCCO3)CC2)n1	0.0	0.0	not tested	not tested	0.0	0.0
SN00783116	SC011330	CCCNC(=O)CN1CCCN(CC1)C(=O)c1cccc(C)n1	0.0	0.0	not tested	not tested	0.0	0.0
SN00783129	SC011809	Cc1onc(CN2CCCN(CC2)c2cccnn2)n1	0.0	0.0	not tested	not tested	0.0	0.0
SN00784443	CL0892	COc1cc(NC(=O)Cc2ccc(cc2)n2c(=O)[nH]c3ccsc3c2=O)cc(OC)c1	0.0	0.0	not tested	not tested	0.0	0.0
SN00784446	CL1789	CCC1Oc2ccc(C)cc2N(CC(=O)NCc2ccccc2)C1=O	0.0	0.0	not tested	not tested	0.0	0.0
SN00784448	CL1790	O=c1[nH]c2ccc(cc2c(c1)C(=O)NCc1ccccc1Cl)S(=O)(=O)N1CCCCCC1	0.0	0.0	not tested	not tested	0.0	0.0
SN00784469	CL2032	O=C(NCc1ccccc1)c1noc(c1)c1ccco1	0.0	0.0	not tested	not tested	0.0	0.0
SN00785598	CL4056	CCOC(=O)c1ccc(cc1)NC(=O)NC1CC(=O)N(C1)C1CCCC1	0.0	0.0	not tested	not tested	0.0	0.0
SN00785622	CL4062	CCOC(=O)c1ccc(cc1)NC(=O)Cn1cc2CCCCCc2n1	0.0	0.0	not tested	not tested	0.0	0.0
SN00786739	CL7520	COc1cc(ccc1OC)c1ccnc2cc(nn12)C(=O)Nc1ccc(F)cc1F	0.0	0.0	not tested	not tested	0.0	0.0
SN00786745	CL7520	CCc1ccc(cc1)NC(=O)c1cc2nccc(c3ccc(OC)c(OC)c3)n2n1	0.0	0.0	not tested	not tested	0.0	0.0
SN00786783	CL7519	O=C(NC1CCCCC1)c1nn(C)c(c1)C(F)(F)F	0.0	0.0	not tested	not tested	0.0	0.0
SN00787951	CL5298A	O=C(NCc1ccc(C)o1)CN1C(=O)CCCc2ccccc12	0.0	0.0	not tested	not tested	0.0	0.0
SN00787953	CL5298A	O=C(NCCc1ccco1)CN1C(=O)CCCc2ccccc12	0.0	0.0	not tested	not tested	0.0	0.0
SN00790252	CL7460	CCCC(=O)Nc1ccc2nc(cc(C(=O)O)c2c1)N1CCCC1	0.0	0.0	not tested	not tested	0.0	0.0
SN00791335	CL6481	O=C(NCCC1=CCCCC1)c1cnc2sc(nn2c1=O)N1CCCCC1	0.0	0.0	not tested	not tested	0.0	0.0
SN00791353	CL6481	CC(C)c1ccc(cc1)NC(=O)c1cnc2sc(nn2c1=O)N1CCCC1	0.0	0.0	not tested	not tested	0.0	0.0
SN00791367	CL6564	Cc1cccc(c1)CN1C(=O)c2oc3ccccc3c2OCC1(C)C(=O)NC1CCCC1	0.0	0.0	not tested	not tested	0.0	0.0
SN00793655	CL7067C	Cc1ccc(cc1)CN1CCN(C2CCN(CC2)C(=O)Nc2ccc(Cl)cc2)C(=O)C1=O	0.0	0.0	not tested	not tested	0.0	0.0
SN00794802	CL8624	CSc1cccc(c1)NC(=O)N1CCN(CC1)c1nc2ccccc2n1CC	0.0	0.0	not tested	not tested	0.0	0.0
SN00795940	CL8682B	Cc1onc(C)c1S(=O)(=O)N1CCCC(C)(C1)C(=O)NCc1ccccc1	0.0	0.0	not tested	not tested	0.0	0.0
SN00795957	CL8922	Brc1ccc(o1)C(=O)Nc1cc(F)c2nc(ccc2c1)N1CCOCC1	0.0	0.0	not tested	not tested	0.0	0.0
SN00797098	CL9311	Clc1cccc(c1)NC(=O)c1cnn2C(/C=C/c3ccccc3)C=C(Nc12)C(=O)O	0.0	0.0	not tested	not tested	0.0	0.0
SN00797128	CL8861A	COc1cc(OC)cc(c1)NC(=O)C1CCCN(C1)c1nccnc1Oc1ccccc1C	0.0	0.0	not tested	not tested	0.0	0.0
SN00797150	CL9329A	CC(=O)Nc1ccc(cc1)c1noc(n1)C1CCN(CC1)C(=O)C1(CC1)c1ccccc1	0.0	0.0	not tested	not tested	0.0	0.0
SN00797161	CL9329A	CC(=O)Nc1ccc(cc1)c1noc(n1)C1CCN(CC1)C(=O)COc1ccc(F)cc1	0.0	0.0	not tested	not tested	0.0	0.0
SN00798246	CL9556	COc1cccc(CNC(=O)[CH]N2CCN(CCC2=O)C(C)C)c1	0.0	0.0	not tested	not tested	0.0	0.0
SN00798249	CL9509	Clc1ccc(cc1)NC(=O)c1cccn1Cc1noc(C)n1	0.0	0.0	not tested	not tested	0.0	0.0
SN00798268	CL9509	Fc1ccc(cc1)NC(=O)c1cccn1Cc1noc(n1)c1ccccc1	0.0	0.0	not tested	not tested	0.0	0.0
SN00798277	CL9559	O=c1ccc(n[nH]1)c1sc(C)c(c1)S(=O)(=O)NCc1cccnc1	0.0	0.0	not tested	not tested	0.0	0.0
SN00798293	CL9559	CCNS(=O)(=O)c1cc(sc1C)c1ccc(=O)n(CC)n1	0.0	0.0	not tested	not tested	0.0	0.0
SN00781905	SC015174	O=C(c1csc(n1)C1CC1)N1CCn2c(nnc2C)C1	0.0	0.0	not tested	not tested	0.0	0.0
SN00781916	SC015174	O=C(c1csc(n1)C1CC1)N1CCn2c(nnc2C(C)C)C1	0.0	0.0	not tested	not tested	0.0	0.0
SN00781919	SC015176	O=C(N1CCn2c(nnc2C)C1)c1ccc(nc1C)c1cccs1	0.0	0.0	not tested	not tested	0.0	0.0
SN00781945	SC015176	O=C(c1ccc(nc1)NC1CC1)N1CCn2c(nnc2C(C)C)C1	0.0	0.0	not tested	not tested	0.0	0.0
SN00783007	SC010190	CN1CCCC1C1CCCN1C(=O)c1nnn(c2ccccc2)c1C	0.0	0.0	not tested	not tested	0.0	0.0
SN00783011	SC010225	CCCCc1onc(CN2CCCC2Cn2nc(C)nc2C)n1	0.0	0.0	not tested	not tested	0.0	0.0
SN00783025	SC010358	CCOC1CC(NC(=O)Nc2nccs2)C21CCC2	0.0	0.0	not tested	not tested	0.0	0.0
SN00783058	SC010804	CCOC(=O)CCCS(=O)(=O)N1CCCC1CN1CCCC1	0.0	0.0	not tested	not tested	0.0	0.0
SN00784295	CL1728	CSc1ccc(cc1)c1nc2cnccn2c1NC1CCCCC1	0.0	0.0	not tested	not tested	0.0	0.0
SN00784401	CL7855	CCOc1ccc(cc1)NS(=O)(=O)c1n[nH]c(c1)C(=O)NCc1ccccc1	0.0	0.0	not tested	not tested	0.0	0.0

Compound ID	Scaffold	Smiles	% Inhibition n=1		% Inhibition n=2		Average % Inhibition	
			24h	48h	24h	48h	24h	48h
SN00785527	CL4805A	CCOC(=O)c1nn2CC(C)(C(=O)NCCC(C)C)N(c3ccc(Cl)cc3)C(=O)c2c1	0.0	0.0	not tested	not tested	0.0	0.0
SN00785551	CL6301A	COc1ccc(cc1)C1N(Cc2ccccc2n2cccc12)C(=O)CCC(=O)NC1CCCCC1	0.0	0.0	not tested	not tested	0.0	0.0
SN00785560	CL1344	O=C(OCc1ccccc1)Cn1cnc2n(ncc2c1=O)c1ccccc1	0.0	0.0	not tested	not tested	0.0	0.0
SN00785569	CL1344	Cc1ccc(cc1C)n1ncc2c(=O)n(cnc12)Cc1ccccc1	0.0	0.0	not tested	not tested	0.0	0.0
SN00786676	CL7184	Fc1ccc(cc1)c1nc2N(C(=O)C)[C@H]3CCCC[C@H]3C3(CCCCC3)n2n1	0.0	0.0	not tested	not tested	0.0	0.0
SN00786689	CL9254	CC(=O)N1CCN(CC1)C(c1ccccc1)c1oc2ccccc2c1NC(=O)c1ccccc1	0.0	0.0	not tested	not tested	0.0	0.0
SN00786692	CL9254	COc1cccc(c1)C(N1CCOCC1)c1oc2ccccc2c1NC(=O)c1ccccc1	0.0	0.0	not tested	not tested	0.0	0.0
SN00787879	CL5266	CCOC(=O)CC(NC(=O)N1CCC(CC1)CN1CCOCC1)c1ccccc1	0.0	0.0	not tested	not tested	0.0	0.0
SN00787882	CL5266	O=C(NC12CC3CC(CC(C3)C2)C1)N1CCC(CC1)CN1CCCC1	0.0	0.0	not tested	not tested	0.0	0.0
SN00787962	CL5424B	O=N(=O)c1ccc(cc1)N1CCN(CC1)c1ncnc2c1nc1CCCCCn21	0.0	0.0	not tested	not tested	0.0	0.0
SN00787965	CL5424B	CCc1ccc(cc1)Nc1ncnc2c1nc1CCCCCn21	0.0	0.0	not tested	not tested	0.0	0.0
SN00787972	CL3428	Cc1ccc(cc1)S(=O)(=O)Nc1cncc(c1)C(=O)N1CCN(CC1)Cc1ccccc1	0.0	0.0	not tested	not tested	0.0	0.0
SN00787977	CL3428	COc1ccc(cc1)NC(=O)c1cncc(c1)NS(=O)(=O)c1ccc(C)cc1	0.0	0.0	not tested	not tested	0.0	0.0
SN00789042	CL4903	COc1cccc(c1)c1nn(Cc2nc(oc2C)c2ccccc2)c(=O)c2noc(C)c12	0.0	0.0	not tested	not tested	0.0	0.0
SN00789069	CL4903	COc1ccc(Br)cc1c1oc(C)c(n1)Cn1nc(C)c2c(C)onc2c1=O	0.0	0.0	not tested	not tested	0.0	0.0
SN00789083	CL4852	Brc1cccc(c1)NC(=O)C1CC2CSCN2C21C(=O)Nc1ccccc21	0.0	0.0	not tested	not tested	0.0	0.0
SN00790183	CL1459	O=C(C1CCN(CC1)c1ccc2nncn2n1)N1CCCCCC1	0.0	0.0	not tested	not tested	0.0	0.0
SN00790193	CL7966A	COc1ccc2cc([nH]c2c1)C(=O)N1CCC(C)(CC1)C(=O)NCc1ccccc1	0.0	0.0	not tested	not tested	0.0	0.0
SN00790195	CL7966A	COc1ccc(cc1)CNC(=O)C1(C)CCN(CC1)C(=O)CSc1ccccc1	0.0	0.0	not tested	not tested	0.0	0.0
SN00790217	CL7966B	Cc1ccc(cc1)S(=O)(=O)N1CCC(C)(CC1)C(=O)NCc1ccccc1	0.0	0.0	not tested	not tested	0.0	0.0
SN00791322	CL6359	Brc1ccc(cc1)n1nnc(c2nsc(NC(=O)c3cccs3)n2)c1C	0.0	0.0	not tested	not tested	0.0	0.0
SN00791391	CL6150	Cc1ccc(cc1)c1noc(n1)C1CC(=O)N(C1)c1cccc(Cl)c1	0.0	0.0	not tested	not tested	0.0	0.0
SN00792419	CL7194	CSc1cccc(NC(=O)CN2C(=O)CCn3nc(cc23)c2cn(C)c3ccccc23)c1	0.0	0.0	not tested	not tested	0.0	0.0
SN00792463	CL7138	Fc1ccc(cc1)Nc1nc(nc2ccccc12)C(=O)N1CCCCCC1	0.0	0.0	not tested	not tested	0.0	0.0
SN00792476	CL5508A	COCCCN(Cc1ccco1)C(=O)Cn1cnc2onc(c3ccc(Cl)cc3)c2c1=O	0.0	0.0	not tested	not tested	0.0	0.0
SN00792506	CL5449B	Clc1ccc(cc1)c1noc(n1)C1CCCN(C1)S(=O)(=O)c1ccc(Br)cc1	0.0	0.0	not tested	not tested	0.0	0.0
SN00792525	CL6074	Fc1ccc(cc1)c1noc(n1)c1c[nH]c2ccccc2c1=O	0.0	0.0	not tested	not tested	0.0	0.0
SN00792530	CL6074	COc1cc(OC)cc(c1)c1noc(n1)c1cn(C)c2ccccc2c1=O	0.0	0.0	not tested	not tested	0.0	0.0
SN00781491	SC014211	CCCCCC(=O)N1CCC(CC1)c1scc(n1)c1ccncc1	0.0	0.0	not tested	not tested	0.0	0.0
SN00781514	SC014212	CCC(N1CCC(CC1)c1noc(C)n1)c1ccc(F)cc1	0.0	0.0	not tested	not tested	0.0	0.0
SN00783913	CL1652	COc1ccc(cc1)c1nc2cc(nn2c(c1)C(F)(F)F)C(=O)NCC(C)C	0.0	0.0	not tested	not tested	0.0	0.0
SN00783915	CL3387	Clc1ccc(cc1)C1=NOC(C1)C(=O)N1CCCCC1	0.0	0.0	not tested	not tested	0.0	0.0
SN00785004	CL2764	O=C(Nc1ccc(cc1)OC(F)(F)F)C1CCCN(C1)S(=O)(=O)c1c(C)n[nH]c1C	0.0	0.0	not tested	not tested	0.0	0.0
SN00785021	CL2508	CCOc1ccc2nc(NC(=O)C3CCN(CC3)S(=O)(=O)c3c(C)nn(C)c3C)sc2c1	0.0	0.0	not tested	not tested	0.0	0.0
SN00785041	CL2799	CCCN1Cc2cccc(C(=O)Nc3ccc(F)cc3)c2C1=O	0.0	0.0	not tested	not tested	0.0	0.0
SN00785060	CL2799	CCOC(=O)N1CCC(CC1)N1Cc2cccc(C(=O)Nc3ccc(cc3)C(=O)C)c2C1=O	0.0	0.0	not tested	not tested	0.0	0.0
SN00787121	CL6511	COC(=O)c1cc2c(ccn(c3ccccc3Cl)c2=O)nc1C	0.0	0.0	not tested	not tested	0.0	0.0
SN00787128	CL1162B	Cc1nc2nc(C)nn2c(c1)N1CCN(CC1)Cc1ccccc1	0.0	0.0	not tested	not tested	0.0	0.0
SN00787138	CL4175	O=C(Nc1cccc(c1)C(F)(F)F)c1oc2CCc3cn[nH]c3c2c1C	0.0	0.0	not tested	not tested	0.0	0.0
SN00787272	CL3683	CCCC(=O)Nc1nnc(s1)S(=O)(=O)N(C)Cc1ccco1	0.0	0.0	not tested	not tested	0.0	0.0
SN00787291	CL3682	CCOc1ccc2nc(sc2c1)NC(=O)c1cc2c(nc3ccccn3c2=O)n1C	0.0	0.0	not tested	not tested	0.0	0.0
SN00787328	CL4405B	COc1c(cccc1OC)CNC(=O)C1CCCN(C1)C(=O)N1CC(C)Oc2ccccc12	0.0	0.0	not tested	not tested	0.0	0.0
SN00787335	CL4405B	CC1Oc2ccc(C)cc2N(C1)C(=O)N1CCCC(C1)C(=O)NCc1ccc2OCOc2c1	0.0	0.0	not tested	not tested	0.0	0.0
SN00787356	CL4804	COc1ccccc1NC(=O)c1ccc(cc1)CN1CC(=O)N2CCCC2C1=O	0.0	0.0	not tested	not tested	0.0	0.0
SN00787358	CL4804	CN(CCCNC(=O)c1ccc(cc1)CN1CC(=O)N2CCCC2C1=O)Cc1ccccc1	0.0	0.0	not tested	not tested	0.0	0.0
SN00788328	CL5552A	CCN1CCCC1CNC(=O)C1CCN(CC1)Cc1onc(n1)c1ccc(OC)c(OC)c1	0.0	0.0	not tested	not tested	0.0	0.0

Compound ID	Scaffold	Smiles	% Inhibition n=1		% Inhibition n=2		Average % Inhibition	
			24h	48h	24h	48h	24h	48h
SN00788330	CL5552A	CCCN(CCC)CCNC(=O)C1CCN(CC1)Cc1onc(n1)c1ccc(OC)c(OC)c1	0.0	0.0	not tested	not tested	0.0	0.0
SN00788496	CL6065	O=C1CS(=O)(=O)C2(C(=O)N(Cc3ccccc3)c3ccccc23)N1c1ccccc1	0.0	0.0	not tested	not tested	0.0	0.0
SN00788542	CL5306	CCOc1ccc(cc1)NC(=O)N1CCCn2nc(C)cc12	0.0	0.0	not tested	not tested	0.0	0.0
SN00788577	CL7467B	CCOC(=O)Cc1csc(n1)NC(=O)C1CCN(CC1)c1ncnc2onc(C)c12	0.0	0.0	not tested	not tested	0.0	0.0
SN00788591	CL7467B	O=C(C1CCN(CC1)c1ncnc2onc(C)c12)N1CCN(CC1)Cc1ccc2OCOc2c1	0.0	0.0	not tested	not tested	0.0	0.0
SN00789642	CL8009	O=C(Nc1ccc(cc1)c1noc(n1)C1CCC1)N1CCN(CC1)c1ccc(cc1)C(=O)C	0.0	0.0	not tested	not tested	0.0	0.0
SN00789672	CL8016	COc1ccc(cc1)CNS(=O)(=O)c1scc(c1)C(=O)N1CCCCCC1	0.0	0.0	not tested	not tested	0.0	0.0
SN00789685	CL8017	CCCn1c2ccc(cc2nc(C)c1=O)c1noc(n1)c1cc2ccccc2oc1=O	0.0	0.0	not tested	not tested	0.0	0.0
SN00789689	CL8017	CCCn1c(=O)c(C)nc2cc(ccc12)c1noc(n1)c1cccc(NC(=O)c2ccco2)c1	0.0	0.0	not tested	not tested	0.0	0.0
SN00789703	CL8018	O=c1[nH]c2cc(ccc2n(C)c1=O)c1noc(n1)C1(CC1)c1ccccc1	0.0	0.0	not tested	not tested	0.0	0.0
SN00789729	CL8018	CC(C)Cn1c2ccc(cc2[nH]c(=O)c1=O)c1noc(n1)c1ccccc1	0.0	0.0	not tested	not tested	0.0	0.0
SN00789733	CL8884	CCCn1c(=O)[nH]c2cc(ccc12)c1noc(n1)CN1C(=O)COc2ccccc12	0.0	0.0	not tested	not tested	0.0	0.0
SN00790784	CL7515	Cc1ccc(cc1)C(=O)N1CCN(CC1)S(=O)(=O)c1c(C)nn(C(C)C)c1C	0.0	0.0	not tested	not tested	0.0	0.0
SN00791914	CL6155	O=C(NC1CCCCC1)c1noc2ncn(C)c(=O)c12	0.0	0.0	not tested	not tested	0.0	0.0
SN00791915	CL6155	Cc1ccc(C)c(c1)N1CCN(CC1)C(=O)c1noc2ncn(C)c(=O)c12	0.0	0.0	not tested	not tested	0.0	0.0
SN00791919	CL5797	COC(=O)c1ncn2CC(C)(C(=O)NCc3ccccc3)N(Cc3ccc(C)cc3)C(=O)c12	0.0	0.0	not tested	not tested	0.0	0.0
SN00791920	CL5797	COC(=O)c1ncn2CC(C)(C(=O)NCc3ccccc3)N(CCCc3ccccc3)C(=O)c12	0.0	0.0	not tested	not tested	0.0	0.0
SN00791977	CL3167	Cc1ccc2[nH]c(CCNC(=O)C3CCCN(C3)S(=O)(=O)c3ncn(c3)C(C)C)nc2c1	0.0	0.0	not tested	not tested	0.0	0.0
SN00793062	CL4660A	Fc1ccc(cc1)N1CCN(CC1)C(=O)C1CCN(CC1)c1onc(n1)c1ccccc1	0.0	0.0	not tested	not tested	0.0	0.0
SN00793174	CL2892	O=c1[nH]c2cc(Cl)c(cc2[nH]1)S(=O)(=O)Nc1ccc(C)c(C)c1	0.0	0.0	not tested	not tested	0.0	0.0
SN00797793	CL9393	O=C(Nc1ccc(cc1)C(C)C)C1CCN(CC1)c1nccc2sccc12	0.0	0.0	not tested	not tested	0.0	0.0
SN00782415	SC008115	Cc1onc(CN2CCCN(CC2)C(=O)C)c1	0.0	0.0	not tested	not tested	0.0	0.0
SN00784877	CL2541	CCOc1ccc(CCNC(=O)c2nnn(CC(=O)Nc3ccc(C)cc3)c2N)cc1OCC	0.0	0.0	not tested	not tested	0.0	0.0
SN00784910	CL3314	CN(CCNC(=O)c1ccc(cc1)N1CCCC1=O)Cc1ccccc1	0.0	0.0	not tested	not tested	0.0	0.0
SN00786007	CM2505	OC(=O)c1cnc(nc1C)N1CCOCC1	0.0	0.0	not tested	not tested	0.0	0.0
SN00787202	CL4606	Cc1ccc(cc1)Cn1c(=O)c2ccccc2n2c(nnc12)c1ccc(C)s1	0.0	0.0	not tested	not tested	0.0	0.0
SN00787205	CL4606	Fc1ccc(cc1)Cn1c(=O)c2ccccc2n2c(nnc12)c1cccs1	0.0	0.0	not tested	not tested	0.0	0.0
SN00788383	CL7467	Cc1noc2ncnc(N3CCN(CC3)Cc3ccc4OCOc4c3)c12	0.0	0.0	not tested	not tested	0.0	0.0
SN00792917	CL7284A	CCOc1ccc(cc1)n1nnnc1CNS(=O)(=O)C	0.0	0.0	not tested	not tested	0.0	0.0
SN00792927	CL7284A	CCS(=O)(=O)NCc1nnnn1c1ccc(C)cc1	0.0	0.0	not tested	not tested	0.0	0.0
SN00794077	CL8321	Cc1onc(c1)NC(=O)CN1c2ccccc2NC2=C(C(=O)CC(C)(C)C2)C1c1ccccc1	0.0	0.0	not tested	not tested	0.0	0.0
SN00794126	CL8331	O=C(NCc1ccc2OCCCOc2c1)c1ccco1	0.0	0.0	not tested	not tested	0.0	0.0
SN00795265	CL7767A	COc1cccc(c1)Cc1onc(n1)c1ccc(cc1)NS(=O)(=O)c1ccccc1	0.0	0.0	not tested	not tested	0.0	0.0
SN00795281	CL8091	COc1ccc(cc1)c1c(nn2c(C)cc(=O)[nH]c12)C(=O)Nc1ccc(Cl)cc1C	0.0	0.0	not tested	not tested	0.0	0.0
SN00796470	CL7880	O=c1[nH]c2ccccc2nc1CN1CCN(CC1)c1cccc(c1)C(F)(F)F	0.0	0.0	not tested	not tested	0.0	0.0
SN00796478	CL7880	Cc1ccc(cc1)N1CCN(CC1)Cc1nc2ccccc2[nH]c1=O	0.0	0.0	not tested	not tested	0.0	0.0
SN00796502	CL4472	CCc1nc(sc1C(=O)NCc1cccc(OC)c1)N1CCCC1=O	0.0	0.0	not tested	not tested	0.0	0.0
SN00797528	CL6358	COc1ccc(cc1)c1noc(n1)c1ccc(=O)n(CC(=O)N2CCN(CC2)c2ccc(F)cc2)c1	0.0	0.0	not tested	not tested	0.0	0.0
SN00797550	CL4671	O=C(N1CCCCC1)c1noc2ccc(cc12)S(=O)(=O)N1CCN(CC1)Cc1ccccc1	0.0	0.0	not tested	not tested	0.0	0.0
SN00797564	CL9398A	CC(=O)Nc1cccc(c1)N1CC(CC1=O)C(=O)NCCC1=CCCCC1	0.0	0.0	not tested	not tested	0.0	0.0
SN00781574	SC014215	O=C(CCn1cncc1)N1CCCCC1c1scc(n1)C(C)(C)C	0.0	0.0	not tested	not tested	0.0	0.0
SN00781581	SC014453	Cc1oc(nc1CSc1n[nH]c(=O)n1C)c1cscc1	0.0	0.0	not tested	not tested	0.0	0.0
SN00781600	SC014453	O=C(Cc1nc(oc1C)c1cscc1)N1CCn2c(nnc2C)C1	0.0	0.0	not tested	not tested	0.0	0.0
SN00782659	SC015216	CC(N1CCn2c(nnc2c2ccccc2)C1)c1onc(n1)C(C)C	0.0	0.0	not tested	not tested	0.0	0.0
SN00782660	SC015354	CC1CN(CC2CCCO2)CC1N1CCN(CC1)C(=O)C	0.0	0.0	not tested	not tested	0.0	0.0
SN00782662	SC015371	CCOc1cccc(c1)C(=O)N1CCN(CC1)CC1CCCCN1C	0.0	0.0	not tested	not tested	0.0	0.0

Compound ID	Scaffold	Smiles	% Inhibition n=1		% Inhibition n=2		Average % Inhibition	
			24h	48h	24h	48h	24h	48h
SN00782707	SC000870	Cc1onc(CN2CCN(CC2)C(=O)c2cc(C)nn2C)c1	0.0	0.0	not tested	not tested	0.0	0.0
SN00784044	CL2120	CCC(=O)Nc1nc(COc2ccc(F)cc2)nc(n1)N(C)C	0.0	0.0	not tested	not tested	0.0	0.0
SN00784053	CL1530	Fc1ccc(cc1)NC(=O)c1n[nH]nc1N	0.0	0.0	not tested	not tested	0.0	0.0
SN00784056	CL1281	CCn1c(Oc2cccnc2)nc2c1c(=O)n(C)c(=O)n2C	0.0	0.0	not tested	not tested	0.0	0.0
SN00785145	CL5373	Cc1ccc(cc1)CCN1C(=O)Cn2cccc2C1C(=O)NC1CCCCC1C	0.0	0.0	not tested	not tested	0.0	0.0
SN00785155	CL3167	O=C(NC1CCCCC1C)C1CCCN(C1)S(=O)(=O)c1c[nH]cn1	0.0	0.0	not tested	not tested	0.0	0.0
SN00785186	CL3222	Cc1ccc(cc1)S(=O)(=O)N1CCN(CC1)C(=O)c1[nH]nc(C)c1	0.0	0.0	not tested	not tested	0.0	0.0
SN00786329	CL7370	Cc1cccc(c1)c1noc(n1)c1cccnc1N1CCOCC1	0.0	0.0	not tested	not tested	0.0	0.0
SN00786363	CL7376	CCOc1ccc(cc1)C(=O)N1CCCC(C1)c1nc2ccccc2[nH]1	0.0	0.0	not tested	not tested	0.0	0.0
SN00787475	CL4509	CC(=O)N1CCOc2cc(c(C)cc12)S(=O)(=O)N1CCc2ccccc12	0.0	0.0	not tested	not tested	0.0	0.0
SN00787489	CL4509	CCOC(=O)c1cccc(c1)NS(=O)(=O)c1cc2OCCN(C(=O)C)c2cc1Cl	0.0	0.0	not tested	not tested	0.0	0.0
SN00787506	CL3592	O=C(N1CCC(=CC1)c1ccccc1)c1ccc2c(c1)nc1CCCCn1c2=O	0.0	0.0	not tested	not tested	0.0	0.0
SN00788671	CL1247	Cc1ncn(c1)S(=O)(=O)c1cccs1	0.0	0.0	not tested	not tested	0.0	0.0
SN00788693	CL4247	CCN1CCc2nc(sc2C1)NC(=O)c1ccccc1	0.0	0.0	not tested	not tested	0.0	0.0
SN00788728	CL6477	Fc1ccc(cc1)N1CCN(CC1)c1ccc2cc(ccc2n1)S(=O)(=O)N1CCCC1	0.0	0.0	not tested	not tested	0.0	0.0
SN00789795	CL6993	COc1ccc(cc1)CNC(=O)c1ccc(s1)S(=O)(=O)N1CCC(C)CC1	0.0	0.0	not tested	not tested	0.0	0.0
SN00789858	CL7967A	CCOc1ccc(cc1)S(=O)(=O)N1CCc2n(C)nc(C(=O)N3CCCC3)c2C1	0.0	0.0	not tested	not tested	0.0	0.0
SN00789878	CL7967B	CCOC(=O)Cc1ccc(cc1)NC(=O)N1CCc2n(C)nc(C(=O)N3CCCCC3)c2C1	0.0	0.0	not tested	not tested	0.0	0.0
SN00789892	CL7967B	CCCNC(=O)N1CCc2n(C)nc(C(=O)Nc3ccccc3)c2C1	0.0	0.0	not tested	not tested	0.0	0.0
SN00791027	CL7785B	COc1ccc(CC(=O)N2CCCN(CC2)c2ccc(=O)n(n2)c2ccccc2)c(Cl)c1	0.0	0.0	not tested	not tested	0.0	0.0
SN00792156	CL4105	O=C(CCc1nc2c(C)nn(c3ccccc3)c2n(C)c1=O)Nc1cccc(CN2CCCC2)c1	0.0	0.0	not tested	not tested	0.0	0.0
SN00795567	CL8922A	COC(=O)c1cccc(NC(=O)Nc2cc(F)c3nc(ccc3c2)N2CCOCC2)c1	0.0	0.0	not tested	not tested	0.0	0.0
SN00796659	CL9306	Cc1ccc(cc1)N(C)S(=O)(=O)c1scc(c1)c1onc(n1)c1ccc(Cl)cc1	0.0	0.0	not tested	not tested	0.0	0.0
SN00796685	CL9306	Fc1cccc(c1)c1noc(n1)c1csc(c1)S(=O)(=O)N1CCCCC1	0.0	0.0	not tested	not tested	0.0	0.0
SN00796701	CL9325	Cc1nnc(o1)c1csc(c1)S(=O)(=O)NCc1ccccc1	0.0	0.0	not tested	not tested	0.0	0.0
SN00796709	CL9325	CCc1nnc(o1)c1csc(c1)S(=O)(=O)Nc1ccc(F)cc1C	0.0	0.0	not tested	not tested	0.0	0.0
SN00787404	CL4469B	Brc1cccc(CNC(=O)C2CCCN(C2)c2nc3ccccc3n3cccc23)c1	0.0	0.0	not tested	not tested	0.0	0.0
SN00789800	CL6993	O=C(NCCc1ccco1)c1ccc(s1)S(=O)(=O)N1CCCCCC1	0.0	0.0	not tested	not tested	0.0	0.0
SN00789820	CL7744	COCCNC(=O)C1(C)Cn2c(cc3ccsc23)C(=O)N1Cc1ccco1	0.0	0.0	not tested	not tested	0.0	0.0
SN00792173	CL6075	O=C(Nc1ccccc1Br)CS(=O)(=O)c1ccc2NC=NS(=O)(=O)c2c1	0.0	0.0	not tested	not tested	0.0	0.0
SN00793273	CL7349	Cc1occ(n1)c1ccc(cc1)S(=O)(=O)NC1CCCCCC1	0.0	0.0	not tested	not tested	0.0	0.0
SN00793275	CL7349	CCOC(=O)c1cccc(c1)NS(=O)(=O)c1ccc(cc1)c1coc(C)n1	0.0	0.0	not tested	not tested	0.0	0.0
SN00793311	CL8009	CSc1cccc(NC(=O)Nc2ccc(cc2)c2noc(C)n2)c1	0.0	0.0	not tested	not tested	0.0	0.0
SN00794383	CL4855	Cc1ccc(cc1)CNC1=NS(=O)(=O)c2ccccc2N1	0.0	0.0	not tested	not tested	0.0	0.0
SN00799030	CL9740A	COc1ccc(cc1)c1ncc2CN(CCc2n1)C(=O)Nc1cccc(C)c1C	0.0	0.0	not tested	not tested	0.0	0.0
SN00793209	CL7156	Clc1ccc(cc1)c1nn2ccn(Cc3nc(oc3C)c3ccccc3)c(=O)c2c1	0.0	0.0	not tested	not tested	0.0	0.0
SN00795553	CL8922A	COc1cccc(CNC(=O)Nc2cc(F)c3nc(ccc3c2)N2CCCCC2)c1	0.0	0.0	not tested	not tested	0.0	0.0
SN00796726	CL9208	Cn1nc(c2onc(n2)c2ccccc2F)c2CN(CCc12)C(=O)c1ccc(C)c(C)c1	0.0	0.0	not tested	not tested	0.0	0.0
SN00796727	CL9208	COc1cccc(c1)CC(=O)N1CCc2n(C)nc(c3onc(n3)c3ccccc3F)c2C1	0.0	0.0	not tested	not tested	0.0	0.0
SN00799403	CL9953	COc1cccc(c1)c1nn2ccn(CCC(=O)N3CCN(CC3)C(=O)C)c(=O)c2c1	0.0	0.0	not tested	not tested	0.0	0.0
SN00799489	CL2046	Cc1ccc(cc1)NC(=O)C1=NN(CC(=O)Nc2ccc3OCCOc3c2)C(=O)CC1	0.0	0.0	not tested	not tested	0.0	0.0
SN00799516	CL6598	CC1CCN(CC1)Cc1csc(n1)N1CCN(CC1)C(=O)COc1ccccc1	0.0	0.0	not tested	not tested	0.0	0.0
SN00800531	CM1467	COc1cccc(CCC(=O)N2CCCN(CC2)c2ncccc2c2onc(n2)C2CC2)c1	0.0	0.0	not tested	not tested	0.0	0.0
SN00801663	CM1450	Fc1ccc(cc1)c1noc(n1)C1CCCN1C(=O)C1CCN(CC1)C(=O)c1ccccc1	0.0	0.0	not tested	not tested	0.0	0.0
SN00801756	CM1574	O=C(OCc1onc(n1)c1ccc(cc1)OC(F)(F)F)C1=CN(C)S(=O)(=O)N=C1C	0.0	0.0	not tested	not tested	0.0	0.0
SN00801768	CM1574	O=C(OCc1ccc(cc1)C(C)(C)C)C1=CN(C)S(=O)(=O)N=C1C	0.0	0.0	not tested	not tested	0.0	0.0

Compound ID	Scaffold	Smiles	% Inhibition n=1		% Inhibition n=2		Average % Inhibition	
			24h	48h	24h	48h	24h	48h
SN00801821	CM0270	CCn1c2cc(ccc2n2nccc2c1=O)C(=O)NCCc1nc2ccccc2n1C	0.0	0.0	not tested	not tested	0.0	0.0
SN00802849	CL7567	CCN1CCN(CC1)Cc1nc(Nc2ccc(F)cc2)nc(N)n1	0.0	0.0	not tested	not tested	0.0	0.0
SN00802881	CL2573	COc1ccc(cc1)NC(=O)Cn1c(=O)n(CC)c(=O)c2ccccc12	0.0	0.0	not tested	not tested	0.0	0.0
SN00799335	CL9946	COc1ccc(CNS(=O)(=O)c2c(nc3sc(C)cn23)C(C)C)c(OC)c1	0.0	0.0	not tested	not tested	0.0	0.0
SN00799356	CL9947	CC(C)c1sc2nc(C)c(n2n1)S(=O)(=O)NCc1ccc2OCOc2c1	0.0	0.0	not tested	not tested	0.0	0.0
SN00800286	CM1091	CN1CCC(CC1)NC(=O)c1n[nH]c2CCN(Cc12)C(=O)c1ccccc1	0.0	0.0	not tested	not tested	0.0	0.0
SN00800295	CM1254	O=C(N1CCN(CC1)c1ncccn1)c1cccnc1N1CCNC(C1)c1ccccc1	0.0	0.0	not tested	not tested	0.0	0.0
SN00800373	CM1205	CC(=O)Nc1ccc(cc1)C(=O)NC1CCN(CC1)c1[nH]nc(c1)c1ccc(F)cc1	0.0	0.0	not tested	not tested	0.0	0.0
SN00800481	CM1476	COc1cccc(CCC(=O)NC2(CCCCC2)c2onc(C)n2)c1	0.0	0.0	not tested	not tested	0.0	0.0
SN00800606	CM1426	Cc1ccc(cc1)CNS(=O)(=O)c1cn2CCCNC(=O)c2c1	0.0	0.0	not tested	not tested	0.0	0.0
SN00800634	CM1451	CN(C)CCCNS(=O)(=O)c1ccc(o1)c1[nH]ncc1C(=O)N1CCCCC1	0.0	0.0	not tested	not tested	0.0	0.0
SN00800636	CM1451	O=C(N1CCCCC1)c1cn[nH]c1c1ccc(o1)S(=O)(=O)NCc1ccco1	0.0	0.0	not tested	not tested	0.0	0.0
SN00800676	CM1452	CN(C)C(=O)c1cn[nH]c1c1oc(C)c(c1)S(=O)(=O)N1CCN(CC1)c1ccccn1	0.0	0.0	not tested	not tested	0.0	0.0
SN00801493	CM1393	COCCCNc1ncnc2sc3CN(CCc3c12)Cc1ccco1	0.0	0.0	not tested	not tested	0.0	0.0
SN00801507	CM2368	CNC(=O)N1C[C@@H](CN(C)C)[C@H](C1)C1CCN(CC1)Cc1ccccn1	0.0	0.0	not tested	not tested	0.0	0.0
SN00802925	CL0948	CCn1cc(C(=O)Nc2ccc(O)cc2)c(=O)c2cc(F)c(cc12)N1CCN(CC1)C(=O)C	0.0	0.0	not tested	not tested	0.0	0.0
SN00802965	CL3097	CCCCC(=O)Nc1ccc(N2CCOCC2)c2nonc12	0.0	0.0	not tested	not tested	0.0	0.0
SN00807835	SC012620	O=C(Nc1nc2ccccc2[nH]1)c1c[nH]n2c1nc(=O)c1ccccc21	0.0	0.0	not tested	not tested	0.0	0.0
SN00807842	SC012433	Clc1ccc(cc1)CN1CCCN(CC1)C(=O)c1ccc([nH]c1=O)c1ccco1	0.0	0.0	not tested	not tested	0.0	0.0
SN00781631	SC014597	Fc1ccc(cc1)N1CCN(CC1)C(=O)CCc1nnc2n1ccn(c1cc(C)cc(C)c1)c2=O	0.0	0.0	not tested	not tested	0.0	0.0
SN00787546	CL5109A	O=C(NCCCc1ccccc1)CN1C(=O)C(Oc2cccnc12)c1ccccc1	0.0	0.0	not tested	not tested	0.0	0.0
SN00787554	CL5109A	CCN1CCN(CCCNC(=O)CN2c3ncccc3OC(c3ccccc3)C2=O)CC1	0.0	0.0	not tested	not tested	0.0	0.0
SN00787566	CL4811	O=C1c2cc3ccccc3n2CC(C)(C(=O)NCC2CCCO2)N1C1CCCc2ccccc12	0.0	0.0	not tested	not tested	0.0	0.0
SN00787585	CL5111A	CCOCCCNC(=O)Cn1c(=O)c2cccn2c2cccnc12	0.0	0.0	not tested	not tested	0.0	0.0
SN00799035	CL9740A	COc1cccc(c1)NC(=O)N1CCc2nc(ncc2C1)c1ccc(F)cc1	0.0	0.0	not tested	not tested	0.0	0.0
SN00800467	CM1392	CCN(c1ccccc1)C(=O)c1nc(C)n(C2CCN(CC2)S(=O)(=O)C)c1C	0.0	0.0	not tested	not tested	0.0	0.0
SN00801437	CM3061	O=C(CN1CCCC1=O)N1CCC(CC1)Cc1ccccc1	0.0	0.0	not tested	not tested	0.0	0.0
SN00801445	CM2464	CCC(=O)N1C[C@@H]2[C@H](C1)C(=O)N(Cc1ccc3OCOc3c1)C2=O	0.0	0.0	not tested	not tested	0.0	0.0
SN00801470	CM2464	O=C1CCCN1CCCN1C(=O)[C@@H]2CN(Cc3cccs3)C[C@@H]2C1=O	0.0	0.0	not tested	not tested	0.0	0.0
SN00801540	CM2931	CN1CCC(CC1)OC1CCN(CC1)C(=O)c1ccccn1	0.0	0.0	not tested	not tested	0.0	0.0
SN00801582	CM3040	CCC(=O)N1CCC(CC1)C1CCN(CCC(C)C)C1	0.0	0.0	not tested	not tested	0.0	0.0
SN00801606	CM1573	O=C(Cn1cccc1c1onc(n1)c1ccccc1)N1CCc2ccccc12	0.0	0.0	not tested	not tested	0.0	0.0
SN00801625	CM1573	COc1ccc(cc1F)c1noc(n1)c1cccn1CC(=O)Nc1cccc(F)c1	0.0	0.0	not tested	not tested	0.0	0.0
SN00802571	CL7245	Fc1ccc(cc1)Cn1c(=O)c(nn(c2ccccc2)c1=O)C(=O)N1CCOCC1	0.0	0.0	not tested	not tested	0.0	0.0
SN00802603	CL2726A	O=C(N1CCN(CC1)c1ccccc1F)C1CC(=O)N(Cc2ccccc2)C1	0.0	0.0	not tested	not tested	0.0	0.0
SN00802654	CL3391	COc1cccc(c1)n1nnc2c(=O)n(cnc12)CC(=O)N1CCCC1	0.0	0.0	not tested	not tested	0.0	0.0
SN00802697	CL1058	Fc1c(F)cc(F)c(F)c1N1C(=O)c2ccncc2C1=O	0.0	0.0	not tested	not tested	0.0	0.0
SN00802722	CL5077	O=C(C(C)C)N1CCN(CC1)c1nc2ccccc2c2nnc(c3cccs3)n12	0.0	0.0	not tested	not tested	0.0	0.0
SN00802747	CM1616	COc1cccc(c1)NC(=O)N1CCCCC1c1onc(n1)c1ccc(Br)cc1	0.0	0.0	not tested	not tested	0.0	0.0
SN00807771	SC012620	Clc1ccc(s1)C(C)NC(=O)c1c[nH]n2c1nc(=O)c1ccccc21	0.0	0.0	not tested	not tested	0.0	0.0
SN00781612	SC014472	FC(F)CN1CCN(CC1)c1nc(nc2CCCc12)c1ccncc1	0.0	0.0	not tested	not tested	0.0	0.0
SN00782769	SC001166	COCCn1nc(ccc1=O)C(=O)N1CC(C)OCC1C	0.0	0.0	not tested	not tested	0.0	0.0
SN00786413	CL1728	COC(=O)c1ccc(cc1)c1nc2cnccn2c1Nc1cccc(c1)C(=O)OC	0.0	0.0	not tested	not tested	0.0	0.0
SN00786422	CL8245	Cc1nc(cc(n1)N(C)C)N1CCN(CC1)C(=O)c1cc2ccccc2oc1=O	0.0	0.0	not tested	not tested	0.0	0.0
SN00787518	CL4157	Fc1cccc(CNC(=O)Cn2cc3CCc4oc(C(=O)N5CCOCC5)c(C)c4c3n2)c1	0.0	0.0	not tested	not tested	0.0	0.0
SN00787519	CL4157	CCOC(=O)N1CCN(CC1)C(=O)Cn1nc2c(CCc3oc(C(=O)N4CCCC4)c(C)c23)c1	0.0	0.0	not tested	not tested	0.0	0.0

Compound ID	Scaffold	Smiles	% Inhibition n=1		% Inhibition n=2		Average % Inhibition	
			24h	48h	24h	48h	24h	48h
SN00794462	CL2258	O=C(Nc1ccc(cc1)C(C)C)c1ccc(F)c(c1)S(=O)(=O)N1CCN(CC1)c1ccccc1	0.0	0.0	not tested	not tested	0.0	0.0
SN00794482	CL5373	Cc1ccc(cc1)CN1C(=O)Cn2cccc2C1C(=O)NC1CCCCCCC1	0.0	0.0	not tested	not tested	0.0	0.0
SN00795580	CL6768A	COc1ccc(cc1)S(=O)(=O)N1CCCN(CC1)c1sc2ncccc2n1	0.0	0.0	not tested	not tested	0.0	0.0
SN00795617	CL9166	O=C(N1CCN(CC1)c1ccccc1)c1onc(n1)c1ccc(cc1)N1CCCCCC1	0.0	0.0	not tested	not tested	0.0	0.0
SN00795626	CL9166	CC1CCN(CC1)c1ccc(cc1)c1noc(n1)C(=O)N1CCN(CC1)C(=O)c1sccc1C	0.0	0.0	not tested	not tested	0.0	0.0
SN00795633	CL6150	Clc1cccc(c1)N1CC(CC1=O)c1onc(n1)c1ccc(cc1)c1csc(C)n1	0.0	0.0	not tested	not tested	0.0	0.0
SN00795648	CL8804	c1ccc(cc1)c1scc(n1)c1ccc(cc1)c1noc(n1)c1ccncc1	0.0	0.0	not tested	not tested	0.0	0.0
SN00796747	CL9208A	Fc1ccc(cc1)NC(=O)N1CCc2n(C)nc(c3onc(C)n3)c2C1	0.0	0.0	not tested	not tested	0.0	0.0
SN00799100	CL9760	O=C(N(Cc1ccccc1)Cc1ccco1)c1ccc(cc1)c1ccnc(n1)N1CCCC1	0.0	0.0	not tested	not tested	0.0	0.0
SN00800137	CM0330A	O1CCN(CC1)c1nccnc1OC1CNCC1	0.0	0.0	not tested	not tested	0.0	0.0
SN00801107	CM3131	COc1cccc(c1)CC(=O)N(C)C1CCN(C1)c1nccnc1OC	0.0	0.0	not tested	not tested	0.0	0.0
SN00801173	CM2946	COCCc1noc(COC2CCCN(C2)C(=O)NC2CCCC2)n1	0.0	0.0	not tested	not tested	0.0	0.0
SN00802243	CM1830	CC(C)CCN1CCc2oc(C)c(C(=O)NC3CCN(C)CC3)c2C1	0.0	0.0	not tested	not tested	0.0	0.0
SN00802251	CM1834	CCC1(CCN(C1)C(=O)C(C)C)N1CCN(CC1)C(=O)Nc1cccc(F)c1C	0.0	0.0	not tested	not tested	0.0	0.0
SN00790868	CL8415	Cc1ccc(C)c(c1)NC(=O)C1CCN(CC1)c1nccccc1n1cccc1	0.0	0.0	not tested	not tested	0.0	0.0
SN00790884	CL8199B	COc1ccc(cc1)c1nn2c(n1)sc1CN(CCc21)C(=O)NCC1CCCO1	0.0	0.0	not tested	not tested	0.0	0.0
SN00799998	CM0325	CN1CCC(C1)Oc1ccc(cn1)NC(=O)NCc1ccccc1	0.0	0.0	not tested	not tested	0.0	0.0
SN00800038	CM0327	O=C(CCc1ccccc1)N1CCC(C1)Oc1ncccc1C(=O)N1CCCCC1	0.0	0.0	not tested	not tested	0.0	0.0
SN00800250	CL4901	Fc1ccc2c(c1)[nH]c(=O)c(c1onc(n1)C(C)(C)C)c2O	0.0	0.0	not tested	not tested	0.0	0.0
SN00800258	CL4901	Cc1ccc2[nH]c(=O)c(c3onc(n3)C(C)(C)C)c(O)c2c1	0.0	0.0	not tested	not tested	0.0	0.0
SN00801056	CM3105	COc1ccc(cc1)C(=O)N1CCC(Nc2cccnc2)C1	0.0	0.0	not tested	not tested	0.0	0.0
SN00801280	CM2392	OC[C@H]1CCN(CC2CCCCC2)C[C@H]1N(C)C	0.0	0.0	not tested	not tested	0.0	0.0
SN00801350	CM3034	COCC1CN(CC21CCOCC2)C(=O)COc1ccc(OC)cc1	0.0	0.0	not tested	not tested	0.0	0.0
SN00801363	CM2239	Cc1onc(C)c1CN1CCCCC1c1cccnc1	0.0	0.0	not tested	not tested	0.0	0.0
SN00801368	CM2239	CCCNC(=O)N1CCN(CC1)Cc1c(C)noc1C	0.0	0.0	not tested	not tested	0.0	0.0
SN00801393	CM2247	O=C(c1ccc(F)c(F)c1)N1CCCC(C)(C1)c1onc(n1)C1CC1	0.0	0.0	not tested	not tested	0.0	0.0
SN00802446	CL4868B	Cc1ccc2NC3(CCN(CC3)C(=O)N(C)C)NC(=O)c2c1	0.0	0.0	not tested	not tested	0.0	0.0
SN00802467	CL1367	Cc1cccc(c1)n1nc(nc1c1ccco1)C(=O)NCc1ccncc1	0.0	0.0	not tested	not tested	0.0	0.0
SN00802479	CL6428	O=C(NCCc1ccccn1)c1cc(nc2cc(nn12)c1ccccc1)c1ccccc1	0.0	0.0	not tested	not tested	0.0	0.0
SN00802490	CL3461	O=N(=O)c1ccccc1S(=O)(=O)N1CCn2c(nc3ccccc23)C1	0.0	0.0	not tested	not tested	0.0	0.0
SN00802491	CL3461	O=S(=O)(N1CCn2c(nc3ccccc23)C1)c1ccc2CCCCc2c1	0.0	0.0	not tested	not tested	0.0	0.0
SN00799629	CM0164	Brc1cncc(c1)C(=O)NCc1ccc(cc1)c1scc(C)n1	0.0	0.0	not tested	not tested	0.0	0.0
SN00799705	CM0662A	O=C(NCCC1=CCCCC1)Cn1ccc2ccn(C)c2c1=O	0.0	0.0	not tested	not tested	0.0	0.0
SN00799717	CM0615A	COCc1cc(=O)n(CCN2CCCCC2)c(n1)N1CCCC1	0.0	0.0	not tested	not tested	0.0	0.0
SN00799752	CM0662A	C=CCn1ccc2ccn(Cc3c(C)noc3C)c(=O)c12	0.0	0.0	not tested	not tested	0.0	0.0
SN00799766	CM0219	COc1ccccc1CC(=O)Nc1cnc(C)nc1	0.0	0.0	not tested	not tested	0.0	0.0
SN00799821	CL7439	CCN1CCCc2cc(sc12)C(=O)N1CCOCC1	0.0	0.0	not tested	not tested	0.0	0.0
SN00800050	CM0323	CN1CCC(C1)Oc1ncccc1C(=O)N1CCCCC1	0.0	0.0	not tested	not tested	0.0	0.0
SN00800086	CM0324	CCC(=O)N1CCC(CC1)Oc1ccc(cn1)C(=O)N1CCOCC1	0.0	0.0	not tested	not tested	0.0	0.0
SN00800847	CM1544	COc1nc(OC)ccc1C(=O)N1CCc2nc3cc[nH]n3c(=O)c2C1	0.0	0.0	not tested	not tested	0.0	0.0
SN00800913	CM1886	COCCNC1=Nc2ccccc2CNC21CCN(C2)C(=O)Nc1cc(C)ccc1OC	0.0	0.0	not tested	not tested	0.0	0.0
SN00802042	CM1409	COc1ccccc1N1CCN(CC1)Cc1ccc(=O)n(C)c1	0.0	0.0	not tested	not tested	0.0	0.0
SN00802054	CM1409	CCn1cc(ccc1=O)CN1CCN(CC1)C(=O)CCc1ccccc1	0.0	0.0	not tested	not tested	0.0	0.0
SN00802088	CM1429	CC(=O)N1CCCN(Cc2ccc3OCOc3c2)c2ccccc12	0.0	0.0	not tested	not tested	0.0	0.0
SN00802090	CM1429	COCCN1CCCN(C(=O)c2cccc(Cl)c2)c2ccccc12	0.0	0.0	not tested	not tested	0.0	0.0
SN00802300	CM1835	COCCn1c(=O)c2cc(nn2c2ccccc12)C(=O)NCC1CCCN1CC	0.0	0.0	not tested	not tested	0.0	0.0

Compound ID	Scaffold	Smiles	% Inhibition n=1		% Inhibition n=2		Average % Inhibition	
			24h	48h	24h	48h	24h	48h
SN00802306	CM1855	O=C1CC[C@@]2(CCCC[C@H]2N1C)C(=O)NCc1ccco1	0.0	0.0	not tested	not tested	0.0	0.0
SN00802336	CM1867	NC(=O)C1CNC[C@H]1c1ccncc1	0.0	0.0	not tested	not tested	0.0	0.0
SN00802340	CM1867	O=C(N1CCCCC1)C1CN(C[C@H]1c1ccncc1)C(=O)c1ccncc1	0.0	0.0	not tested	not tested	0.0	0.0
SN00802360	CM2664	COCCN1CCC(N2CCN(CC2)C(=O)c2cccc(c2)C(F)(F)F)C1=O	0.0	0.0	not tested	not tested	0.0	0.0
SN00802367	CM2664	O=C(CCc1nc2ccccc2[nH]1)N1CCN(CC1)C1CCN(C)C1=O	0.0	0.0	not tested	not tested	0.0	0.0
SN00803044	CL6946	O=C(N1CCCCC1)c1cnn2ccc(cc12)C1CCN(CC1)C(=O)C(C)(C)C	0.0	0.0	not tested	not tested	0.0	0.0
SN00803222	CL9788	CCn1cnc2ccc(cc2c1=O)C(=O)N1CCCC1	0.0	0.0	not tested	not tested	0.0	0.0
SN00803242	CL3387	CCCNC(=O)C1ON=C(C)C1	0.0	0.0	not tested	not tested	0.0	0.0
SN00803250	CL0385	O=C(Nc1ccc2nc(oc2c1)C(C)C)C1CCC1	0.0	0.0	not tested	not tested	0.0	0.0
SN00799568	CM0152A	CCN1CCN(CCNC(=O)c2cccc3N(CCc23)C(=O)Nc2cccc(C)c2)CC1	0.0	0.0	not tested	not tested	0.0	0.0
SN00799670	CM0194	CCC(=O)NCc1cnn(c1)c1ccc(cc1)C(=O)Nc1cccc(C)c1	0.0	0.0	not tested	not tested	0.0	0.0
SN00799681	CM0489	O=C(NCCC1=CCCCC1)c1cn(C)c2ncccc12	0.0	0.0	not tested	not tested	0.0	0.0
SN00799828	CL7444	CCN1CCCC(CC1)(C(=O)NCc1ccccc1F)c1ccccc1	0.0	0.0	not tested	not tested	0.0	0.0
SN00799838	CL7449	COc1cccc(c1)N1CCN(CC1)C(=O)CC1c2ncccc2C(=O)N1c1ccccc1	0.0	0.0	not tested	not tested	0.0	0.0
SN00799874	CM0272	COc1ccc2nc(cn2n1)C(=O)N1CCN(CC1)c1cccc(C)c1C	0.0	0.0	not tested	not tested	0.0	0.0
SN00800699	CM2362	Cc1nn(CC(=O)N2CCC(C2)c2onc(n2)c2ccncc2)c(C)c1	0.0	0.0	not tested	not tested	0.0	0.0
SN00800709	CL9902	Fc1ccc(CN2CCCC2c2nnc(C)[nH]2)c(F)c1	0.0	0.0	not tested	not tested	0.0	0.0
SN00800713	CL9902	O=C(NCc1ccccc1)N1CCCC1c1nnc([nH]1)c1ccccc1	0.0	0.0	not tested	not tested	0.0	0.0
SN00800737	CM2844	O=C(NC1CCCC1)N1CCCCC1c1nncn1CC1CC1	0.0	0.0	not tested	not tested	0.0	0.0
SN00800739	CM2844	O=C(c1ccncc1)N1CCCCC1c1nncn1Cc1ccccc1	0.0	0.0	not tested	not tested	0.0	0.0
SN00800750	CM2856	O=C(N1CCC(CC1)n1cnnc1c1ccccc1)c1ccco1	0.0	0.0	not tested	not tested	0.0	0.0
SN00800769	CM2856	COCc1nncn1C1CCN(CC1)C(=O)Cc1ccc(Cl)cc1	0.0	0.0	not tested	not tested	0.0	0.0
SN00800778	CM2882	O=C(Nc1cc(F)c(F)c(F)c1)N1CCC(C1)n1cnnc1C1CC1	0.0	0.0	not tested	not tested	0.0	0.0
SN00800780	CM2882	O=C(Nc1ccccc1C(F)(F)F)N1CCC(C1)n1cnnc1C1CC1	0.0	0.0	not tested	not tested	0.0	0.0
SN00800786	CL1193	NC(=O)c1nonc1N	0.0	0.0	not tested	not tested	0.0	0.0
SN00800800	CM1507	O=C(c1[nH]nc(c1)c1ccccc1)N1CCc2c(C1)[nH]c(nc2=O)c1ccccc1	0.0	0.0	not tested	not tested	0.0	0.0
SN00800803	CM1507	CCOC(=O)CCNC(=O)N1CCc2c(C1)[nH]c(nc2=O)c1ccccc1	0.0	0.0	not tested	not tested	0.0	0.0
SN00800819	CM1524	O=C(Cc1cscc1)N1CCc2c(C1)nc1cc[nH]n1c2=O	0.0	0.0	not tested	not tested	0.0	0.0
SN00800923	CM1886	O=C(N1CCC2(NCc3ccccc3N=C2NC2CCCC2)C1)c1n[nH]c2ccccc12	0.0	0.0	not tested	not tested	0.0	0.0
SN00800955	CM2379A	Fc1cc(F)cc(c1)S(=O)(=O)N1C[C@@H]2C[C@H](NC(=O)c3cccnc3)[C@H]1C2	0.0	0.0	not tested	not tested	0.0	0.0
SN00801011	CM2380B	O=C(c1ccc(cc1)OC(F)(F)F)N1C[C@@H]2C[C@H]1C[C@@H]2NS(=O)(=O)c1cccs1	0.0	0.0	not tested	not tested	0.0	0.0
SN00801025	CM2417	Cc1ccc(cc1C)S(=O)(=O)N1CCc2c(CC1)nc([nH]c2=O)N1CCOCC1	0.0	0.0	not tested	not tested	0.0	0.0
SN00801042	CM2417	Brc1ccc(cc1)CN1CCc2c(CC1)nc([nH]c2=O)N1CCOCC1	0.0	0.0	not tested	not tested	0.0	0.0
SN00801849	CM0986	Fc1cccc(c1)C(=O)Nc1ccc(nc1)N1CCN(Cc2ccccc2)C1=O	0.0	0.0	not tested	not tested	0.0	0.0
SN00801865	CM0998	COc1ccc(NC(=O)C2CCc3nc(sc3C2)N2CCN(CC2)C(=O)C)cc1F	0.0	0.0	not tested	not tested	0.0	0.0
SN00801884	CM0998	NC(=O)C1CCc2nc(sc2C1)N1CCN(CC1)C(=O)C1CCC1	0.0	0.0	not tested	not tested	0.0	0.0
SN00801896	CM0953	O=c1[nH]ncc(c1)N1CCCC(C1)C(=O)NC1CCCCCC1	0.0	0.0	not tested	not tested	0.0	0.0
SN00801913	CM0953	O=C(NCCN1CCCCCC1)C1CCCN(C1)c1cnn(C)c(=O)c1	0.0	0.0	not tested	not tested	0.0	0.0
SN00801944	CM0954	Cc1ccc(cc1)c1nccn2cc(nc12)C(=O)NCCN1CCCCC1	0.0	0.0	not tested	not tested	0.0	0.0
SN00801984	CM1047	CCn1c(nc2cccnc12)C1CCN(C1)C(=O)c1ccc(Br)cc1	0.0	0.0	not tested	not tested	0.0	0.0
SN00802143	CM2338A	CCC(=O)N1CC(C(=O)O)C2(CCOCC2)C1	0.0	0.0	not tested	not tested	0.0	0.0
SN00803008	CL9010	COc1cccc(CNC(=O)CCC(=O)Nc2nnc3ccccn23)c1	0.0	0.0	not tested	not tested	0.0	0.0
SN00803015	CL9010	COc1cccc2nnc(NC(=O)CCC(=O)NC3CCCCC3)n12	0.0	0.0	not tested	not tested	0.0	0.0
SN00803069	CL6946A	COc1cccc(CNC(=O)N2CCC(CC2)c2ccn3ncc(C(=O)N4CCCCC4)c3c2)c1	0.0	0.0	not tested	not tested	0.0	0.0
SN00803106	CL9312	COc1cc(ccc1OC)C1CC(=NC2(CCN(CC2)C(C)C)N1)c1ccccc1O	0.0	0.0	not tested	not tested	0.0	0.0
SN00803111	CL9312	COc1cc(ccc1OC)C1CC(=NC2(CCN(CC2)C(=O)C)N1)c1cc(Cl)ccc1O	0.0	0.0	not tested	not tested	0.0	0.0

Compound ID	Scaffold	Smiles	% Inhibition n=1		% Inhibition n=2		Average % Inhibition	
			24h	48h	24h	48h	24h	48h
SN00803131	CL9064	Fc1ccc(cc1)n1nc(C)c2sc(nc12)N1CCC(CC1)NC(=O)c1ccc(Cl)s1	0.0	0.0	not tested	not tested	0.0	0.0
SN00803255	CL8308	O=C(c1ccccc1)N1CCCC1c1c(C)noc1C	0.0	0.0	not tested	not tested	0.0	0.0
SN00803258	CL8307C	O=C(NC(C)(C)C)N1CCCC1c1onc(c1)C(C)C	0.0	0.0	not tested	not tested	0.0	0.0
SN00803271	CL0347	O=C(CSc1ccccc1)Nc1nnc[nH]1	0.0	0.0	not tested	not tested	0.0	0.0
SN00803272	CL0347	Clc1ccc(cc1)S(=O)(=O)N(C)CC(=O)Nc1[nH]cnn1	0.0	0.0	not tested	not tested	0.0	0.0
SN00799550	CL9527	COCCC(=O)Nc1c(nc2sc(nn12)N1CCCCC1)c1cccc(F)c1	0.0	0.0	not tested	not tested	0.0	0.0
SN00802161	CM1644	O=C(N1CCn2c(nnc2c2ccccc2)C1)c1cccs1	0.0	0.0	not tested	not tested	0.0	0.0
SN00802169	CM1644	COc1ccc(CC(=O)N2CCn3c(nnc3C)C2)cc1Cl	0.0	0.0	not tested	not tested	0.0	0.0
SN00802975	CL4666	O=C(NCCCN1CCCN(CC1)c1ccccc1)c1onc(n1)c1c[nH]c2ccccc12	0.0	0.0	not tested	not tested	0.0	0.0
SN00802994	CL9788	O=C(NCc1cccnc1)c1ccc2nc[nH]c(=O)c2c1	0.0	0.0	not tested	not tested	0.0	0.0
SN00781113	SC013130	O1CCN(CC1)c1nnc(SC2CCCC2)s1	0.0	0.0	not tested	not tested	0.0	0.0
SN00786991	CL7917B	O=C(NCc1noc(n1)c1cccs1)c1ccccc1C(F)(F)F	0.0	0.0	not tested	not tested	0.0	0.0
SN00793959	CL8308C	COc1c(OC)cc(cc1OC)NC(=O)N1CCCC1c1c(C)noc1C	0.0	0.0	not tested	not tested	0.0	0.0
SN00781068	SC012765	O=c1[nH]c(CCN2CCOc3ccccc3C2)nc2ccccc12	0.0	0.0	not tested	not tested	0.0	0.0
SN00782219	SC016288	CCOC(=O)c1ccc(cc1)S(=O)(=O)N1CCC(CC1)C1(C)NC(=O)NC1=O	0.0	0.0	not tested	not tested	0.0	0.0
SN00786913	CL9235	Cc1ccc(cc1)CNC(=O)c1cc(c2ccc(C)c(C)c2)n(n1)C1CCS(=O)(=O)C1	0.0	0.0	not tested	not tested	0.0	0.0
SN00788114	CL5794	O=S(=O)(NCc1ccc2OCOc2c1)c1ccc(cc1)n1cccn1	0.0	0.0	not tested	not tested	0.0	0.0
SN00789332	CL8003	Brc1ccc(NC(=O)CN(C)C(=O)c2ccc3c(nnn3C3CCCC3)c2)c(F)c1	0.0	0.0	not tested	not tested	0.0	0.0
SN00789379	CL7658	O=C(CN1C(=O)C2CCCC2=Nc2cc(C)c(C)cc12)N1CCC(CC1)Cc1ccccc1	0.0	0.0	not tested	not tested	0.0	0.0
SN00792782	CL7245	COc1ccc(cc1)n1nc(C(=O)N2CCCCC2c2cccnc2)c(=O)n(C)c1=O	0.0	0.0	not tested	not tested	0.0	0.0
SN00790424	CL7771B	COc1ccc(cc1)S(=O)(=O)N1CCN(CC)c2ncccc12	0.0	0.0	not tested	not tested	0.0	0.0
SN00796188	CL8797A	Cc1ccc(cc1)S(=O)(=O)N1CCC(CC1)C(=O)N1CCC(CC1)c1noc(n1)c1ccccc1	0.0	0.0	not tested	not tested	0.0	0.0
SN00787051	CL6690	OC(=O)[C@H]1NC(c2ccc(cc2)C(C)C)c2[nH]c3ccccc3c2C1	0.0	0.0	not tested	not tested	0.0	0.0
SN00788301	CL5552B	COc1cc(ccc1OC)c1noc(CN2CCCC(C2)C(=O)NCc2ccco2)n1	0.0	0.0	not tested	not tested	0.0	0.0
SN00788308	CL5552B	COc1cc(ccc1OC)c1noc(CN2CCCC(C2)C(=O)NC2CCCC2)n1	0.0	0.0	not tested	not tested	0.0	0.0
SN00789286	CL7913	C1CCC(C1)c1onc(n1)c1ccc(cc1)N1CCCC1	0.0	0.0	not tested	not tested	0.0	0.0
SN00789292	CL7913	CC1CCN(CC1)c1ccc(cc1)c1noc(n1)C(C)NS(=O)(=O)c1ccc(C)cc1	0.0	0.0	not tested	not tested	0.0	0.0
SN00789434	CL6708	Clc1cccc(c1)Nc1[nH]nnc1C(=O)Nc1ccc(C)c(Cl)c1	0.0	0.0	not tested	not tested	0.0	0.0
SN00789435	CL6708	CSc1cccc(c1)NC(=O)c1nn[nH]c1Nc1cccc(Cl)c1	0.0	0.0	not tested	not tested	0.0	0.0
SN00790548	CL6286	O=C(C1CCN(CC1)S(=O)(=O)c1ccc(o1)c1[nH]ncc1)N1CCN(CC1)c1ccccc1C	0.0	0.0	not tested	not tested	0.0	0.0
SN00791708	CL6874	O=C(NCC1CCCO1)c1noc(CN2CCOCC2)c1	0.0	0.0	not tested	not tested	0.0	0.0
SN00794058	CL8321	CCCNC(=O)CN1c2ccccc2NC2=C(C(=O)CC(C)(C)C2)C1c1ccccc1OC	0.0	0.0	not tested	not tested	0.0	0.0
SN00795908	CL8682A	COCCNC(=O)N1CCCC(C)(C1)C(=O)NCc1ccc(C)cc1	0.0	0.0	not tested	not tested	0.0	0.0
SN00797061	CL8111A	Cc1ccc(NC(=O)Cn2c(=O)nc(N3CCCCC3)c3ccccc23)c(F)c1	0.0	0.0	not tested	not tested	0.0	0.0
SN00784784	CL6193	CCN1CCN(CCCNc2nn3nnnc3c3ccccc23)CC1	0.0	0.0	not tested	not tested	0.0	0.0
SN00797506	CL9375	O=C(Cn1cccc1c1scc(n1)c1ccccc1)N1CCN(CC1)C(=O)c1ccco1	0.0	0.0	not tested	not tested	0.0	0.0
SN00798637	CL9614	COc1cccc(c1)c1onc(n1)C1CCCN(C1)C(=O)c1cc(C)ccc1C	0.0	0.0	not tested	not tested	0.0	0.0
SN00783588	CL2183	CCCC1=NN(C(=O)c2ccc(OC)cc2)C(O)(C1)C(F)(F)F	0.0	0.0	not tested	not tested	0.0	0.0
SN00785876	CL5632	COc1ccc(cc1)n1nnnc1C1CCN(CC1)S(=O)(=O)c1ccccc1S(=O)(=O)C	0.0	0.0	not tested	not tested	0.0	0.0
SN00785950	CL3359	COc1ccc(cc1)N1CC(CC1=O)c1nc2ccccc2[nH]1	0.0	0.0	not tested	not tested	0.0	0.0
SN00787019	CL7917B	O=C(NCc1noc(n1)c1cccs1)c1ccco1	0.0	0.0	not tested	not tested	0.0	0.0
SN00787024	CL9708	O=C(Nc1cc(ccc1C)c1nc2ccccc2[nH]1)C(C)C	0.0	0.0	not tested	not tested	0.0	0.0
SN00787103	CL6517	O=c1n(CCc2ccccc2)ccc2nc(ncc12)N1CCCCC1	0.0	0.0	not tested	not tested	0.0	0.0
SN00787112	CL7741	CCOC(=O)N1CCN(CC1)CC(=O)N1CCNC(=O)C1CC(=O)Nc1ccccc1C	0.0	0.0	not tested	not tested	0.0	0.0
SN00787851	CL3586B	CC(=O)N1CCC2(CC1)OC(=O)C(=C2C(=O)N1CCc2ccccc2C1)C	0.0	0.0	not tested	not tested	0.0	0.0
SN00790623	CL7819	O=C(Cn1nc2c3cc(nn3ccn2c1=O)c1ccccc1)N1CCCc2ccccc12	0.0	0.0	not tested	not tested	0.0	0.0

Compound ID	Scaffold	Smiles	% Inhibition n=1		% Inhibition n=2		Average % Inhibition	
			24h	48h	24h	48h	24h	48h
SN00794678	CL4756	O=C(NCCCN1CCC(CC1)Cc1ccccc1)c1cc2c(C)nccc2[nH]c1=O	0.0	0.0	not tested	not tested	0.0	0.0
SN00795117	CL3358	CN(Cc1ccccc1)/C(=N\S(=O)(=O)C)/Nc1ccccc1	0.0	0.0	not tested	not tested	0.0	0.0
SN00795832	CL9007A	O=C(Nc1ccc(C)c(F)c1)Nc1c([nH]c2ccc(Cl)cc12)c1ccncc1	0.0	0.0	not tested	not tested	0.0	0.0
SN00790160	CL7867	CCc1ccc(cc1)n1nc(ccc1=O)N1CCC(CC1)C(=O)NC1CC1	0.0	0.0	not tested	not tested	0.0	0.0
SN00783933	CL1247	COc1ccc(cc1C)S(=O)(=O)n1cc(C)nc1c1ccccc1	0.0	0.0	not tested	not tested	0.0	0.0
SN00783936	CL2614	NCC1=C(N)Oc2[nH]nc(C)c2C1c1ccc(C)cc1	0.0	0.0	not tested	not tested	0.0	0.0
SN00794312	CL5859	Cc1ccccc1NC(=O)c1cn2CCc3cccc(c1=O)c23	0.0	0.0	not tested	not tested	0.0	0.0
SN00798851	CL9718	CCc1csc(n1)c1ccc(s1)S(=O)(=O)NC(C)c1ccccc1	0.0	0.0	not tested	not tested	0.0	0.0
SN00798885	CL9733	CC(NC(=O)C1CCN(CC1)c1cnc(nc1)N1CCCCC1)c1ccccc1	0.0	0.0	not tested	not tested	0.0	0.0
SN00790798	CL8435	Cc1noc(c1)c1sc(C)c(c1)S(=O)(=O)N1CCN(CC1)C(=O)c1ccco1	0.0	0.0	not tested	not tested	0.0	0.0
SN00781259	SC013809	O=C(NC1CCCC1)CN1CCC(CC1)c1[nH]ncc1	0.0	0.0	not tested	not tested	0.0	0.0
SN00782378	SC007571	Cn1ncc(c1)C1CCCN1Cc1ncc(o1)C(C)(C)C	0.0	0.0	not tested	not tested	0.0	0.0
SN00782411	SC008114	O=C(C1CCCC1)N1CCCN(CC1)Cc1nnnn1C1CC1	0.0	0.0	not tested	not tested	0.0	0.0
SN00782485	SC009997	CCC(C)(C)C(=O)N1CCCN(CC1)C(=O)c1ccncc1	0.0	0.0	not tested	not tested	0.0	0.0
SN00782502	SC011213	C1CCN(CC1)CC1CCCN1CC1CCCO1	0.0	0.0	not tested	not tested	0.0	0.0
SN00789553	CL7310	O=C(NCCc1ccco1)C1CCn2c(C1)nc1ccccc21	0.0	0.0	not tested	not tested	0.0	0.0
SN00790746	CL8383B	CCCn1nnc2cc(ccc12)S(=O)(=O)N1CCCC1C(=O)N1CCN(CC1)c1ccccn1	0.0	0.0	not tested	not tested	0.0	0.0
SN00790751	CL8383B	Cc1ccc(cc1)CNC(=O)C1CCCN1S(=O)(=O)c1ccc2c(nnn2C(C)C)c1	0.0	0.0	not tested	not tested	0.0	0.0
SN00793048	CL2023A	CCOc1ccc(cc1OC)C1=Nc2ncnn2/C/1=N/Cc1ccccc1	0.0	0.0	not tested	not tested	0.0	0.0
SN00772544	SC013240	O=C(c1cccs1)N1CCC[C@H]1C(=O)N1CCN(CC1)S(=O)(=O)/C=C/c1ccccc1	0.0	17.9	not tested	not tested	0.0	17.9
SN00771579	SC008751	O=C(OCC(=O)N1c2ccccc2NC(=O)C21CCCC2)Cc1nn(C)c(=O)c2ccccc12	0.0	0.0	not tested	not tested	0.0	0.0
SN00784318	CL1729	Cc1ccc(cc1)Nc1c(nc2ccccn12)c1ccc(cc1)N1CCOCC1	0.0	0.3	not tested	not tested	0.0	0.3
SN00781544	SC014214	O=C(CCn1nncc1)N1CCCC(C1)c1scc(C)n1	0.0	0.0	not tested	not tested	0.0	0.0

Table S2. Activity of selected Compounds Australia Scaffold Library compounds in cytotoxicity and *Giardia* dose response assays.

Compounds were assessed for activity against *G. duodenalis* BRIS/91/HEPU/1279 and NFF in dose response assays from a maximum concentration of 10µM

Comp. ID	Scaffold	*G. duodenalis* IC_{50} (µM±SD)			NFF			SI	Bacterial Inhibition (% @ 50µM)			
		24h	48h	Comment	20µM inhibition (%±SD)	IC_{50} (µM±SD)	Comment	(48h Gd)	*S. aureus*	*E. coli*	*A. baumannii*	*K. pneumoniae*
SN00797640	CL9406	0.38±0.05	0.18±0.02		Not tested	>10		>55	-11.13	3.64	-5.15	-14.58
SN00776497	SC003542	0.25±0.27	0.08±0.09		Not tested	8.1	IC_{50} achieved in only one assay	101	55.84	5.45	19.63	8.25
SN00788467	CL3439	0.24±0.04	0.06±0.06		Not tested	>10		>166	99.07	3.73	3.19	-1.21
SN00798525	CL9569	1.21	1.17±0.00	24h IC_{50} achieved in only one assay. 48h IC_{50} nM SD	Not tested	>10		>8	22.17	-0.27	4.92	6.11
SN00776374	SC003155	1.76±0.35	1.01±0.29		100.4±0.2	-		<20	16.58	2.94	13.35	11.73
SN00790641	CL7819	2.84	3.41±2.02	24h IC_{50} achieved in only one assay.	40.4±1.1	-		>5	-6.6	0.25	4.72	6.73
SN00772378	SC012476	>10	4.79±1.45		1.2±1.0	-	IC_{50} undetermined	-	34.43	5.55	4.73	15.64
SN00785732	CL4038	7.64	7.68	IC_{50} achieved in only one assay	41.6±2.7	-		>2	2.41	0.23	9.69	9.49
SN00783960	CL3097	3.10±2.29	3.27±4.08		98.5±3.8	-		<6	95.3	11.99	6.26	19.87
SN00784346	CL5887	>10	7.66	48h IC_{50} n=1 7.66µM; n=2 >10µM	-1.1±0.8	-		>2	5.68	3.09	0.24	-2.01
SN00793100	CL2800	>10	>10		46.7±6.8	-	IC_{50} undetermined	-	10.13	1.35	9.06	7.52
SN00793512	CL6605B	8.74	8.63±1.91	24h IC_{50} achieved in only one assay.	35.2±2.5	-		>2	6.36	3.7	16.79	4.81
SN00796218	CL4659	>10	>10		29.7±10.3	-	IC_{50} undetermined	-	-2.23	3.03	4.35	3.63
SN00776738	SC005830	>10	>10		16.0±0.6	-	IC_{50} undetermined	-	-13.41	5.37	-4.28	6.87
SN00769982	SC001350	>10	9.59	48h IC_{50} n=1 >10 µM; n=2 9.59µM	-9.1±3.8	-	IC_{50} undetermined	-	3.41	6.08	2.57	15.58
SN00778038	SC007824	>10	6.39±2.80		8.4±4.7	-		>5	-9.58	-2.44	-8.73	-1.96
SN00792461	CL7138	5.35	7.82	24h &48h IC_{50} achieved in only one assay	21.3±27.1	-		>2	8.09	3.63	10.82	12.55
SN00799289	CL9921	>10	3.36±1.52		11.4±13.4	-		>5	3.06	1.89	4.74	14.37
SN00792764	CL7220	>10	>10	45.9 ± 5.4% @ 10µM 48h	16.6±20.4	-			-1.85	3.43	4.12	5.08
SN00792841	CL7285A	>10	4.75±4.02		-24.8±15.3	-		>4	-0.24	-0.09	2.57	7.3
SN00788144	CL5291	>10	>10		31.4±11.3	-	IC_{50} undetermined	-	-2.91	2.85	20.81	13.54
SN00782023	SC015404	6.80	6.51	24h &48h IC_{50} achieved in only one assay.	-6.3±3.6	-		>3	10	5.99	9.49	8.15
SN00798457	CL9915	>10	>10		16.3±20.4	-	IC_{50} undetermined	-	-16.61	1.98	-2.12	0.63
SN00788523	CL5077	>10	6.91	48h IC_{50} n=1 6.91µM; n=2 >10µM (44% inhibition @ 10µM)	-0.2±22.2	-		>2	1.08	2.37	1.85	22.06
SN00769776	SC001254	>10	>10		24.8±11.7	-	IC_{50} undetermined	-	6.45	8.92	23.41	32.13
SN00790147	CL8253	>10	>10		53.3±7.1	-	IC_{50} undetermined	-	11.9	-1.53	3.03	6.94
SN00783575	CL2800	>10	>10		26.9±2.5	-	IC_{50} undetermined	-	6.02	-4.12	15.1	16.34
SN00781107	SC013130	6.87±1.88	1.86±2.52		-19.8±44.9	-			4.13	2.28	3.43	10.57
SN00780011	SC010778	>10	5.48	48h IC_{50} n=1 5.48µM; n=2 >10µM	9.2±12.4	-		>3	11.74	8.95	10.46	16.44
SN00776314	SC003136	>10	9.16	48h IC_{50} n=1 9.16µM; n=2 >10µM 48.3% inhibition @10µM	24.0±26.8	-		>2	13.17	5.58	9.53	10.87
SN00798431	CL8499	>10	5.93±5.70		19.3±27.0	-		>3	13.35	-0.77	42.54	13.7
SN00794765	CL8472	>10	>10		23.1±8.6	-	IC_{50} undetermined	-	2.05	3.31	-0.4	1.61
SN00784168	CL3010	>10	4.58±4.9		75.1±0.3	-		<4	-10.68	3.98	-2.44	-3.07
SN00798315	CL9560	>10	4.74±6.40		-9.0±1.9	-		>4	1.07	2.55	1.7	9.56

Comp. ID	Scaffold	*G. duodenalis* IC_{50} (μM)			NFF			SI	Bacterial Inhibition (% @ 50μM)			
		24h	48h	Comment	20μM inhibition (%±SD)	IC_{50} (μM±SD)	Comment	(48hGd)	*S. aureus*	*E. coli*	*A. baumannii*	*K. pneumoniae*
SN00795179	CL8496	>10	>10		25.2±21.6	-	IC_{50} undetermined	-	4.52	2.96	30.27	11.39
SN00769770	SC001254	9.76	4.10±1.07	24h IC_{50} n=1 9.76μM; n=2 >10μM	-4.7±11.3	-		>4	13.26	-1.82	21.1	27.55
SN00798443	CL8499	>10	>10		19.0±12.8	-	IC_{50} undetermined	-	3.04	7.01	8.07	12.87
SN00795214	CL8595	>10	>10		22.8±13.3	-	IC_{50} undetermined	-	-0.3	-0.58	28.09	27.47
SN00788158	CL5291	>10	>10		14.9±7.5	-	IC_{50} undetermined	-	-16.86	-1.94	6.41	7.11
SN00798298	CL9560	>10	>10		2.7±30.4	-	IC_{50} undetermined	-	49.52	7.43	11.62	3.13
SN00784170	CL3010	>10	9.98	48h IC_{50} n=1 >10 μM; n=2 9.98μM	23.5±19.1	-		>2	8.16	2.07	5.37	11.47
SN00796169	CL5888	>10	>10	34.9 ± 15.5% @ 10μM 48h	12.5±0.2	-	IC_{50} undetermined	-	14.72	7.52	3.86	4.99
SN00795169	CL8496	>10	>10		60.3±22.0	-	IC_{50} undetermined	-	22.64	1.21	8.17	11.84
SN00799296	CL9921	>10	3.29	48h IC_{50} n=1 >10 μM; n=2 3.29μM	-25.0±17.0	-		>6	-3.5	3.24	5.31	6.89
SN00786504	CL8245B	>10	>10		9.7±12.4	-	IC_{50} undetermined	-	-1.52	-2.17	-7.58	-4.35
SN00779996	SC010778	>10	>10	35.8 ± 5.7% @ 10μM 48h	11.5±2.8	-	IC_{50} undetermined	-	20.28	8.93	6.95	8.88
Albendazole		0.07±0.02	0.04±0.02		-	1.1±0.5		>27	-0.15	2.47	-13.87	1.51
